# Identification of candidate genes and natural allelic variants for QTLs governing plant height in chickpea

**DOI:** 10.1038/srep27968

**Published:** 2016-06-20

**Authors:** Alice Kujur, Hari D. Upadhyaya, Deepak Bajaj, C. L. L. Gowda, Shivali Sharma, Akhilesh K. Tyagi, Swarup K. Parida

**Affiliations:** 1 National Institute of Plant Genome Research (NIPGR), Aruna Asaf Ali Marg, New Delhi 110067, India; 2 International Crops Research Institute for the Semi-Arid Tropics (ICRISAT), Patancheru 502324, Telangana, India

## Abstract

In the present study, molecular mapping of high-resolution plant height QTLs was performed by integrating 3625 *desi* genome-derived GBS (genotyping-by-sequencing)-SNPs on an ultra-high resolution intra-specific chickpea genetic linkage map (dwarf/semi-dwarf *desi* cv. ICC12299 x tall *kabuli* cv. ICC8261). The identified six major genomic regions harboring six robust QTLs (11.5–21.3 PVE), associated with plant height, were mapped within <0.5 cM average marker intervals on six chromosomes. Five SNPs-containing genes tightly linked to the five plant height QTLs, were validated based upon their high potential for target trait association (12.9–20.8 PVE) in 65 *desi* and *kabuli* chickpea accessions. The vegetative tissue-specific expression, including higher differential up-regulation (>5-fold) of five genes especially in shoot, young leaf, shoot apical meristem of tall mapping parental accession (ICC8261) as compared to that of dwarf/semi-dwarf parent (ICC12299) was apparent. Overall, combining high-resolution QTL mapping with genetic association analysis and differential expression profiling, delineated natural allelic variants in five candidate genes (encoding cytochrome-c-biosynthesis protein, malic oxidoreductase, NADH dehydrogenase iron-sulfur protein, expressed protein and *bZIP* transcription factor) regulating plant height in chickpea. These molecular tags have potential to dissect complex plant height trait and accelerate marker-assisted genetic enhancement for developing cultivars with desirable plant height ideotypes in chickpea.

Plant height is a vital yield-component trait. It affects crop’s performance, particularly lodging and subsequently grain yield and grain quality^1^,^2^. Crop plants of erect and a very tall habit tend to lodge as they approach maturity, whereas erect accessions with reduced height can avoid wind and rain damage, and are expected to be more lodging resistant. These desirable characteristics in turn enhance the potential of dwarf/semi-dwarf crop plant types to produce higher grain yield that are also more amenable to mechanical harvesting^1^,^2^. The reduction of plant height has therefore, been an important breeding and genomics research objective for many decades. Remarkably, the development of dwarf and semi-dwarf varieties of wheat and rice invoking “Green Revolution” during the 1960s and 1970s, is a famous historical milestone achieved in crop genetic improvement^3^,^4^. This has paved the way for plant breeders/molecular geneticists to substantially increase the grain yield through identification of variants (major dwarfing or semi-dwarfing genes) that reduce the plant height without adversely affecting the yield potential. However, tallness in plants has almost universally been shown to be dominant over dwarfness since Mendel’s times and thus developing plants with reduced height is still a challenging task.

Chickpea (*Cicer arietinum* L.) belonging to family Fabaceae, is a self-pollinated and diploid legume crop species with a genome size of ~740 Mb^5^,^6^. It is an annual, much branched and herbaceous crop with plant height mostly ranging from 30 to 70 cm. The genomes of two most common types of chickpea cultivars - *desi* and *kabuli* have been sequenced^7^,^8^,^9^. These available genomic resources have accelerated the process of NGS (next-generation sequencing)-based whole genome and transcriptome sequencing of numerous cultivated (*desi* and *kabuli*) and wild accessions as well as NGS-/array-based large-scale discovery and high-throughput genotyping of informative microsatellite and SNP (single nucleotide polymorphism) markers in natural and mapping populations of chickpea^10^,^11^,^12^,^13^,^14^,^15^,^16^,^17^,^18^. Consequently, these efforts have significantly driven the process of constructing high-density intra- and inter-specific genetic linkage maps and genetic/association mapping to identify QTLs (quantitative trait loci)/genes governing useful agronomic traits in chickpea^12^,^19^,^20^,^21^,^22^,^23^,^24^,^25^,^26^,^27^,^28^,^29^,^30^,^31^,^32^,^33^,^34^,^35^. One such most promising outcome includes the use of GBS (genotyping-by-sequencing) assay in discovery/genotyping of genome-wide SNPs in natural germplasm lines and mapping populations for understanding domestication and LD (linkage disequilibrium) pattern and constructing ultra-high density genetic linkage maps. This will further assist in fine mapping and high-resolution genome-wide association study (GWAS) to identify genomic loci (candidate genes) harboring major/minor QTLs regulating stress tolerance and yield-contributing traits for genomics-assisted crop improvement of chickpea^26^,^34^,^36^,^37^,^38^.

Considering the added-advantages of QTL and association mapping in quantitative dissection of complex traits, a number of genes underlying QTLs associated with plant height that rely on these approaches, have been identified in crop plants, including legumes^39^,^40^,^41^,^42^,^43^,^44^,^45^,^46^,^47^,^48^,^49^,^50^,^51^,^52^,^53^,^54^. Similar major efforts have been made in the past few years towards molecular mapping of low-resolution QTLs governing plant height on multiple intra- as well as inter-specific genetic linkage maps of chickpea^6^,^29^,^34^,^37^,^55^,^56^,^57^,^58^. However, none of these plant height QTLs could essentially be validated hitherto in diverse genetic backgrounds of chickpea accessions across multiple geographical locations (years). Consequently, none of the potential genes underlying plant height QTLs have been isolated and functionally validated/characterized through fine-mapping/map-based cloning and further functional genomic approaches to be effectively deployed in marker-assisted genetic enhancement of chickpea.

To expedite these useful genetic applications, the identification of functionally relevant genes harboring high-resolution QTLs at a genome-wide scale, by construction of SNP marker-based ultra-high resolution genetic linkage maps using GBS assay and their further validation through trait association analysis and differential expression profiling, at present is anticipated as an attractive strategy for chickpea genetic improvement. In this context, the present investigation employed aforesaid combinatorial approach to understand the complex genetic architecture of plant height trait for marker-assisted genetic enhancement with a definitive objective to develop high-yielding cultivars with desirable plant architecture (height) in chickpea.

## Results

### An ultra-high density intra-specific genetic linkage map accelerates molecular mapping of high-resolution PH QTLs in chickpea

The sequencing of 288-plex *Ape*KI libraries constructed from 275 RIL mapping individuals and parents (ICC 12299 x ICC 8261) using the reference *desi* genome-based GBS assay discovered 26785 high-quality SNPs differentiating the two parental accessions (as per Kujur *et al*.^26^). The use of genotyping information (present in parental accessions and all 275 RIL mapping individuals) of 4448 high-quality SNPs in linkage analysis mapped 3625 SNPs on eight LGs of an intra-specific genetic map based on their physical positions (bp) on corresponding *desi* chromosomes (Table S1). As per our previous study^26^, this GBS SNP-based ultra-high density intra-specific genetic map covered a total map-length of 714.1 cM with an average inter-marker distance of 0.20 cM.

**Figure 1:**
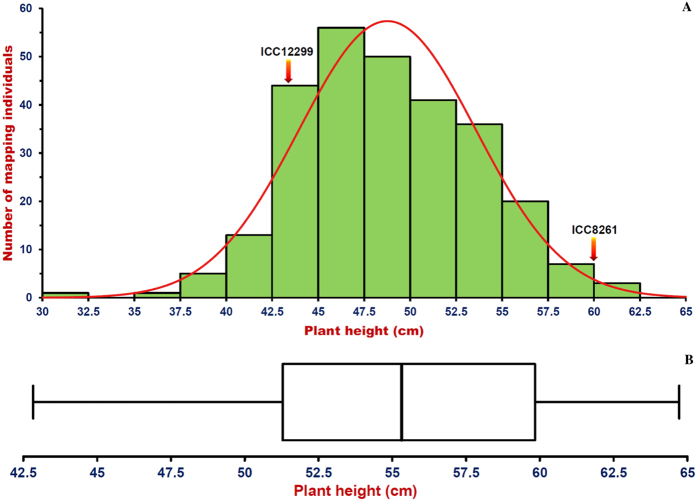
(**A**) Frequency distribution of plant height (PH cm) trait variation estimated in a 275 mapping population (ICC 12299 x ICC 8261) illustrated a goodness of fit to the normal distribution. (**B**) Boxplots illustrating the variation of PH trait among 65 *desi* and *kabuli* chickpea accessions. Box edges denote the lower and upper quantiles with median values in the middle of the box.

**Figure 2:**
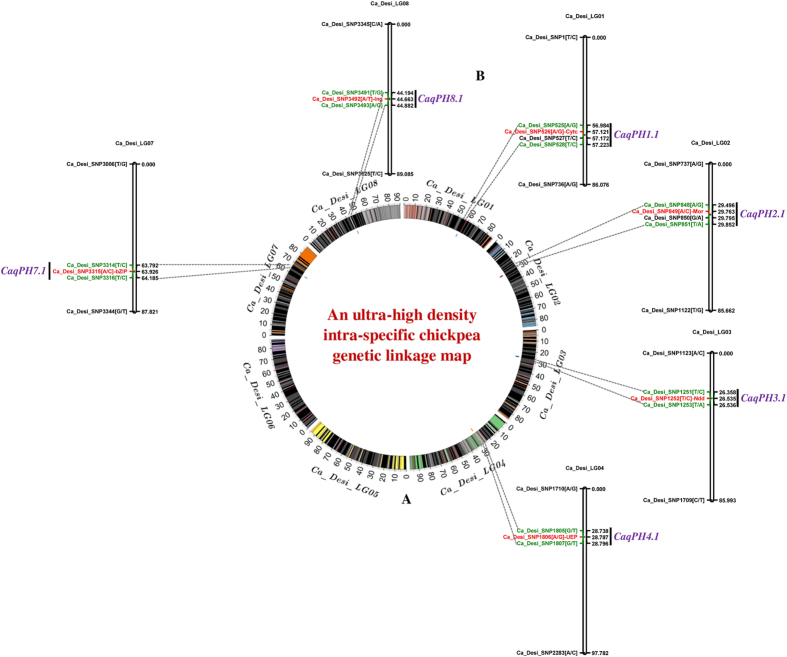
(**A**) An ultra-high resolution intra-specific genetic map (ICC 12299 x ICC 8261), constructed by integrating 3625 SNPs on eight LGs of chickpea, is illustrated by a Circos circular ideogram. The circles signifies the different genetic map length (cM) (spanning 10 cM uniform genetic distance intervals between bins) of eight LGs coded with diverse colours. (**B**) Molecular mapping of six major genomic regions underlying robust PH QTLs on six *desi* LGs/chromosomes (1, 2, 3, 4, 7 and 8). The SNPs exhibiting strong linkage with PH QTLs are highlighted with red colour. The SNPs flanking the PH QTL intervals are marked with green colour. The genetic (cM) distance and identities of the SNPs mapped on the LGs are denoted on the right and left side of LGs, respectively. The detail information regarding SNPs and QTLs are provided in the Table S1 and Table 1.

A significant difference (p ≤ 0.001) of PH trait [ranging 30.6–61.3 cm with mean ± standard deviation (SD) of 48.8 ± 4.8 and 82% H^2^ (broad-sense heritability)] in parental accessions and 275 RIL mapping individuals was observed across two years based on ANOVA. The coefficient of variation (CV) of PH trait was measured as 9.8%. A bi-directional transgressive segregation, including normal frequency distribution of PH trait in the generated RIL mapping population was evident (Fig. 1A). For molecular mapping of QTLs, the genotyping data of 3625 parental polymorphic *desi*-genome based SNPs mapped on an ultra-high density intra-specific genetic linkage map was correlated with phenotyping information of 275 RIL mapping individuals and parental accessions of chickpea. This identified six major genomic regions harboring six robust (6.1–10.7 LOD) QTLs (*qPH1.1*, *qPH2.1*, *qPH3.1*, *qPH4.1*, *qPH7.1* and *qPH8.1*) governing PH, which were mapped on six LGs (except LG5 and LG6) of chickpea (Table 1, Fig. 2A,B). The proportion of phenotypic variation explained (PVE) for PH trait by individual QTL ranged from 11.5–21.3%. These six QTLs in combination gave 35.8% PVE for PH trait. All these QTLs, validated across two geographical locations and years/seasons in the field and revealed consistent phenotypic expression (PVE: >10%) and major effects on PH traits, were thus considered as ‘robust PH QTLs’ in chickpea. The major genomic regions (from 0.058 cM on LG4 to 0.688 cM on LG8) underlying the robust QTLs covered with 20 SNPs, were mapped on six LGs (Table 1, Fig. 2B). These QTLs exhibited mostly the additive gene effects (1.7 to 4.5) signifying the effective contributions of ICC 12299 alleles at these loci for dwarf/semi-dwarf characteristics in chickpea. The identified major genomic regions harboring six robust PH QTLs as well as SNPs flanking/tightly linked to these PH QTLs mapped on an ultra-high density intra-specific genetic linkage maps were annotated based upon their structural and functional annotation information as documented in our previous study^26^. This analysis identified diverse coding (synonymous and non-synonymous) and non-coding regulatory and intronic SNPs in the five genes [encoding cytochrome c biosynthesis protein, malic oxidoreductase, NADH dehydrogenase iron-sulfur protein, unknown expressed protein and basic leucine zipper (*bZIP*) transcription factor] and one intergenic SNP tightly linked to the QTLs controlling PH trait in chickpea (Table 1, Fig. 2B).

### Trait association mapping to delineate candidate gene(s) regulating PH trait in chickpea

For genetic association mapping, the SNPs (flanking/linked to PH QTLs) mapped at the six major PH QTL regions of an ultra-high density intra-specific genetic linkage map were genotyped in 65 *desi* and *kabuli* chickpea accessions (Table S2). The SNP genotyping information was integrated with PH field phenotyping data (varying 42.8–64.7 cm with mean ± standard deviation of 55.1 ± 5.7 and 80% H^2^) of 65 accessions (Fig. 1B). The association analysis based on CMLM and false discovery rate (FDR) correction (minimizing the confounding effect of population structure) detected five SNPs-containing genes revealing significant association (1.3 × 10^−5^ to 2.1 × 10^−6^ P with 12.9 to 20.8% R^2^) with PH trait in chickpea (Table 1). These PH-associated SNPs-carrying genes localized at the major genomic regions harboring five robust QTLs were also found to be tightly linked with PH QTLs based on high-resolution QTL mapping (Table 1). Henceforth, five functionally relevant SNP loci-carrying genes (encoding cytochrome c biosynthesis protein, malic oxidoreductase, NADH dehydrogenase iron-sulfur protein, unknown expressed protein and *bZIP* transcription factor) validated both by high-resolution QTL and association mapping, were considered as candidates for plant height regulation in chickpea. Interestingly, two upstream regulatory (URR) and intronic SNPs (A/G and A/C)-containing cytochrome c biosynthesis protein-coding gene and *bZIP* transcription factor gene, respectively, tightly linked to the PH QTLs (*qPH1.1* and *qPH7.1*) exhibited strong association (average 20% PVE) with plant height based on QTL mapping and trait association analysis in chickpea.

### Expression profiling of PH-associated genes

**Figure 3:**
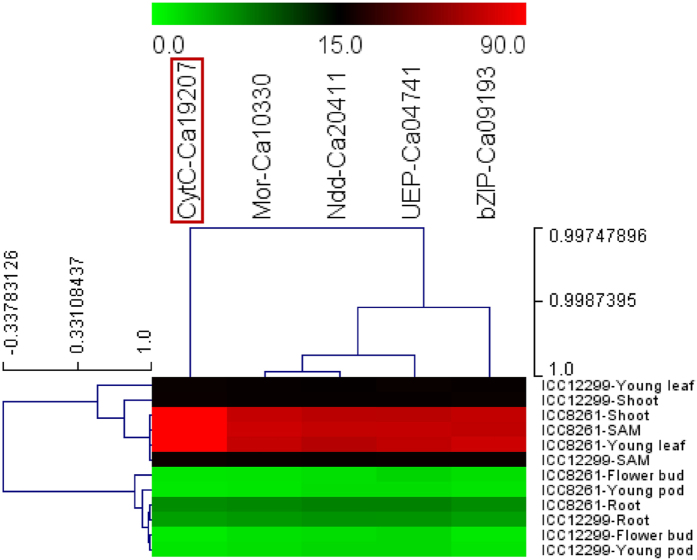
Hierarchical cluster display depicting the differential expression profiles of five PH-associated genes (validated by QTL mapping and association analysis) in vegetative and reproductive tissues (root, shoot, young leaf, shoot apical meristem, flower bud and young pod) of tall (ICC 8261) and dwarf/semi-dwarf (ICC 12299) parental accessions of an intra-specific mapping population (ICC 12299 x ICC 8261) using quantitative RT-PCR assay. The colour scale at the top represents the average log signal expression values of genes in different tissues; in which green, black and red color signify low, medium and high level of expression, respectively. The gene revealing pronounced differential up-regulation especially in shoot, young leaf and shoot apical meristem of tall accessions is highlighted with red box. The details regarding genes are provided in the Table 1. The tissues and genes utilized for expression profiling are illustrated on the right and top side of expression map, respectively. To normalize the expression values across diverse tissues of accessions, an endogenous control *elongation factor-1 alpha* was utilized. *CytC*: *Cytochrome C*, *Mor*: *Malic oxidoreductase*, *Ndd*: *NADH dehydrogenase*, UEP: Unknown expressed protein and *bZIP*: *basic leucine zipper*.

To determine the differential gene regulatory expression patterns, five PH-associated SNPs-containing genes delineated at the major genomic regions harboring robust PH QTLs (by high-resolution QTL and association mapping) were utilized in expression analysis. The differential expression profiles of these genes in diverse vegetative and reproductive tissues (root, shoot, young leaf, shoot apical meristem, flower bud and young pod) of tall (ICC 8261) and dwarf/semi-dwarf (ICC 12299) parental accessions of an intra-specific mapping population (ICC 12299 x ICC 8261) were determined (Fig. 3). This revealed vegetative tissue-specific expression (as compared to reproductive tissues) as well as up-regulation (>5-folds) of five SNPs-carrying genes especially in shoot, young leaves and shoot apical meristem of tall parental accession (ICC 8261) than that of dwarf/semi-dwarf parent (ICC 12299). Notably, two strong PH-associated URR and intronic SNPs-containing vegetative tissue-specific cytochrome c biosynthesis protein-coding gene and *bZIP* transcription factor tightly linked to the PH QTLs (*qPH1.1* and *qPH7.1*) exhibited pronounced up-regulation (>6–7 folds) in shoots, young leaves and shoot apical meristems of tall parental accession (ICC 8261) as compared to that of dwarf/semi-dwarf parent (ICC 12299) (Fig. 3). Therefore, natural allelic variants-containing five potential candidate genes validated by an integrated genomic approach encompassing QTL and association mapping and expression profiling, were selected as target candidates for quantitative dissection of complex plant height trait in chickpea (Table 1).

## Discussion

The implication of GBS-assay in simultaneous genome-wide mining and high-throughput genotyping of high-quality and non-erroneous SNPs in diverse advanced generation intra- and inter-specific mapping populations for construction of ultra-high resolution genetic linkage maps have been demonstrated in chickpea^26^,^36^,^37^. A 3265 genome-wide SNP markers-led ultra-high density intra-specific genetic linkage map constructed by us using a 275 chickpea RIL mapping population (ICC 12299 x ICC 8261) exhibited a much higher mean map-density (with a low average inter-marker distance 0.20 cM). This is highly saturated and/or comparable to the latest available high-resolution inter- and intra-specific genetic linkage maps (0.16–3.68 cM) in chickpea^14^,^22^,^26^,^29^,^35^,^36^,^37^,^59^,^60^,^61^. Therefore, an ultra-high density intra-specific genetic linkage map constructed in the present study has efficacy in identification and fine-mapping (map-based cloning) of major/minor QTLs governing traits of agricultural importance in chickpea.

The use of ultra-high density intra-specific genetic linkage map generated in our study as a reference identified and mapped six major genomic regions underlying six robust QTLs (11.5–21.3% PVE with 35.8% combined PVE) governing PH traits in chickpea. To ascertain the robustness and novelty of the identified PH QTLs, the major genomic regions underlying six robust PH QTLs were compared/correlated with that of previous QTL mapping studies based on correspondence of physical positions of SNPs covering (flanked/linked) the target QTL intervals^37^,^58^. None of these six QTLs were found to be congruent with previous QTL mapping studies involving diverse intra- and inter-specific mapping populations of chickpea. Henceforth, novelty and population-specific characteristics of the identified six major and robust QTLs governing PH trait was apparent in chickpea. A number of QTL mapping studies involving high resolution intra- and inter-specific genetic linkage maps as references/anchors have been undertaken for rapid molecular mapping as well as fine mapping/map-based cloning of major genes/QTLs governing multiple stress tolerance- and yield-component traits to expedite marker-assisted genetic enhancement in chickpea^26^,^28^,^29^,^35^,^36^,^37^,^59^. Interestingly, with the use of an ultra-high density genetic linkage map as a reference in the present investigation, the major PH QTLs significantly scaled-down into the marker intervals of 0.058–0.688 cM (average <0.5 cM) in chickpea. This is much lower than that documented (1.05–3.6 cM) till date particularly for PH QTLs in different QTL mapping studies of chickpea^37^,^59^. The comparative analysis between past and present QTL mapping study suggests the efficacy of these identified major and high-resolution robust PH QTLs for understanding the genetic inheritance pattern and in dissection of complex plant height trait of chickpea. Therefore, the SNPs derived from diverse coding and non-coding sequence components of genes and/or intergenic regions tightly linked to the six major PH QTLs can be deployed in marker-assisted breeding to select cultivars with desirable plant height for genetic improvement in chickpea. The subsequent validation of the PH QTLs in multiple genetic backgrounds/environments along with their fine mapping/map-based cloning will be helpful to select favorable and superior molecular tags for accelerating genomics-assisted crop improvement in chickpea.

Interestingly, the integration of high-resolution QTL and association mapping validated informative SNPs-carrying five candidate genes harboring five major PH QTLs reflecting the robustness and reliability of identified molecular tags for their immense deployment in marker-assisted genetic improvement of chickpea. The efficacy of these genes in regulating PH trait was further ascertained by their vegetative tissue-specific expression and pronounced up-regulation in tall than that of dwarf/semi-dwarf mapping parental accessions. The added-advantage of such integrated genomics-assisted breeding strategy for delineation of potential candidate genes regulating important yield-contributing traits have been demonstrated in chickpea^23^,^24^,^25^,^26^,^31^,^32^,^33^,^35^,^38^. The SNPs-carrying genes (encoding cytochrome c biosynthesis protein, NADH dehydrogenase iron-sulfur protein and malic oxidoreductase) regulating PH trait scaled-down by the integrated genomic approach, are evolutionarily conserved key components of mitochondrial respiratory complex and known to play a central role in classical electron transport chain (ETC) and tricarboxylic acid (TCA) cycle for regulating mitochondrial organization and respiration in crop plants^62^,^63^,^64^,^65^,^66^,^67^. The meristem-specific expression, including differential expression and accumulation of transcripts of these mitochondrial genes affect the cellular events related to mitochondrial respiration and photosynthetic activity by providing energy for biosynthesis and biomass storage during plant growth and development^64^,^66^,^68^. The regulation of these genes at transcriptional and post-transcriptional levels in different tissues is known to stimulate the mitochondrial respiratory apparatus of plants by assisting in shaping the organ development^69^. Another PH-associated basic leucine zipper functional domain-containing *bZIP* transcription factor gene regulates plant morphology by modulating the endogenous contents of gibberellins (GAs) and GA homeostasis. Interestingly, the reduction of GAs by down-regulation of genes (specifically *GA20-oxidase* and *GA3*) encoding GA biosynthetic enzymes severely inhibit the process of cell elongation/expansion and growth of stem internodes resulting in dwarf phenotype in multiple monocot and dicot plant species. Such GA response modulators, including green revolution genes- *Rht* (reduced height), *d8* (dwarf 8), *GAI* (gibberellin insensitive) and *Sd1* (semi-dwarf) governing GA signaling as well as plant height (dwarfism) have been identified and functionally characterized in wheat, maize, *Arabidopsis* and rice, respectively^3^,^4^,^70^,^71^. The correlation and interaction between mitochondrial ETC-mediated cytochrome c genes and GA synthesis-controlling *bZIP* transcription factors basing upon their similar expression characteristics in diverse tissues/developmental stages of crop plants is well deciphered. This indicates the involvement of these genes in an analogous functional regulatory pathway underlying both GA biosynthesis and mitochondrial respiration for maintaining growth and development of organs, including plant height^72^,^73^. Collectively, the PH-associated natural allelic variants-containing five genes delineated in the present study by integrating QTL and association mapping with differential expression profiling apparently play a crucial role in GA biosynthesis and mitochondrial respiration in crop plants. Henceforth, these molecular tags have got functional significance for understanding the regulation and quantitative dissection of complex plant height (dwarfism) trait in chickpea. The PH-associated superior natural allelic variants identified in the potential candidate genes once validated by translational genomic approaches (marker-assisted breeding and transgenics) can be deployed in crop genetic enhancement for developing genetically tailored improved chickpea cultivars with desirable plant height and architecture.

## Methods

### Generation and phenotyping of mapping population for plant height

An intra-specific F_7_ RIL mapping population (ICC 12299 x ICC 8261) consisting of 275 individuals was generated using a single seed descent method. The mapping parents ICC 12299 and ICC 8261 are dwarf/semi-dwarf and tall *desi* and *kabuli* chickpea (*C. arietinum*) accessions with plant height of 43 and 60 cm, respectively. These RIL mapping individuals along with parental accessions were grown in the field (following alpha-lattice design with two replications) for two consecutive years (2012–2013) at two diverse geographical locations of India. Subsequently, the RILs and parents were phenotyped individually for plant height (PH). The PH was documented by measuring the mean canopy height (cm) of 10 representative plants (selected from each RILs) from soil surface at the time of their flower ending/pod setting initiation stage. The diverse statistical parameters, including broad-sense heritability (H^2^%), mean, standard deviation, coefficient of variation (CV%), frequency distribution and analysis of variance (ANOVA) of PH trait in a RIL mapping population were determined using SPSS v17.0 and following Saxena *et al*.^31^ and Bajaj *et al*.^33^.

### Mining and genotyping of genome-wide GBS-SNPs

The genomic DNA isolated from 275 RIL mapping individuals and two parental accessions (as biological replicates) was used to construct the 288-plex (3 × 96-plex) GBS libraries as per Elshire *et al*.^74^ and Kujur *et al*.^25^,^26^. The sequencing of these pooled libraries were performed by Illumina HiSeq2000 NGS platform. The sequencing data generated were submitted to a publicly accessible NCBI-SRA (sequence read achieve) database (http://www.ncbi.nlm.nih.gov/sra) with accession number under BioProject PRJNA320682. The raw FASTA 100-bp single end sequence reads were processed for quality assessment, and subsequently high-quality sequences were filtered and mapped onto the reference genome using TASSEL 5.0 GBS Pipeline v2. The sequence reads with ≥10 *phred* Q-score across first 72-bp nucleotide sequence were considered high-quality, which were further ascertained through FASTQC v0.10.1. The retained good-quality sequences were de-multiplexed using unique barcodes tagged with each mapping parents and RIL individuals. The high-quality de-multiplexed reads of each mapping parents and RIL individuals were mapped against *desi* chickpea genome^7^ using Burrows-Wheeler Alignment (BWA) tool with default parameters following Kujur *et al*.^25^,^26^. The SNPs were discovered among mapping parents and RIL individuals using SAMtools. To discover the high-quality SNPs, the imputation of missing SNP allele data was performed using haplotype probability method of fastPHASE^26^. The SNPs with base-quality ≥20 supported by minimum sequence read depth of 10 (< 10% missing data per individual and 100% reproducibility) in RIL mapping individuals and parental accessions were considered as high-quality SNPs.

### Construction of an intra-specific genetic linkage map

The genotyping data of reference *desi* genome-based high-quality GBS-SNPs revealing polymorphism between parental accessions (ICC 12299 and ICC 8261) and 275 RIL mapping individuals were analyzed using JoinMap 4.1 (as per Bajaj *et al*.^33^ and Kujur *et al*.^26^). At a higher LOD (logarithm of odds) threshold of >10.0, the SNPs were integrated [based on centiMorgan (cM) genetic distances] into eight linkage groups (LGs) of an intra-specific genetic map. The RECORD algorithm of RECORD_WIN was employed to select the best marker-ordered genetic linkage maps with shortest map distances (cM). The LGs with genetically mapped SNPs were designated (LG1 to LG8) according to their corresponding physical positions (bp) on the chromosomes of *desi* chickpea genome.

### QTL mapping

The reference genome-based GBS-SNP genotyping information was integrated with the genetic linkage map data of SNPs mapped on eight LGs of an intra-specific genetic map and PH field phenotyping data of 275 RIL mapping individuals and parental accessions using composite interval mapping functions of QTL Cartographer v2.5 and MapQTL v6.0. The novel genomic regions underlying the major QTLs associated with PH were identified and mapped on LGs at a significant LOD threshold score of ≥5.0 (1000 permutation at a p < 0.05). The identified QTLs that validated across multiple environments (locations)/years were considered as ‘robust QTLs’ governing PH trait^31^. The phenotypic variation explained (PVE %) by QTLs and their additive effects (estimated by favorable alleles with parental origin) on PH trait were determined. The structural and functional annotation of genome-based SNPs, mapped on the eight LGs of the intra-specific genetic maps at target PH QTL regions, were performed according to the genome annotation (CGAP_v1.0) information (GFF) of *desi* chickpea^7^ following Kujur *et al*.^25^,^26^.

### Association analysis

The SNPs (flanking/linked to PH QTLs) exhibiting differentiation between dwarf (ICC 12299) and tall (ICC 8261)-PH types parental accessions, mapped at the major QTL intervals, were genotyped in 65 *desi* and *kabuli* chickpea accessions (selected from the study of Kujur *et al*.^25^,^38^) using the Illumina GoldenGate SNP genotyping assay (as per Bajaj *et al*.^33^). The high-resolution LD (linkage disequilibrium)-based association analysis (compressed mixed model approach of EMMA) was performed following Saxena *et al*.^31^, Thudi *et al*.^34^ and Kujur *et al*.^38^. The SNPs exhibiting strong association (R^2^: correlation potential of significant SNPs with traits) with PH trait at a significant cut-off P ≤ 10^−5^ were selected. This was performed by eliminating the confounding effect of population structure and implementing the Bonferroni correction of P-value (based on multiple comparisons) for each PH-associated SNPs at the false discovery rate (FDR) of 5% significance.

### Differential gene expression profiling

The high PH-associated genes with SNPs delineated at the major genomic regions harboring robust PH QTLs (validated by QTL and association mapping) were selected to determine their regulation by differential expression profiling. The RNA isolated from diverse vegetative and reproductive tissues (root, shoot, young leaf, shoot apical meristem, flower bud and young pod) of tall (ICC 8261) and dwarf/semi-dwarf (ICC 12299) parental accessions was amplified using the gene-specific primers through quantitative RT-PCR assay (as per Bajaj *et al*.^32^).

## Additional Information

**How to cite this article**: Kujur, A. *et al*. Identification of candidate genes and natural allelic variants for QTLs governing plant height in chickpea. *Sci. Rep.* **6**, 27968; doi: 10.1038/srep27968 (2016).

## Supplementary Material

Supplementary Information

## Figures and Tables

**Table 1:** Candidate genes harbouring major QTLs regulating PH identified by QTL mapping and genetic association analysis in chickpea.

QTL mapping						Association mapping		SNPs tightly linkedto QTLs (trait-associated SNPs)	Structuralannotation	Functional annotation
Linkagegroups(LGs)	QTLsidentities	Marker intervalswith geneticpositions (cM)	LOD	Phenotypicvariationexplained (PVE%)by QTLs	Additiveeffects	P	R^2^ (%)			
*Ca_Desi_LG01*	*qPH1.1*	0.239: *Ca_Desi_SNP525* (56.984) to *Ca_Desi_SNP528* (57.223)	10.7	21.3	4.5	2.1 × 10^−6^	20.8	*Ca_Desi_SNP526* (A/G)	Ca19207 (URR)	Cytochrome C biosynthesis protein
*Ca_Desi_LG02*	*qPH2.1*	0.356: *Ca_DesiSNP848* (29.496) to *Ca_Desi_SNP851* (29.852)	8.6	15.9	3.9	4.2 × 10^−6^	17.9	*Ca_Desi_SNP849* (A/C)	Ca10330 (CDS-Syn)	Mitochondrial NAD-dependent malic enzyme (Malic oxidoreductase)
*Ca_Desi_LG03*	*qPH3.1*	0.178: *Ca_DesiSNP1251* (26.358) to *Ca_Desi_SNP1253* (26.536)	8.1	16.2	2.8	1.3 × 10^−5^	16.7	*Ca_Desi_SNP1252* (T/C)	Ca20411 (DRR)	NADH dehydrogenase [ubiquinone] iron-sulfur protein 3
*Ca_Desi_LG04*	*qPH4.1*	0.058:*Ca_Desi_SNP1805* (28.738) to *Ca_Desi_SNP1807* (28.796)	7.8	12.4	2.5	1.9 × 10^−5^	12.9	*Ca_Desi_SNP1806* (A/G)	Ca04741 (Intron)	Unknown expressed protein
*Ca_Desi_LG07*	*qPH7.1*	0.393: *Ca_Desi_SNP3314* (63.792) to *Ca_Desi_SNP3316* (64.185)	9.5	20.5	2.1	3.9 × 10^−6^	20.3	*Ca_Desi_SNP3315* (A/C)	Ca09193 (Intron)	Basic leucine zipper (*bZIP*) transcription factor 60
*Ca_Desi_LG08*	*qPH8.1*	0.688: *Ca_Desi_SNP3491* (44.194) to *Ca_Desi_SNP3493* (44.882)	6.1	11.5	1.7	4.8 × 10^−5^	–	*Ca_Desi_SNP3492* (A/T)	Intergenic	–
