# Supplementary material for: Identification of candidate genes and natural allelic variants for QTLs governing plant height in chickpea
Source: Sci Rep. 2016 Jun 20;6:27968. doi: 10.1038/srep27968 (PMC4913251; doi:10.1038/srep27968)
Supplement: Supplementary Information [file srep27968-s1.pdf]

# Identification of candidate genes and natural allelic variants for QTLs governing plant height in chickpea

**Alice Kujur[1], Hari D. Upadhyaya[2], Deepak Bajaj[1], C.L.L. Gowda[2], Shivali Sharma[2], Akhilesh K. Tyagi[1], Swarup K. Parida[1]***

[1]National Institute of Plant Genome Research (NIPGR), Aruna Asaf Ali Marg, New Delhi 110067, India

[2]International Crops Research Institute for the Semi-Arid Tropics (ICRISAT), Patancheru 502324, Telangana, India

*Corresponding author

**Table S1: A 3625 high-quality *desi* genome-derived GBS-SNPs mapped on an ultra-high density intra-specific genetic linkage map of chickpea**

| **SNP IDs** | **SNPs** | **Linkage Group (LGs)** | **Genetic positions (cM) of mapped SNPs** |
|---|---|---|---|
| Ca_Desi_SNP1 | [T/C] | *Ca_Desi_LG01* | 0.000 |
| Ca_Desi_SNP2 | [C/G] | *Ca_Desi_LG01* | 0.146 |
| Ca_Desi_SNP3 | [C/T] | *Ca_Desi_LG01* | 0.559 |
| Ca_Desi_SNP4 | [A/C] | *Ca_Desi_LG01* | 0.856 |
| Ca_Desi_SNP5 | [A/G] | *Ca_Desi_LG01* | 0.923 |
| Ca_Desi_SNP6 | [G/T] | *Ca_Desi_LG01* | 1.215 |
| Ca_Desi_SNP7 | [A/C] | *Ca_Desi_LG01* | 2.584 |
| Ca_Desi_SNP8 | [C/T] | *Ca_Desi_LG01* | 2.603 |
| Ca_Desi_SNP9 | [G/C] | *Ca_Desi_LG01* | 3.543 |
| Ca_Desi_SNP10 | [T/G] | *Ca_Desi_LG01* | 4.062 |
| Ca_Desi_SNP11 | [A/C] | *Ca_Desi_LG01* | 4.585 |
| Ca_Desi_SNP12 | [T/G] | *Ca_Desi_LG01* | 5.086 |
| Ca_Desi_SNP13 | [A/G] | *Ca_Desi_LG01* | 5.238 |
| Ca_Desi_SNP14 | [C/A] | *Ca_Desi_LG01* | 5.410 |
| Ca_Desi_SNP15 | [A/T] | *Ca_Desi_LG01* | 5.540 |
| Ca_Desi_SNP16 | [T/C] | *Ca_Desi_LG01* | 5.562 |
| Ca_Desi_SNP17 | [T/C] | *Ca_Desi_LG01* | 7.518 |
| Ca_Desi_SNP18 | [C/T] | *Ca_Desi_LG01* | 7.553 |
| Ca_Desi_SNP19 | [C/A] | *Ca_Desi_LG01* | 8.101 |
| Ca_Desi_SNP20 | [A/C] | *Ca_Desi_LG01* | 8.356 |
| Ca_Desi_SNP21 | [T/C] | *Ca_Desi_LG01* | 8.403 |
| Ca_Desi_SNP22 | [A/G] | *Ca_Desi_LG01* | 8.844 |
| Ca_Desi_SNP23 | [C/A] | *Ca_Desi_LG01* | 9.458 |
| Ca_Desi_SNP24 | [T/C] | *Ca_Desi_LG01* | 9.870 |
| Ca_Desi_SNP25 | [T/G] | *Ca_Desi_LG01* | 10.180 |
| Ca_Desi_SNP26 | [T/G] | *Ca_Desi_LG01* | 10.209 |
| Ca_Desi_SNP27 | [T/C] | *Ca_Desi_LG01* | 10.570 |
| Ca_Desi_SNP28 | [T/C] | *Ca_Desi_LG01* | 10.835 |
| Ca_Desi_SNP29 | [T/G] | *Ca_Desi_LG01* | 10.862 |
| Ca_Desi_SNP30 | [A/G] | *Ca_Desi_LG01* | 12.036 |
| Ca_Desi_SNP31 | [A/C] | *Ca_Desi_LG01* | 12.317 |
| Ca_Desi_SNP32 | [T/C] | *Ca_Desi_LG01* | 12.508 |
| Ca_Desi_SNP33 | [T/C] | *Ca_Desi_LG01* | 12.618 |
| Ca_Desi_SNP34 | [T/C] | *Ca_Desi_LG01* | 12.694 |
| Ca_Desi_SNP35 | [T/G] | *Ca_Desi_LG01* | 13.178 |
| Ca_Desi_SNP36 | [T/C] | *Ca_Desi_LG01* | 13.207 |
| Ca_Desi_SNP37 | [T/G] | *Ca_Desi_LG01* | 13.525 |
| Ca_Desi_SNP38 | [T/C] | *Ca_Desi_LG01* | 13.649 |
| Ca_Desi_SNP39 | [A/G] | *Ca_Desi_LG01* | 13.710 |
| Ca_Desi_SNP40 | [A/G] | *Ca_Desi_LG01* | 13.733 |
| Ca_Desi_SNP41 | [T/C] | *Ca_Desi_LG01* | 13.930 |
| Ca_Desi_SNP42 | [G/A] | *Ca_Desi_LG01* | 14.480 |
| Ca_Desi_SNP43 | [A/G] | *Ca_Desi_LG01* | 15.473 |
| Ca_Desi_SNP44 | [T/A] | *Ca_Desi_LG01* | 15.641 |
| Ca_Desi_SNP45 | [C/T] | *Ca_Desi_LG01* | 15.646 |

| SNP IDs | SNPs | Linkage Group (LGs) | Genetic positions (cM) of mapped SNPs |
|---|---|---|---|
| Ca_Desi_SNP46 | [A/T] | *Ca_Desi_LG01* | 15.953 |
| Ca_Desi_SNP47 | [T/C] | *Ca_Desi_LG01* | 16.168 |
| Ca_Desi_SNP48 | [C/A] | *Ca_Desi_LG01* | 16.247 |
| Ca_Desi_SNP49 | [A/C] | *Ca_Desi_LG01* | 16.247 |
| Ca_Desi_SNP50 | [A/G] | *Ca_Desi_LG01* | 16.385 |
| Ca_Desi_SNP51 | [G/T] | *Ca_Desi_LG01* | 16.460 |
| Ca_Desi_SNP52 | [C/T] | *Ca_Desi_LG01* | 16.521 |
| Ca_Desi_SNP53 | [T/G] | *Ca_Desi_LG01* | 16.666 |
| Ca_Desi_SNP54 | [A/C] | *Ca_Desi_LG01* | 16.737 |
| Ca_Desi_SNP55 | [C/A] | *Ca_Desi_LG01* | 16.820 |
| Ca_Desi_SNP56 | [A/G] | *Ca_Desi_LG01* | 16.833 |
| Ca_Desi_SNP57 | [G/A] | *Ca_Desi_LG01* | 17.345 |
| Ca_Desi_SNP58 | [A/T] | *Ca_Desi_LG01* | 17.452 |
| Ca_Desi_SNP59 | [T/C] | *Ca_Desi_LG01* | 17.519 |
| Ca_Desi_SNP60 | [A/C] | *Ca_Desi_LG01* | 17.522 |
| Ca_Desi_SNP61 | [G/T] | *Ca_Desi_LG01* | 18.048 |
| Ca_Desi_SNP62 | [A/G] | *Ca_Desi_LG01* | 18.485 |
| Ca_Desi_SNP63 | [G/T] | *Ca_Desi_LG01* | 18.616 |
| Ca_Desi_SNP64 | [T/C] | *Ca_Desi_LG01* | 18.686 |
| Ca_Desi_SNP65 | [T/G] | *Ca_Desi_LG01* | 18.796 |
| Ca_Desi_SNP66 | [A/G] | *Ca_Desi_LG01* | 19.017 |
| Ca_Desi_SNP67 | [T/C] | *Ca_Desi_LG01* | 19.215 |
| Ca_Desi_SNP68 | [T/G] | *Ca_Desi_LG01* | 19.319 |
| Ca_Desi_SNP69 | [G/C] | *Ca_Desi_LG01* | 19.335 |
| Ca_Desi_SNP70 | [T/C] | *Ca_Desi_LG01* | 19.570 |
| Ca_Desi_SNP71 | [C/T] | *Ca_Desi_LG01* | 19.738 |
| Ca_Desi_SNP72 | [G/A] | *Ca_Desi_LG01* | 19.751 |
| Ca_Desi_SNP73 | [A/G] | *Ca_Desi_LG01* | 20.095 |
| Ca_Desi_SNP74 | [A/G] | *Ca_Desi_LG01* | 20.246 |
| Ca_Desi_SNP75 | [A/C] | *Ca_Desi_LG01* | 20.251 |
| Ca_Desi_SNP76 | [A/G] | *Ca_Desi_LG01* | 20.446 |
| Ca_Desi_SNP77 | [G/C] | *Ca_Desi_LG01* | 20.499 |
| Ca_Desi_SNP78 | [A/G] | *Ca_Desi_LG01* | 20.697 |
| Ca_Desi_SNP79 | [C/A] | *Ca_Desi_LG01* | 20.789 |
| Ca_Desi_SNP80 | [A/G] | *Ca_Desi_LG01* | 20.876 |
| Ca_Desi_SNP81 | [C/T] | *Ca_Desi_LG01* | 20.950 |
| Ca_Desi_SNP82 | [T/C] | *Ca_Desi_LG01* | 21.033 |
| Ca_Desi_SNP83 | [A/G] | *Ca_Desi_LG01* | 21.062 |
| Ca_Desi_SNP84 | [T/C] | *Ca_Desi_LG01* | 21.063 |
| Ca_Desi_SNP85 | [T/C] | *Ca_Desi_LG01* | 21.137 |
| Ca_Desi_SNP86 | [T/G] | *Ca_Desi_LG01* | 21.157 |
| Ca_Desi_SNP87 | [T/G] | *Ca_Desi_LG01* | 21.184 |
| Ca_Desi_SNP88 | [C/A] | *Ca_Desi_LG01* | 21.275 |
| Ca_Desi_SNP89 | [T/C] | *Ca_Desi_LG01* | 21.376 |
| Ca_Desi_SNP90 | [A/C] | *Ca_Desi_LG01* | 21.388 |
| Ca_Desi_SNP91 | [G/C] | *Ca_Desi_LG01* | 21.590 |
| Ca_Desi_SNP92 | [A/G] | *Ca_Desi_LG01* | 21.631 |
| Ca_Desi_SNP93 | [T/G] | *Ca_Desi_LG01* | 21.779 |

| **SNP IDs** | **SNPs** | **Linkage Group (LGs)** | **Genetic positions (cM) of mapped SNPs** |
|---|---|---|---|
| Ca_Desi_SNP94 | [G/T] | *Ca_Desi_LG01* | 22.083 |
| Ca_Desi_SNP95 | [T/C] | *Ca_Desi_LG01* | 22.101 |
| Ca_Desi_SNP96 | [G/T] | *Ca_Desi_LG01* | 22.270 |
| Ca_Desi_SNP97 | [A/C] | *Ca_Desi_LG01* | 22.343 |
| Ca_Desi_SNP98 | [T/G] | *Ca_Desi_LG01* | 22.501 |
| Ca_Desi_SNP99 | [C/T] | *Ca_Desi_LG01* | 22.503 |
| Ca_Desi_SNP100 | [G/T] | *Ca_Desi_LG01* | 22.516 |
| Ca_Desi_SNP101 | [G/T] | *Ca_Desi_LG01* | 22.550 |
| Ca_Desi_SNP102 | [A/G] | *Ca_Desi_LG01* | 22.554 |
| Ca_Desi_SNP103 | [A/C] | *Ca_Desi_LG01* | 22.645 |
| Ca_Desi_SNP104 | [T/C] | *Ca_Desi_LG01* | 22.695 |
| Ca_Desi_SNP105 | [G/C] | *Ca_Desi_LG01* | 22.775 |
| Ca_Desi_SNP106 | [C/T] | *Ca_Desi_LG01* | 22.854 |
| Ca_Desi_SNP107 | [A/G] | *Ca_Desi_LG01* | 22.861 |
| Ca_Desi_SNP108 | [A/G] | *Ca_Desi_LG01* | 22.911 |
| Ca_Desi_SNP109 | [C/G] | *Ca_Desi_LG01* | 22.938 |
| Ca_Desi_SNP110 | [A/C] | *Ca_Desi_LG01* | 23.190 |
| Ca_Desi_SNP111 | [A/C] | *Ca_Desi_LG01* | 23.224 |
| Ca_Desi_SNP112 | [A/C] | *Ca_Desi_LG01* | 23.297 |
| Ca_Desi_SNP113 | [T/C] | *Ca_Desi_LG01* | 23.469 |
| Ca_Desi_SNP114 | [G/A] | *Ca_Desi_LG01* | 23.498 |
| Ca_Desi_SNP115 | [G/A] | *Ca_Desi_LG01* | 23.577 |
| Ca_Desi_SNP116 | [A/C] | *Ca_Desi_LG01* | 23.580 |
| Ca_Desi_SNP117 | [C/T] | *Ca_Desi_LG01* | 23.647 |
| Ca_Desi_SNP118 | [A/G] | *Ca_Desi_LG01* | 23.756 |
| Ca_Desi_SNP119 | [G/A] | *Ca_Desi_LG01* | 23.826 |
| Ca_Desi_SNP120 | [T/C] | *Ca_Desi_LG01* | 23.840 |
| Ca_Desi_SNP121 | [G/T] | *Ca_Desi_LG01* | 23.886 |
| Ca_Desi_SNP122 | [T/C] | *Ca_Desi_LG01* | 24.023 |
| Ca_Desi_SNP123 | [C/T] | *Ca_Desi_LG01* | 24.050 |
| Ca_Desi_SNP124 | [T/G] | *Ca_Desi_LG01* | 24.127 |
| Ca_Desi_SNP125 | [T/C] | *Ca_Desi_LG01* | 24.187 |
| Ca_Desi_SNP126 | [A/G] | *Ca_Desi_LG01* | 24.256 |
| Ca_Desi_SNP127 | [A/C] | *Ca_Desi_LG01* | 24.391 |
| Ca_Desi_SNP128 | [C/T] | *Ca_Desi_LG01* | 24.501 |
| Ca_Desi_SNP129 | [C/A] | *Ca_Desi_LG01* | 24.568 |
| Ca_Desi_SNP130 | [A/G] | *Ca_Desi_LG01* | 24.774 |
| Ca_Desi_SNP131 | [C/T] | *Ca_Desi_LG01* | 24.813 |
| Ca_Desi_SNP132 | [T/C] | *Ca_Desi_LG01* | 24.909 |
| Ca_Desi_SNP133 | [A/G] | *Ca_Desi_LG01* | 24.977 |
| Ca_Desi_SNP134 | [C/T] | *Ca_Desi_LG01* | 25.031 |
| Ca_Desi_SNP135 | [T/C] | *Ca_Desi_LG01* | 25.220 |
| Ca_Desi_SNP136 | [A/C] | *Ca_Desi_LG01* | 25.243 |
| Ca_Desi_SNP137 | [G/T] | *Ca_Desi_LG01* | 25.380 |
| Ca_Desi_SNP138 | [A/G] | *Ca_Desi_LG01* | 25.394 |
| Ca_Desi_SNP139 | [G/A] | *Ca_Desi_LG01* | 25.489 |
| Ca_Desi_SNP140 | [T/C] | *Ca_Desi_LG01* | 25.750 |
| Ca_Desi_SNP141 | [T/C] | *Ca_Desi_LG01* | 25.792 |

| **SNP IDs** | **SNPs** | **Linkage Group (LGs)** | **Genetic positions (cM) of mapped SNPs** |
|---|---|---|---|
| Ca_Desi_SNP142 | [T/C] | *Ca_Desi_LG01* | 25.834 |
| Ca_Desi_SNP143 | [T/C] | *Ca_Desi_LG01* | 25.904 |
| Ca_Desi_SNP144 | [T/A] | *Ca_Desi_LG01* | 25.933 |
| Ca_Desi_SNP145 | [A/G] | *Ca_Desi_LG01* | 25.970 |
| Ca_Desi_SNP146 | [C/T] | *Ca_Desi_LG01* | 26.034 |
| Ca_Desi_SNP147 | [T/G] | *Ca_Desi_LG01* | 26.129 |
| Ca_Desi_SNP148 | [C/A] | *Ca_Desi_LG01* | 26.146 |
| Ca_Desi_SNP149 | [T/G] | *Ca_Desi_LG01* | 26.198 |
| Ca_Desi_SNP150 | [T/G] | *Ca_Desi_LG01* | 26.418 |
| Ca_Desi_SNP151 | [T/C] | *Ca_Desi_LG01* | 26.510 |
| Ca_Desi_SNP152 | [A/G] | *Ca_Desi_LG01* | 26.521 |
| Ca_Desi_SNP153 | [A/G] | *Ca_Desi_LG01* | 26.553 |
| Ca_Desi_SNP154 | [C/G] | *Ca_Desi_LG01* | 26.678 |
| Ca_Desi_SNP155 | [A/C] | *Ca_Desi_LG01* | 26.686 |
| Ca_Desi_SNP156 | [G/T] | *Ca_Desi_LG01* | 26.745 |
| Ca_Desi_SNP157 | [G/C] | *Ca_Desi_LG01* | 26.903 |
| Ca_Desi_SNP158 | [G/A] | *Ca_Desi_LG01* | 26.972 |
| Ca_Desi_SNP159 | [A/G] | *Ca_Desi_LG01* | 27.048 |
| Ca_Desi_SNP160 | [C/T] | *Ca_Desi_LG01* | 27.105 |
| Ca_Desi_SNP161 | [G/T] | *Ca_Desi_LG01* | 27.239 |
| Ca_Desi_SNP162 | [T/C] | *Ca_Desi_LG01* | 27.283 |
| Ca_Desi_SNP163 | [T/C] | *Ca_Desi_LG01* | 27.329 |
| Ca_Desi_SNP164 | [A/C] | *Ca_Desi_LG01* | 27.545 |
| Ca_Desi_SNP165 | [T/C] | *Ca_Desi_LG01* | 27.580 |
| Ca_Desi_SNP166 | [T/G] | *Ca_Desi_LG01* | 27.676 |
| Ca_Desi_SNP167 | [A/G] | *Ca_Desi_LG01* | 27.789 |
| Ca_Desi_SNP168 | [A/C] | *Ca_Desi_LG01* | 27.892 |
| Ca_Desi_SNP169 | [G/A] | *Ca_Desi_LG01* | 28.100 |
| Ca_Desi_SNP170 | [C/A] | *Ca_Desi_LG01* | 28.135 |
| Ca_Desi_SNP171 | [T/C] | *Ca_Desi_LG01* | 28.158 |
| Ca_Desi_SNP172 | [A/G] | *Ca_Desi_LG01* | 28.166 |
| Ca_Desi_SNP173 | [A/C] | *Ca_Desi_LG01* | 28.195 |
| Ca_Desi_SNP174 | [A/G] | *Ca_Desi_LG01* | 28.307 |
| Ca_Desi_SNP175 | [T/C] | *Ca_Desi_LG01* | 28.428 |
| Ca_Desi_SNP176 | [G/C] | *Ca_Desi_LG01* | 28.485 |
| Ca_Desi_SNP177 | [T/C] | *Ca_Desi_LG01* | 28.644 |
| Ca_Desi_SNP178 | [A/T] | *Ca_Desi_LG01* | 28.784 |
| Ca_Desi_SNP179 | [A/G] | *Ca_Desi_LG01* | 28.870 |
| Ca_Desi_SNP180 | [G/T] | *Ca_Desi_LG01* | 29.009 |
| Ca_Desi_SNP181 | [T/G] | *Ca_Desi_LG01* | 29.069 |
| Ca_Desi_SNP182 | [C/T] | *Ca_Desi_LG01* | 29.166 |
| Ca_Desi_SNP183 | [T/C] | *Ca_Desi_LG01* | 29.285 |
| Ca_Desi_SNP184 | [A/G] | *Ca_Desi_LG01* | 29.306 |
| Ca_Desi_SNP185 | [A/G] | *Ca_Desi_LG01* | 29.321 |
| Ca_Desi_SNP186 | [T/C] | *Ca_Desi_LG01* | 29.326 |
| Ca_Desi_SNP187 | [G/C] | *Ca_Desi_LG01* | 29.349 |
| Ca_Desi_SNP188 | [G/A] | *Ca_Desi_LG01* | 29.359 |
| Ca_Desi_SNP189 | [T/G] | *Ca_Desi_LG01* | 29.372 |

| **SNP IDs** | **SNPs** | **Linkage Group (LGs)** | **Genetic positions (cM) of mapped SNPs** |
|---|---|---|---|
| Ca_Desi_SNP190 | [A/T] | *Ca_Desi_LG01* | 29.427 |
| Ca_Desi_SNP191 | [G/A] | *Ca_Desi_LG01* | 29.612 |
| Ca_Desi_SNP192 | [A/C] | *Ca_Desi_LG01* | 29.709 |
| Ca_Desi_SNP193 | [A/G] | *Ca_Desi_LG01* | 29.772 |
| Ca_Desi_SNP194 | [A/C] | *Ca_Desi_LG01* | 29.881 |
| Ca_Desi_SNP195 | [T/A] | *Ca_Desi_LG01* | 29.923 |
| Ca_Desi_SNP196 | [A/C] | *Ca_Desi_LG01* | 29.977 |
| Ca_Desi_SNP197 | [T/G] | *Ca_Desi_LG01* | 29.978 |
| Ca_Desi_SNP198 | [A/C] | *Ca_Desi_LG01* | 30.017 |
| Ca_Desi_SNP199 | [T/G] | *Ca_Desi_LG01* | 30.020 |
| Ca_Desi_SNP200 | [T/C] | *Ca_Desi_LG01* | 30.034 |
| Ca_Desi_SNP201 | [A/C] | *Ca_Desi_LG01* | 30.164 |
| Ca_Desi_SNP202 | [C/G] | *Ca_Desi_LG01* | 30.186 |
| Ca_Desi_SNP203 | [A/G] | *Ca_Desi_LG01* | 30.456 |
| Ca_Desi_SNP204 | [T/G] | *Ca_Desi_LG01* | 30.463 |
| Ca_Desi_SNP205 | [T/G] | *Ca_Desi_LG01* | 30.466 |
| Ca_Desi_SNP206 | [A/G] | *Ca_Desi_LG01* | 30.555 |
| Ca_Desi_SNP207 | [C/T] | *Ca_Desi_LG01* | 30.735 |
| Ca_Desi_SNP208 | [T/G] | *Ca_Desi_LG01* | 30.777 |
| Ca_Desi_SNP209 | [T/G] | *Ca_Desi_LG01* | 30.918 |
| Ca_Desi_SNP210 | [C/T] | *Ca_Desi_LG01* | 30.946 |
| Ca_Desi_SNP211 | [C/T] | *Ca_Desi_LG01* | 31.129 |
| Ca_Desi_SNP212 | [C/A] | *Ca_Desi_LG01* | 31.156 |
| Ca_Desi_SNP213 | [A/T] | *Ca_Desi_LG01* | 31.237 |
| Ca_Desi_SNP214 | [T/G] | *Ca_Desi_LG01* | 31.367 |
| Ca_Desi_SNP215 | [G/C] | *Ca_Desi_LG01* | 31.532 |
| Ca_Desi_SNP216 | [T/G] | *Ca_Desi_LG01* | 31.546 |
| Ca_Desi_SNP217 | [A/G] | *Ca_Desi_LG01* | 31.566 |
| Ca_Desi_SNP218 | [C/T] | *Ca_Desi_LG01* | 31.666 |
| Ca_Desi_SNP219 | [A/T] | *Ca_Desi_LG01* | 31.702 |
| Ca_Desi_SNP220 | [C/G] | *Ca_Desi_LG01* | 31.825 |
| Ca_Desi_SNP221 | [T/C] | *Ca_Desi_LG01* | 31.851 |
| Ca_Desi_SNP222 | [T/C] | *Ca_Desi_LG01* | 31.870 |
| Ca_Desi_SNP223 | [A/G] | *Ca_Desi_LG01* | 31.957 |
| Ca_Desi_SNP224 | [T/C] | *Ca_Desi_LG01* | 32.027 |
| Ca_Desi_SNP225 | [T/A] | *Ca_Desi_LG01* | 32.033 |
| Ca_Desi_SNP226 | [C/T] | *Ca_Desi_LG01* | 32.056 |
| Ca_Desi_SNP227 | [A/G] | *Ca_Desi_LG01* | 32.080 |
| Ca_Desi_SNP228 | [A/C] | *Ca_Desi_LG01* | 32.190 |
| Ca_Desi_SNP229 | [T/C] | *Ca_Desi_LG01* | 32.328 |
| Ca_Desi_SNP230 | [C/T] | *Ca_Desi_LG01* | 32.362 |
| Ca_Desi_SNP231 | [G/T] | *Ca_Desi_LG01* | 32.398 |
| Ca_Desi_SNP232 | [A/C] | *Ca_Desi_LG01* | 32.448 |
| Ca_Desi_SNP233 | [A/G] | *Ca_Desi_LG01* | 32.460 |
| Ca_Desi_SNP234 | [T/A] | *Ca_Desi_LG01* | 32.495 |
| Ca_Desi_SNP235 | [G/A] | *Ca_Desi_LG01* | 32.501 |
| Ca_Desi_SNP236 | [A/C] | *Ca_Desi_LG01* | 32.707 |
| Ca_Desi_SNP237 | [G/T] | *Ca_Desi_LG01* | 32.956 |

| **SNP IDs** | **SNPs** | **Linkage Group (LGs)** | **Genetic positions (cM) of mapped SNPs** |
|---|---|---|---|
| Ca_Desi_SNP238 | [A/G] | *Ca_Desi_LG01* | 32.989 |
| Ca_Desi_SNP239 | [T/A] | *Ca_Desi_LG01* | 33.102 |
| Ca_Desi_SNP240 | [T/C] | *Ca_Desi_LG01* | 33.139 |
| Ca_Desi_SNP241 | [C/G] | *Ca_Desi_LG01* | 33.241 |
| Ca_Desi_SNP242 | [G/A] | *Ca_Desi_LG01* | 33.301 |
| Ca_Desi_SNP243 | [A/T] | *Ca_Desi_LG01* | 33.324 |
| Ca_Desi_SNP244 | [G/A] | *Ca_Desi_LG01* | 33.376 |
| Ca_Desi_SNP245 | [G/A] | *Ca_Desi_LG01* | 33.427 |
| Ca_Desi_SNP246 | [G/A] | *Ca_Desi_LG01* | 33.642 |
| Ca_Desi_SNP247 | [T/C] | *Ca_Desi_LG01* | 33.751 |
| Ca_Desi_SNP248 | [A/C] | *Ca_Desi_LG01* | 34.019 |
| Ca_Desi_SNP249 | [A/G] | *Ca_Desi_LG01* | 34.247 |
| Ca_Desi_SNP250 | [C/G] | *Ca_Desi_LG01* | 34.293 |
| Ca_Desi_SNP251 | [C/A] | *Ca_Desi_LG01* | 34.372 |
| Ca_Desi_SNP252 | [C/T] | *Ca_Desi_LG01* | 34.453 |
| Ca_Desi_SNP253 | [A/G] | *Ca_Desi_LG01* | 34.466 |
| Ca_Desi_SNP254 | [C/A] | *Ca_Desi_LG01* | 34.520 |
| Ca_Desi_SNP255 | [A/C] | *Ca_Desi_LG01* | 34.683 |
| Ca_Desi_SNP256 | [T/C] | *Ca_Desi_LG01* | 34.918 |
| Ca_Desi_SNP257 | [C/T] | *Ca_Desi_LG01* | 35.214 |
| Ca_Desi_SNP258 | [C/T] | *Ca_Desi_LG01* | 35.417 |
| Ca_Desi_SNP259 | [A/C] | *Ca_Desi_LG01* | 35.478 |
| Ca_Desi_SNP260 | [T/G] | *Ca_Desi_LG01* | 35.536 |
| Ca_Desi_SNP261 | [G/A] | *Ca_Desi_LG01* | 35.554 |
| Ca_Desi_SNP262 | [T/G] | *Ca_Desi_LG01* | 35.782 |
| Ca_Desi_SNP263 | [A/G] | *Ca_Desi_LG01* | 36.163 |
| Ca_Desi_SNP264 | [A/G] | *Ca_Desi_LG01* | 36.358 |
| Ca_Desi_SNP265 | [T/C] | *Ca_Desi_LG01* | 36.514 |
| Ca_Desi_SNP266 | [C/T] | *Ca_Desi_LG01* | 36.525 |
| Ca_Desi_SNP267 | [C/T] | *Ca_Desi_LG01* | 36.560 |
| Ca_Desi_SNP268 | [A/C] | *Ca_Desi_LG01* | 36.582 |
| Ca_Desi_SNP269 | [G/A] | *Ca_Desi_LG01* | 36.653 |
| Ca_Desi_SNP270 | [G/A] | *Ca_Desi_LG01* | 36.698 |
| Ca_Desi_SNP271 | [T/G] | *Ca_Desi_LG01* | 36.743 |
| Ca_Desi_SNP272 | [C/G] | *Ca_Desi_LG01* | 36.750 |
| Ca_Desi_SNP273 | [A/G] | *Ca_Desi_LG01* | 36.796 |
| Ca_Desi_SNP274 | [A/C] | *Ca_Desi_LG01* | 36.836 |
| Ca_Desi_SNP275 | [G/A] | *Ca_Desi_LG01* | 36.879 |
| Ca_Desi_SNP276 | [A/C] | *Ca_Desi_LG01* | 36.964 |
| Ca_Desi_SNP277 | [T/C] | *Ca_Desi_LG01* | 37.126 |
| Ca_Desi_SNP278 | [T/C] | *Ca_Desi_LG01* | 37.132 |
| Ca_Desi_SNP279 | [T/C] | *Ca_Desi_LG01* | 37.136 |
| Ca_Desi_SNP280 | [A/G] | *Ca_Desi_LG01* | 37.646 |
| Ca_Desi_SNP281 | [C/A] | *Ca_Desi_LG01* | 37.674 |
| Ca_Desi_SNP282 | [G/C] | *Ca_Desi_LG01* | 37.748 |
| Ca_Desi_SNP283 | [A/G] | *Ca_Desi_LG01* | 37.762 |
| Ca_Desi_SNP284 | [C/T] | *Ca_Desi_LG01* | 37.964 |
| Ca_Desi_SNP285 | [T/A] | *Ca_Desi_LG01* | 38.033 |

| **SNP IDs** | **SNPs** | **Linkage Group (LGs)** | **Genetic positions (cM) of mapped SNPs** |
|---|---|---|---|
| Ca_Desi_SNP286 | [T/A] | *Ca_Desi_LG01* | 38.049 |
| Ca_Desi_SNP287 | [T/C] | *Ca_Desi_LG01* | 38.051 |
| Ca_Desi_SNP288 | [C/T] | *Ca_Desi_LG01* | 38.142 |
| Ca_Desi_SNP289 | [C/T] | *Ca_Desi_LG01* | 38.376 |
| Ca_Desi_SNP290 | [A/T] | *Ca_Desi_LG01* | 38.702 |
| Ca_Desi_SNP291 | [G/C] | *Ca_Desi_LG01* | 38.731 |
| Ca_Desi_SNP292 | [A/C] | *Ca_Desi_LG01* | 38.810 |
| Ca_Desi_SNP293 | [C/T] | *Ca_Desi_LG01* | 38.827 |
| Ca_Desi_SNP294 | [C/G] | *Ca_Desi_LG01* | 39.080 |
| Ca_Desi_SNP295 | [C/T] | *Ca_Desi_LG01* | 39.127 |
| Ca_Desi_SNP296 | [A/G] | *Ca_Desi_LG01* | 39.166 |
| Ca_Desi_SNP297 | [A/G] | *Ca_Desi_LG01* | 39.397 |
| Ca_Desi_SNP298 | [G/T] | *Ca_Desi_LG01* | 39.425 |
| Ca_Desi_SNP299 | [A/C] | *Ca_Desi_LG01* | 39.439 |
| Ca_Desi_SNP300 | [G/A] | *Ca_Desi_LG01* | 39.484 |
| Ca_Desi_SNP301 | [A/C] | *Ca_Desi_LG01* | 39.506 |
| Ca_Desi_SNP302 | [A/G] | *Ca_Desi_LG01* | 39.508 |
| Ca_Desi_SNP303 | [A/C] | *Ca_Desi_LG01* | 39.538 |
| Ca_Desi_SNP304 | [T/G] | *Ca_Desi_LG01* | 39.610 |
| Ca_Desi_SNP305 | [C/T] | *Ca_Desi_LG01* | 39.719 |
| Ca_Desi_SNP306 | [G/A] | *Ca_Desi_LG01* | 39.793 |
| Ca_Desi_SNP307 | [A/G] | *Ca_Desi_LG01* | 39.842 |
| Ca_Desi_SNP308 | [G/C] | *Ca_Desi_LG01* | 39.893 |
| Ca_Desi_SNP309 | [G/T] | *Ca_Desi_LG01* | 39.980 |
| Ca_Desi_SNP310 | [T/C] | *Ca_Desi_LG01* | 40.186 |
| Ca_Desi_SNP311 | [A/G] | *Ca_Desi_LG01* | 40.216 |
| Ca_Desi_SNP312 | [T/C] | *Ca_Desi_LG01* | 40.347 |
| Ca_Desi_SNP313 | [C/T] | *Ca_Desi_LG01* | 40.403 |
| Ca_Desi_SNP314 | [A/G] | *Ca_Desi_LG01* | 40.431 |
| Ca_Desi_SNP315 | [T/C] | *Ca_Desi_LG01* | 40.558 |
| Ca_Desi_SNP316 | [A/G] | *Ca_Desi_LG01* | 40.604 |
| Ca_Desi_SNP317 | [T/C] | *Ca_Desi_LG01* | 41.006 |
| Ca_Desi_SNP318 | [T/C] | *Ca_Desi_LG01* | 41.013 |
| Ca_Desi_SNP319 | [C/A] | *Ca_Desi_LG01* | 41.032 |
| Ca_Desi_SNP320 | [T/C] | *Ca_Desi_LG01* | 41.144 |
| Ca_Desi_SNP321 | [T/C] | *Ca_Desi_LG01* | 41.149 |
| Ca_Desi_SNP322 | [T/G] | *Ca_Desi_LG01* | 41.224 |
| Ca_Desi_SNP323 | [T/G] | *Ca_Desi_LG01* | 41.255 |
| Ca_Desi_SNP324 | [G/A] | *Ca_Desi_LG01* | 41.310 |
| Ca_Desi_SNP325 | [A/G] | *Ca_Desi_LG01* | 41.414 |
| Ca_Desi_SNP326 | [T/C] | *Ca_Desi_LG01* | 41.489 |
| Ca_Desi_SNP327 | [G/A] | *Ca_Desi_LG01* | 41.490 |
| Ca_Desi_SNP328 | [A/G] | *Ca_Desi_LG01* | 41.561 |
| Ca_Desi_SNP329 | [G/A] | *Ca_Desi_LG01* | 41.598 |
| Ca_Desi_SNP330 | [C/T] | *Ca_Desi_LG01* | 41.721 |
| Ca_Desi_SNP331 | [T/C] | *Ca_Desi_LG01* | 41.819 |
| Ca_Desi_SNP332 | [T/C] | *Ca_Desi_LG01* | 41.909 |
| Ca_Desi_SNP333 | [T/C] | *Ca_Desi_LG01* | 42.172 |

| **SNP IDs** | **SNPs** | **Linkage Group (LGs)** | **Genetic positions (cM) of mapped SNPs** |
|---|---|---|---|
| Ca_Desi_SNP334 | [A/T] | *Ca_Desi_LG01* | 42.374 |
| Ca_Desi_SNP335 | [G/A] | *Ca_Desi_LG01* | 42.400 |
| Ca_Desi_SNP336 | [T/C] | *Ca_Desi_LG01* | 42.430 |
| Ca_Desi_SNP337 | [C/T] | *Ca_Desi_LG01* | 42.664 |
| Ca_Desi_SNP338 | [A/C] | *Ca_Desi_LG01* | 42.667 |
| Ca_Desi_SNP339 | [C/T] | *Ca_Desi_LG01* | 42.830 |
| Ca_Desi_SNP340 | [C/T] | *Ca_Desi_LG01* | 42.927 |
| Ca_Desi_SNP341 | [G/T] | *Ca_Desi_LG01* | 42.947 |
| Ca_Desi_SNP342 | [A/G] | *Ca_Desi_LG01* | 42.989 |
| Ca_Desi_SNP343 | [T/G] | *Ca_Desi_LG01* | 43.057 |
| Ca_Desi_SNP344 | [T/C] | *Ca_Desi_LG01* | 43.146 |
| Ca_Desi_SNP345 | [A/G] | *Ca_Desi_LG01* | 43.261 |
| Ca_Desi_SNP346 | [A/G] | *Ca_Desi_LG01* | 43.282 |
| Ca_Desi_SNP347 | [C/A] | *Ca_Desi_LG01* | 43.445 |
| Ca_Desi_SNP348 | [A/G] | *Ca_Desi_LG01* | 43.467 |
| Ca_Desi_SNP349 | [A/T] | *Ca_Desi_LG01* | 43.745 |
| Ca_Desi_SNP350 | [G/T] | *Ca_Desi_LG01* | 43.968 |
| Ca_Desi_SNP351 | [T/G] | *Ca_Desi_LG01* | 44.063 |
| Ca_Desi_SNP352 | [A/G] | *Ca_Desi_LG01* | 44.121 |
| Ca_Desi_SNP353 | [T/C] | *Ca_Desi_LG01* | 44.159 |
| Ca_Desi_SNP354 | [C/T] | *Ca_Desi_LG01* | 44.308 |
| Ca_Desi_SNP355 | [T/C] | *Ca_Desi_LG01* | 44.325 |
| Ca_Desi_SNP356 | [A/C] | *Ca_Desi_LG01* | 44.327 |
| Ca_Desi_SNP357 | [A/G] | *Ca_Desi_LG01* | 44.362 |
| Ca_Desi_SNP358 | [A/C] | *Ca_Desi_LG01* | 44.480 |
| Ca_Desi_SNP359 | [A/G] | *Ca_Desi_LG01* | 44.588 |
| Ca_Desi_SNP360 | [C/T] | *Ca_Desi_LG01* | 44.844 |
| Ca_Desi_SNP361 | [A/G] | *Ca_Desi_LG01* | 44.856 |
| Ca_Desi_SNP362 | [G/A] | *Ca_Desi_LG01* | 44.873 |
| Ca_Desi_SNP363 | [T/C] | *Ca_Desi_LG01* | 45.043 |
| Ca_Desi_SNP364 | [C/A] | *Ca_Desi_LG01* | 45.048 |
| Ca_Desi_SNP365 | [T/A] | *Ca_Desi_LG01* | 45.079 |
| Ca_Desi_SNP366 | [C/T] | *Ca_Desi_LG01* | 45.210 |
| Ca_Desi_SNP367 | [A/G] | *Ca_Desi_LG01* | 45.225 |
| Ca_Desi_SNP368 | [G/A] | *Ca_Desi_LG01* | 45.447 |
| Ca_Desi_SNP369 | [G/C] | *Ca_Desi_LG01* | 45.488 |
| Ca_Desi_SNP370 | [A/G] | *Ca_Desi_LG01* | 45.539 |
| Ca_Desi_SNP371 | [A/C] | *Ca_Desi_LG01* | 45.656 |
| Ca_Desi_SNP372 | [C/T] | *Ca_Desi_LG01* | 45.723 |
| Ca_Desi_SNP373 | [C/T] | *Ca_Desi_LG01* | 45.726 |
| Ca_Desi_SNP374 | [A/C] | *Ca_Desi_LG01* | 45.748 |
| Ca_Desi_SNP375 | [T/G] | *Ca_Desi_LG01* | 45.765 |
| Ca_Desi_SNP376 | [T/C] | *Ca_Desi_LG01* | 45.800 |
| Ca_Desi_SNP377 | [C/T] | *Ca_Desi_LG01* | 45.974 |
| Ca_Desi_SNP378 | [G/A] | *Ca_Desi_LG01* | 46.133 |
| Ca_Desi_SNP379 | [T/G] | *Ca_Desi_LG01* | 46.235 |
| Ca_Desi_SNP380 | [G/A] | *Ca_Desi_LG01* | 46.295 |
| Ca_Desi_SNP381 | [A/G] | *Ca_Desi_LG01* | 46.497 |

| **SNP IDs** | **SNPs** | **Linkage Group (LGs)** | **Genetic positions (cM) of mapped SNPs** |
|---|---|---|---|
| Ca_Desi_SNP382 | [A/G] | *Ca_Desi_LG01* | 46.768 |
| Ca_Desi_SNP383 | [T/G] | *Ca_Desi_LG01* | 46.770 |
| Ca_Desi_SNP384 | [A/C] | *Ca_Desi_LG01* | 46.791 |
| Ca_Desi_SNP385 | [C/T] | *Ca_Desi_LG01* | 46.870 |
| Ca_Desi_SNP386 | [T/C] | *Ca_Desi_LG01* | 46.942 |
| Ca_Desi_SNP387 | [T/C] | *Ca_Desi_LG01* | 47.032 |
| Ca_Desi_SNP388 | [T/C] | *Ca_Desi_LG01* | 47.299 |
| Ca_Desi_SNP389 | [A/C] | *Ca_Desi_LG01* | 47.453 |
| Ca_Desi_SNP390 | [T/C] | *Ca_Desi_LG01* | 47.458 |
| Ca_Desi_SNP391 | [A/G] | *Ca_Desi_LG01* | 47.484 |
| Ca_Desi_SNP392 | [C/A] | *Ca_Desi_LG01* | 47.566 |
| Ca_Desi_SNP393 | [A/C] | *Ca_Desi_LG01* | 47.658 |
| Ca_Desi_SNP394 | [T/G] | *Ca_Desi_LG01* | 47.793 |
| Ca_Desi_SNP395 | [A/G] | *Ca_Desi_LG01* | 48.130 |
| Ca_Desi_SNP396 | [C/T] | *Ca_Desi_LG01* | 48.136 |
| Ca_Desi_SNP397 | [A/T] | *Ca_Desi_LG01* | 48.623 |
| Ca_Desi_SNP398 | [C/T] | *Ca_Desi_LG01* | 48.663 |
| Ca_Desi_SNP399 | [G/A] | *Ca_Desi_LG01* | 48.690 |
| Ca_Desi_SNP400 | [T/C] | *Ca_Desi_LG01* | 48.765 |
| Ca_Desi_SNP401 | [C/A] | *Ca_Desi_LG01* | 48.793 |
| Ca_Desi_SNP402 | [C/A] | *Ca_Desi_LG01* | 48.807 |
| Ca_Desi_SNP403 | [A/G] | *Ca_Desi_LG01* | 48.911 |
| Ca_Desi_SNP404 | [A/G] | *Ca_Desi_LG01* | 49.194 |
| Ca_Desi_SNP405 | [T/C] | *Ca_Desi_LG01* | 49.198 |
| Ca_Desi_SNP406 | [T/C] | *Ca_Desi_LG01* | 49.398 |
| Ca_Desi_SNP407 | [C/G] | *Ca_Desi_LG01* | 49.411 |
| Ca_Desi_SNP408 | [T/G] | *Ca_Desi_LG01* | 49.586 |
| Ca_Desi_SNP409 | [T/G] | *Ca_Desi_LG01* | 49.635 |
| Ca_Desi_SNP410 | [T/G] | *Ca_Desi_LG01* | 49.730 |
| Ca_Desi_SNP411 | [T/A] | *Ca_Desi_LG01* | 49.755 |
| Ca_Desi_SNP412 | [G/C] | *Ca_Desi_LG01* | 50.066 |
| Ca_Desi_SNP413 | [A/G] | *Ca_Desi_LG01* | 50.267 |
| Ca_Desi_SNP414 | [C/A] | *Ca_Desi_LG01* | 50.537 |
| Ca_Desi_SNP415 | [T/C] | *Ca_Desi_LG01* | 50.556 |
| Ca_Desi_SNP416 | [T/C] | *Ca_Desi_LG01* | 50.565 |
| Ca_Desi_SNP417 | [T/C] | *Ca_Desi_LG01* | 50.617 |
| Ca_Desi_SNP418 | [A/G] | *Ca_Desi_LG01* | 50.671 |
| Ca_Desi_SNP419 | [A/C] | *Ca_Desi_LG01* | 50.686 |
| Ca_Desi_SNP420 | [T/C] | *Ca_Desi_LG01* | 50.698 |
| Ca_Desi_SNP421 | [C/A] | *Ca_Desi_LG01* | 50.717 |
| Ca_Desi_SNP422 | [T/C] | *Ca_Desi_LG01* | 50.746 |
| Ca_Desi_SNP423 | [A/T] | *Ca_Desi_LG01* | 50.789 |
| Ca_Desi_SNP424 | [A/G] | *Ca_Desi_LG01* | 50.850 |
| Ca_Desi_SNP425 | [T/G] | *Ca_Desi_LG01* | 50.949 |
| Ca_Desi_SNP426 | [C/T] | *Ca_Desi_LG01* | 50.983 |
| Ca_Desi_SNP427 | [C/T] | *Ca_Desi_LG01* | 51.121 |
| Ca_Desi_SNP428 | [C/G] | *Ca_Desi_LG01* | 51.170 |
| Ca_Desi_SNP429 | [T/A] | *Ca_Desi_LG01* | 51.199 |

| **SNP IDs** | **SNPs** | **Linkage Group (LGs)** | **Genetic positions (cM) of mapped SNPs** |
|---|---|---|---|
| Ca_Desi_SNP430 | [G/C] | *Ca_Desi_LG01* | 51.211 |
| Ca_Desi_SNP431 | [C/A] | *Ca_Desi_LG01* | 51.251 |
| Ca_Desi_SNP432 | [A/G] | *Ca_Desi_LG01* | 51.305 |
| Ca_Desi_SNP433 | [T/C] | *Ca_Desi_LG01* | 51.332 |
| Ca_Desi_SNP434 | [A/G] | *Ca_Desi_LG01* | 51.343 |
| Ca_Desi_SNP435 | [T/G] | *Ca_Desi_LG01* | 51.387 |
| Ca_Desi_SNP436 | [T/C] | *Ca_Desi_LG01* | 51.398 |
| Ca_Desi_SNP437 | [A/G] | *Ca_Desi_LG01* | 51.512 |
| Ca_Desi_SNP438 | [G/T] | *Ca_Desi_LG01* | 51.531 |
| Ca_Desi_SNP439 | [A/G] | *Ca_Desi_LG01* | 51.552 |
| Ca_Desi_SNP440 | [A/C] | *Ca_Desi_LG01* | 51.653 |
| Ca_Desi_SNP441 | [T/A] | *Ca_Desi_LG01* | 51.899 |
| Ca_Desi_SNP442 | [T/G] | *Ca_Desi_LG01* | 51.928 |
| Ca_Desi_SNP443 | [A/G] | *Ca_Desi_LG01* | 51.995 |
| Ca_Desi_SNP444 | [T/A] | *Ca_Desi_LG01* | 52.008 |
| Ca_Desi_SNP445 | [A/G] | *Ca_Desi_LG01* | 52.196 |
| Ca_Desi_SNP446 | [A/T] | *Ca_Desi_LG01* | 52.210 |
| Ca_Desi_SNP447 | [T/C] | *Ca_Desi_LG01* | 52.279 |
| Ca_Desi_SNP448 | [A/G] | *Ca_Desi_LG01* | 52.282 |
| Ca_Desi_SNP449 | [T/C] | *Ca_Desi_LG01* | 52.290 |
| Ca_Desi_SNP450 | [A/G] | *Ca_Desi_LG01* | 52.318 |
| Ca_Desi_SNP451 | [G/T] | *Ca_Desi_LG01* | 52.321 |
| Ca_Desi_SNP452 | [T/C] | *Ca_Desi_LG01* | 52.377 |
| Ca_Desi_SNP453 | [A/G] | *Ca_Desi_LG01* | 52.439 |
| Ca_Desi_SNP454 | [G/T] | *Ca_Desi_LG01* | 52.446 |
| Ca_Desi_SNP455 | [A/T] | *Ca_Desi_LG01* | 52.460 |
| Ca_Desi_SNP456 | [A/G] | *Ca_Desi_LG01* | 52.521 |
| Ca_Desi_SNP457 | [C/T] | *Ca_Desi_LG01* | 52.670 |
| Ca_Desi_SNP458 | [A/G] | *Ca_Desi_LG01* | 52.730 |
| Ca_Desi_SNP459 | [A/C] | *Ca_Desi_LG01* | 52.751 |
| Ca_Desi_SNP460 | [A/C] | *Ca_Desi_LG01* | 52.758 |
| Ca_Desi_SNP461 | [T/C] | *Ca_Desi_LG01* | 52.803 |
| Ca_Desi_SNP462 | [G/A] | *Ca_Desi_LG01* | 52.830 |
| Ca_Desi_SNP463 | [A/T] | *Ca_Desi_LG01* | 52.834 |
| Ca_Desi_SNP464 | [C/T] | *Ca_Desi_LG01* | 52.959 |
| Ca_Desi_SNP465 | [T/G] | *Ca_Desi_LG01* | 53.054 |
| Ca_Desi_SNP466 | [G/T] | *Ca_Desi_LG01* | 53.292 |
| Ca_Desi_SNP467 | [A/C] | *Ca_Desi_LG01* | 53.407 |
| Ca_Desi_SNP468 | [G/T] | *Ca_Desi_LG01* | 53.519 |
| Ca_Desi_SNP469 | [T/C] | *Ca_Desi_LG01* | 53.535 |
| Ca_Desi_SNP470 | [C/T] | *Ca_Desi_LG01* | 53.579 |
| Ca_Desi_SNP471 | [T/G] | *Ca_Desi_LG01* | 53.581 |
| Ca_Desi_SNP472 | [G/T] | *Ca_Desi_LG01* | 53.621 |
| Ca_Desi_SNP473 | [A/C] | *Ca_Desi_LG01* | 53.728 |
| Ca_Desi_SNP474 | [A/G] | *Ca_Desi_LG01* | 53.729 |
| Ca_Desi_SNP475 | [G/A] | *Ca_Desi_LG01* | 53.738 |
| Ca_Desi_SNP476 | [A/C] | *Ca_Desi_LG01* | 53.739 |
| Ca_Desi_SNP477 | [T/G] | *Ca_Desi_LG01* | 54.094 |

| **SNP IDs** | **SNPs** | **Linkage Group (LGs)** | **Genetic positions (cM) of mapped SNPs** |
|---|---|---|---|
| Ca_Desi_SNP478 | [T/C] | *Ca_Desi_LG01* | 54.128 |
| Ca_Desi_SNP479 | [T/C] | *Ca_Desi_LG01* | 54.141 |
| Ca_Desi_SNP480 | [C/G] | *Ca_Desi_LG01* | 54.145 |
| Ca_Desi_SNP481 | [T/C] | *Ca_Desi_LG01* | 54.172 |
| Ca_Desi_SNP482 | [G/T] | *Ca_Desi_LG01* | 54.344 |
| Ca_Desi_SNP483 | [T/C] | *Ca_Desi_LG01* | 54.383 |
| Ca_Desi_SNP484 | [C/T] | *Ca_Desi_LG01* | 54.441 |
| Ca_Desi_SNP485 | [A/G] | *Ca_Desi_LG01* | 54.508 |
| Ca_Desi_SNP486 | [A/C] | *Ca_Desi_LG01* | 54.514 |
| Ca_Desi_SNP487 | [T/C] | *Ca_Desi_LG01* | 54.536 |
| Ca_Desi_SNP488 | [T/C] | *Ca_Desi_LG01* | 54.597 |
| Ca_Desi_SNP489 | [G/A] | *Ca_Desi_LG01* | 54.713 |
| Ca_Desi_SNP490 | [A/G] | *Ca_Desi_LG01* | 54.869 |
| Ca_Desi_SNP491 | [T/C] | *Ca_Desi_LG01* | 54.883 |
| Ca_Desi_SNP492 | [G/C] | *Ca_Desi_LG01* | 55.000 |
| Ca_Desi_SNP493 | [T/A] | *Ca_Desi_LG01* | 55.012 |
| Ca_Desi_SNP494 | [A/G] | *Ca_Desi_LG01* | 55.109 |
| Ca_Desi_SNP495 | [A/C] | *Ca_Desi_LG01* | 55.115 |
| Ca_Desi_SNP496 | [A/G] | *Ca_Desi_LG01* | 55.118 |
| Ca_Desi_SNP497 | [G/A] | *Ca_Desi_LG01* | 55.160 |
| Ca_Desi_SNP498 | [T/C] | *Ca_Desi_LG01* | 55.161 |
| Ca_Desi_SNP499 | [A/T] | *Ca_Desi_LG01* | 55.224 |
| Ca_Desi_SNP500 | [A/G] | *Ca_Desi_LG01* | 55.245 |
| Ca_Desi_SNP501 | [A/C] | *Ca_Desi_LG01* | 55.295 |
| Ca_Desi_SNP502 | [C/T] | *Ca_Desi_LG01* | 55.318 |
| Ca_Desi_SNP503 | [T/C] | *Ca_Desi_LG01* | 55.349 |
| Ca_Desi_SNP504 | [A/G] | *Ca_Desi_LG01* | 55.433 |
| Ca_Desi_SNP505 | [A/G] | *Ca_Desi_LG01* | 55.442 |
| Ca_Desi_SNP506 | [A/G] | *Ca_Desi_LG01* | 55.456 |
| Ca_Desi_SNP507 | [T/C] | *Ca_Desi_LG01* | 55.475 |
| Ca_Desi_SNP508 | [T/C] | *Ca_Desi_LG01* | 55.538 |
| Ca_Desi_SNP509 | [T/A] | *Ca_Desi_LG01* | 55.724 |
| Ca_Desi_SNP510 | [C/A] | *Ca_Desi_LG01* | 55.732 |
| Ca_Desi_SNP511 | [T/C] | *Ca_Desi_LG01* | 55.757 |
| Ca_Desi_SNP512 | [T/C] | *Ca_Desi_LG01* | 56.022 |
| Ca_Desi_SNP513 | [C/T] | *Ca_Desi_LG01* | 56.043 |
| Ca_Desi_SNP514 | [A/G] | *Ca_Desi_LG01* | 56.130 |
| Ca_Desi_SNP515 | [A/G] | *Ca_Desi_LG01* | 56.158 |
| Ca_Desi_SNP516 | [A/C] | *Ca_Desi_LG01* | 56.301 |
| Ca_Desi_SNP517 | [A/C] | *Ca_Desi_LG01* | 56.350 |
| Ca_Desi_SNP518 | [A/T] | *Ca_Desi_LG01* | 56.514 |
| Ca_Desi_SNP519 | [T/C] | *Ca_Desi_LG01* | 56.572 |
| Ca_Desi_SNP520 | [T/G] | *Ca_Desi_LG01* | 56.606 |
| Ca_Desi_SNP521 | [A/G] | *Ca_Desi_LG01* | 56.661 |
| Ca_Desi_SNP522 | [A/T] | *Ca_Desi_LG01* | 56.690 |
| Ca_Desi_SNP523 | [T/C] | *Ca_Desi_LG01* | 56.760 |
| Ca_Desi_SNP524 | [A/T] | *Ca_Desi_LG01* | 56.786 |
| Ca_Desi_SNP525 | [A/G] | *Ca_Desi_LG01* | 56.984 |

| **SNP IDs** | **SNPs** | **Linkage Group (LGs)** | **Genetic positions (cM) of mapped SNPs** |
|---|---|---|---|
| Ca_Desi_SNP526 | [A/G] | *Ca_Desi_LG01* | 57.121 |
| Ca_Desi_SNP527 | [T/C] | *Ca_Desi_LG01* | 57.172 |
| Ca_Desi_SNP528 | [T/C] | *Ca_Desi_LG01* | 57.223 |
| Ca_Desi_SNP529 | [T/C] | *Ca_Desi_LG01* | 57.294 |
| Ca_Desi_SNP530 | [G/C] | *Ca_Desi_LG01* | 57.334 |
| Ca_Desi_SNP531 | [A/G] | *Ca_Desi_LG01* | 57.540 |
| Ca_Desi_SNP532 | [T/C] | *Ca_Desi_LG01* | 57.632 |
| Ca_Desi_SNP533 | [A/G] | *Ca_Desi_LG01* | 57.688 |
| Ca_Desi_SNP534 | [A/C] | *Ca_Desi_LG01* | 57.692 |
| Ca_Desi_SNP535 | [T/G] | *Ca_Desi_LG01* | 57.700 |
| Ca_Desi_SNP536 | [G/A] | *Ca_Desi_LG01* | 57.720 |
| Ca_Desi_SNP537 | [A/C] | *Ca_Desi_LG01* | 57.906 |
| Ca_Desi_SNP538 | [A/G] | *Ca_Desi_LG01* | 57.927 |
| Ca_Desi_SNP539 | [T/G] | *Ca_Desi_LG01* | 58.070 |
| Ca_Desi_SNP540 | [G/T] | *Ca_Desi_LG01* | 58.093 |
| Ca_Desi_SNP541 | [T/G] | *Ca_Desi_LG01* | 58.304 |
| Ca_Desi_SNP542 | [A/C] | *Ca_Desi_LG01* | 58.358 |
| Ca_Desi_SNP543 | [T/A] | *Ca_Desi_LG01* | 58.390 |
| Ca_Desi_SNP544 | [A/C] | *Ca_Desi_LG01* | 58.530 |
| Ca_Desi_SNP545 | [T/C] | *Ca_Desi_LG01* | 58.758 |
| Ca_Desi_SNP546 | [T/G] | *Ca_Desi_LG01* | 58.790 |
| Ca_Desi_SNP547 | [T/C] | *Ca_Desi_LG01* | 58.843 |
| Ca_Desi_SNP548 | [T/C] | *Ca_Desi_LG01* | 58.876 |
| Ca_Desi_SNP549 | [T/C] | *Ca_Desi_LG01* | 58.928 |
| Ca_Desi_SNP550 | [G/A] | *Ca_Desi_LG01* | 58.934 |
| Ca_Desi_SNP551 | [T/G] | *Ca_Desi_LG01* | 58.947 |
| Ca_Desi_SNP552 | [C/A] | *Ca_Desi_LG01* | 58.974 |
| Ca_Desi_SNP553 | [T/G] | *Ca_Desi_LG01* | 58.981 |
| Ca_Desi_SNP554 | [A/T] | *Ca_Desi_LG01* | 59.042 |
| Ca_Desi_SNP555 | [G/A] | *Ca_Desi_LG01* | 59.057 |
| Ca_Desi_SNP556 | [T/C] | *Ca_Desi_LG01* | 59.075 |
| Ca_Desi_SNP557 | [G/A] | *Ca_Desi_LG01* | 59.131 |
| Ca_Desi_SNP558 | [A/G] | *Ca_Desi_LG01* | 59.154 |
| Ca_Desi_SNP559 | [A/G] | *Ca_Desi_LG01* | 59.239 |
| Ca_Desi_SNP560 | [A/T] | *Ca_Desi_LG01* | 59.271 |
| Ca_Desi_SNP561 | [A/C] | *Ca_Desi_LG01* | 59.306 |
| Ca_Desi_SNP562 | [T/G] | *Ca_Desi_LG01* | 59.429 |
| Ca_Desi_SNP563 | [A/C] | *Ca_Desi_LG01* | 59.578 |
| Ca_Desi_SNP564 | [T/C] | *Ca_Desi_LG01* | 59.584 |
| Ca_Desi_SNP565 | [T/C] | *Ca_Desi_LG01* | 59.590 |
| Ca_Desi_SNP566 | [C/A] | *Ca_Desi_LG01* | 59.671 |
| Ca_Desi_SNP567 | [C/T] | *Ca_Desi_LG01* | 59.822 |
| Ca_Desi_SNP568 | [G/C] | *Ca_Desi_LG01* | 59.845 |
| Ca_Desi_SNP569 | [G/A] | *Ca_Desi_LG01* | 59.869 |
| Ca_Desi_SNP570 | [C/G] | *Ca_Desi_LG01* | 60.108 |
| Ca_Desi_SNP571 | [A/G] | *Ca_Desi_LG01* | 60.189 |
| Ca_Desi_SNP572 | [A/G] | *Ca_Desi_LG01* | 60.401 |
| Ca_Desi_SNP573 | [A/G] | *Ca_Desi_LG01* | 60.446 |

| **SNP IDs** | **SNPs** | **Linkage Group (LGs)** | **Genetic positions (cM) of mapped SNPs** |
|---|---|---|---|
| Ca_Desi_SNP574 | [A/G] | *Ca_Desi_LG01* | 60.495 |
| Ca_Desi_SNP575 | [A/G] | *Ca_Desi_LG01* | 60.548 |
| Ca_Desi_SNP576 | [C/T] | *Ca_Desi_LG01* | 60.616 |
| Ca_Desi_SNP577 | [T/C] | *Ca_Desi_LG01* | 60.619 |
| Ca_Desi_SNP578 | [G/T] | *Ca_Desi_LG01* | 60.673 |
| Ca_Desi_SNP579 | [G/A] | *Ca_Desi_LG01* | 60.722 |
| Ca_Desi_SNP580 | [T/C] | *Ca_Desi_LG01* | 60.775 |
| Ca_Desi_SNP581 | [A/C] | *Ca_Desi_LG01* | 60.915 |
| Ca_Desi_SNP582 | [T/C] | *Ca_Desi_LG01* | 60.938 |
| Ca_Desi_SNP583 | [A/G] | *Ca_Desi_LG01* | 61.090 |
| Ca_Desi_SNP584 | [A/C] | *Ca_Desi_LG01* | 61.091 |
| Ca_Desi_SNP585 | [A/G] | *Ca_Desi_LG01* | 61.093 |
| Ca_Desi_SNP586 | [G/A] | *Ca_Desi_LG01* | 61.159 |
| Ca_Desi_SNP587 | [T/A] | *Ca_Desi_LG01* | 61.182 |
| Ca_Desi_SNP588 | [A/G] | *Ca_Desi_LG01* | 61.355 |
| Ca_Desi_SNP589 | [T/C] | *Ca_Desi_LG01* | 61.361 |
| Ca_Desi_SNP590 | [T/C] | *Ca_Desi_LG01* | 61.417 |
| Ca_Desi_SNP591 | [T/C] | *Ca_Desi_LG01* | 61.419 |
| Ca_Desi_SNP592 | [T/G] | *Ca_Desi_LG01* | 61.466 |
| Ca_Desi_SNP593 | [A/C] | *Ca_Desi_LG01* | 61.615 |
| Ca_Desi_SNP594 | [C/T] | *Ca_Desi_LG01* | 61.623 |
| Ca_Desi_SNP595 | [T/G] | *Ca_Desi_LG01* | 61.738 |
| Ca_Desi_SNP596 | [T/C] | *Ca_Desi_LG01* | 61.997 |
| Ca_Desi_SNP597 | [T/C] | *Ca_Desi_LG01* | 62.083 |
| Ca_Desi_SNP598 | [A/G] | *Ca_Desi_LG01* | 62.174 |
| Ca_Desi_SNP599 | [A/G] | *Ca_Desi_LG01* | 62.397 |
| Ca_Desi_SNP600 | [A/G] | *Ca_Desi_LG01* | 62.456 |
| Ca_Desi_SNP601 | [G/A] | *Ca_Desi_LG01* | 62.570 |
| Ca_Desi_SNP602 | [T/C] | *Ca_Desi_LG01* | 62.707 |
| Ca_Desi_SNP603 | [T/C] | *Ca_Desi_LG01* | 62.708 |
| Ca_Desi_SNP604 | [T/G] | *Ca_Desi_LG01* | 62.819 |
| Ca_Desi_SNP605 | [A/T] | *Ca_Desi_LG01* | 62.823 |
| Ca_Desi_SNP606 | [A/G] | *Ca_Desi_LG01* | 62.887 |
| Ca_Desi_SNP607 | [T/A] | *Ca_Desi_LG01* | 63.022 |
| Ca_Desi_SNP608 | [C/T] | *Ca_Desi_LG01* | 63.083 |
| Ca_Desi_SNP609 | [A/G] | *Ca_Desi_LG01* | 63.198 |
| Ca_Desi_SNP610 | [A/G] | *Ca_Desi_LG01* | 63.255 |
| Ca_Desi_SNP611 | [T/C] | *Ca_Desi_LG01* | 63.312 |
| Ca_Desi_SNP612 | [T/G] | *Ca_Desi_LG01* | 63.341 |
| Ca_Desi_SNP613 | [A/G] | *Ca_Desi_LG01* | 63.381 |
| Ca_Desi_SNP614 | [T/C] | *Ca_Desi_LG01* | 63.469 |
| Ca_Desi_SNP615 | [A/G] | *Ca_Desi_LG01* | 63.538 |
| Ca_Desi_SNP616 | [T/G] | *Ca_Desi_LG01* | 63.546 |
| Ca_Desi_SNP617 | [T/G] | *Ca_Desi_LG01* | 63.583 |
| Ca_Desi_SNP618 | [A/G] | *Ca_Desi_LG01* | 63.722 |
| Ca_Desi_SNP619 | [G/A] | *Ca_Desi_LG01* | 63.920 |
| Ca_Desi_SNP620 | [A/G] | *Ca_Desi_LG01* | 63.980 |
| Ca_Desi_SNP621 | [G/T] | *Ca_Desi_LG01* | 64.000 |

| **SNP IDs** | **SNPs** | **Linkage Group (LGs)** | **Genetic positions (cM) of mapped SNPs** |
|---|---|---|---|
| Ca_Desi_SNP622 | [C/T] | *Ca_Desi_LG01* | 64.053 |
| Ca_Desi_SNP623 | [A/C] | *Ca_Desi_LG01* | 64.064 |
| Ca_Desi_SNP624 | [A/C] | *Ca_Desi_LG01* | 64.169 |
| Ca_Desi_SNP625 | [T/C] | *Ca_Desi_LG01* | 64.180 |
| Ca_Desi_SNP626 | [A/G] | *Ca_Desi_LG01* | 64.183 |
| Ca_Desi_SNP627 | [T/G] | *Ca_Desi_LG01* | 64.237 |
| Ca_Desi_SNP628 | [A/G] | *Ca_Desi_LG01* | 64.307 |
| Ca_Desi_SNP629 | [A/G] | *Ca_Desi_LG01* | 64.359 |
| Ca_Desi_SNP630 | [A/C] | *Ca_Desi_LG01* | 64.397 |
| Ca_Desi_SNP631 | [G/A] | *Ca_Desi_LG01* | 64.423 |
| Ca_Desi_SNP632 | [T/G] | *Ca_Desi_LG01* | 64.462 |
| Ca_Desi_SNP633 | [A/G] | *Ca_Desi_LG01* | 64.494 |
| Ca_Desi_SNP634 | [C/T] | *Ca_Desi_LG01* | 64.545 |
| Ca_Desi_SNP635 | [C/A] | *Ca_Desi_LG01* | 64.572 |
| Ca_Desi_SNP636 | [G/A] | *Ca_Desi_LG01* | 64.655 |
| Ca_Desi_SNP637 | [T/G] | *Ca_Desi_LG01* | 64.707 |
| Ca_Desi_SNP638 | [G/A] | *Ca_Desi_LG01* | 64.817 |
| Ca_Desi_SNP639 | [A/C] | *Ca_Desi_LG01* | 64.977 |
| Ca_Desi_SNP640 | [A/G] | *Ca_Desi_LG01* | 65.111 |
| Ca_Desi_SNP641 | [A/G] | *Ca_Desi_LG01* | 65.404 |
| Ca_Desi_SNP642 | [A/C] | *Ca_Desi_LG01* | 65.547 |
| Ca_Desi_SNP643 | [C/G] | *Ca_Desi_LG01* | 65.622 |
| Ca_Desi_SNP644 | [A/G] | *Ca_Desi_LG01* | 65.630 |
| Ca_Desi_SNP645 | [T/C] | *Ca_Desi_LG01* | 65.674 |
| Ca_Desi_SNP646 | [T/C] | *Ca_Desi_LG01* | 65.773 |
| Ca_Desi_SNP647 | [C/A] | *Ca_Desi_LG01* | 65.941 |
| Ca_Desi_SNP648 | [T/C] | *Ca_Desi_LG01* | 66.014 |
| Ca_Desi_SNP649 | [A/C] | *Ca_Desi_LG01* | 66.061 |
| Ca_Desi_SNP650 | [T/G] | *Ca_Desi_LG01* | 66.206 |
| Ca_Desi_SNP651 | [A/G] | *Ca_Desi_LG01* | 66.299 |
| Ca_Desi_SNP652 | [G/A] | *Ca_Desi_LG01* | 66.744 |
| Ca_Desi_SNP653 | [A/G] | *Ca_Desi_LG01* | 66.813 |
| Ca_Desi_SNP654 | [A/C] | *Ca_Desi_LG01* | 66.876 |
| Ca_Desi_SNP655 | [T/A] | *Ca_Desi_LG01* | 67.106 |
| Ca_Desi_SNP656 | [A/C] | *Ca_Desi_LG01* | 67.134 |
| Ca_Desi_SNP657 | [T/C] | *Ca_Desi_LG01* | 67.220 |
| Ca_Desi_SNP658 | [T/C] | *Ca_Desi_LG01* | 67.262 |
| Ca_Desi_SNP659 | [T/G] | *Ca_Desi_LG01* | 67.383 |
| Ca_Desi_SNP660 | [T/A] | *Ca_Desi_LG01* | 67.535 |
| Ca_Desi_SNP661 | [T/C] | *Ca_Desi_LG01* | 67.922 |
| Ca_Desi_SNP662 | [G/C] | *Ca_Desi_LG01* | 68.187 |
| Ca_Desi_SNP663 | [A/C] | *Ca_Desi_LG01* | 68.264 |
| Ca_Desi_SNP664 | [A/G] | *Ca_Desi_LG01* | 68.342 |
| Ca_Desi_SNP665 | [T/G] | *Ca_Desi_LG01* | 68.556 |
| Ca_Desi_SNP666 | [G/A] | *Ca_Desi_LG01* | 68.593 |
| Ca_Desi_SNP667 | [T/C] | *Ca_Desi_LG01* | 68.602 |
| Ca_Desi_SNP668 | [C/T] | *Ca_Desi_LG01* | 68.874 |
| Ca_Desi_SNP669 | [T/C] | *Ca_Desi_LG01* | 68.933 |

| **SNP IDs** | **SNPs** | **Linkage Group (LGs)** | **Genetic positions (cM) of mapped SNPs** |
|---|---|---|---|
| Ca_Desi_SNP670 | [C/T] | *Ca_Desi_LG01* | 69.055 |
| Ca_Desi_SNP671 | [A/G] | *Ca_Desi_LG01* | 69.095 |
| Ca_Desi_SNP672 | [A/G] | *Ca_Desi_LG01* | 69.194 |
| Ca_Desi_SNP673 | [T/C] | *Ca_Desi_LG01* | 69.204 |
| Ca_Desi_SNP674 | [T/C] | *Ca_Desi_LG01* | 69.303 |
| Ca_Desi_SNP675 | [T/C] | *Ca_Desi_LG01* | 69.389 |
| Ca_Desi_SNP676 | [A/G] | *Ca_Desi_LG01* | 69.453 |
| Ca_Desi_SNP677 | [A/G] | *Ca_Desi_LG01* | 69.737 |
| Ca_Desi_SNP678 | [A/G] | *Ca_Desi_LG01* | 69.757 |
| Ca_Desi_SNP679 | [A/T] | *Ca_Desi_LG01* | 69.876 |
| Ca_Desi_SNP680 | [C/A] | *Ca_Desi_LG01* | 70.529 |
| Ca_Desi_SNP681 | [A/T] | *Ca_Desi_LG01* | 70.639 |
| Ca_Desi_SNP682 | [A/G] | *Ca_Desi_LG01* | 71.370 |
| Ca_Desi_SNP683 | [G/A] | *Ca_Desi_LG01* | 71.452 |
| Ca_Desi_SNP684 | [T/C] | *Ca_Desi_LG01* | 71.548 |
| Ca_Desi_SNP685 | [G/A] | *Ca_Desi_LG01* | 71.700 |
| Ca_Desi_SNP686 | [A/T] | *Ca_Desi_LG01* | 71.827 |
| Ca_Desi_SNP687 | [C/A] | *Ca_Desi_LG01* | 71.914 |
| Ca_Desi_SNP688 | [A/G] | *Ca_Desi_LG01* | 71.993 |
| Ca_Desi_SNP689 | [T/C] | *Ca_Desi_LG01* | 72.305 |
| Ca_Desi_SNP690 | [T/G] | *Ca_Desi_LG01* | 72.454 |
| Ca_Desi_SNP691 | [T/G] | *Ca_Desi_LG01* | 72.506 |
| Ca_Desi_SNP692 | [A/C] | *Ca_Desi_LG01* | 72.543 |
| Ca_Desi_SNP693 | [A/C] | *Ca_Desi_LG01* | 72.636 |
| Ca_Desi_SNP694 | [T/G] | *Ca_Desi_LG01* | 72.752 |
| Ca_Desi_SNP695 | [A/C] | *Ca_Desi_LG01* | 72.769 |
| Ca_Desi_SNP696 | [T/C] | *Ca_Desi_LG01* | 72.878 |
| Ca_Desi_SNP697 | [C/A] | *Ca_Desi_LG01* | 73.001 |
| Ca_Desi_SNP698 | [T/G] | *Ca_Desi_LG01* | 73.166 |
| Ca_Desi_SNP699 | [A/G] | *Ca_Desi_LG01* | 73.737 |
| Ca_Desi_SNP700 | [A/G] | *Ca_Desi_LG01* | 73.820 |
| Ca_Desi_SNP701 | [C/T] | *Ca_Desi_LG01* | 73.822 |
| Ca_Desi_SNP702 | [T/C] | *Ca_Desi_LG01* | 73.978 |
| Ca_Desi_SNP703 | [C/T] | *Ca_Desi_LG01* | 73.997 |
| Ca_Desi_SNP704 | [A/C] | *Ca_Desi_LG01* | 74.092 |
| Ca_Desi_SNP705 | [A/C] | *Ca_Desi_LG01* | 74.200 |
| Ca_Desi_SNP706 | [T/C] | *Ca_Desi_LG01* | 75.068 |
| Ca_Desi_SNP707 | [A/G] | *Ca_Desi_LG01* | 75.258 |
| Ca_Desi_SNP708 | [A/G] | *Ca_Desi_LG01* | 75.381 |
| Ca_Desi_SNP709 | [T/A] | *Ca_Desi_LG01* | 75.405 |
| Ca_Desi_SNP710 | [G/C] | *Ca_Desi_LG01* | 75.652 |
| Ca_Desi_SNP711 | [C/T] | *Ca_Desi_LG01* | 75.838 |
| Ca_Desi_SNP712 | [A/T] | *Ca_Desi_LG01* | 75.972 |
| Ca_Desi_SNP713 | [T/C] | *Ca_Desi_LG01* | 76.187 |
| Ca_Desi_SNP714 | [C/A] | *Ca_Desi_LG01* | 76.275 |
| Ca_Desi_SNP715 | [T/G] | *Ca_Desi_LG01* | 76.317 |
| Ca_Desi_SNP716 | [T/G] | *Ca_Desi_LG01* | 76.792 |
| Ca_Desi_SNP717 | [T/C] | *Ca_Desi_LG01* | 77.331 |

| **SNP IDs** | **SNPs** | **Linkage Group (LGs)** | **Genetic positions (cM) of mapped SNPs** |
|---|---|---|---|
| Ca_Desi_SNP718 | [A/C] | *Ca_Desi_LG01* | 77.653 |
| Ca_Desi_SNP719 | [A/G] | *Ca_Desi_LG01* | 78.635 |
| Ca_Desi_SNP720 | [A/G] | *Ca_Desi_LG01* | 78.771 |
| Ca_Desi_SNP721 | [T/G] | *Ca_Desi_LG01* | 78.995 |
| Ca_Desi_SNP722 | [T/G] | *Ca_Desi_LG01* | 79.324 |
| Ca_Desi_SNP723 | [A/G] | *Ca_Desi_LG01* | 79.431 |
| Ca_Desi_SNP724 | [G/A] | *Ca_Desi_LG01* | 80.720 |
| Ca_Desi_SNP725 | [T/G] | *Ca_Desi_LG01* | 80.791 |
| Ca_Desi_SNP726 | [T/G] | *Ca_Desi_LG01* | 80.988 |
| Ca_Desi_SNP727 | [A/C] | *Ca_Desi_LG01* | 81.116 |
| Ca_Desi_SNP728 | [C/T] | *Ca_Desi_LG01* | 81.151 |
| Ca_Desi_SNP729 | [T/C] | *Ca_Desi_LG01* | 81.184 |
| Ca_Desi_SNP730 | [C/T] | *Ca_Desi_LG01* | 81.557 |
| Ca_Desi_SNP731 | [T/C] | *Ca_Desi_LG01* | 82.277 |
| Ca_Desi_SNP732 | [A/G] | *Ca_Desi_LG01* | 83.102 |
| Ca_Desi_SNP733 | [G/A] | *Ca_Desi_LG01* | 83.657 |
| Ca_Desi_SNP734 | [A/T] | *Ca_Desi_LG01* | 84.062 |
| Ca_Desi_SNP735 | [T/C] | *Ca_Desi_LG01* | 85.443 |
| Ca_Desi_SNP736 | [A/G] | *Ca_Desi_LG01* | 86.076 |
| Ca_Desi_SNP737 | [A/G] | *Ca_Desi_LG02* | 0.000 |
| Ca_Desi_SNP738 | [A/C] | *Ca_Desi_LG02* | 1.214 |
| Ca_Desi_SNP739 | [T/C] | *Ca_Desi_LG02* | 2.230 |
| Ca_Desi_SNP740 | [C/A] | *Ca_Desi_LG02* | 2.321 |
| Ca_Desi_SNP741 | [T/A] | *Ca_Desi_LG02* | 2.705 |
| Ca_Desi_SNP742 | [A/C] | *Ca_Desi_LG02* | 2.849 |
| Ca_Desi_SNP743 | [G/C] | *Ca_Desi_LG02* | 3.178 |
| Ca_Desi_SNP744 | [T/C] | *Ca_Desi_LG02* | 3.418 |
| Ca_Desi_SNP745 | [G/A] | *Ca_Desi_LG02* | 3.585 |
| Ca_Desi_SNP746 | [G/C] | *Ca_Desi_LG02* | 4.524 |
| Ca_Desi_SNP747 | [T/G] | *Ca_Desi_LG02* | 4.552 |
| Ca_Desi_SNP748 | [A/G] | *Ca_Desi_LG02* | 5.233 |
| Ca_Desi_SNP749 | [G/A] | *Ca_Desi_LG02* | 5.373 |
| Ca_Desi_SNP750 | [T/C] | *Ca_Desi_LG02* | 5.473 |
| Ca_Desi_SNP751 | [T/C] | *Ca_Desi_LG02* | 7.891 |
| Ca_Desi_SNP752 | [G/A] | *Ca_Desi_LG02* | 9.224 |
| Ca_Desi_SNP753 | [T/C] | *Ca_Desi_LG02* | 9.275 |
| Ca_Desi_SNP754 | [G/A] | *Ca_Desi_LG02* | 10.101 |
| Ca_Desi_SNP755 | [T/C] | *Ca_Desi_LG02* | 11.016 |
| Ca_Desi_SNP756 | [T/G] | *Ca_Desi_LG02* | 11.641 |
| Ca_Desi_SNP757 | [T/C] | *Ca_Desi_LG02* | 11.958 |
| Ca_Desi_SNP758 | [T/C] | *Ca_Desi_LG02* | 12.025 |
| Ca_Desi_SNP759 | [A/C] | *Ca_Desi_LG02* | 12.451 |
| Ca_Desi_SNP760 | [C/A] | *Ca_Desi_LG02* | 12.634 |
| Ca_Desi_SNP761 | [T/G] | *Ca_Desi_LG02* | 12.680 |
| Ca_Desi_SNP762 | [C/G] | *Ca_Desi_LG02* | 13.743 |
| Ca_Desi_SNP763 | [A/G] | *Ca_Desi_LG02* | 14.012 |
| Ca_Desi_SNP764 | [T/C] | *Ca_Desi_LG02* | 14.531 |
| Ca_Desi_SNP765 | [T/C] | *Ca_Desi_LG02* | 14.980 |

| **SNP IDs** | **SNPs** | **Linkage Group (LGs)** | **Genetic positions (cM) of mapped SNPs** |
|---|---|---|---|
| Ca_Desi_SNP766 | [G/A] | *Ca_Desi_LG02* | 15.083 |
| Ca_Desi_SNP767 | [A/T] | *Ca_Desi_LG02* | 15.150 |
| Ca_Desi_SNP768 | [G/T] | *Ca_Desi_LG02* | 15.258 |
| Ca_Desi_SNP769 | [A/T] | *Ca_Desi_LG02* | 15.314 |
| Ca_Desi_SNP770 | [T/G] | *Ca_Desi_LG02* | 15.882 |
| Ca_Desi_SNP771 | [T/C] | *Ca_Desi_LG02* | 16.304 |
| Ca_Desi_SNP772 | [A/T] | *Ca_Desi_LG02* | 16.619 |
| Ca_Desi_SNP773 | [A/T] | *Ca_Desi_LG02* | 16.870 |
| Ca_Desi_SNP774 | [A/C] | *Ca_Desi_LG02* | 17.433 |
| Ca_Desi_SNP775 | [T/C] | *Ca_Desi_LG02* | 17.461 |
| Ca_Desi_SNP776 | [C/A] | *Ca_Desi_LG02* | 17.586 |
| Ca_Desi_SNP777 | [T/G] | *Ca_Desi_LG02* | 17.692 |
| Ca_Desi_SNP778 | [A/G] | *Ca_Desi_LG02* | 18.172 |
| Ca_Desi_SNP779 | [T/C] | *Ca_Desi_LG02* | 18.309 |
| Ca_Desi_SNP780 | [T/G] | *Ca_Desi_LG02* | 18.389 |
| Ca_Desi_SNP781 | [T/C] | *Ca_Desi_LG02* | 18.513 |
| Ca_Desi_SNP782 | [C/A] | *Ca_Desi_LG02* | 18.518 |
| Ca_Desi_SNP783 | [A/C] | *Ca_Desi_LG02* | 19.265 |
| Ca_Desi_SNP784 | [T/C] | *Ca_Desi_LG02* | 19.321 |
| Ca_Desi_SNP785 | [T/C] | *Ca_Desi_LG02* | 19.553 |
| Ca_Desi_SNP786 | [T/G] | *Ca_Desi_LG02* | 19.609 |
| Ca_Desi_SNP787 | [A/C] | *Ca_Desi_LG02* | 19.941 |
| Ca_Desi_SNP788 | [T/C] | *Ca_Desi_LG02* | 20.521 |
| Ca_Desi_SNP789 | [T/G] | *Ca_Desi_LG02* | 20.549 |
| Ca_Desi_SNP790 | [T/C] | *Ca_Desi_LG02* | 20.817 |
| Ca_Desi_SNP791 | [C/T] | *Ca_Desi_LG02* | 20.818 |
| Ca_Desi_SNP792 | [G/A] | *Ca_Desi_LG02* | 20.867 |
| Ca_Desi_SNP793 | [T/C] | *Ca_Desi_LG02* | 20.974 |
| Ca_Desi_SNP794 | [G/A] | *Ca_Desi_LG02* | 21.063 |
| Ca_Desi_SNP795 | [A/G] | *Ca_Desi_LG02* | 21.077 |
| Ca_Desi_SNP796 | [A/G] | *Ca_Desi_LG02* | 21.137 |
| Ca_Desi_SNP797 | [T/G] | *Ca_Desi_LG02* | 21.428 |
| Ca_Desi_SNP798 | [T/G] | *Ca_Desi_LG02* | 21.477 |
| Ca_Desi_SNP799 | [G/A] | *Ca_Desi_LG02* | 21.635 |
| Ca_Desi_SNP800 | [T/C] | *Ca_Desi_LG02* | 21.691 |
| Ca_Desi_SNP801 | [A/C] | *Ca_Desi_LG02* | 21.729 |
| Ca_Desi_SNP802 | [G/A] | *Ca_Desi_LG02* | 21.745 |
| Ca_Desi_SNP803 | [C/A] | *Ca_Desi_LG02* | 21.885 |
| Ca_Desi_SNP804 | [T/C] | *Ca_Desi_LG02* | 21.920 |
| Ca_Desi_SNP805 | [T/C] | *Ca_Desi_LG02* | 22.093 |
| Ca_Desi_SNP806 | [A/C] | *Ca_Desi_LG02* | 22.119 |
| Ca_Desi_SNP807 | [T/A] | *Ca_Desi_LG02* | 22.167 |
| Ca_Desi_SNP808 | [A/G] | *Ca_Desi_LG02* | 22.278 |
| Ca_Desi_SNP809 | [A/C] | *Ca_Desi_LG02* | 22.364 |
| Ca_Desi_SNP810 | [A/G] | *Ca_Desi_LG02* | 22.608 |
| Ca_Desi_SNP811 | [A/C] | *Ca_Desi_LG02* | 22.887 |
| Ca_Desi_SNP812 | [T/C] | *Ca_Desi_LG02* | 22.891 |
| Ca_Desi_SNP813 | [A/G] | *Ca_Desi_LG02* | 23.051 |

| **SNP IDs** | **SNPs** | **Linkage Group (LGs)** | **Genetic positions (cM) of mapped SNPs** |
|---|---|---|---|
| Ca_Desi_SNP814 | [T/C] | *Ca_Desi_LG02* | 23.058 |
| Ca_Desi_SNP815 | [C/T] | *Ca_Desi_LG02* | 23.124 |
| Ca_Desi_SNP816 | [A/G] | *Ca_Desi_LG02* | 23.432 |
| Ca_Desi_SNP817 | [G/A] | *Ca_Desi_LG02* | 23.490 |
| Ca_Desi_SNP818 | [A/G] | *Ca_Desi_LG02* | 23.629 |
| Ca_Desi_SNP819 | [T/C] | *Ca_Desi_LG02* | 23.857 |
| Ca_Desi_SNP820 | [T/C] | *Ca_Desi_LG02* | 24.070 |
| Ca_Desi_SNP821 | [T/G] | *Ca_Desi_LG02* | 24.119 |
| Ca_Desi_SNP822 | [A/G] | *Ca_Desi_LG02* | 24.210 |
| Ca_Desi_SNP823 | [G/A] | *Ca_Desi_LG02* | 24.800 |
| Ca_Desi_SNP824 | [A/G] | *Ca_Desi_LG02* | 24.898 |
| Ca_Desi_SNP825 | [T/C] | *Ca_Desi_LG02* | 25.050 |
| Ca_Desi_SNP826 | [T/A] | *Ca_Desi_LG02* | 25.066 |
| Ca_Desi_SNP827 | [T/A] | *Ca_Desi_LG02* | 25.120 |
| Ca_Desi_SNP828 | [A/G] | *Ca_Desi_LG02* | 25.309 |
| Ca_Desi_SNP829 | [T/C] | *Ca_Desi_LG02* | 25.333 |
| Ca_Desi_SNP830 | [A/G] | *Ca_Desi_LG02* | 25.439 |
| Ca_Desi_SNP831 | [C/T] | *Ca_Desi_LG02* | 25.855 |
| Ca_Desi_SNP832 | [A/C] | *Ca_Desi_LG02* | 25.904 |
| Ca_Desi_SNP833 | [A/C] | *Ca_Desi_LG02* | 25.933 |
| Ca_Desi_SNP834 | [A/T] | *Ca_Desi_LG02* | 26.100 |
| Ca_Desi_SNP835 | [C/G] | *Ca_Desi_LG02* | 26.656 |
| Ca_Desi_SNP836 | [T/A] | *Ca_Desi_LG02* | 27.158 |
| Ca_Desi_SNP837 | [A/G] | *Ca_Desi_LG02* | 27.533 |
| Ca_Desi_SNP838 | [C/T] | *Ca_Desi_LG02* | 27.628 |
| Ca_Desi_SNP839 | [G/A] | *Ca_Desi_LG02* | 27.721 |
| Ca_Desi_SNP840 | [A/G] | *Ca_Desi_LG02* | 27.733 |
| Ca_Desi_SNP841 | [A/C] | *Ca_Desi_LG02* | 27.796 |
| Ca_Desi_SNP842 | [G/A] | *Ca_Desi_LG02* | 28.259 |
| Ca_Desi_SNP843 | [T/C] | *Ca_Desi_LG02* | 28.908 |
| Ca_Desi_SNP844 | [G/A] | *Ca_Desi_LG02* | 29.047 |
| Ca_Desi_SNP845 | [T/A] | *Ca_Desi_LG02* | 29.183 |
| Ca_Desi_SNP846 | [T/G] | *Ca_Desi_LG02* | 29.245 |
| Ca_Desi_SNP847 | [A/G] | *Ca_Desi_LG02* | 29.256 |
| Ca_Desi_SNP848 | [A/G] | *Ca_Desi_LG02* | 29.496 |
| Ca_Desi_SNP849 | [A/C] | *Ca_Desi_LG02* | 29.763 |
| Ca_Desi_SNP850 | [G/A] | *Ca_Desi_LG02* | 29.795 |
| Ca_Desi_SNP851 | [T/A] | *Ca_Desi_LG02* | 29.852 |
| Ca_Desi_SNP852 | [A/T] | *Ca_Desi_LG02* | 29.921 |
| Ca_Desi_SNP853 | [T/G] | *Ca_Desi_LG02* | 30.107 |
| Ca_Desi_SNP854 | [T/C] | *Ca_Desi_LG02* | 30.425 |
| Ca_Desi_SNP855 | [T/C] | *Ca_Desi_LG02* | 30.539 |
| Ca_Desi_SNP856 | [A/G] | *Ca_Desi_LG02* | 30.705 |
| Ca_Desi_SNP857 | [C/A] | *Ca_Desi_LG02* | 31.030 |
| Ca_Desi_SNP858 | [A/C] | *Ca_Desi_LG02* | 31.546 |
| Ca_Desi_SNP859 | [A/C] | *Ca_Desi_LG02* | 31.685 |
| Ca_Desi_SNP860 | [C/A] | *Ca_Desi_LG02* | 31.885 |
| Ca_Desi_SNP861 | [T/C] | *Ca_Desi_LG02* | 32.043 |

| **SNP IDs** | **SNPs** | **Linkage Group (LGs)** | **Genetic positions (cM) of mapped SNPs** |
|---|---|---|---|
| Ca_Desi_SNP862 | [A/G] | *Ca_Desi_LG02* | 32.138 |
| Ca_Desi_SNP863 | [T/C] | *Ca_Desi_LG02* | 32.173 |
| Ca_Desi_SNP864 | [A/G] | *Ca_Desi_LG02* | 32.299 |
| Ca_Desi_SNP865 | [C/G] | *Ca_Desi_LG02* | 32.838 |
| Ca_Desi_SNP866 | [A/G] | *Ca_Desi_LG02* | 33.033 |
| Ca_Desi_SNP867 | [T/G] | *Ca_Desi_LG02* | 33.064 |
| Ca_Desi_SNP868 | [C/A] | *Ca_Desi_LG02* | 33.317 |
| Ca_Desi_SNP869 | [A/G] | *Ca_Desi_LG02* | 33.349 |
| Ca_Desi_SNP870 | [T/C] | *Ca_Desi_LG02* | 33.681 |
| Ca_Desi_SNP871 | [A/C] | *Ca_Desi_LG02* | 33.849 |
| Ca_Desi_SNP872 | [T/C] | *Ca_Desi_LG02* | 33.916 |
| Ca_Desi_SNP873 | [C/T] | *Ca_Desi_LG02* | 34.145 |
| Ca_Desi_SNP874 | [T/C] | *Ca_Desi_LG02* | 34.232 |
| Ca_Desi_SNP875 | [T/G] | *Ca_Desi_LG02* | 34.764 |
| Ca_Desi_SNP876 | [T/G] | *Ca_Desi_LG02* | 34.918 |
| Ca_Desi_SNP877 | [T/G] | *Ca_Desi_LG02* | 34.948 |
| Ca_Desi_SNP878 | [A/C] | *Ca_Desi_LG02* | 35.093 |
| Ca_Desi_SNP879 | [T/G] | *Ca_Desi_LG02* | 35.137 |
| Ca_Desi_SNP880 | [G/T] | *Ca_Desi_LG02* | 35.342 |
| Ca_Desi_SNP881 | [T/G] | *Ca_Desi_LG02* | 35.542 |
| Ca_Desi_SNP882 | [A/C] | *Ca_Desi_LG02* | 35.612 |
| Ca_Desi_SNP883 | [C/T] | *Ca_Desi_LG02* | 36.356 |
| Ca_Desi_SNP884 | [A/G] | *Ca_Desi_LG02* | 36.425 |
| Ca_Desi_SNP885 | [T/G] | *Ca_Desi_LG02* | 36.447 |
| Ca_Desi_SNP886 | [C/T] | *Ca_Desi_LG02* | 36.827 |
| Ca_Desi_SNP887 | [T/C] | *Ca_Desi_LG02* | 36.944 |
| Ca_Desi_SNP888 | [A/G] | *Ca_Desi_LG02* | 37.013 |
| Ca_Desi_SNP889 | [T/C] | *Ca_Desi_LG02* | 37.014 |
| Ca_Desi_SNP890 | [C/T] | *Ca_Desi_LG02* | 37.154 |
| Ca_Desi_SNP891 | [T/G] | *Ca_Desi_LG02* | 37.288 |
| Ca_Desi_SNP892 | [T/G] | *Ca_Desi_LG02* | 37.346 |
| Ca_Desi_SNP893 | [T/C] | *Ca_Desi_LG02* | 37.627 |
| Ca_Desi_SNP894 | [A/T] | *Ca_Desi_LG02* | 37.650 |
| Ca_Desi_SNP895 | [A/G] | *Ca_Desi_LG02* | 37.724 |
| Ca_Desi_SNP896 | [A/G] | *Ca_Desi_LG02* | 37.791 |
| Ca_Desi_SNP897 | [C/A] | *Ca_Desi_LG02* | 38.127 |
| Ca_Desi_SNP898 | [A/T] | *Ca_Desi_LG02* | 38.647 |
| Ca_Desi_SNP899 | [A/C] | *Ca_Desi_LG02* | 38.934 |
| Ca_Desi_SNP900 | [A/C] | *Ca_Desi_LG02* | 38.993 |
| Ca_Desi_SNP901 | [G/A] | *Ca_Desi_LG02* | 39.121 |
| Ca_Desi_SNP902 | [A/G] | *Ca_Desi_LG02* | 39.180 |
| Ca_Desi_SNP903 | [G/T] | *Ca_Desi_LG02* | 39.185 |
| Ca_Desi_SNP904 | [T/G] | *Ca_Desi_LG02* | 39.252 |
| Ca_Desi_SNP905 | [T/G] | *Ca_Desi_LG02* | 39.293 |
| Ca_Desi_SNP906 | [A/G] | *Ca_Desi_LG02* | 39.444 |
| Ca_Desi_SNP907 | [C/T] | *Ca_Desi_LG02* | 39.562 |
| Ca_Desi_SNP908 | [A/C] | *Ca_Desi_LG02* | 39.624 |
| Ca_Desi_SNP909 | [A/C] | *Ca_Desi_LG02* | 39.980 |

| **SNP IDs** | **SNPs** | **Linkage Group (LGs)** | **Genetic positions (cM) of mapped SNPs** |
|---|---|---|---|
| Ca_Desi_SNP910 | [A/G] | *Ca_Desi_LG02* | 40.124 |
| Ca_Desi_SNP911 | [C/T] | *Ca_Desi_LG02* | 40.142 |
| Ca_Desi_SNP912 | [T/G] | *Ca_Desi_LG02* | 40.301 |
| Ca_Desi_SNP913 | [A/G] | *Ca_Desi_LG02* | 40.539 |
| Ca_Desi_SNP914 | [T/C] | *Ca_Desi_LG02* | 40.704 |
| Ca_Desi_SNP915 | [G/A] | *Ca_Desi_LG02* | 40.720 |
| Ca_Desi_SNP916 | [T/C] | *Ca_Desi_LG02* | 41.617 |
| Ca_Desi_SNP917 | [G/A] | *Ca_Desi_LG02* | 41.658 |
| Ca_Desi_SNP918 | [A/C] | *Ca_Desi_LG02* | 41.692 |
| Ca_Desi_SNP919 | [A/G] | *Ca_Desi_LG02* | 42.477 |
| Ca_Desi_SNP920 | [G/T] | *Ca_Desi_LG02* | 42.882 |
| Ca_Desi_SNP921 | [T/C] | *Ca_Desi_LG02* | 42.945 |
| Ca_Desi_SNP922 | [A/T] | *Ca_Desi_LG02* | 43.413 |
| Ca_Desi_SNP923 | [T/C] | *Ca_Desi_LG02* | 43.734 |
| Ca_Desi_SNP924 | [C/T] | *Ca_Desi_LG02* | 43.831 |
| Ca_Desi_SNP925 | [G/C] | *Ca_Desi_LG02* | 44.265 |
| Ca_Desi_SNP926 | [A/G] | *Ca_Desi_LG02* | 44.290 |
| Ca_Desi_SNP927 | [T/G] | *Ca_Desi_LG02* | 44.375 |
| Ca_Desi_SNP928 | [A/G] | *Ca_Desi_LG02* | 44.910 |
| Ca_Desi_SNP929 | [A/G] | *Ca_Desi_LG02* | 44.960 |
| Ca_Desi_SNP930 | [A/G] | *Ca_Desi_LG02* | 45.077 |
| Ca_Desi_SNP931 | [T/C] | *Ca_Desi_LG02* | 45.150 |
| Ca_Desi_SNP932 | [T/C] | *Ca_Desi_LG02* | 45.243 |
| Ca_Desi_SNP933 | [A/G] | *Ca_Desi_LG02* | 45.244 |
| Ca_Desi_SNP934 | [G/C] | *Ca_Desi_LG02* | 45.348 |
| Ca_Desi_SNP935 | [C/T] | *Ca_Desi_LG02* | 45.682 |
| Ca_Desi_SNP936 | [T/A] | *Ca_Desi_LG02* | 46.011 |
| Ca_Desi_SNP937 | [T/C] | *Ca_Desi_LG02* | 46.237 |
| Ca_Desi_SNP938 | [A/G] | *Ca_Desi_LG02* | 46.457 |
| Ca_Desi_SNP939 | [T/G] | *Ca_Desi_LG02* | 47.310 |
| Ca_Desi_SNP940 | [G/A] | *Ca_Desi_LG02* | 47.410 |
| Ca_Desi_SNP941 | [T/C] | *Ca_Desi_LG02* | 47.550 |
| Ca_Desi_SNP942 | [C/T] | *Ca_Desi_LG02* | 47.879 |
| Ca_Desi_SNP943 | [C/T] | *Ca_Desi_LG02* | 47.915 |
| Ca_Desi_SNP944 | [C/A] | *Ca_Desi_LG02* | 47.932 |
| Ca_Desi_SNP945 | [G/A] | *Ca_Desi_LG02* | 48.243 |
| Ca_Desi_SNP946 | [T/C] | *Ca_Desi_LG02* | 48.400 |
| Ca_Desi_SNP947 | [C/T] | *Ca_Desi_LG02* | 48.937 |
| Ca_Desi_SNP948 | [G/A] | *Ca_Desi_LG02* | 48.982 |
| Ca_Desi_SNP949 | [T/C] | *Ca_Desi_LG02* | 49.010 |
| Ca_Desi_SNP950 | [T/A] | *Ca_Desi_LG02* | 49.068 |
| Ca_Desi_SNP951 | [G/A] | *Ca_Desi_LG02* | 49.205 |
| Ca_Desi_SNP952 | [T/C] | *Ca_Desi_LG02* | 49.209 |
| Ca_Desi_SNP953 | [T/A] | *Ca_Desi_LG02* | 49.216 |
| Ca_Desi_SNP954 | [G/A] | *Ca_Desi_LG02* | 49.449 |
| Ca_Desi_SNP955 | [G/A] | *Ca_Desi_LG02* | 49.517 |
| Ca_Desi_SNP956 | [C/T] | *Ca_Desi_LG02* | 49.985 |
| Ca_Desi_SNP957 | [T/G] | *Ca_Desi_LG02* | 50.016 |

| **SNP IDs** | **SNPs** | **Linkage Group (LGs)** | **Genetic positions (cM) of mapped SNPs** |
|---|---|---|---|
| Ca_Desi_SNP958 | [C/A] | *Ca_Desi_LG02* | 50.036 |
| Ca_Desi_SNP959 | [C/G] | *Ca_Desi_LG02* | 50.308 |
| Ca_Desi_SNP960 | [T/C] | *Ca_Desi_LG02* | 50.363 |
| Ca_Desi_SNP961 | [T/C] | *Ca_Desi_LG02* | 50.639 |
| Ca_Desi_SNP962 | [T/C] | *Ca_Desi_LG02* | 50.648 |
| Ca_Desi_SNP963 | [G/T] | *Ca_Desi_LG02* | 50.652 |
| Ca_Desi_SNP964 | [T/G] | *Ca_Desi_LG02* | 50.946 |
| Ca_Desi_SNP965 | [C/A] | *Ca_Desi_LG02* | 51.231 |
| Ca_Desi_SNP966 | [G/A] | *Ca_Desi_LG02* | 51.885 |
| Ca_Desi_SNP967 | [A/C] | *Ca_Desi_LG02* | 51.989 |
| Ca_Desi_SNP968 | [G/C] | *Ca_Desi_LG02* | 52.048 |
| Ca_Desi_SNP969 | [T/A] | *Ca_Desi_LG02* | 52.068 |
| Ca_Desi_SNP970 | [T/C] | *Ca_Desi_LG02* | 52.123 |
| Ca_Desi_SNP971 | [A/G] | *Ca_Desi_LG02* | 52.349 |
| Ca_Desi_SNP972 | [A/T] | *Ca_Desi_LG02* | 52.638 |
| Ca_Desi_SNP973 | [C/A] | *Ca_Desi_LG02* | 52.651 |
| Ca_Desi_SNP974 | [T/G] | *Ca_Desi_LG02* | 52.735 |
| Ca_Desi_SNP975 | [G/T] | *Ca_Desi_LG02* | 53.175 |
| Ca_Desi_SNP976 | [C/T] | *Ca_Desi_LG02* | 53.193 |
| Ca_Desi_SNP977 | [T/G] | *Ca_Desi_LG02* | 53.228 |
| Ca_Desi_SNP978 | [T/C] | *Ca_Desi_LG02* | 53.302 |
| Ca_Desi_SNP979 | [T/G] | *Ca_Desi_LG02* | 53.826 |
| Ca_Desi_SNP980 | [G/T] | *Ca_Desi_LG02* | 53.899 |
| Ca_Desi_SNP981 | [A/G] | *Ca_Desi_LG02* | 54.043 |
| Ca_Desi_SNP982 | [A/G] | *Ca_Desi_LG02* | 54.108 |
| Ca_Desi_SNP983 | [C/T] | *Ca_Desi_LG02* | 54.195 |
| Ca_Desi_SNP984 | [G/T] | *Ca_Desi_LG02* | 54.289 |
| Ca_Desi_SNP985 | [C/A] | *Ca_Desi_LG02* | 54.437 |
| Ca_Desi_SNP986 | [G/A] | *Ca_Desi_LG02* | 54.503 |
| Ca_Desi_SNP987 | [C/G] | *Ca_Desi_LG02* | 54.567 |
| Ca_Desi_SNP988 | [G/A] | *Ca_Desi_LG02* | 54.772 |
| Ca_Desi_SNP989 | [A/T] | *Ca_Desi_LG02* | 54.802 |
| Ca_Desi_SNP990 | [A/C] | *Ca_Desi_LG02* | 54.924 |
| Ca_Desi_SNP991 | [A/G] | *Ca_Desi_LG02* | 55.085 |
| Ca_Desi_SNP992 | [T/C] | *Ca_Desi_LG02* | 55.249 |
| Ca_Desi_SNP993 | [G/A] | *Ca_Desi_LG02* | 55.257 |
| Ca_Desi_SNP994 | [C/A] | *Ca_Desi_LG02* | 55.312 |
| Ca_Desi_SNP995 | [T/A] | *Ca_Desi_LG02* | 55.927 |
| Ca_Desi_SNP996 | [A/G] | *Ca_Desi_LG02* | 55.981 |
| Ca_Desi_SNP997 | [A/T] | *Ca_Desi_LG02* | 55.986 |
| Ca_Desi_SNP998 | [A/G] | *Ca_Desi_LG02* | 56.016 |
| Ca_Desi_SNP999 | [G/T] | *Ca_Desi_LG02* | 56.089 |
| Ca_Desi_SNP1000 | [G/A] | *Ca_Desi_LG02* | 56.102 |
| Ca_Desi_SNP1001 | [T/G] | *Ca_Desi_LG02* | 56.228 |
| Ca_Desi_SNP1002 | [A/C] | *Ca_Desi_LG02* | 56.264 |
| Ca_Desi_SNP1003 | [A/G] | *Ca_Desi_LG02* | 56.473 |
| Ca_Desi_SNP1004 | [T/C] | *Ca_Desi_LG02* | 56.846 |
| Ca_Desi_SNP1005 | [T/G] | *Ca_Desi_LG02* | 57.359 |

| **SNP IDs** | **SNPs** | **Linkage Group (LGs)** | **Genetic positions (cM) of mapped SNPs** |
|---|---|---|---|
| Ca_Desi_SNP1006 | [C/T] | *Ca_Desi_LG02* | 57.412 |
| Ca_Desi_SNP1007 | [C/A] | *Ca_Desi_LG02* | 57.551 |
| Ca_Desi_SNP1008 | [A/T] | *Ca_Desi_LG02* | 57.713 |
| Ca_Desi_SNP1009 | [G/A] | *Ca_Desi_LG02* | 57.750 |
| Ca_Desi_SNP1010 | [T/G] | *Ca_Desi_LG02* | 57.777 |
| Ca_Desi_SNP1011 | [A/C] | *Ca_Desi_LG02* | 58.051 |
| Ca_Desi_SNP1012 | [G/A] | *Ca_Desi_LG02* | 58.324 |
| Ca_Desi_SNP1013 | [A/T] | *Ca_Desi_LG02* | 58.332 |
| Ca_Desi_SNP1014 | [G/C] | *Ca_Desi_LG02* | 58.377 |
| Ca_Desi_SNP1015 | [A/C] | *Ca_Desi_LG02* | 58.567 |
| Ca_Desi_SNP1016 | [T/G] | *Ca_Desi_LG02* | 58.677 |
| Ca_Desi_SNP1017 | [C/A] | *Ca_Desi_LG02* | 58.762 |
| Ca_Desi_SNP1018 | [T/G] | *Ca_Desi_LG02* | 58.762 |
| Ca_Desi_SNP1019 | [C/T] | *Ca_Desi_LG02* | 58.922 |
| Ca_Desi_SNP1020 | [T/C] | *Ca_Desi_LG02* | 58.949 |
| Ca_Desi_SNP1021 | [C/T] | *Ca_Desi_LG02* | 59.223 |
| Ca_Desi_SNP1022 | [T/A] | *Ca_Desi_LG02* | 59.346 |
| Ca_Desi_SNP1023 | [T/A] | *Ca_Desi_LG02* | 59.390 |
| Ca_Desi_SNP1024 | [T/A] | *Ca_Desi_LG02* | 59.450 |
| Ca_Desi_SNP1025 | [T/G] | *Ca_Desi_LG02* | 59.468 |
| Ca_Desi_SNP1026 | [A/C] | *Ca_Desi_LG02* | 59.628 |
| Ca_Desi_SNP1027 | [C/G] | *Ca_Desi_LG02* | 59.686 |
| Ca_Desi_SNP1028 | [T/C] | *Ca_Desi_LG02* | 59.829 |
| Ca_Desi_SNP1029 | [A/G] | *Ca_Desi_LG02* | 59.951 |
| Ca_Desi_SNP1030 | [C/A] | *Ca_Desi_LG02* | 59.959 |
| Ca_Desi_SNP1031 | [T/C] | *Ca_Desi_LG02* | 60.029 |
| Ca_Desi_SNP1032 | [C/T] | *Ca_Desi_LG02* | 60.051 |
| Ca_Desi_SNP1033 | [G/A] | *Ca_Desi_LG02* | 60.245 |
| Ca_Desi_SNP1034 | [T/A] | *Ca_Desi_LG02* | 60.455 |
| Ca_Desi_SNP1035 | [A/G] | *Ca_Desi_LG02* | 60.602 |
| Ca_Desi_SNP1036 | [T/A] | *Ca_Desi_LG02* | 60.686 |
| Ca_Desi_SNP1037 | [G/T] | *Ca_Desi_LG02* | 60.867 |
| Ca_Desi_SNP1038 | [T/C] | *Ca_Desi_LG02* | 60.894 |
| Ca_Desi_SNP1039 | [T/G] | *Ca_Desi_LG02* | 60.983 |
| Ca_Desi_SNP1040 | [A/G] | *Ca_Desi_LG02* | 61.095 |
| Ca_Desi_SNP1041 | [G/A] | *Ca_Desi_LG02* | 61.202 |
| Ca_Desi_SNP1042 | [G/T] | *Ca_Desi_LG02* | 61.380 |
| Ca_Desi_SNP1043 | [G/A] | *Ca_Desi_LG02* | 61.623 |
| Ca_Desi_SNP1044 | [G/T] | *Ca_Desi_LG02* | 61.640 |
| Ca_Desi_SNP1045 | [C/T] | *Ca_Desi_LG02* | 61.801 |
| Ca_Desi_SNP1046 | [A/G] | *Ca_Desi_LG02* | 62.123 |
| Ca_Desi_SNP1047 | [C/T] | *Ca_Desi_LG02* | 62.540 |
| Ca_Desi_SNP1048 | [A/G] | *Ca_Desi_LG02* | 62.579 |
| Ca_Desi_SNP1049 | [T/C] | *Ca_Desi_LG02* | 62.887 |
| Ca_Desi_SNP1050 | [C/A] | *Ca_Desi_LG02* | 63.252 |
| Ca_Desi_SNP1051 | [G/C] | *Ca_Desi_LG02* | 63.283 |
| Ca_Desi_SNP1052 | [T/C] | *Ca_Desi_LG02* | 63.294 |
| Ca_Desi_SNP1053 | [A/C] | *Ca_Desi_LG02* | 63.480 |

| **SNP IDs** | **SNPs** | **Linkage Group (LGs)** | **Genetic positions (cM) of mapped SNPs** |
|---|---|---|---|
| Ca_Desi_SNP1054 | [G/C] | *Ca_Desi_LG02* | 63.596 |
| Ca_Desi_SNP1055 | [G/A] | *Ca_Desi_LG02* | 63.960 |
| Ca_Desi_SNP1056 | [A/C] | *Ca_Desi_LG02* | 63.979 |
| Ca_Desi_SNP1057 | [T/G] | *Ca_Desi_LG02* | 64.504 |
| Ca_Desi_SNP1058 | [A/G] | *Ca_Desi_LG02* | 64.966 |
| Ca_Desi_SNP1059 | [G/A] | *Ca_Desi_LG02* | 65.037 |
| Ca_Desi_SNP1060 | [C/T] | *Ca_Desi_LG02* | 65.170 |
| Ca_Desi_SNP1061 | [T/C] | *Ca_Desi_LG02* | 65.331 |
| Ca_Desi_SNP1062 | [G/A] | *Ca_Desi_LG02* | 65.342 |
| Ca_Desi_SNP1063 | [A/T] | *Ca_Desi_LG02* | 65.470 |
| Ca_Desi_SNP1064 | [G/C] | *Ca_Desi_LG02* | 65.752 |
| Ca_Desi_SNP1065 | [C/A] | *Ca_Desi_LG02* | 65.898 |
| Ca_Desi_SNP1066 | [C/T] | *Ca_Desi_LG02* | 65.927 |
| Ca_Desi_SNP1067 | [C/A] | *Ca_Desi_LG02* | 66.023 |
| Ca_Desi_SNP1068 | [C/T] | *Ca_Desi_LG02* | 66.216 |
| Ca_Desi_SNP1069 | [T/C] | *Ca_Desi_LG02* | 66.261 |
| Ca_Desi_SNP1070 | [C/G] | *Ca_Desi_LG02* | 66.323 |
| Ca_Desi_SNP1071 | [G/T] | *Ca_Desi_LG02* | 66.454 |
| Ca_Desi_SNP1072 | [A/G] | *Ca_Desi_LG02* | 66.487 |
| Ca_Desi_SNP1073 | [T/G] | *Ca_Desi_LG02* | 66.489 |
| Ca_Desi_SNP1074 | [G/A] | *Ca_Desi_LG02* | 66.676 |
| Ca_Desi_SNP1075 | [T/G] | *Ca_Desi_LG02* | 66.885 |
| Ca_Desi_SNP1076 | [C/T] | *Ca_Desi_LG02* | 66.944 |
| Ca_Desi_SNP1077 | [A/G] | *Ca_Desi_LG02* | 67.180 |
| Ca_Desi_SNP1078 | [T/C] | *Ca_Desi_LG02* | 67.189 |
| Ca_Desi_SNP1079 | [C/A] | *Ca_Desi_LG02* | 67.473 |
| Ca_Desi_SNP1080 | [T/C] | *Ca_Desi_LG02* | 68.055 |
| Ca_Desi_SNP1081 | [T/G] | *Ca_Desi_LG02* | 68.116 |
| Ca_Desi_SNP1082 | [C/G] | *Ca_Desi_LG02* | 68.176 |
| Ca_Desi_SNP1083 | [A/C] | *Ca_Desi_LG02* | 68.702 |
| Ca_Desi_SNP1084 | [G/A] | *Ca_Desi_LG02* | 69.229 |
| Ca_Desi_SNP1085 | [T/C] | *Ca_Desi_LG02* | 69.305 |
| Ca_Desi_SNP1086 | [A/T] | *Ca_Desi_LG02* | 69.517 |
| Ca_Desi_SNP1087 | [C/A] | *Ca_Desi_LG02* | 70.652 |
| Ca_Desi_SNP1088 | [T/C] | *Ca_Desi_LG02* | 71.220 |
| Ca_Desi_SNP1089 | [G/A] | *Ca_Desi_LG02* | 71.426 |
| Ca_Desi_SNP1090 | [T/C] | *Ca_Desi_LG02* | 71.617 |
| Ca_Desi_SNP1091 | [T/C] | *Ca_Desi_LG02* | 72.225 |
| Ca_Desi_SNP1092 | [G/T] | *Ca_Desi_LG02* | 72.553 |
| Ca_Desi_SNP1093 | [T/G] | *Ca_Desi_LG02* | 72.650 |
| Ca_Desi_SNP1094 | [G/A] | *Ca_Desi_LG02* | 72.651 |
| Ca_Desi_SNP1095 | [T/C] | *Ca_Desi_LG02* | 72.654 |
| Ca_Desi_SNP1096 | [A/G] | *Ca_Desi_LG02* | 72.858 |
| Ca_Desi_SNP1097 | [T/C] | *Ca_Desi_LG02* | 73.001 |
| Ca_Desi_SNP1098 | [T/G] | *Ca_Desi_LG02* | 73.106 |
| Ca_Desi_SNP1099 | [T/C] | *Ca_Desi_LG02* | 73.744 |
| Ca_Desi_SNP1100 | [T/G] | *Ca_Desi_LG02* | 73.919 |
| Ca_Desi_SNP1101 | [A/T] | *Ca_Desi_LG02* | 74.938 |

| **SNP IDs** | **SNPs** | **Linkage Group (LGs)** | **Genetic positions (cM) of mapped SNPs** |
|---|---|---|---|
| Ca_Desi_SNP1102 | [T/G] | *Ca_Desi_LG02* | 75.118 |
| Ca_Desi_SNP1103 | [T/G] | *Ca_Desi_LG02* | 75.188 |
| Ca_Desi_SNP1104 | [A/C] | *Ca_Desi_LG02* | 75.481 |
| Ca_Desi_SNP1105 | [A/G] | *Ca_Desi_LG02* | 75.564 |
| Ca_Desi_SNP1106 | [G/A] | *Ca_Desi_LG02* | 75.604 |
| Ca_Desi_SNP1107 | [A/G] | *Ca_Desi_LG02* | 76.236 |
| Ca_Desi_SNP1108 | [T/C] | *Ca_Desi_LG02* | 76.299 |
| Ca_Desi_SNP1109 | [T/C] | *Ca_Desi_LG02* | 76.413 |
| Ca_Desi_SNP1110 | [T/G] | *Ca_Desi_LG02* | 77.015 |
| Ca_Desi_SNP1111 | [A/C] | *Ca_Desi_LG02* | 77.413 |
| Ca_Desi_SNP1112 | [G/A] | *Ca_Desi_LG02* | 78.259 |
| Ca_Desi_SNP1113 | [A/G] | *Ca_Desi_LG02* | 80.057 |
| Ca_Desi_SNP1114 | [T/C] | *Ca_Desi_LG02* | 80.649 |
| Ca_Desi_SNP1115 | [T/C] | *Ca_Desi_LG02* | 81.060 |
| Ca_Desi_SNP1116 | [A/G] | *Ca_Desi_LG02* | 81.772 |
| Ca_Desi_SNP1117 | [G/A] | *Ca_Desi_LG02* | 81.851 |
| Ca_Desi_SNP1118 | [G/A] | *Ca_Desi_LG02* | 82.194 |
| Ca_Desi_SNP1119 | [T/A] | *Ca_Desi_LG02* | 82.517 |
| Ca_Desi_SNP1120 | [T/G] | *Ca_Desi_LG02* | 82.886 |
| Ca_Desi_SNP1121 | [A/G] | *Ca_Desi_LG02* | 83.282 |
| Ca_Desi_SNP1122 | [T/G] | *Ca_Desi_LG02* | 85.662 |
| Ca_Desi_SNP1123 | [A/C] | *Ca_Desi_LG03* | 0.000 |
| Ca_Desi_SNP1124 | [A/G] | *Ca_Desi_LG03* | 0.042 |
| Ca_Desi_SNP1125 | [A/G] | *Ca_Desi_LG03* | 0.388 |
| Ca_Desi_SNP1126 | [G/A] | *Ca_Desi_LG03* | 0.565 |
| Ca_Desi_SNP1127 | [C/T] | *Ca_Desi_LG03* | 0.622 |
| Ca_Desi_SNP1128 | [A/C] | *Ca_Desi_LG03* | 1.412 |
| Ca_Desi_SNP1129 | [T/C] | *Ca_Desi_LG03* | 2.133 |
| Ca_Desi_SNP1130 | [T/C] | *Ca_Desi_LG03* | 2.815 |
| Ca_Desi_SNP1131 | [T/G] | *Ca_Desi_LG03* | 3.631 |
| Ca_Desi_SNP1132 | [G/A] | *Ca_Desi_LG03* | 3.906 |
| Ca_Desi_SNP1133 | [C/A] | *Ca_Desi_LG03* | 3.909 |
| Ca_Desi_SNP1134 | [A/G] | *Ca_Desi_LG03* | 4.790 |
| Ca_Desi_SNP1135 | [C/T] | *Ca_Desi_LG03* | 5.127 |
| Ca_Desi_SNP1136 | [C/T] | *Ca_Desi_LG03* | 5.285 |
| Ca_Desi_SNP1137 | [T/G] | *Ca_Desi_LG03* | 6.550 |
| Ca_Desi_SNP1138 | [A/G] | *Ca_Desi_LG03* | 6.609 |
| Ca_Desi_SNP1139 | [A/T] | *Ca_Desi_LG03* | 6.847 |
| Ca_Desi_SNP1140 | [T/G] | *Ca_Desi_LG03* | 7.583 |
| Ca_Desi_SNP1141 | [T/C] | *Ca_Desi_LG03* | 7.885 |
| Ca_Desi_SNP1142 | [T/C] | *Ca_Desi_LG03* | 7.896 |
| Ca_Desi_SNP1143 | [A/T] | *Ca_Desi_LG03* | 8.022 |
| Ca_Desi_SNP1144 | [A/C] | *Ca_Desi_LG03* | 8.417 |
| Ca_Desi_SNP1145 | [T/C] | *Ca_Desi_LG03* | 8.777 |
| Ca_Desi_SNP1146 | [T/A] | *Ca_Desi_LG03* | 8.906 |
| Ca_Desi_SNP1147 | [T/G] | *Ca_Desi_LG03* | 9.661 |
| Ca_Desi_SNP1148 | [A/G] | *Ca_Desi_LG03* | 10.182 |
| Ca_Desi_SNP1149 | [T/C] | *Ca_Desi_LG03* | 10.278 |

| **SNP IDs** | **SNPs** | **Linkage Group (LGs)** | **Genetic positions (cM) of mapped SNPs** |
|---|---|---|---|
| Ca_Desi_SNP1150 | [A/C] | *Ca_Desi_LG03* | 10.368 |
| Ca_Desi_SNP1151 | [T/C] | *Ca_Desi_LG03* | 11.047 |
| Ca_Desi_SNP1152 | [A/G] | *Ca_Desi_LG03* | 11.064 |
| Ca_Desi_SNP1153 | [G/A] | *Ca_Desi_LG03* | 11.104 |
| Ca_Desi_SNP1154 | [T/C] | *Ca_Desi_LG03* | 11.186 |
| Ca_Desi_SNP1155 | [A/G] | *Ca_Desi_LG03* | 11.221 |
| Ca_Desi_SNP1156 | [T/C] | *Ca_Desi_LG03* | 11.927 |
| Ca_Desi_SNP1157 | [C/T] | *Ca_Desi_LG03* | 11.982 |
| Ca_Desi_SNP1158 | [C/A] | *Ca_Desi_LG03* | 12.864 |
| Ca_Desi_SNP1159 | [C/T] | *Ca_Desi_LG03* | 13.115 |
| Ca_Desi_SNP1160 | [G/A] | *Ca_Desi_LG03* | 13.338 |
| Ca_Desi_SNP1161 | [A/G] | *Ca_Desi_LG03* | 13.354 |
| Ca_Desi_SNP1162 | [A/G] | *Ca_Desi_LG03* | 13.436 |
| Ca_Desi_SNP1163 | [T/G] | *Ca_Desi_LG03* | 13.885 |
| Ca_Desi_SNP1164 | [C/T] | *Ca_Desi_LG03* | 13.962 |
| Ca_Desi_SNP1165 | [A/C] | *Ca_Desi_LG03* | 14.156 |
| Ca_Desi_SNP1166 | [T/C] | *Ca_Desi_LG03* | 14.667 |
| Ca_Desi_SNP1167 | [T/C] | *Ca_Desi_LG03* | 14.913 |
| Ca_Desi_SNP1168 | [C/T] | *Ca_Desi_LG03* | 14.989 |
| Ca_Desi_SNP1169 | [T/G] | *Ca_Desi_LG03* | 15.361 |
| Ca_Desi_SNP1170 | [T/C] | *Ca_Desi_LG03* | 15.369 |
| Ca_Desi_SNP1171 | [A/G] | *Ca_Desi_LG03* | 15.397 |
| Ca_Desi_SNP1172 | [T/C] | *Ca_Desi_LG03* | 15.475 |
| Ca_Desi_SNP1173 | [A/C] | *Ca_Desi_LG03* | 15.809 |
| Ca_Desi_SNP1174 | [T/G] | *Ca_Desi_LG03* | 15.870 |
| Ca_Desi_SNP1175 | [A/G] | *Ca_Desi_LG03* | 15.898 |
| Ca_Desi_SNP1176 | [C/T] | *Ca_Desi_LG03* | 15.980 |
| Ca_Desi_SNP1177 | [A/G] | *Ca_Desi_LG03* | 15.981 |
| Ca_Desi_SNP1178 | [A/G] | *Ca_Desi_LG03* | 16.225 |
| Ca_Desi_SNP1179 | [T/G] | *Ca_Desi_LG03* | 16.230 |
| Ca_Desi_SNP1180 | [T/C] | *Ca_Desi_LG03* | 16.428 |
| Ca_Desi_SNP1181 | [C/T] | *Ca_Desi_LG03* | 16.583 |
| Ca_Desi_SNP1182 | [T/A] | *Ca_Desi_LG03* | 16.856 |
| Ca_Desi_SNP1183 | [C/A] | *Ca_Desi_LG03* | 17.280 |
| Ca_Desi_SNP1184 | [A/G] | *Ca_Desi_LG03* | 17.344 |
| Ca_Desi_SNP1185 | [A/G] | *Ca_Desi_LG03* | 17.349 |
| Ca_Desi_SNP1186 | [A/G] | *Ca_Desi_LG03* | 17.402 |
| Ca_Desi_SNP1187 | [A/G] | *Ca_Desi_LG03* | 17.404 |
| Ca_Desi_SNP1188 | [T/G] | *Ca_Desi_LG03* | 17.447 |
| Ca_Desi_SNP1189 | [C/G] | *Ca_Desi_LG03* | 17.620 |
| Ca_Desi_SNP1190 | [T/A] | *Ca_Desi_LG03* | 18.070 |
| Ca_Desi_SNP1191 | [A/G] | *Ca_Desi_LG03* | 18.071 |
| Ca_Desi_SNP1192 | [C/T] | *Ca_Desi_LG03* | 18.164 |
| Ca_Desi_SNP1193 | [C/G] | *Ca_Desi_LG03* | 18.172 |
| Ca_Desi_SNP1194 | [T/C] | *Ca_Desi_LG03* | 18.362 |
| Ca_Desi_SNP1195 | [T/G] | *Ca_Desi_LG03* | 18.554 |
| Ca_Desi_SNP1196 | [A/G] | *Ca_Desi_LG03* | 18.574 |
| Ca_Desi_SNP1197 | [A/T] | *Ca_Desi_LG03* | 18.641 |

| SNP IDs | SNPs | Linkage Group (LGs) | Genetic positions (cM) of mapped SNPs |
|---|---|---|---|
| Ca_Desi_SNP1198 | [A/G] | *Ca_Desi_LG03* | 18.685 |
| Ca_Desi_SNP1199 | [C/A] | *Ca_Desi_LG03* | 19.083 |
| Ca_Desi_SNP1200 | [A/G] | *Ca_Desi_LG03* | 19.088 |
| Ca_Desi_SNP1201 | [T/G] | *Ca_Desi_LG03* | 19.092 |
| Ca_Desi_SNP1202 | [G/A] | *Ca_Desi_LG03* | 19.132 |
| Ca_Desi_SNP1203 | [T/C] | *Ca_Desi_LG03* | 19.244 |
| Ca_Desi_SNP1204 | [G/A] | *Ca_Desi_LG03* | 19.666 |
| Ca_Desi_SNP1205 | [T/C] | *Ca_Desi_LG03* | 19.757 |
| Ca_Desi_SNP1206 | [T/G] | *Ca_Desi_LG03* | 19.906 |
| Ca_Desi_SNP1207 | [G/T] | *Ca_Desi_LG03* | 20.059 |
| Ca_Desi_SNP1208 | [C/T] | *Ca_Desi_LG03* | 20.158 |
| Ca_Desi_SNP1209 | [C/A] | *Ca_Desi_LG03* | 20.223 |
| Ca_Desi_SNP1210 | [C/A] | *Ca_Desi_LG03* | 20.732 |
| Ca_Desi_SNP1211 | [C/T] | *Ca_Desi_LG03* | 20.977 |
| Ca_Desi_SNP1212 | [G/A] | *Ca_Desi_LG03* | 21.043 |
| Ca_Desi_SNP1213 | [A/C] | *Ca_Desi_LG03* | 21.209 |
| Ca_Desi_SNP1214 | [G/A] | *Ca_Desi_LG03* | 21.351 |
| Ca_Desi_SNP1215 | [T/A] | *Ca_Desi_LG03* | 21.447 |
| Ca_Desi_SNP1216 | [G/T] | *Ca_Desi_LG03* | 21.495 |
| Ca_Desi_SNP1217 | [G/T] | *Ca_Desi_LG03* | 21.615 |
| Ca_Desi_SNP1218 | [A/G] | *Ca_Desi_LG03* | 21.832 |
| Ca_Desi_SNP1219 | [T/C] | *Ca_Desi_LG03* | 21.875 |
| Ca_Desi_SNP1220 | [T/G] | *Ca_Desi_LG03* | 21.901 |
| Ca_Desi_SNP1221 | [C/T] | *Ca_Desi_LG03* | 22.008 |
| Ca_Desi_SNP1222 | [C/A] | *Ca_Desi_LG03* | 22.022 |
| Ca_Desi_SNP1223 | [T/G] | *Ca_Desi_LG03* | 22.161 |
| Ca_Desi_SNP1224 | [G/A] | *Ca_Desi_LG03* | 22.180 |
| Ca_Desi_SNP1225 | [T/A] | *Ca_Desi_LG03* | 22.423 |
| Ca_Desi_SNP1226 | [T/G] | *Ca_Desi_LG03* | 22.536 |
| Ca_Desi_SNP1227 | [A/G] | *Ca_Desi_LG03* | 22.595 |
| Ca_Desi_SNP1228 | [A/C] | *Ca_Desi_LG03* | 22.679 |
| Ca_Desi_SNP1229 | [T/C] | *Ca_Desi_LG03* | 22.693 |
| Ca_Desi_SNP1230 | [T/C] | *Ca_Desi_LG03* | 22.728 |
| Ca_Desi_SNP1231 | [A/C] | *Ca_Desi_LG03* | 22.846 |
| Ca_Desi_SNP1232 | [A/C] | *Ca_Desi_LG03* | 22.995 |
| Ca_Desi_SNP1233 | [T/C] | *Ca_Desi_LG03* | 23.095 |
| Ca_Desi_SNP1234 | [A/G] | *Ca_Desi_LG03* | 23.231 |
| Ca_Desi_SNP1235 | [A/G] | *Ca_Desi_LG03* | 23.383 |
| Ca_Desi_SNP1236 | [C/T] | *Ca_Desi_LG03* | 23.412 |
| Ca_Desi_SNP1237 | [A/C] | *Ca_Desi_LG03* | 23.508 |
| Ca_Desi_SNP1238 | [C/T] | *Ca_Desi_LG03* | 23.565 |
| Ca_Desi_SNP1239 | [T/C] | *Ca_Desi_LG03* | 23.747 |
| Ca_Desi_SNP1240 | [T/C] | *Ca_Desi_LG03* | 23.787 |
| Ca_Desi_SNP1241 | [C/A] | *Ca_Desi_LG03* | 23.974 |
| Ca_Desi_SNP1242 | [T/C] | *Ca_Desi_LG03* | 24.024 |
| Ca_Desi_SNP1243 | [C/T] | *Ca_Desi_LG03* | 24.185 |
| Ca_Desi_SNP1244 | [T/G] | *Ca_Desi_LG03* | 24.399 |
| Ca_Desi_SNP1245 | [A/G] | *Ca_Desi_LG03* | 25.059 |

| SNP IDs | SNPs | Linkage Group (LGs) | Genetic positions (cM) of mapped SNPs |
|---|---|---|---|
| Ca_Desi_SNP1246 | [A/C] | *Ca_Desi_LG03* | 25.076 |
| Ca_Desi_SNP1247 | [T/C] | *Ca_Desi_LG03* | 25.216 |
| Ca_Desi_SNP1248 | [T/C] | *Ca_Desi_LG03* | 25.237 |
| Ca_Desi_SNP1249 | [T/G] | *Ca_Desi_LG03* | 25.599 |
| Ca_Desi_SNP1250 | [G/T] | *Ca_Desi_LG03* | 25.777 |
| Ca_Desi_SNP1251 | [T/C] | *Ca_Desi_LG03* | 26.358 |
| Ca_Desi_SNP1252 | [T/C] | *Ca_Desi_LG03* | 26.535 |
| Ca_Desi_SNP1253 | [T/A] | *Ca_Desi_LG03* | 26.536 |
| Ca_Desi_SNP1254 | [A/C] | *Ca_Desi_LG03* | 26.582 |
| Ca_Desi_SNP1255 | [T/G] | *Ca_Desi_LG03* | 26.736 |
| Ca_Desi_SNP1256 | [C/A] | *Ca_Desi_LG03* | 26.968 |
| Ca_Desi_SNP1257 | [G/T] | *Ca_Desi_LG03* | 27.014 |
| Ca_Desi_SNP1258 | [A/G] | *Ca_Desi_LG03* | 27.071 |
| Ca_Desi_SNP1259 | [A/T] | *Ca_Desi_LG03* | 27.143 |
| Ca_Desi_SNP1260 | [T/C] | *Ca_Desi_LG03* | 27.197 |
| Ca_Desi_SNP1261 | [A/C] | *Ca_Desi_LG03* | 27.359 |
| Ca_Desi_SNP1262 | [A/G] | *Ca_Desi_LG03* | 27.454 |
| Ca_Desi_SNP1263 | [C/A] | *Ca_Desi_LG03* | 27.480 |
| Ca_Desi_SNP1264 | [A/G] | *Ca_Desi_LG03* | 27.506 |
| Ca_Desi_SNP1265 | [A/G] | *Ca_Desi_LG03* | 27.526 |
| Ca_Desi_SNP1266 | [T/A] | *Ca_Desi_LG03* | 27.560 |
| Ca_Desi_SNP1267 | [A/G] | *Ca_Desi_LG03* | 27.583 |
| Ca_Desi_SNP1268 | [T/C] | *Ca_Desi_LG03* | 27.704 |
| Ca_Desi_SNP1269 | [C/T] | *Ca_Desi_LG03* | 27.891 |
| Ca_Desi_SNP1270 | [T/G] | *Ca_Desi_LG03* | 27.953 |
| Ca_Desi_SNP1271 | [G/A] | *Ca_Desi_LG03* | 28.006 |
| Ca_Desi_SNP1272 | [G/T] | *Ca_Desi_LG03* | 28.017 |
| Ca_Desi_SNP1273 | [A/G] | *Ca_Desi_LG03* | 28.035 |
| Ca_Desi_SNP1274 | [C/T] | *Ca_Desi_LG03* | 28.451 |
| Ca_Desi_SNP1275 | [G/T] | *Ca_Desi_LG03* | 28.511 |
| Ca_Desi_SNP1276 | [A/T] | *Ca_Desi_LG03* | 28.563 |
| Ca_Desi_SNP1277 | [A/G] | *Ca_Desi_LG03* | 28.631 |
| Ca_Desi_SNP1278 | [T/A] | *Ca_Desi_LG03* | 28.706 |
| Ca_Desi_SNP1279 | [G/A] | *Ca_Desi_LG03* | 29.153 |
| Ca_Desi_SNP1280 | [A/C] | *Ca_Desi_LG03* | 29.357 |
| Ca_Desi_SNP1281 | [A/G] | *Ca_Desi_LG03* | 29.407 |
| Ca_Desi_SNP1282 | [A/C] | *Ca_Desi_LG03* | 29.820 |
| Ca_Desi_SNP1283 | [A/G] | *Ca_Desi_LG03* | 29.869 |
| Ca_Desi_SNP1284 | [T/C] | *Ca_Desi_LG03* | 30.261 |
| Ca_Desi_SNP1285 | [T/C] | *Ca_Desi_LG03* | 30.305 |
| Ca_Desi_SNP1286 | [T/G] | *Ca_Desi_LG03* | 30.512 |
| Ca_Desi_SNP1287 | [T/G] | *Ca_Desi_LG03* | 30.670 |
| Ca_Desi_SNP1288 | [A/C] | *Ca_Desi_LG03* | 31.041 |
| Ca_Desi_SNP1289 | [T/G] | *Ca_Desi_LG03* | 31.055 |
| Ca_Desi_SNP1290 | [T/C] | *Ca_Desi_LG03* | 31.179 |
| Ca_Desi_SNP1291 | [A/G] | *Ca_Desi_LG03* | 31.261 |
| Ca_Desi_SNP1292 | [C/A] | *Ca_Desi_LG03* | 31.388 |
| Ca_Desi_SNP1293 | [A/G] | *Ca_Desi_LG03* | 31.431 |

| **SNP IDs** | **SNPs** | **Linkage Group (LGs)** | **Genetic positions (cM) of mapped SNPs** |
|---|---|---|---|
| Ca_Desi_SNP1294 | [G/T] | *Ca_Desi_LG03* | 31.493 |
| Ca_Desi_SNP1295 | [G/T] | *Ca_Desi_LG03* | 31.509 |
| Ca_Desi_SNP1296 | [G/T] | *Ca_Desi_LG03* | 31.555 |
| Ca_Desi_SNP1297 | [A/C] | *Ca_Desi_LG03* | 31.767 |
| Ca_Desi_SNP1298 | [G/C] | *Ca_Desi_LG03* | 31.841 |
| Ca_Desi_SNP1299 | [G/A] | *Ca_Desi_LG03* | 31.878 |
| Ca_Desi_SNP1300 | [A/T] | *Ca_Desi_LG03* | 31.907 |
| Ca_Desi_SNP1301 | [A/G] | *Ca_Desi_LG03* | 31.913 |
| Ca_Desi_SNP1302 | [G/T] | *Ca_Desi_LG03* | 32.195 |
| Ca_Desi_SNP1303 | [T/G] | *Ca_Desi_LG03* | 32.207 |
| Ca_Desi_SNP1304 | [C/T] | *Ca_Desi_LG03* | 32.480 |
| Ca_Desi_SNP1305 | [T/C] | *Ca_Desi_LG03* | 32.627 |
| Ca_Desi_SNP1306 | [G/A] | *Ca_Desi_LG03* | 32.689 |
| Ca_Desi_SNP1307 | [A/T] | *Ca_Desi_LG03* | 32.857 |
| Ca_Desi_SNP1308 | [T/C] | *Ca_Desi_LG03* | 33.217 |
| Ca_Desi_SNP1309 | [A/G] | *Ca_Desi_LG03* | 33.321 |
| Ca_Desi_SNP1310 | [C/A] | *Ca_Desi_LG03* | 33.602 |
| Ca_Desi_SNP1311 | [T/C] | *Ca_Desi_LG03* | 33.626 |
| Ca_Desi_SNP1312 | [G/A] | *Ca_Desi_LG03* | 33.652 |
| Ca_Desi_SNP1313 | [A/G] | *Ca_Desi_LG03* | 33.822 |
| Ca_Desi_SNP1314 | [T/C] | *Ca_Desi_LG03* | 33.922 |
| Ca_Desi_SNP1315 | [C/A] | *Ca_Desi_LG03* | 34.070 |
| Ca_Desi_SNP1316 | [C/T] | *Ca_Desi_LG03* | 34.268 |
| Ca_Desi_SNP1317 | [A/C] | *Ca_Desi_LG03* | 34.440 |
| Ca_Desi_SNP1318 | [A/C] | *Ca_Desi_LG03* | 34.449 |
| Ca_Desi_SNP1319 | [C/A] | *Ca_Desi_LG03* | 34.458 |
| Ca_Desi_SNP1320 | [T/C] | *Ca_Desi_LG03* | 34.475 |
| Ca_Desi_SNP1321 | [A/G] | *Ca_Desi_LG03* | 34.675 |
| Ca_Desi_SNP1322 | [T/G] | *Ca_Desi_LG03* | 34.753 |
| Ca_Desi_SNP1323 | [T/C] | *Ca_Desi_LG03* | 34.897 |
| Ca_Desi_SNP1324 | [G/A] | *Ca_Desi_LG03* | 35.072 |
| Ca_Desi_SNP1325 | [A/G] | *Ca_Desi_LG03* | 35.729 |
| Ca_Desi_SNP1326 | [A/C] | *Ca_Desi_LG03* | 35.994 |
| Ca_Desi_SNP1327 | [C/T] | *Ca_Desi_LG03* | 36.007 |
| Ca_Desi_SNP1328 | [G/A] | *Ca_Desi_LG03* | 36.130 |
| Ca_Desi_SNP1329 | [A/C] | *Ca_Desi_LG03* | 36.155 |
| Ca_Desi_SNP1330 | [C/T] | *Ca_Desi_LG03* | 36.189 |
| Ca_Desi_SNP1331 | [T/C] | *Ca_Desi_LG03* | 36.211 |
| Ca_Desi_SNP1332 | [G/A] | *Ca_Desi_LG03* | 36.309 |
| Ca_Desi_SNP1333 | [A/G] | *Ca_Desi_LG03* | 36.368 |
| Ca_Desi_SNP1334 | [T/G] | *Ca_Desi_LG03* | 36.372 |
| Ca_Desi_SNP1335 | [T/A] | *Ca_Desi_LG03* | 36.510 |
| Ca_Desi_SNP1336 | [A/G] | *Ca_Desi_LG03* | 36.649 |
| Ca_Desi_SNP1337 | [T/C] | *Ca_Desi_LG03* | 36.652 |
| Ca_Desi_SNP1338 | [T/C] | *Ca_Desi_LG03* | 36.704 |
| Ca_Desi_SNP1339 | [T/C] | *Ca_Desi_LG03* | 36.959 |
| Ca_Desi_SNP1340 | [G/C] | *Ca_Desi_LG03* | 37.072 |
| Ca_Desi_SNP1341 | [G/T] | *Ca_Desi_LG03* | 37.227 |

| **SNP IDs** | **SNPs** | **Linkage Group (LGs)** | **Genetic positions (cM) of mapped SNPs** |
|---|---|---|---|
| Ca_Desi_SNP1342 | [C/T] | *Ca_Desi_LG03* | 37.253 |
| Ca_Desi_SNP1343 | [A/G] | *Ca_Desi_LG03* | 37.311 |
| Ca_Desi_SNP1344 | [G/T] | *Ca_Desi_LG03* | 37.372 |
| Ca_Desi_SNP1345 | [G/A] | *Ca_Desi_LG03* | 37.438 |
| Ca_Desi_SNP1346 | [A/C] | *Ca_Desi_LG03* | 37.539 |
| Ca_Desi_SNP1347 | [T/C] | *Ca_Desi_LG03* | 37.574 |
| Ca_Desi_SNP1348 | [T/A] | *Ca_Desi_LG03* | 37.589 |
| Ca_Desi_SNP1349 | [C/T] | *Ca_Desi_LG03* | 37.635 |
| Ca_Desi_SNP1350 | [G/A] | *Ca_Desi_LG03* | 38.025 |
| Ca_Desi_SNP1351 | [A/G] | *Ca_Desi_LG03* | 38.235 |
| Ca_Desi_SNP1352 | [T/G] | *Ca_Desi_LG03* | 38.362 |
| Ca_Desi_SNP1353 | [T/C] | *Ca_Desi_LG03* | 38.464 |
| Ca_Desi_SNP1354 | [A/C] | *Ca_Desi_LG03* | 38.510 |
| Ca_Desi_SNP1355 | [T/C] | *Ca_Desi_LG03* | 38.673 |
| Ca_Desi_SNP1356 | [C/T] | *Ca_Desi_LG03* | 38.753 |
| Ca_Desi_SNP1357 | [C/A] | *Ca_Desi_LG03* | 38.855 |
| Ca_Desi_SNP1358 | [T/C] | *Ca_Desi_LG03* | 38.879 |
| Ca_Desi_SNP1359 | [A/C] | *Ca_Desi_LG03* | 39.084 |
| Ca_Desi_SNP1360 | [C/G] | *Ca_Desi_LG03* | 39.456 |
| Ca_Desi_SNP1361 | [C/T] | *Ca_Desi_LG03* | 39.533 |
| Ca_Desi_SNP1362 | [A/C] | *Ca_Desi_LG03* | 39.543 |
| Ca_Desi_SNP1363 | [A/G] | *Ca_Desi_LG03* | 39.849 |
| Ca_Desi_SNP1364 | [G/A] | *Ca_Desi_LG03* | 39.858 |
| Ca_Desi_SNP1365 | [T/G] | *Ca_Desi_LG03* | 40.054 |
| Ca_Desi_SNP1366 | [A/G] | *Ca_Desi_LG03* | 40.095 |
| Ca_Desi_SNP1367 | [T/G] | *Ca_Desi_LG03* | 40.204 |
| Ca_Desi_SNP1368 | [G/T] | *Ca_Desi_LG03* | 40.220 |
| Ca_Desi_SNP1369 | [A/C] | *Ca_Desi_LG03* | 40.405 |
| Ca_Desi_SNP1370 | [C/A] | *Ca_Desi_LG03* | 40.439 |
| Ca_Desi_SNP1371 | [A/T] | *Ca_Desi_LG03* | 40.454 |
| Ca_Desi_SNP1372 | [T/C] | *Ca_Desi_LG03* | 40.483 |
| Ca_Desi_SNP1373 | [G/T] | *Ca_Desi_LG03* | 40.586 |
| Ca_Desi_SNP1374 | [T/A] | *Ca_Desi_LG03* | 40.700 |
| Ca_Desi_SNP1375 | [G/C] | *Ca_Desi_LG03* | 40.765 |
| Ca_Desi_SNP1376 | [C/T] | *Ca_Desi_LG03* | 40.768 |
| Ca_Desi_SNP1377 | [A/C] | *Ca_Desi_LG03* | 40.972 |
| Ca_Desi_SNP1378 | [G/A] | *Ca_Desi_LG03* | 41.143 |
| Ca_Desi_SNP1379 | [C/T] | *Ca_Desi_LG03* | 41.169 |
| Ca_Desi_SNP1380 | [G/A] | *Ca_Desi_LG03* | 41.228 |
| Ca_Desi_SNP1381 | [T/G] | *Ca_Desi_LG03* | 41.229 |
| Ca_Desi_SNP1382 | [A/G] | *Ca_Desi_LG03* | 41.513 |
| Ca_Desi_SNP1383 | [G/A] | *Ca_Desi_LG03* | 41.589 |
| Ca_Desi_SNP1384 | [T/C] | *Ca_Desi_LG03* | 41.630 |
| Ca_Desi_SNP1385 | [G/A] | *Ca_Desi_LG03* | 41.631 |
| Ca_Desi_SNP1386 | [A/T] | *Ca_Desi_LG03* | 41.668 |
| Ca_Desi_SNP1387 | [C/A] | *Ca_Desi_LG03* | 41.698 |
| Ca_Desi_SNP1388 | [G/A] | *Ca_Desi_LG03* | 41.812 |
| Ca_Desi_SNP1389 | [T/C] | *Ca_Desi_LG03* | 41.890 |

| **SNP IDs** | **SNPs** | **Linkage Group (LGs)** | **Genetic positions (cM) of mapped SNPs** |
|---|---|---|---|
| Ca_Desi_SNP1390 | [T/C] | *Ca_Desi_LG03* | 41.996 |
| Ca_Desi_SNP1391 | [G/T] | *Ca_Desi_LG03* | 42.003 |
| Ca_Desi_SNP1392 | [T/G] | *Ca_Desi_LG03* | 42.050 |
| Ca_Desi_SNP1393 | [T/C] | *Ca_Desi_LG03* | 42.098 |
| Ca_Desi_SNP1394 | [T/G] | *Ca_Desi_LG03* | 42.263 |
| Ca_Desi_SNP1395 | [A/G] | *Ca_Desi_LG03* | 42.332 |
| Ca_Desi_SNP1396 | [A/C] | *Ca_Desi_LG03* | 42.344 |
| Ca_Desi_SNP1397 | [T/G] | *Ca_Desi_LG03* | 42.481 |
| Ca_Desi_SNP1398 | [T/G] | *Ca_Desi_LG03* | 42.482 |
| Ca_Desi_SNP1399 | [T/C] | *Ca_Desi_LG03* | 42.568 |
| Ca_Desi_SNP1400 | [C/T] | *Ca_Desi_LG03* | 42.627 |
| Ca_Desi_SNP1401 | [A/G] | *Ca_Desi_LG03* | 42.649 |
| Ca_Desi_SNP1402 | [C/A] | *Ca_Desi_LG03* | 42.732 |
| Ca_Desi_SNP1403 | [A/C] | *Ca_Desi_LG03* | 42.863 |
| Ca_Desi_SNP1404 | [A/G] | *Ca_Desi_LG03* | 42.866 |
| Ca_Desi_SNP1405 | [T/G] | *Ca_Desi_LG03* | 42.895 |
| Ca_Desi_SNP1406 | [A/C] | *Ca_Desi_LG03* | 42.916 |
| Ca_Desi_SNP1407 | [A/C] | *Ca_Desi_LG03* | 42.959 |
| Ca_Desi_SNP1408 | [A/T] | *Ca_Desi_LG03* | 43.049 |
| Ca_Desi_SNP1409 | [C/T] | *Ca_Desi_LG03* | 43.085 |
| Ca_Desi_SNP1410 | [T/C] | *Ca_Desi_LG03* | 43.209 |
| Ca_Desi_SNP1411 | [C/T] | *Ca_Desi_LG03* | 43.213 |
| Ca_Desi_SNP1412 | [T/C] | *Ca_Desi_LG03* | 43.269 |
| Ca_Desi_SNP1413 | [T/G] | *Ca_Desi_LG03* | 43.271 |
| Ca_Desi_SNP1414 | [T/G] | *Ca_Desi_LG03* | 43.446 |
| Ca_Desi_SNP1415 | [C/T] | *Ca_Desi_LG03* | 43.506 |
| Ca_Desi_SNP1416 | [T/G] | *Ca_Desi_LG03* | 43.746 |
| Ca_Desi_SNP1417 | [T/C] | *Ca_Desi_LG03* | 43.808 |
| Ca_Desi_SNP1418 | [T/G] | *Ca_Desi_LG03* | 43.914 |
| Ca_Desi_SNP1419 | [A/G] | *Ca_Desi_LG03* | 44.024 |
| Ca_Desi_SNP1420 | [A/C] | *Ca_Desi_LG03* | 44.078 |
| Ca_Desi_SNP1421 | [A/G] | *Ca_Desi_LG03* | 44.113 |
| Ca_Desi_SNP1422 | [T/C] | *Ca_Desi_LG03* | 44.121 |
| Ca_Desi_SNP1423 | [T/C] | *Ca_Desi_LG03* | 44.246 |
| Ca_Desi_SNP1424 | [G/A] | *Ca_Desi_LG03* | 44.399 |
| Ca_Desi_SNP1425 | [C/A] | *Ca_Desi_LG03* | 44.496 |
| Ca_Desi_SNP1426 | [A/G] | *Ca_Desi_LG03* | 44.721 |
| Ca_Desi_SNP1427 | [A/G] | *Ca_Desi_LG03* | 44.896 |
| Ca_Desi_SNP1428 | [C/A] | *Ca_Desi_LG03* | 45.030 |
| Ca_Desi_SNP1429 | [A/C] | *Ca_Desi_LG03* | 45.057 |
| Ca_Desi_SNP1430 | [T/C] | *Ca_Desi_LG03* | 45.090 |
| Ca_Desi_SNP1431 | [G/C] | *Ca_Desi_LG03* | 45.485 |
| Ca_Desi_SNP1432 | [T/C] | *Ca_Desi_LG03* | 45.553 |
| Ca_Desi_SNP1433 | [G/A] | *Ca_Desi_LG03* | 45.647 |
| Ca_Desi_SNP1434 | [T/C] | *Ca_Desi_LG03* | 45.678 |
| Ca_Desi_SNP1435 | [A/C] | *Ca_Desi_LG03* | 45.745 |
| Ca_Desi_SNP1436 | [T/C] | *Ca_Desi_LG03* | 45.829 |
| Ca_Desi_SNP1437 | [T/C] | *Ca_Desi_LG03* | 45.839 |

| **SNP IDs** | **SNPs** | **Linkage Group (LGs)** | **Genetic positions (cM) of mapped SNPs** |
|---|---|---|---|
| Ca_Desi_SNP1438 | [A/G] | *Ca_Desi_LG03* | 45.973 |
| Ca_Desi_SNP1439 | [T/G] | *Ca_Desi_LG03* | 46.132 |
| Ca_Desi_SNP1440 | [T/C] | *Ca_Desi_LG03* | 46.159 |
| Ca_Desi_SNP1441 | [C/A] | *Ca_Desi_LG03* | 46.171 |
| Ca_Desi_SNP1442 | [T/C] | *Ca_Desi_LG03* | 46.173 |
| Ca_Desi_SNP1443 | [A/C] | *Ca_Desi_LG03* | 46.466 |
| Ca_Desi_SNP1444 | [T/C] | *Ca_Desi_LG03* | 46.508 |
| Ca_Desi_SNP1445 | [A/C] | *Ca_Desi_LG03* | 46.529 |
| Ca_Desi_SNP1446 | [A/G] | *Ca_Desi_LG03* | 47.027 |
| Ca_Desi_SNP1447 | [T/C] | *Ca_Desi_LG03* | 47.074 |
| Ca_Desi_SNP1448 | [A/G] | *Ca_Desi_LG03* | 47.227 |
| Ca_Desi_SNP1449 | [T/G] | *Ca_Desi_LG03* | 47.350 |
| Ca_Desi_SNP1450 | [A/G] | *Ca_Desi_LG03* | 47.394 |
| Ca_Desi_SNP1451 | [T/C] | *Ca_Desi_LG03* | 47.687 |
| Ca_Desi_SNP1452 | [G/A] | *Ca_Desi_LG03* | 47.992 |
| Ca_Desi_SNP1453 | [A/G] | *Ca_Desi_LG03* | 48.006 |
| Ca_Desi_SNP1454 | [A/G] | *Ca_Desi_LG03* | 48.027 |
| Ca_Desi_SNP1455 | [A/C] | *Ca_Desi_LG03* | 48.038 |
| Ca_Desi_SNP1456 | [T/C] | *Ca_Desi_LG03* | 48.114 |
| Ca_Desi_SNP1457 | [A/G] | *Ca_Desi_LG03* | 48.302 |
| Ca_Desi_SNP1458 | [T/G] | *Ca_Desi_LG03* | 48.313 |
| Ca_Desi_SNP1459 | [C/T] | *Ca_Desi_LG03* | 48.353 |
| Ca_Desi_SNP1460 | [A/T] | *Ca_Desi_LG03* | 48.583 |
| Ca_Desi_SNP1461 | [C/T] | *Ca_Desi_LG03* | 48.923 |
| Ca_Desi_SNP1462 | [A/C] | *Ca_Desi_LG03* | 49.068 |
| Ca_Desi_SNP1463 | [A/G] | *Ca_Desi_LG03* | 49.174 |
| Ca_Desi_SNP1464 | [C/T] | *Ca_Desi_LG03* | 49.373 |
| Ca_Desi_SNP1465 | [T/C] | *Ca_Desi_LG03* | 49.404 |
| Ca_Desi_SNP1466 | [G/T] | *Ca_Desi_LG03* | 49.717 |
| Ca_Desi_SNP1467 | [T/C] | *Ca_Desi_LG03* | 49.973 |
| Ca_Desi_SNP1468 | [T/C] | *Ca_Desi_LG03* | 50.032 |
| Ca_Desi_SNP1469 | [G/C] | *Ca_Desi_LG03* | 50.155 |
| Ca_Desi_SNP1470 | [G/T] | *Ca_Desi_LG03* | 50.176 |
| Ca_Desi_SNP1471 | [T/C] | *Ca_Desi_LG03* | 50.212 |
| Ca_Desi_SNP1472 | [G/A] | *Ca_Desi_LG03* | 50.290 |
| Ca_Desi_SNP1473 | [G/A] | *Ca_Desi_LG03* | 50.387 |
| Ca_Desi_SNP1474 | [A/C] | *Ca_Desi_LG03* | 50.445 |
| Ca_Desi_SNP1475 | [A/G] | *Ca_Desi_LG03* | 50.489 |
| Ca_Desi_SNP1476 | [T/G] | *Ca_Desi_LG03* | 50.548 |
| Ca_Desi_SNP1477 | [A/T] | *Ca_Desi_LG03* | 50.660 |
| Ca_Desi_SNP1478 | [C/T] | *Ca_Desi_LG03* | 50.771 |
| Ca_Desi_SNP1479 | [G/A] | *Ca_Desi_LG03* | 50.792 |
| Ca_Desi_SNP1480 | [A/C] | *Ca_Desi_LG03* | 50.832 |
| Ca_Desi_SNP1481 | [T/C] | *Ca_Desi_LG03* | 50.983 |
| Ca_Desi_SNP1482 | [G/T] | *Ca_Desi_LG03* | 51.327 |
| Ca_Desi_SNP1483 | [C/T] | *Ca_Desi_LG03* | 51.953 |
| Ca_Desi_SNP1484 | [T/C] | *Ca_Desi_LG03* | 52.068 |
| Ca_Desi_SNP1485 | [T/C] | *Ca_Desi_LG03* | 52.177 |

| SNP IDs | SNPs | Linkage Group (LGs) | Genetic positions (cM) of mapped SNPs |
|---|---|---|---|
| Ca_Desi_SNP1486 | [A/G] | *Ca_Desi_LG03* | 52.290 |
| Ca_Desi_SNP1487 | [A/T] | *Ca_Desi_LG03* | 52.354 |
| Ca_Desi_SNP1488 | [T/C] | *Ca_Desi_LG03* | 52.423 |
| Ca_Desi_SNP1489 | [A/C] | *Ca_Desi_LG03* | 52.566 |
| Ca_Desi_SNP1490 | [G/T] | *Ca_Desi_LG03* | 52.585 |
| Ca_Desi_SNP1491 | [T/A] | *Ca_Desi_LG03* | 52.596 |
| Ca_Desi_SNP1492 | [G/A] | *Ca_Desi_LG03* | 53.010 |
| Ca_Desi_SNP1493 | [A/T] | *Ca_Desi_LG03* | 53.360 |
| Ca_Desi_SNP1494 | [T/A] | *Ca_Desi_LG03* | 53.525 |
| Ca_Desi_SNP1495 | [T/C] | *Ca_Desi_LG03* | 53.594 |
| Ca_Desi_SNP1496 | [A/C] | *Ca_Desi_LG03* | 53.732 |
| Ca_Desi_SNP1497 | [A/G] | *Ca_Desi_LG03* | 53.935 |
| Ca_Desi_SNP1498 | [C/A] | *Ca_Desi_LG03* | 54.051 |
| Ca_Desi_SNP1499 | [G/T] | *Ca_Desi_LG03* | 54.102 |
| Ca_Desi_SNP1500 | [A/C] | *Ca_Desi_LG03* | 54.103 |
| Ca_Desi_SNP1501 | [T/G] | *Ca_Desi_LG03* | 54.537 |
| Ca_Desi_SNP1502 | [G/A] | *Ca_Desi_LG03* | 54.788 |
| Ca_Desi_SNP1503 | [C/A] | *Ca_Desi_LG03* | 54.881 |
| Ca_Desi_SNP1504 | [A/C] | *Ca_Desi_LG03* | 54.888 |
| Ca_Desi_SNP1505 | [T/C] | *Ca_Desi_LG03* | 54.947 |
| Ca_Desi_SNP1506 | [G/T] | *Ca_Desi_LG03* | 54.973 |
| Ca_Desi_SNP1507 | [C/A] | *Ca_Desi_LG03* | 55.052 |
| Ca_Desi_SNP1508 | [A/C] | *Ca_Desi_LG03* | 55.098 |
| Ca_Desi_SNP1509 | [G/T] | *Ca_Desi_LG03* | 55.323 |
| Ca_Desi_SNP1510 | [T/G] | *Ca_Desi_LG03* | 55.363 |
| Ca_Desi_SNP1511 | [G/A] | *Ca_Desi_LG03* | 55.413 |
| Ca_Desi_SNP1512 | [A/C] | *Ca_Desi_LG03* | 55.471 |
| Ca_Desi_SNP1513 | [T/G] | *Ca_Desi_LG03* | 55.477 |
| Ca_Desi_SNP1514 | [A/C] | *Ca_Desi_LG03* | 55.480 |
| Ca_Desi_SNP1515 | [C/T] | *Ca_Desi_LG03* | 55.542 |
| Ca_Desi_SNP1516 | [T/G] | *Ca_Desi_LG03* | 55.619 |
| Ca_Desi_SNP1517 | [T/G] | *Ca_Desi_LG03* | 55.793 |
| Ca_Desi_SNP1518 | [G/T] | *Ca_Desi_LG03* | 56.118 |
| Ca_Desi_SNP1519 | [A/G] | *Ca_Desi_LG03* | 56.131 |
| Ca_Desi_SNP1520 | [A/G] | *Ca_Desi_LG03* | 56.159 |
| Ca_Desi_SNP1521 | [C/A] | *Ca_Desi_LG03* | 56.212 |
| Ca_Desi_SNP1522 | [G/A] | *Ca_Desi_LG03* | 56.306 |
| Ca_Desi_SNP1523 | [G/A] | *Ca_Desi_LG03* | 56.331 |
| Ca_Desi_SNP1524 | [C/A] | *Ca_Desi_LG03* | 56.445 |
| Ca_Desi_SNP1525 | [T/G] | *Ca_Desi_LG03* | 56.488 |
| Ca_Desi_SNP1526 | [A/C] | *Ca_Desi_LG03* | 56.674 |
| Ca_Desi_SNP1527 | [A/G] | *Ca_Desi_LG03* | 56.919 |
| Ca_Desi_SNP1528 | [G/A] | *Ca_Desi_LG03* | 57.030 |
| Ca_Desi_SNP1529 | [A/G] | *Ca_Desi_LG03* | 57.287 |
| Ca_Desi_SNP1530 | [C/G] | *Ca_Desi_LG03* | 57.291 |
| Ca_Desi_SNP1531 | [G/T] | *Ca_Desi_LG03* | 57.452 |
| Ca_Desi_SNP1532 | [A/T] | *Ca_Desi_LG03* | 57.528 |
| Ca_Desi_SNP1533 | [A/G] | *Ca_Desi_LG03* | 57.560 |

| **SNP IDs** | **SNPs** | **Linkage Group (LGs)** | **Genetic positions (cM) of mapped SNPs** |
|---|---|---|---|
| Ca_Desi_SNP1534 | [A/C] | *Ca_Desi_LG03* | 57.583 |
| Ca_Desi_SNP1535 | [G/T] | *Ca_Desi_LG03* | 57.654 |
| Ca_Desi_SNP1536 | [T/C] | *Ca_Desi_LG03* | 57.692 |
| Ca_Desi_SNP1537 | [G/T] | *Ca_Desi_LG03* | 57.702 |
| Ca_Desi_SNP1538 | [T/A] | *Ca_Desi_LG03* | 57.753 |
| Ca_Desi_SNP1539 | [G/A] | *Ca_Desi_LG03* | 57.766 |
| Ca_Desi_SNP1540 | [A/C] | *Ca_Desi_LG03* | 57.846 |
| Ca_Desi_SNP1541 | [A/C] | *Ca_Desi_LG03* | 57.924 |
| Ca_Desi_SNP1542 | [G/T] | *Ca_Desi_LG03* | 57.954 |
| Ca_Desi_SNP1543 | [T/C] | *Ca_Desi_LG03* | 57.975 |
| Ca_Desi_SNP1544 | [A/T] | *Ca_Desi_LG03* | 58.198 |
| Ca_Desi_SNP1545 | [C/T] | *Ca_Desi_LG03* | 58.212 |
| Ca_Desi_SNP1546 | [A/C] | *Ca_Desi_LG03* | 58.235 |
| Ca_Desi_SNP1547 | [A/G] | *Ca_Desi_LG03* | 58.450 |
| Ca_Desi_SNP1548 | [C/A] | *Ca_Desi_LG03* | 58.561 |
| Ca_Desi_SNP1549 | [C/A] | *Ca_Desi_LG03* | 58.674 |
| Ca_Desi_SNP1550 | [T/C] | *Ca_Desi_LG03* | 58.683 |
| Ca_Desi_SNP1551 | [T/C] | *Ca_Desi_LG03* | 58.690 |
| Ca_Desi_SNP1552 | [T/C] | *Ca_Desi_LG03* | 58.788 |
| Ca_Desi_SNP1553 | [A/C] | *Ca_Desi_LG03* | 58.856 |
| Ca_Desi_SNP1554 | [C/G] | *Ca_Desi_LG03* | 58.871 |
| Ca_Desi_SNP1555 | [T/A] | *Ca_Desi_LG03* | 59.015 |
| Ca_Desi_SNP1556 | [G/C] | *Ca_Desi_LG03* | 59.069 |
| Ca_Desi_SNP1557 | [T/G] | *Ca_Desi_LG03* | 59.354 |
| Ca_Desi_SNP1558 | [T/C] | *Ca_Desi_LG03* | 59.365 |
| Ca_Desi_SNP1559 | [G/A] | *Ca_Desi_LG03* | 59.415 |
| Ca_Desi_SNP1560 | [G/T] | *Ca_Desi_LG03* | 59.451 |
| Ca_Desi_SNP1561 | [T/C] | *Ca_Desi_LG03* | 59.489 |
| Ca_Desi_SNP1562 | [A/G] | *Ca_Desi_LG03* | 59.590 |
| Ca_Desi_SNP1563 | [A/G] | *Ca_Desi_LG03* | 59.714 |
| Ca_Desi_SNP1564 | [A/G] | *Ca_Desi_LG03* | 60.360 |
| Ca_Desi_SNP1565 | [T/C] | *Ca_Desi_LG03* | 60.412 |
| Ca_Desi_SNP1566 | [T/C] | *Ca_Desi_LG03* | 60.818 |
| Ca_Desi_SNP1567 | [A/G] | *Ca_Desi_LG03* | 61.023 |
| Ca_Desi_SNP1568 | [A/C] | *Ca_Desi_LG03* | 61.121 |
| Ca_Desi_SNP1569 | [A/C] | *Ca_Desi_LG03* | 61.147 |
| Ca_Desi_SNP1570 | [T/G] | *Ca_Desi_LG03* | 61.500 |
| Ca_Desi_SNP1571 | [T/C] | *Ca_Desi_LG03* | 61.561 |
| Ca_Desi_SNP1572 | [T/C] | *Ca_Desi_LG03* | 61.599 |
| Ca_Desi_SNP1573 | [A/G] | *Ca_Desi_LG03* | 61.777 |
| Ca_Desi_SNP1574 | [G/C] | *Ca_Desi_LG03* | 61.824 |
| Ca_Desi_SNP1575 | [C/T] | *Ca_Desi_LG03* | 61.835 |
| Ca_Desi_SNP1576 | [G/A] | *Ca_Desi_LG03* | 62.021 |
| Ca_Desi_SNP1577 | [T/A] | *Ca_Desi_LG03* | 62.031 |
| Ca_Desi_SNP1578 | [C/T] | *Ca_Desi_LG03* | 62.305 |
| Ca_Desi_SNP1579 | [G/C] | *Ca_Desi_LG03* | 62.368 |
| Ca_Desi_SNP1580 | [T/C] | *Ca_Desi_LG03* | 62.465 |
| Ca_Desi_SNP1581 | [T/G] | *Ca_Desi_LG03* | 62.509 |

| **SNP IDs** | **SNPs** | **Linkage Group (LGs)** | **Genetic positions (cM) of mapped SNPs** |
|---|---|---|---|
| Ca_Desi_SNP1582 | [A/G] | *Ca_Desi_LG03* | 62.612 |
| Ca_Desi_SNP1583 | [C/T] | *Ca_Desi_LG03* | 62.744 |
| Ca_Desi_SNP1584 | [T/G] | *Ca_Desi_LG03* | 62.995 |
| Ca_Desi_SNP1585 | [A/G] | *Ca_Desi_LG03* | 63.033 |
| Ca_Desi_SNP1586 | [T/G] | *Ca_Desi_LG03* | 63.297 |
| Ca_Desi_SNP1587 | [T/A] | *Ca_Desi_LG03* | 63.334 |
| Ca_Desi_SNP1588 | [T/C] | *Ca_Desi_LG03* | 63.485 |
| Ca_Desi_SNP1589 | [C/T] | *Ca_Desi_LG03* | 63.568 |
| Ca_Desi_SNP1590 | [A/C] | *Ca_Desi_LG03* | 63.742 |
| Ca_Desi_SNP1591 | [G/T] | *Ca_Desi_LG03* | 63.918 |
| Ca_Desi_SNP1592 | [A/G] | *Ca_Desi_LG03* | 63.973 |
| Ca_Desi_SNP1593 | [C/A] | *Ca_Desi_LG03* | 64.281 |
| Ca_Desi_SNP1594 | [A/G] | *Ca_Desi_LG03* | 64.373 |
| Ca_Desi_SNP1595 | [A/C] | *Ca_Desi_LG03* | 64.420 |
| Ca_Desi_SNP1596 | [A/C] | *Ca_Desi_LG03* | 64.660 |
| Ca_Desi_SNP1597 | [A/G] | *Ca_Desi_LG03* | 64.661 |
| Ca_Desi_SNP1598 | [A/G] | *Ca_Desi_LG03* | 64.699 |
| Ca_Desi_SNP1599 | [T/C] | *Ca_Desi_LG03* | 64.775 |
| Ca_Desi_SNP1600 | [A/G] | *Ca_Desi_LG03* | 64.911 |
| Ca_Desi_SNP1601 | [A/C] | *Ca_Desi_LG03* | 65.284 |
| Ca_Desi_SNP1602 | [A/C] | *Ca_Desi_LG03* | 65.354 |
| Ca_Desi_SNP1603 | [A/T] | *Ca_Desi_LG03* | 65.588 |
| Ca_Desi_SNP1604 | [C/T] | *Ca_Desi_LG03* | 65.605 |
| Ca_Desi_SNP1605 | [A/T] | *Ca_Desi_LG03* | 65.686 |
| Ca_Desi_SNP1606 | [A/G] | *Ca_Desi_LG03* | 65.904 |
| Ca_Desi_SNP1607 | [G/T] | *Ca_Desi_LG03* | 66.208 |
| Ca_Desi_SNP1608 | [A/G] | *Ca_Desi_LG03* | 66.345 |
| Ca_Desi_SNP1609 | [T/A] | *Ca_Desi_LG03* | 66.447 |
| Ca_Desi_SNP1610 | [G/C] | *Ca_Desi_LG03* | 66.557 |
| Ca_Desi_SNP1611 | [A/G] | *Ca_Desi_LG03* | 67.020 |
| Ca_Desi_SNP1612 | [T/C] | *Ca_Desi_LG03* | 67.204 |
| Ca_Desi_SNP1613 | [T/C] | *Ca_Desi_LG03* | 67.232 |
| Ca_Desi_SNP1614 | [A/C] | *Ca_Desi_LG03* | 67.278 |
| Ca_Desi_SNP1615 | [A/G] | *Ca_Desi_LG03* | 67.423 |
| Ca_Desi_SNP1616 | [C/G] | *Ca_Desi_LG03* | 67.755 |
| Ca_Desi_SNP1617 | [A/C] | *Ca_Desi_LG03* | 67.801 |
| Ca_Desi_SNP1618 | [A/G] | *Ca_Desi_LG03* | 67.861 |
| Ca_Desi_SNP1619 | [G/A] | *Ca_Desi_LG03* | 67.867 |
| Ca_Desi_SNP1620 | [A/C] | *Ca_Desi_LG03* | 67.958 |
| Ca_Desi_SNP1621 | [T/C] | *Ca_Desi_LG03* | 68.079 |
| Ca_Desi_SNP1622 | [C/T] | *Ca_Desi_LG03* | 68.142 |
| Ca_Desi_SNP1623 | [G/T] | *Ca_Desi_LG03* | 68.192 |
| Ca_Desi_SNP1624 | [G/A] | *Ca_Desi_LG03* | 68.300 |
| Ca_Desi_SNP1625 | [T/C] | *Ca_Desi_LG03* | 68.425 |
| Ca_Desi_SNP1626 | [T/C] | *Ca_Desi_LG03* | 69.023 |
| Ca_Desi_SNP1627 | [C/T] | *Ca_Desi_LG03* | 69.074 |
| Ca_Desi_SNP1628 | [C/T] | *Ca_Desi_LG03* | 69.175 |
| Ca_Desi_SNP1629 | [G/A] | *Ca_Desi_LG03* | 69.180 |

| **SNP IDs** | **SNPs** | **Linkage Group (LGs)** | **Genetic positions (cM) of mapped SNPs** |
|---|---|---|---|
| Ca_Desi_SNP1630 | [A/C] | *Ca_Desi_LG03* | 69.301 |
| Ca_Desi_SNP1631 | [T/G] | *Ca_Desi_LG03* | 69.564 |
| Ca_Desi_SNP1632 | [T/C] | *Ca_Desi_LG03* | 69.583 |
| Ca_Desi_SNP1633 | [T/A] | *Ca_Desi_LG03* | 69.685 |
| Ca_Desi_SNP1634 | [G/A] | *Ca_Desi_LG03* | 70.062 |
| Ca_Desi_SNP1635 | [G/A] | *Ca_Desi_LG03* | 70.088 |
| Ca_Desi_SNP1636 | [G/A] | *Ca_Desi_LG03* | 70.212 |
| Ca_Desi_SNP1637 | [C/T] | *Ca_Desi_LG03* | 71.001 |
| Ca_Desi_SNP1638 | [A/G] | *Ca_Desi_LG03* | 71.116 |
| Ca_Desi_SNP1639 | [T/C] | *Ca_Desi_LG03* | 71.229 |
| Ca_Desi_SNP1640 | [C/G] | *Ca_Desi_LG03* | 71.266 |
| Ca_Desi_SNP1641 | [A/G] | *Ca_Desi_LG03* | 71.664 |
| Ca_Desi_SNP1642 | [T/C] | *Ca_Desi_LG03* | 71.815 |
| Ca_Desi_SNP1643 | [A/G] | *Ca_Desi_LG03* | 71.971 |
| Ca_Desi_SNP1644 | [A/G] | *Ca_Desi_LG03* | 72.091 |
| Ca_Desi_SNP1645 | [T/C] | *Ca_Desi_LG03* | 72.129 |
| Ca_Desi_SNP1646 | [A/C] | *Ca_Desi_LG03* | 72.228 |
| Ca_Desi_SNP1647 | [G/C] | *Ca_Desi_LG03* | 72.445 |
| Ca_Desi_SNP1648 | [C/G] | *Ca_Desi_LG03* | 72.758 |
| Ca_Desi_SNP1649 | [T/G] | *Ca_Desi_LG03* | 72.966 |
| Ca_Desi_SNP1650 | [T/C] | *Ca_Desi_LG03* | 73.031 |
| Ca_Desi_SNP1651 | [C/A] | *Ca_Desi_LG03* | 73.304 |
| Ca_Desi_SNP1652 | [T/G] | *Ca_Desi_LG03* | 73.662 |
| Ca_Desi_SNP1653 | [T/A] | *Ca_Desi_LG03* | 73.879 |
| Ca_Desi_SNP1654 | [T/C] | *Ca_Desi_LG03* | 74.345 |
| Ca_Desi_SNP1655 | [A/G] | *Ca_Desi_LG03* | 74.424 |
| Ca_Desi_SNP1656 | [G/T] | *Ca_Desi_LG03* | 74.794 |
| Ca_Desi_SNP1657 | [G/A] | *Ca_Desi_LG03* | 75.763 |
| Ca_Desi_SNP1658 | [A/C] | *Ca_Desi_LG03* | 76.174 |
| Ca_Desi_SNP1659 | [G/A] | *Ca_Desi_LG03* | 76.280 |
| Ca_Desi_SNP1660 | [T/C] | *Ca_Desi_LG03* | 76.350 |
| Ca_Desi_SNP1661 | [T/G] | *Ca_Desi_LG03* | 76.412 |
| Ca_Desi_SNP1662 | [G/C] | *Ca_Desi_LG03* | 76.463 |
| Ca_Desi_SNP1663 | [G/T] | *Ca_Desi_LG03* | 76.500 |
| Ca_Desi_SNP1664 | [T/A] | *Ca_Desi_LG03* | 76.699 |
| Ca_Desi_SNP1665 | [T/C] | *Ca_Desi_LG03* | 76.872 |
| Ca_Desi_SNP1666 | [T/C] | *Ca_Desi_LG03* | 76.979 |
| Ca_Desi_SNP1667 | [C/A] | *Ca_Desi_LG03* | 77.398 |
| Ca_Desi_SNP1668 | [A/G] | *Ca_Desi_LG03* | 77.559 |
| Ca_Desi_SNP1669 | [T/A] | *Ca_Desi_LG03* | 77.765 |
| Ca_Desi_SNP1670 | [G/C] | *Ca_Desi_LG03* | 77.810 |
| Ca_Desi_SNP1671 | [C/T] | *Ca_Desi_LG03* | 78.486 |
| Ca_Desi_SNP1672 | [G/A] | *Ca_Desi_LG03* | 78.511 |
| Ca_Desi_SNP1673 | [A/G] | *Ca_Desi_LG03* | 78.524 |
| Ca_Desi_SNP1674 | [A/G] | *Ca_Desi_LG03* | 78.706 |
| Ca_Desi_SNP1675 | [A/G] | *Ca_Desi_LG03* | 78.712 |
| Ca_Desi_SNP1676 | [C/A] | *Ca_Desi_LG03* | 78.728 |
| Ca_Desi_SNP1677 | [C/G] | *Ca_Desi_LG03* | 78.750 |

| SNP IDs | SNPs | Linkage Group (LGs) | Genetic positions (cM) of mapped SNPs |
|---|---|---|---|
| Ca_Desi_SNP1678 | [A/C] | *Ca_Desi_LG03* | 78.982 |
| Ca_Desi_SNP1679 | [A/C] | *Ca_Desi_LG03* | 78.988 |
| Ca_Desi_SNP1680 | [C/T] | *Ca_Desi_LG03* | 79.005 |
| Ca_Desi_SNP1681 | [C/A] | *Ca_Desi_LG03* | 79.095 |
| Ca_Desi_SNP1682 | [C/A] | *Ca_Desi_LG03* | 79.229 |
| Ca_Desi_SNP1683 | [T/C] | *Ca_Desi_LG03* | 79.426 |
| Ca_Desi_SNP1684 | [A/G] | *Ca_Desi_LG03* | 79.504 |
| Ca_Desi_SNP1685 | [C/T] | *Ca_Desi_LG03* | 79.709 |
| Ca_Desi_SNP1686 | [A/G] | *Ca_Desi_LG03* | 79.757 |
| Ca_Desi_SNP1687 | [A/G] | *Ca_Desi_LG03* | 79.762 |
| Ca_Desi_SNP1688 | [C/T] | *Ca_Desi_LG03* | 80.216 |
| Ca_Desi_SNP1689 | [G/A] | *Ca_Desi_LG03* | 80.490 |
| Ca_Desi_SNP1690 | [A/G] | *Ca_Desi_LG03* | 80.779 |
| Ca_Desi_SNP1691 | [A/G] | *Ca_Desi_LG03* | 81.295 |
| Ca_Desi_SNP1692 | [A/G] | *Ca_Desi_LG03* | 81.347 |
| Ca_Desi_SNP1693 | [T/C] | *Ca_Desi_LG03* | 81.595 |
| Ca_Desi_SNP1694 | [C/T] | *Ca_Desi_LG03* | 81.619 |
| Ca_Desi_SNP1695 | [A/G] | *Ca_Desi_LG03* | 81.783 |
| Ca_Desi_SNP1696 | [C/T] | *Ca_Desi_LG03* | 81.788 |
| Ca_Desi_SNP1697 | [G/T] | *Ca_Desi_LG03* | 81.991 |
| Ca_Desi_SNP1698 | [A/G] | *Ca_Desi_LG03* | 82.001 |
| Ca_Desi_SNP1699 | [T/A] | *Ca_Desi_LG03* | 82.099 |
| Ca_Desi_SNP1700 | [C/T] | *Ca_Desi_LG03* | 82.496 |
| Ca_Desi_SNP1701 | [C/T] | *Ca_Desi_LG03* | 82.532 |
| Ca_Desi_SNP1702 | [T/C] | *Ca_Desi_LG03* | 83.322 |
| Ca_Desi_SNP1703 | [A/T] | *Ca_Desi_LG03* | 83.663 |
| Ca_Desi_SNP1704 | [T/A] | *Ca_Desi_LG03* | 83.986 |
| Ca_Desi_SNP1705 | [G/C] | *Ca_Desi_LG03* | 84.689 |
| Ca_Desi_SNP1706 | [A/G] | *Ca_Desi_LG03* | 84.731 |
| Ca_Desi_SNP1707 | [T/C] | *Ca_Desi_LG03* | 85.071 |
| Ca_Desi_SNP1708 | [C/T] | *Ca_Desi_LG03* | 85.779 |
| Ca_Desi_SNP1709 | [C/T] | *Ca_Desi_LG03* | 85.993 |
| Ca_Desi_SNP1710 | [A/G] | *Ca_Desi_LG04* | 0.000 |
| Ca_Desi_SNP1711 | [T/C] | *Ca_Desi_LG04* | 10.181 |
| Ca_Desi_SNP1712 | [G/C] | *Ca_Desi_LG04* | 10.511 |
| Ca_Desi_SNP1713 | [T/C] | *Ca_Desi_LG04* | 10.735 |
| Ca_Desi_SNP1714 | [A/T] | *Ca_Desi_LG04* | 11.298 |
| Ca_Desi_SNP1715 | [G/T] | *Ca_Desi_LG04* | 12.115 |
| Ca_Desi_SNP1716 | [T/A] | *Ca_Desi_LG04* | 12.456 |
| Ca_Desi_SNP1717 | [C/A] | *Ca_Desi_LG04* | 13.273 |
| Ca_Desi_SNP1718 | [T/C] | *Ca_Desi_LG04* | 13.702 |
| Ca_Desi_SNP1719 | [T/C] | *Ca_Desi_LG04* | 14.874 |
| Ca_Desi_SNP1720 | [T/C] | *Ca_Desi_LG04* | 15.130 |
| Ca_Desi_SNP1721 | [G/T] | *Ca_Desi_LG04* | 15.983 |
| Ca_Desi_SNP1722 | [G/A] | *Ca_Desi_LG04* | 16.006 |
| Ca_Desi_SNP1723 | [G/T] | *Ca_Desi_LG04* | 16.074 |
| Ca_Desi_SNP1724 | [A/C] | *Ca_Desi_LG04* | 16.304 |
| Ca_Desi_SNP1725 | [C/T] | *Ca_Desi_LG04* | 16.742 |

| SNP IDs | SNPs | Linkage Group (LGs) | Genetic positions (cM) of mapped SNPs |
|---|---|---|---|
| Ca_Desi_SNP1726 | [G/A] | *Ca_Desi_LG04* | 16.812 |
| Ca_Desi_SNP1727 | [C/T] | *Ca_Desi_LG04* | 17.222 |
| Ca_Desi_SNP1728 | [T/C] | *Ca_Desi_LG04* | 17.855 |
| Ca_Desi_SNP1729 | [T/G] | *Ca_Desi_LG04* | 18.028 |
| Ca_Desi_SNP1730 | [G/A] | *Ca_Desi_LG04* | 18.427 |
| Ca_Desi_SNP1731 | [A/G] | *Ca_Desi_LG04* | 18.675 |
| Ca_Desi_SNP1732 | [T/C] | *Ca_Desi_LG04* | 18.688 |
| Ca_Desi_SNP1733 | [C/T] | *Ca_Desi_LG04* | 18.859 |
| Ca_Desi_SNP1734 | [C/T] | *Ca_Desi_LG04* | 18.999 |
| Ca_Desi_SNP1735 | [T/G] | *Ca_Desi_LG04* | 19.170 |
| Ca_Desi_SNP1736 | [C/T] | *Ca_Desi_LG04* | 19.374 |
| Ca_Desi_SNP1737 | [A/T] | *Ca_Desi_LG04* | 19.467 |
| Ca_Desi_SNP1738 | [G/C] | *Ca_Desi_LG04* | 19.483 |
| Ca_Desi_SNP1739 | [C/T] | *Ca_Desi_LG04* | 20.158 |
| Ca_Desi_SNP1740 | [A/T] | *Ca_Desi_LG04* | 20.221 |
| Ca_Desi_SNP1741 | [C/T] | *Ca_Desi_LG04* | 20.302 |
| Ca_Desi_SNP1742 | [G/A] | *Ca_Desi_LG04* | 20.484 |
| Ca_Desi_SNP1743 | [G/A] | *Ca_Desi_LG04* | 20.511 |
| Ca_Desi_SNP1744 | [A/G] | *Ca_Desi_LG04* | 20.935 |
| Ca_Desi_SNP1745 | [C/T] | *Ca_Desi_LG04* | 21.144 |
| Ca_Desi_SNP1746 | [C/T] | *Ca_Desi_LG04* | 21.272 |
| Ca_Desi_SNP1747 | [T/G] | *Ca_Desi_LG04* | 21.285 |
| Ca_Desi_SNP1748 | [G/T] | *Ca_Desi_LG04* | 21.503 |
| Ca_Desi_SNP1749 | [A/T] | *Ca_Desi_LG04* | 21.757 |
| Ca_Desi_SNP1750 | [G/A] | *Ca_Desi_LG04* | 21.848 |
| Ca_Desi_SNP1751 | [A/T] | *Ca_Desi_LG04* | 21.851 |
| Ca_Desi_SNP1752 | [C/T] | *Ca_Desi_LG04* | 22.037 |
| Ca_Desi_SNP1753 | [C/G] | *Ca_Desi_LG04* | 22.104 |
| Ca_Desi_SNP1754 | [C/G] | *Ca_Desi_LG04* | 22.163 |
| Ca_Desi_SNP1755 | [T/C] | *Ca_Desi_LG04* | 22.198 |
| Ca_Desi_SNP1756 | [A/C] | *Ca_Desi_LG04* | 22.224 |
| Ca_Desi_SNP1757 | [T/C] | *Ca_Desi_LG04* | 22.238 |
| Ca_Desi_SNP1758 | [T/C] | *Ca_Desi_LG04* | 22.539 |
| Ca_Desi_SNP1759 | [T/A] | *Ca_Desi_LG04* | 22.632 |
| Ca_Desi_SNP1760 | [T/C] | *Ca_Desi_LG04* | 23.095 |
| Ca_Desi_SNP1761 | [T/C] | *Ca_Desi_LG04* | 23.267 |
| Ca_Desi_SNP1762 | [C/T] | *Ca_Desi_LG04* | 23.269 |
| Ca_Desi_SNP1763 | [G/A] | *Ca_Desi_LG04* | 23.343 |
| Ca_Desi_SNP1764 | [T/C] | *Ca_Desi_LG04* | 23.378 |
| Ca_Desi_SNP1765 | [C/T] | *Ca_Desi_LG04* | 23.422 |
| Ca_Desi_SNP1766 | [C/A] | *Ca_Desi_LG04* | 23.475 |
| Ca_Desi_SNP1767 | [A/G] | *Ca_Desi_LG04* | 23.576 |
| Ca_Desi_SNP1768 | [A/G] | *Ca_Desi_LG04* | 23.603 |
| Ca_Desi_SNP1769 | [C/T] | *Ca_Desi_LG04* | 23.780 |
| Ca_Desi_SNP1770 | [G/A] | *Ca_Desi_LG04* | 23.784 |
| Ca_Desi_SNP1771 | [A/G] | *Ca_Desi_LG04* | 24.000 |
| Ca_Desi_SNP1772 | [G/T] | *Ca_Desi_LG04* | 24.107 |
| Ca_Desi_SNP1773 | [G/C] | *Ca_Desi_LG04* | 24.244 |

| **SNP IDs** | **SNPs** | **Linkage Group (LGs)** | **Genetic positions (cM) of mapped SNPs** |
|---|---|---|---|
| Ca_Desi_SNP1774 | [T/C] | *Ca_Desi_LG04* | 24.457 |
| Ca_Desi_SNP1775 | [C/A] | *Ca_Desi_LG04* | 24.505 |
| Ca_Desi_SNP1776 | [T/C] | *Ca_Desi_LG04* | 24.839 |
| Ca_Desi_SNP1777 | [T/C] | *Ca_Desi_LG04* | 25.093 |
| Ca_Desi_SNP1778 | [C/T] | *Ca_Desi_LG04* | 25.130 |
| Ca_Desi_SNP1779 | [G/A] | *Ca_Desi_LG04* | 25.179 |
| Ca_Desi_SNP1780 | [T/C] | *Ca_Desi_LG04* | 25.218 |
| Ca_Desi_SNP1781 | [T/C] | *Ca_Desi_LG04* | 25.234 |
| Ca_Desi_SNP1782 | [C/T] | *Ca_Desi_LG04* | 25.445 |
| Ca_Desi_SNP1783 | [C/T] | *Ca_Desi_LG04* | 25.496 |
| Ca_Desi_SNP1784 | [A/G] | *Ca_Desi_LG04* | 25.676 |
| Ca_Desi_SNP1785 | [G/A] | *Ca_Desi_LG04* | 25.773 |
| Ca_Desi_SNP1786 | [C/G] | *Ca_Desi_LG04* | 25.847 |
| Ca_Desi_SNP1787 | [T/A] | *Ca_Desi_LG04* | 25.862 |
| Ca_Desi_SNP1788 | [A/G] | *Ca_Desi_LG04* | 25.939 |
| Ca_Desi_SNP1789 | [C/G] | *Ca_Desi_LG04* | 26.049 |
| Ca_Desi_SNP1790 | [T/C] | *Ca_Desi_LG04* | 26.106 |
| Ca_Desi_SNP1791 | [T/C] | *Ca_Desi_LG04* | 26.148 |
| Ca_Desi_SNP1792 | [C/G] | *Ca_Desi_LG04* | 26.305 |
| Ca_Desi_SNP1793 | [T/A] | *Ca_Desi_LG04* | 26.465 |
| Ca_Desi_SNP1794 | [A/G] | *Ca_Desi_LG04* | 26.501 |
| Ca_Desi_SNP1795 | [G/A] | *Ca_Desi_LG04* | 26.553 |
| Ca_Desi_SNP1796 | [G/A] | *Ca_Desi_LG04* | 26.730 |
| Ca_Desi_SNP1797 | [C/T] | *Ca_Desi_LG04* | 26.913 |
| Ca_Desi_SNP1798 | [T/C] | *Ca_Desi_LG04* | 26.957 |
| Ca_Desi_SNP1799 | [T/C] | *Ca_Desi_LG04* | 27.367 |
| Ca_Desi_SNP1800 | [G/T] | *Ca_Desi_LG04* | 27.393 |
| Ca_Desi_SNP1801 | [A/C] | *Ca_Desi_LG04* | 27.646 |
| Ca_Desi_SNP1802 | [T/G] | *Ca_Desi_LG04* | 27.809 |
| Ca_Desi_SNP1803 | [A/C] | *Ca_Desi_LG04* | 27.868 |
| Ca_Desi_SNP1804 | [G/T] | *Ca_Desi_LG04* | 28.494 |
| Ca_Desi_SNP1805 | [G/T] | *Ca_Desi_LG04* | 28.738 |
| Ca_Desi_SNP1806 | [A/G] | *Ca_Desi_LG04* | 28.787 |
| Ca_Desi_SNP1807 | [G/T] | *Ca_Desi_LG04* | 28.796 |
| Ca_Desi_SNP1808 | [A/T] | *Ca_Desi_LG04* | 29.122 |
| Ca_Desi_SNP1809 | [G/T] | *Ca_Desi_LG04* | 29.601 |
| Ca_Desi_SNP1810 | [A/G] | *Ca_Desi_LG04* | 30.527 |
| Ca_Desi_SNP1811 | [G/A] | *Ca_Desi_LG04* | 30.944 |
| Ca_Desi_SNP1812 | [A/T] | *Ca_Desi_LG04* | 31.979 |
| Ca_Desi_SNP1813 | [G/T] | *Ca_Desi_LG04* | 32.255 |
| Ca_Desi_SNP1814 | [T/C] | *Ca_Desi_LG04* | 32.976 |
| Ca_Desi_SNP1815 | [G/C] | *Ca_Desi_LG04* | 33.669 |
| Ca_Desi_SNP1816 | [A/G] | *Ca_Desi_LG04* | 33.742 |
| Ca_Desi_SNP1817 | [A/G] | *Ca_Desi_LG04* | 34.507 |
| Ca_Desi_SNP1818 | [T/C] | *Ca_Desi_LG04* | 34.978 |
| Ca_Desi_SNP1819 | [A/C] | *Ca_Desi_LG04* | 35.207 |
| Ca_Desi_SNP1820 | [A/G] | *Ca_Desi_LG04* | 35.604 |
| Ca_Desi_SNP1821 | [A/C] | *Ca_Desi_LG04* | 36.342 |

| SNP IDs | SNPs | Linkage Group (LGs) | Genetic positions (cM) of mapped SNPs |
|---|---|---|---|
| Ca_Desi_SNP1822 | [A/G] | *Ca_Desi_LG04* | 36.362 |
| Ca_Desi_SNP1823 | [A/G] | *Ca_Desi_LG04* | 37.198 |
| Ca_Desi_SNP1824 | [G/A] | *Ca_Desi_LG04* | 37.353 |
| Ca_Desi_SNP1825 | [T/G] | *Ca_Desi_LG04* | 38.530 |
| Ca_Desi_SNP1826 | [A/C] | *Ca_Desi_LG04* | 38.966 |
| Ca_Desi_SNP1827 | [T/A] | *Ca_Desi_LG04* | 39.302 |
| Ca_Desi_SNP1828 | [T/C] | *Ca_Desi_LG04* | 39.319 |
| Ca_Desi_SNP1829 | [T/G] | *Ca_Desi_LG04* | 39.380 |
| Ca_Desi_SNP1830 | [G/A] | *Ca_Desi_LG04* | 39.451 |
| Ca_Desi_SNP1831 | [T/G] | *Ca_Desi_LG04* | 39.648 |
| Ca_Desi_SNP1832 | [G/A] | *Ca_Desi_LG04* | 39.921 |
| Ca_Desi_SNP1833 | [C/T] | *Ca_Desi_LG04* | 40.115 |
| Ca_Desi_SNP1834 | [A/G] | *Ca_Desi_LG04* | 40.386 |
| Ca_Desi_SNP1835 | [A/C] | *Ca_Desi_LG04* | 40.401 |
| Ca_Desi_SNP1836 | [A/G] | *Ca_Desi_LG04* | 41.152 |
| Ca_Desi_SNP1837 | [A/G] | *Ca_Desi_LG04* | 41.203 |
| Ca_Desi_SNP1838 | [A/C] | *Ca_Desi_LG04* | 41.381 |
| Ca_Desi_SNP1839 | [C/A] | *Ca_Desi_LG04* | 41.421 |
| Ca_Desi_SNP1840 | [T/A] | *Ca_Desi_LG04* | 41.524 |
| Ca_Desi_SNP1841 | [T/C] | *Ca_Desi_LG04* | 42.254 |
| Ca_Desi_SNP1842 | [T/A] | *Ca_Desi_LG04* | 42.717 |
| Ca_Desi_SNP1843 | [C/T] | *Ca_Desi_LG04* | 42.994 |
| Ca_Desi_SNP1844 | [T/G] | *Ca_Desi_LG04* | 43.310 |
| Ca_Desi_SNP1845 | [C/T] | *Ca_Desi_LG04* | 43.450 |
| Ca_Desi_SNP1846 | [T/G] | *Ca_Desi_LG04* | 43.508 |
| Ca_Desi_SNP1847 | [C/T] | *Ca_Desi_LG04* | 43.518 |
| Ca_Desi_SNP1848 | [T/A] | *Ca_Desi_LG04* | 43.534 |
| Ca_Desi_SNP1849 | [G/T] | *Ca_Desi_LG04* | 43.584 |
| Ca_Desi_SNP1850 | [A/T] | *Ca_Desi_LG04* | 43.618 |
| Ca_Desi_SNP1851 | [T/A] | *Ca_Desi_LG04* | 43.641 |
| Ca_Desi_SNP1852 | [A/T] | *Ca_Desi_LG04* | 43.692 |
| Ca_Desi_SNP1853 | [C/A] | *Ca_Desi_LG04* | 43.735 |
| Ca_Desi_SNP1854 | [C/T] | *Ca_Desi_LG04* | 43.786 |
| Ca_Desi_SNP1855 | [A/G] | *Ca_Desi_LG04* | 43.799 |
| Ca_Desi_SNP1856 | [A/G] | *Ca_Desi_LG04* | 43.947 |
| Ca_Desi_SNP1857 | [T/C] | *Ca_Desi_LG04* | 43.988 |
| Ca_Desi_SNP1858 | [T/C] | *Ca_Desi_LG04* | 44.009 |
| Ca_Desi_SNP1859 | [A/G] | *Ca_Desi_LG04* | 44.015 |
| Ca_Desi_SNP1860 | [T/G] | *Ca_Desi_LG04* | 44.113 |
| Ca_Desi_SNP1861 | [A/C] | *Ca_Desi_LG04* | 44.170 |
| Ca_Desi_SNP1862 | [A/G] | *Ca_Desi_LG04* | 44.253 |
| Ca_Desi_SNP1863 | [T/C] | *Ca_Desi_LG04* | 44.351 |
| Ca_Desi_SNP1864 | [T/A] | *Ca_Desi_LG04* | 44.370 |
| Ca_Desi_SNP1865 | [G/A] | *Ca_Desi_LG04* | 44.382 |
| Ca_Desi_SNP1866 | [A/G] | *Ca_Desi_LG04* | 44.429 |
| Ca_Desi_SNP1867 | [A/C] | *Ca_Desi_LG04* | 44.433 |
| Ca_Desi_SNP1868 | [C/A] | *Ca_Desi_LG04* | 44.494 |
| Ca_Desi_SNP1869 | [G/A] | *Ca_Desi_LG04* | 44.511 |

| **SNP IDs** | **SNPs** | **Linkage Group (LGs)** | **Genetic positions (cM) of mapped SNPs** |
|---|---|---|---|
| Ca_Desi_SNP1870 | [C/A] | *Ca_Desi_LG04* | 44.645 |
| Ca_Desi_SNP1871 | [G/A] | *Ca_Desi_LG04* | 44.676 |
| Ca_Desi_SNP1872 | [T/C] | *Ca_Desi_LG04* | 44.716 |
| Ca_Desi_SNP1873 | [A/T] | *Ca_Desi_LG04* | 44.754 |
| Ca_Desi_SNP1874 | [T/C] | *Ca_Desi_LG04* | 44.759 |
| Ca_Desi_SNP1875 | [A/C] | *Ca_Desi_LG04* | 44.791 |
| Ca_Desi_SNP1876 | [T/C] | *Ca_Desi_LG04* | 44.841 |
| Ca_Desi_SNP1877 | [G/T] | *Ca_Desi_LG04* | 44.874 |
| Ca_Desi_SNP1878 | [T/G] | *Ca_Desi_LG04* | 44.907 |
| Ca_Desi_SNP1879 | [A/G] | *Ca_Desi_LG04* | 44.963 |
| Ca_Desi_SNP1880 | [A/C] | *Ca_Desi_LG04* | 45.091 |
| Ca_Desi_SNP1881 | [A/C] | *Ca_Desi_LG04* | 45.143 |
| Ca_Desi_SNP1882 | [G/A] | *Ca_Desi_LG04* | 45.362 |
| Ca_Desi_SNP1883 | [C/T] | *Ca_Desi_LG04* | 45.387 |
| Ca_Desi_SNP1884 | [A/T] | *Ca_Desi_LG04* | 45.388 |
| Ca_Desi_SNP1885 | [C/T] | *Ca_Desi_LG04* | 45.397 |
| Ca_Desi_SNP1886 | [T/A] | *Ca_Desi_LG04* | 45.470 |
| Ca_Desi_SNP1887 | [T/C] | *Ca_Desi_LG04* | 45.486 |
| Ca_Desi_SNP1888 | [T/C] | *Ca_Desi_LG04* | 45.700 |
| Ca_Desi_SNP1889 | [A/G] | *Ca_Desi_LG04* | 45.724 |
| Ca_Desi_SNP1890 | [G/A] | *Ca_Desi_LG04* | 45.828 |
| Ca_Desi_SNP1891 | [A/C] | *Ca_Desi_LG04* | 45.873 |
| Ca_Desi_SNP1892 | [C/G] | *Ca_Desi_LG04* | 45.947 |
| Ca_Desi_SNP1893 | [C/T] | *Ca_Desi_LG04* | 45.994 |
| Ca_Desi_SNP1894 | [G/A] | *Ca_Desi_LG04* | 46.016 |
| Ca_Desi_SNP1895 | [T/A] | *Ca_Desi_LG04* | 46.053 |
| Ca_Desi_SNP1896 | [G/A] | *Ca_Desi_LG04* | 46.122 |
| Ca_Desi_SNP1897 | [C/G] | *Ca_Desi_LG04* | 46.144 |
| Ca_Desi_SNP1898 | [T/A] | *Ca_Desi_LG04* | 46.460 |
| Ca_Desi_SNP1899 | [C/A] | *Ca_Desi_LG04* | 46.573 |
| Ca_Desi_SNP1900 | [T/G] | *Ca_Desi_LG04* | 46.820 |
| Ca_Desi_SNP1901 | [T/C] | *Ca_Desi_LG04* | 46.899 |
| Ca_Desi_SNP1902 | [G/A] | *Ca_Desi_LG04* | 46.899 |
| Ca_Desi_SNP1903 | [T/G] | *Ca_Desi_LG04* | 47.110 |
| Ca_Desi_SNP1904 | [T/C] | *Ca_Desi_LG04* | 47.123 |
| Ca_Desi_SNP1905 | [A/G] | *Ca_Desi_LG04* | 47.162 |
| Ca_Desi_SNP1906 | [A/T] | *Ca_Desi_LG04* | 47.236 |
| Ca_Desi_SNP1907 | [T/C] | *Ca_Desi_LG04* | 47.259 |
| Ca_Desi_SNP1908 | [C/T] | *Ca_Desi_LG04* | 47.474 |
| Ca_Desi_SNP1909 | [C/A] | *Ca_Desi_LG04* | 47.538 |
| Ca_Desi_SNP1910 | [A/G] | *Ca_Desi_LG04* | 47.538 |
| Ca_Desi_SNP1911 | [C/A] | *Ca_Desi_LG04* | 47.566 |
| Ca_Desi_SNP1912 | [T/C] | *Ca_Desi_LG04* | 47.572 |
| Ca_Desi_SNP1913 | [G/A] | *Ca_Desi_LG04* | 47.584 |
| Ca_Desi_SNP1914 | [A/G] | *Ca_Desi_LG04* | 47.605 |
| Ca_Desi_SNP1915 | [C/T] | *Ca_Desi_LG04* | 47.641 |
| Ca_Desi_SNP1916 | [G/A] | *Ca_Desi_LG04* | 47.693 |
| Ca_Desi_SNP1917 | [A/T] | *Ca_Desi_LG04* | 47.696 |

| **SNP IDs** | **SNPs** | **Linkage Group (LGs)** | **Genetic positions (cM) of mapped SNPs** |
|---|---|---|---|
| Ca_Desi_SNP1918 | [A/C] | *Ca_Desi_LG04* | 47.757 |
| Ca_Desi_SNP1919 | [T/A] | *Ca_Desi_LG04* | 47.766 |
| Ca_Desi_SNP1920 | [A/G] | *Ca_Desi_LG04* | 47.769 |
| Ca_Desi_SNP1921 | [C/A] | *Ca_Desi_LG04* | 47.822 |
| Ca_Desi_SNP1922 | [G/T] | *Ca_Desi_LG04* | 47.879 |
| Ca_Desi_SNP1923 | [C/A] | *Ca_Desi_LG04* | 48.018 |
| Ca_Desi_SNP1924 | [G/T] | *Ca_Desi_LG04* | 48.045 |
| Ca_Desi_SNP1925 | [C/T] | *Ca_Desi_LG04* | 48.048 |
| Ca_Desi_SNP1926 | [G/A] | *Ca_Desi_LG04* | 48.085 |
| Ca_Desi_SNP1927 | [G/A] | *Ca_Desi_LG04* | 48.131 |
| Ca_Desi_SNP1928 | [T/A] | *Ca_Desi_LG04* | 48.147 |
| Ca_Desi_SNP1929 | [T/A] | *Ca_Desi_LG04* | 48.152 |
| Ca_Desi_SNP1930 | [G/T] | *Ca_Desi_LG04* | 48.178 |
| Ca_Desi_SNP1931 | [A/G] | *Ca_Desi_LG04* | 48.197 |
| Ca_Desi_SNP1932 | [T/G] | *Ca_Desi_LG04* | 48.201 |
| Ca_Desi_SNP1933 | [G/T] | *Ca_Desi_LG04* | 48.263 |
| Ca_Desi_SNP1934 | [G/A] | *Ca_Desi_LG04* | 48.266 |
| Ca_Desi_SNP1935 | [T/G] | *Ca_Desi_LG04* | 48.279 |
| Ca_Desi_SNP1936 | [T/C] | *Ca_Desi_LG04* | 48.328 |
| Ca_Desi_SNP1937 | [A/G] | *Ca_Desi_LG04* | 48.442 |
| Ca_Desi_SNP1938 | [A/G] | *Ca_Desi_LG04* | 48.474 |
| Ca_Desi_SNP1939 | [A/G] | *Ca_Desi_LG04* | 48.493 |
| Ca_Desi_SNP1940 | [C/A] | *Ca_Desi_LG04* | 48.502 |
| Ca_Desi_SNP1941 | [A/G] | *Ca_Desi_LG04* | 48.542 |
| Ca_Desi_SNP1942 | [T/C] | *Ca_Desi_LG04* | 48.619 |
| Ca_Desi_SNP1943 | [G/A] | *Ca_Desi_LG04* | 48.632 |
| Ca_Desi_SNP1944 | [A/G] | *Ca_Desi_LG04* | 48.637 |
| Ca_Desi_SNP1945 | [T/A] | *Ca_Desi_LG04* | 48.726 |
| Ca_Desi_SNP1946 | [T/G] | *Ca_Desi_LG04* | 48.769 |
| Ca_Desi_SNP1947 | [A/G] | *Ca_Desi_LG04* | 48.858 |
| Ca_Desi_SNP1948 | [A/G] | *Ca_Desi_LG04* | 48.868 |
| Ca_Desi_SNP1949 | [A/C] | *Ca_Desi_LG04* | 48.928 |
| Ca_Desi_SNP1950 | [T/G] | *Ca_Desi_LG04* | 48.950 |
| Ca_Desi_SNP1951 | [C/T] | *Ca_Desi_LG04* | 48.998 |
| Ca_Desi_SNP1952 | [A/G] | *Ca_Desi_LG04* | 49.023 |
| Ca_Desi_SNP1953 | [T/C] | *Ca_Desi_LG04* | 49.040 |
| Ca_Desi_SNP1954 | [G/A] | *Ca_Desi_LG04* | 49.073 |
| Ca_Desi_SNP1955 | [A/G] | *Ca_Desi_LG04* | 49.188 |
| Ca_Desi_SNP1956 | [A/G] | *Ca_Desi_LG04* | 49.255 |
| Ca_Desi_SNP1957 | [A/G] | *Ca_Desi_LG04* | 49.291 |
| Ca_Desi_SNP1958 | [G/T] | *Ca_Desi_LG04* | 49.305 |
| Ca_Desi_SNP1959 | [T/C] | *Ca_Desi_LG04* | 49.314 |
| Ca_Desi_SNP1960 | [C/T] | *Ca_Desi_LG04* | 49.351 |
| Ca_Desi_SNP1961 | [A/C] | *Ca_Desi_LG04* | 49.409 |
| Ca_Desi_SNP1962 | [T/G] | *Ca_Desi_LG04* | 49.424 |
| Ca_Desi_SNP1963 | [C/G] | *Ca_Desi_LG04* | 49.434 |
| Ca_Desi_SNP1964 | [G/A] | *Ca_Desi_LG04* | 49.475 |
| Ca_Desi_SNP1965 | [C/A] | *Ca_Desi_LG04* | 49.511 |

| **SNP IDs** | **SNPs** | **Linkage Group (LGs)** | **Genetic positions (cM) of mapped SNPs** |
|---|---|---|---|
| Ca_Desi_SNP1966 | [G/A] | *Ca_Desi_LG04* | 49.571 |
| Ca_Desi_SNP1967 | [T/A] | *Ca_Desi_LG04* | 49.594 |
| Ca_Desi_SNP1968 | [A/G] | *Ca_Desi_LG04* | 49.618 |
| Ca_Desi_SNP1969 | [G/A] | *Ca_Desi_LG04* | 49.622 |
| Ca_Desi_SNP1970 | [T/G] | *Ca_Desi_LG04* | 49.670 |
| Ca_Desi_SNP1971 | [C/A] | *Ca_Desi_LG04* | 49.758 |
| Ca_Desi_SNP1972 | [A/G] | *Ca_Desi_LG04* | 49.762 |
| Ca_Desi_SNP1973 | [C/T] | *Ca_Desi_LG04* | 49.810 |
| Ca_Desi_SNP1974 | [C/G] | *Ca_Desi_LG04* | 49.811 |
| Ca_Desi_SNP1975 | [C/T] | *Ca_Desi_LG04* | 49.811 |
| Ca_Desi_SNP1976 | [A/C] | *Ca_Desi_LG04* | 49.815 |
| Ca_Desi_SNP1977 | [A/G] | *Ca_Desi_LG04* | 49.819 |
| Ca_Desi_SNP1978 | [A/G] | *Ca_Desi_LG04* | 49.862 |
| Ca_Desi_SNP1979 | [G/A] | *Ca_Desi_LG04* | 49.866 |
| Ca_Desi_SNP1980 | [C/G] | *Ca_Desi_LG04* | 49.872 |
| Ca_Desi_SNP1981 | [C/A] | *Ca_Desi_LG04* | 49.914 |
| Ca_Desi_SNP1982 | [G/A] | *Ca_Desi_LG04* | 49.916 |
| Ca_Desi_SNP1983 | [G/T] | *Ca_Desi_LG04* | 49.936 |
| Ca_Desi_SNP1984 | [C/G] | *Ca_Desi_LG04* | 49.994 |
| Ca_Desi_SNP1985 | [A/T] | *Ca_Desi_LG04* | 49.997 |
| Ca_Desi_SNP1986 | [T/G] | *Ca_Desi_LG04* | 50.085 |
| Ca_Desi_SNP1987 | [A/T] | *Ca_Desi_LG04* | 50.088 |
| Ca_Desi_SNP1988 | [A/G] | *Ca_Desi_LG04* | 50.200 |
| Ca_Desi_SNP1989 | [A/G] | *Ca_Desi_LG04* | 50.249 |
| Ca_Desi_SNP1990 | [A/C] | *Ca_Desi_LG04* | 50.315 |
| Ca_Desi_SNP1991 | [A/T] | *Ca_Desi_LG04* | 50.316 |
| Ca_Desi_SNP1992 | [C/A] | *Ca_Desi_LG04* | 50.347 |
| Ca_Desi_SNP1993 | [C/T] | *Ca_Desi_LG04* | 50.381 |
| Ca_Desi_SNP1994 | [A/G] | *Ca_Desi_LG04* | 50.388 |
| Ca_Desi_SNP1995 | [G/T] | *Ca_Desi_LG04* | 50.410 |
| Ca_Desi_SNP1996 | [A/C] | *Ca_Desi_LG04* | 50.420 |
| Ca_Desi_SNP1997 | [T/C] | *Ca_Desi_LG04* | 50.637 |
| Ca_Desi_SNP1998 | [T/C] | *Ca_Desi_LG04* | 50.659 |
| Ca_Desi_SNP1999 | [G/A] | *Ca_Desi_LG04* | 50.667 |
| Ca_Desi_SNP2000 | [G/A] | *Ca_Desi_LG04* | 50.693 |
| Ca_Desi_SNP2001 | [T/G] | *Ca_Desi_LG04* | 50.695 |
| Ca_Desi_SNP2002 | [C/A] | *Ca_Desi_LG04* | 50.719 |
| Ca_Desi_SNP2003 | [G/C] | *Ca_Desi_LG04* | 50.730 |
| Ca_Desi_SNP2004 | [T/C] | *Ca_Desi_LG04* | 50.848 |
| Ca_Desi_SNP2005 | [C/G] | *Ca_Desi_LG04* | 50.849 |
| Ca_Desi_SNP2006 | [A/G] | *Ca_Desi_LG04* | 50.860 |
| Ca_Desi_SNP2007 | [C/T] | *Ca_Desi_LG04* | 50.969 |
| Ca_Desi_SNP2008 | [C/G] | *Ca_Desi_LG04* | 51.036 |
| Ca_Desi_SNP2009 | [A/G] | *Ca_Desi_LG04* | 51.119 |
| Ca_Desi_SNP2010 | [G/T] | *Ca_Desi_LG04* | 51.282 |
| Ca_Desi_SNP2011 | [C/A] | *Ca_Desi_LG04* | 51.285 |
| Ca_Desi_SNP2012 | [A/T] | *Ca_Desi_LG04* | 51.287 |
| Ca_Desi_SNP2013 | [T/C] | *Ca_Desi_LG04* | 51.308 |

| SNP IDs | SNPs | Linkage Group (LGs) | Genetic positions (cM) of mapped SNPs |
|---|---|---|---|
| Ca_Desi_SNP2014 | [T/A] | *Ca_Desi_LG04* | 51.372 |
| Ca_Desi_SNP2015 | [A/C] | *Ca_Desi_LG04* | 51.431 |
| Ca_Desi_SNP2016 | [A/G] | *Ca_Desi_LG04* | 51.437 |
| Ca_Desi_SNP2017 | [C/T] | *Ca_Desi_LG04* | 51.473 |
| Ca_Desi_SNP2018 | [T/G] | *Ca_Desi_LG04* | 51.504 |
| Ca_Desi_SNP2019 | [T/C] | *Ca_Desi_LG04* | 51.565 |
| Ca_Desi_SNP2020 | [A/G] | *Ca_Desi_LG04* | 51.584 |
| Ca_Desi_SNP2021 | [T/C] | *Ca_Desi_LG04* | 51.598 |
| Ca_Desi_SNP2022 | [C/A] | *Ca_Desi_LG04* | 51.632 |
| Ca_Desi_SNP2023 | [C/T] | *Ca_Desi_LG04* | 51.702 |
| Ca_Desi_SNP2024 | [T/C] | *Ca_Desi_LG04* | 51.713 |
| Ca_Desi_SNP2025 | [A/C] | *Ca_Desi_LG04* | 51.717 |
| Ca_Desi_SNP2026 | [C/G] | *Ca_Desi_LG04* | 51.799 |
| Ca_Desi_SNP2027 | [T/G] | *Ca_Desi_LG04* | 51.835 |
| Ca_Desi_SNP2028 | [A/T] | *Ca_Desi_LG04* | 51.893 |
| Ca_Desi_SNP2029 | [A/G] | *Ca_Desi_LG04* | 51.899 |
| Ca_Desi_SNP2030 | [A/T] | *Ca_Desi_LG04* | 51.933 |
| Ca_Desi_SNP2031 | [T/C] | *Ca_Desi_LG04* | 52.042 |
| Ca_Desi_SNP2032 | [T/C] | *Ca_Desi_LG04* | 52.079 |
| Ca_Desi_SNP2033 | [A/G] | *Ca_Desi_LG04* | 52.168 |
| Ca_Desi_SNP2034 | [A/C] | *Ca_Desi_LG04* | 52.195 |
| Ca_Desi_SNP2035 | [T/C] | *Ca_Desi_LG04* | 52.283 |
| Ca_Desi_SNP2036 | [T/C] | *Ca_Desi_LG04* | 52.307 |
| Ca_Desi_SNP2037 | [T/A] | *Ca_Desi_LG04* | 52.307 |
| Ca_Desi_SNP2038 | [G/A] | *Ca_Desi_LG04* | 52.311 |
| Ca_Desi_SNP2039 | [T/A] | *Ca_Desi_LG04* | 52.330 |
| Ca_Desi_SNP2040 | [C/T] | *Ca_Desi_LG04* | 52.483 |
| Ca_Desi_SNP2041 | [G/C] | *Ca_Desi_LG04* | 52.749 |
| Ca_Desi_SNP2042 | [C/T] | *Ca_Desi_LG04* | 52.793 |
| Ca_Desi_SNP2043 | [C/T] | *Ca_Desi_LG04* | 52.900 |
| Ca_Desi_SNP2044 | [G/A] | *Ca_Desi_LG04* | 52.965 |
| Ca_Desi_SNP2045 | [A/G] | *Ca_Desi_LG04* | 53.152 |
| Ca_Desi_SNP2046 | [A/C] | *Ca_Desi_LG04* | 53.171 |
| Ca_Desi_SNP2047 | [C/T] | *Ca_Desi_LG04* | 53.216 |
| Ca_Desi_SNP2048 | [T/C] | *Ca_Desi_LG04* | 53.479 |
| Ca_Desi_SNP2049 | [C/T] | *Ca_Desi_LG04* | 53.518 |
| Ca_Desi_SNP2050 | [T/C] | *Ca_Desi_LG04* | 53.592 |
| Ca_Desi_SNP2051 | [T/C] | *Ca_Desi_LG04* | 53.685 |
| Ca_Desi_SNP2052 | [A/G] | *Ca_Desi_LG04* | 53.740 |
| Ca_Desi_SNP2053 | [T/C] | *Ca_Desi_LG04* | 53.805 |
| Ca_Desi_SNP2054 | [T/C] | *Ca_Desi_LG04* | 53.830 |
| Ca_Desi_SNP2055 | [G/A] | *Ca_Desi_LG04* | 53.954 |
| Ca_Desi_SNP2056 | [T/G] | *Ca_Desi_LG04* | 54.021 |
| Ca_Desi_SNP2057 | [T/A] | *Ca_Desi_LG04* | 54.089 |
| Ca_Desi_SNP2058 | [C/A] | *Ca_Desi_LG04* | 54.161 |
| Ca_Desi_SNP2059 | [G/T] | *Ca_Desi_LG04* | 54.167 |
| Ca_Desi_SNP2060 | [T/C] | *Ca_Desi_LG04* | 54.181 |
| Ca_Desi_SNP2061 | [C/T] | *Ca_Desi_LG04* | 54.237 |

| **SNP IDs** | **SNPs** | **Linkage Group (LGs)** | **Genetic positions (cM) of mapped SNPs** |
|---|---|---|---|
| Ca_Desi_SNP2062 | [C/G] | *Ca_Desi_LG04* | 54.263 |
| Ca_Desi_SNP2063 | [A/T] | *Ca_Desi_LG04* | 54.309 |
| Ca_Desi_SNP2064 | [C/T] | *Ca_Desi_LG04* | 54.352 |
| Ca_Desi_SNP2065 | [A/T] | *Ca_Desi_LG04* | 54.360 |
| Ca_Desi_SNP2066 | [A/C] | *Ca_Desi_LG04* | 54.370 |
| Ca_Desi_SNP2067 | [T/G] | *Ca_Desi_LG04* | 54.471 |
| Ca_Desi_SNP2068 | [A/G] | *Ca_Desi_LG04* | 54.662 |
| Ca_Desi_SNP2069 | [C/A] | *Ca_Desi_LG04* | 54.709 |
| Ca_Desi_SNP2070 | [T/C] | *Ca_Desi_LG04* | 54.740 |
| Ca_Desi_SNP2071 | [G/A] | *Ca_Desi_LG04* | 54.918 |
| Ca_Desi_SNP2072 | [A/C] | *Ca_Desi_LG04* | 54.957 |
| Ca_Desi_SNP2073 | [G/A] | *Ca_Desi_LG04* | 55.199 |
| Ca_Desi_SNP2074 | [A/G] | *Ca_Desi_LG04* | 55.305 |
| Ca_Desi_SNP2075 | [T/C] | *Ca_Desi_LG04* | 55.511 |
| Ca_Desi_SNP2076 | [T/C] | *Ca_Desi_LG04* | 55.572 |
| Ca_Desi_SNP2077 | [T/C] | *Ca_Desi_LG04* | 55.715 |
| Ca_Desi_SNP2078 | [C/T] | *Ca_Desi_LG04* | 55.735 |
| Ca_Desi_SNP2079 | [T/G] | *Ca_Desi_LG04* | 55.746 |
| Ca_Desi_SNP2080 | [G/A] | *Ca_Desi_LG04* | 55.821 |
| Ca_Desi_SNP2081 | [T/C] | *Ca_Desi_LG04* | 55.830 |
| Ca_Desi_SNP2082 | [T/A] | *Ca_Desi_LG04* | 55.930 |
| Ca_Desi_SNP2083 | [G/T] | *Ca_Desi_LG04* | 56.077 |
| Ca_Desi_SNP2084 | [A/T] | *Ca_Desi_LG04* | 56.093 |
| Ca_Desi_SNP2085 | [T/C] | *Ca_Desi_LG04* | 56.142 |
| Ca_Desi_SNP2086 | [C/T] | *Ca_Desi_LG04* | 56.189 |
| Ca_Desi_SNP2087 | [G/T] | *Ca_Desi_LG04* | 56.269 |
| Ca_Desi_SNP2088 | [G/A] | *Ca_Desi_LG04* | 56.304 |
| Ca_Desi_SNP2089 | [T/C] | *Ca_Desi_LG04* | 56.435 |
| Ca_Desi_SNP2090 | [A/T] | *Ca_Desi_LG04* | 56.442 |
| Ca_Desi_SNP2091 | [A/C] | *Ca_Desi_LG04* | 56.604 |
| Ca_Desi_SNP2092 | [G/A] | *Ca_Desi_LG04* | 56.636 |
| Ca_Desi_SNP2093 | [A/G] | *Ca_Desi_LG04* | 56.844 |
| Ca_Desi_SNP2094 | [A/G] | *Ca_Desi_LG04* | 56.949 |
| Ca_Desi_SNP2095 | [C/A] | *Ca_Desi_LG04* | 57.090 |
| Ca_Desi_SNP2096 | [T/C] | *Ca_Desi_LG04* | 57.146 |
| Ca_Desi_SNP2097 | [G/A] | *Ca_Desi_LG04* | 57.151 |
| Ca_Desi_SNP2098 | [T/C] | *Ca_Desi_LG04* | 57.365 |
| Ca_Desi_SNP2099 | [T/G] | *Ca_Desi_LG04* | 57.464 |
| Ca_Desi_SNP2100 | [G/C] | *Ca_Desi_LG04* | 57.498 |
| Ca_Desi_SNP2101 | [A/G] | *Ca_Desi_LG04* | 57.543 |
| Ca_Desi_SNP2102 | [C/T] | *Ca_Desi_LG04* | 57.561 |
| Ca_Desi_SNP2103 | [C/T] | *Ca_Desi_LG04* | 57.640 |
| Ca_Desi_SNP2104 | [G/A] | *Ca_Desi_LG04* | 57.948 |
| Ca_Desi_SNP2105 | [A/C] | *Ca_Desi_LG04* | 58.166 |
| Ca_Desi_SNP2106 | [C/A] | *Ca_Desi_LG04* | 58.210 |
| Ca_Desi_SNP2107 | [T/G] | *Ca_Desi_LG04* | 58.385 |
| Ca_Desi_SNP2108 | [G/T] | *Ca_Desi_LG04* | 58.519 |
| Ca_Desi_SNP2109 | [G/A] | *Ca_Desi_LG04* | 58.531 |

| **SNP IDs** | **SNPs** | **Linkage Group (LGs)** | **Genetic positions (cM) of mapped SNPs** |
|---|---|---|---|
| Ca_Desi_SNP2110 | [A/C] | *Ca_Desi_LG04* | 58.549 |
| Ca_Desi_SNP2111 | [T/G] | *Ca_Desi_LG04* | 58.697 |
| Ca_Desi_SNP2112 | [A/G] | *Ca_Desi_LG04* | 59.364 |
| Ca_Desi_SNP2113 | [T/G] | *Ca_Desi_LG04* | 59.452 |
| Ca_Desi_SNP2114 | [T/C] | *Ca_Desi_LG04* | 59.616 |
| Ca_Desi_SNP2115 | [T/C] | *Ca_Desi_LG04* | 59.781 |
| Ca_Desi_SNP2116 | [G/T] | *Ca_Desi_LG04* | 59.818 |
| Ca_Desi_SNP2117 | [C/T] | *Ca_Desi_LG04* | 59.853 |
| Ca_Desi_SNP2118 | [T/C] | *Ca_Desi_LG04* | 60.563 |
| Ca_Desi_SNP2119 | [T/G] | *Ca_Desi_LG04* | 60.887 |
| Ca_Desi_SNP2120 | [C/A] | *Ca_Desi_LG04* | 60.901 |
| Ca_Desi_SNP2121 | [T/C] | *Ca_Desi_LG04* | 61.071 |
| Ca_Desi_SNP2122 | [T/G] | *Ca_Desi_LG04* | 61.124 |
| Ca_Desi_SNP2123 | [T/G] | *Ca_Desi_LG04* | 61.131 |
| Ca_Desi_SNP2124 | [A/C] | *Ca_Desi_LG04* | 61.439 |
| Ca_Desi_SNP2125 | [C/T] | *Ca_Desi_LG04* | 61.603 |
| Ca_Desi_SNP2126 | [C/A] | *Ca_Desi_LG04* | 61.706 |
| Ca_Desi_SNP2127 | [A/C] | *Ca_Desi_LG04* | 61.914 |
| Ca_Desi_SNP2128 | [T/G] | *Ca_Desi_LG04* | 62.546 |
| Ca_Desi_SNP2129 | [A/G] | *Ca_Desi_LG04* | 63.085 |
| Ca_Desi_SNP2130 | [C/A] | *Ca_Desi_LG04* | 63.295 |
| Ca_Desi_SNP2131 | [A/G] | *Ca_Desi_LG04* | 63.969 |
| Ca_Desi_SNP2132 | [G/T] | *Ca_Desi_LG04* | 64.063 |
| Ca_Desi_SNP2133 | [C/A] | *Ca_Desi_LG04* | 64.875 |
| Ca_Desi_SNP2134 | [G/T] | *Ca_Desi_LG04* | 64.952 |
| Ca_Desi_SNP2135 | [C/A] | *Ca_Desi_LG04* | 65.233 |
| Ca_Desi_SNP2136 | [A/G] | *Ca_Desi_LG04* | 65.669 |
| Ca_Desi_SNP2137 | [C/T] | *Ca_Desi_LG04* | 65.891 |
| Ca_Desi_SNP2138 | [A/G] | *Ca_Desi_LG04* | 65.893 |
| Ca_Desi_SNP2139 | [G/A] | *Ca_Desi_LG04* | 65.936 |
| Ca_Desi_SNP2140 | [T/A] | *Ca_Desi_LG04* | 66.146 |
| Ca_Desi_SNP2141 | [C/A] | *Ca_Desi_LG04* | 66.152 |
| Ca_Desi_SNP2142 | [C/A] | *Ca_Desi_LG04* | 66.179 |
| Ca_Desi_SNP2143 | [A/G] | *Ca_Desi_LG04* | 66.370 |
| Ca_Desi_SNP2144 | [A/G] | *Ca_Desi_LG04* | 66.488 |
| Ca_Desi_SNP2145 | [G/A] | *Ca_Desi_LG04* | 66.921 |
| Ca_Desi_SNP2146 | [T/C] | *Ca_Desi_LG04* | 67.018 |
| Ca_Desi_SNP2147 | [C/T] | *Ca_Desi_LG04* | 67.063 |
| Ca_Desi_SNP2148 | [T/A] | *Ca_Desi_LG04* | 67.115 |
| Ca_Desi_SNP2149 | [A/T] | *Ca_Desi_LG04* | 67.225 |
| Ca_Desi_SNP2150 | [T/C] | *Ca_Desi_LG04* | 67.227 |
| Ca_Desi_SNP2151 | [A/C] | *Ca_Desi_LG04* | 67.299 |
| Ca_Desi_SNP2152 | [T/C] | *Ca_Desi_LG04* | 67.322 |
| Ca_Desi_SNP2153 | [T/C] | *Ca_Desi_LG04* | 67.487 |
| Ca_Desi_SNP2154 | [A/C] | *Ca_Desi_LG04* | 67.516 |
| Ca_Desi_SNP2155 | [G/A] | *Ca_Desi_LG04* | 67.633 |
| Ca_Desi_SNP2156 | [A/C] | *Ca_Desi_LG04* | 67.670 |
| Ca_Desi_SNP2157 | [G/A] | *Ca_Desi_LG04* | 67.724 |

| **SNP IDs** | **SNPs** | **Linkage Group (LGs)** | **Genetic positions (cM) of mapped SNPs** |
|---|---|---|---|
| Ca_Desi_SNP2158 | [A/G] | *Ca_Desi_LG04* | 67.746 |
| Ca_Desi_SNP2159 | [A/G] | *Ca_Desi_LG04* | 67.944 |
| Ca_Desi_SNP2160 | [T/C] | *Ca_Desi_LG04* | 68.026 |
| Ca_Desi_SNP2161 | [C/G] | *Ca_Desi_LG04* | 68.223 |
| Ca_Desi_SNP2162 | [C/A] | *Ca_Desi_LG04* | 68.352 |
| Ca_Desi_SNP2163 | [C/T] | *Ca_Desi_LG04* | 68.360 |
| Ca_Desi_SNP2164 | [G/C] | *Ca_Desi_LG04* | 68.598 |
| Ca_Desi_SNP2165 | [G/A] | *Ca_Desi_LG04* | 68.788 |
| Ca_Desi_SNP2166 | [T/C] | *Ca_Desi_LG04* | 68.868 |
| Ca_Desi_SNP2167 | [G/T] | *Ca_Desi_LG04* | 68.916 |
| Ca_Desi_SNP2168 | [A/G] | *Ca_Desi_LG04* | 69.187 |
| Ca_Desi_SNP2169 | [C/T] | *Ca_Desi_LG04* | 69.259 |
| Ca_Desi_SNP2170 | [A/T] | *Ca_Desi_LG04* | 69.377 |
| Ca_Desi_SNP2171 | [A/G] | *Ca_Desi_LG04* | 69.411 |
| Ca_Desi_SNP2172 | [A/G] | *Ca_Desi_LG04* | 69.432 |
| Ca_Desi_SNP2173 | [C/T] | *Ca_Desi_LG04* | 69.831 |
| Ca_Desi_SNP2174 | [G/A] | *Ca_Desi_LG04* | 69.855 |
| Ca_Desi_SNP2175 | [A/G] | *Ca_Desi_LG04* | 69.888 |
| Ca_Desi_SNP2176 | [G/A] | *Ca_Desi_LG04* | 70.080 |
| Ca_Desi_SNP2177 | [A/G] | *Ca_Desi_LG04* | 70.169 |
| Ca_Desi_SNP2178 | [G/C] | *Ca_Desi_LG04* | 70.274 |
| Ca_Desi_SNP2179 | [C/T] | *Ca_Desi_LG04* | 70.393 |
| Ca_Desi_SNP2180 | [T/C] | *Ca_Desi_LG04* | 70.443 |
| Ca_Desi_SNP2181 | [C/G] | *Ca_Desi_LG04* | 70.446 |
| Ca_Desi_SNP2182 | [T/C] | *Ca_Desi_LG04* | 70.540 |
| Ca_Desi_SNP2183 | [C/G] | *Ca_Desi_LG04* | 70.899 |
| Ca_Desi_SNP2184 | [A/G] | *Ca_Desi_LG04* | 70.936 |
| Ca_Desi_SNP2185 | [T/G] | *Ca_Desi_LG04* | 71.021 |
| Ca_Desi_SNP2186 | [T/G] | *Ca_Desi_LG04* | 71.147 |
| Ca_Desi_SNP2187 | [A/G] | *Ca_Desi_LG04* | 71.376 |
| Ca_Desi_SNP2188 | [A/G] | *Ca_Desi_LG04* | 71.553 |
| Ca_Desi_SNP2189 | [A/G] | *Ca_Desi_LG04* | 71.569 |
| Ca_Desi_SNP2190 | [C/T] | *Ca_Desi_LG04* | 71.605 |
| Ca_Desi_SNP2191 | [T/C] | *Ca_Desi_LG04* | 71.623 |
| Ca_Desi_SNP2192 | [A/G] | *Ca_Desi_LG04* | 71.707 |
| Ca_Desi_SNP2193 | [A/G] | *Ca_Desi_LG04* | 71.887 |
| Ca_Desi_SNP2194 | [A/G] | *Ca_Desi_LG04* | 71.938 |
| Ca_Desi_SNP2195 | [T/G] | *Ca_Desi_LG04* | 72.160 |
| Ca_Desi_SNP2196 | [T/A] | *Ca_Desi_LG04* | 72.530 |
| Ca_Desi_SNP2197 | [C/T] | *Ca_Desi_LG04* | 72.810 |
| Ca_Desi_SNP2198 | [G/T] | *Ca_Desi_LG04* | 72.861 |
| Ca_Desi_SNP2199 | [C/T] | *Ca_Desi_LG04* | 73.204 |
| Ca_Desi_SNP2200 | [A/G] | *Ca_Desi_LG04* | 73.329 |
| Ca_Desi_SNP2201 | [A/G] | *Ca_Desi_LG04* | 73.373 |
| Ca_Desi_SNP2202 | [T/C] | *Ca_Desi_LG04* | 73.466 |
| Ca_Desi_SNP2203 | [G/A] | *Ca_Desi_LG04* | 73.718 |
| Ca_Desi_SNP2204 | [C/T] | *Ca_Desi_LG04* | 73.868 |
| Ca_Desi_SNP2205 | [T/G] | *Ca_Desi_LG04* | 73.881 |

| **SNP IDs** | **SNPs** | **Linkage Group (LGs)** | **Genetic positions (cM) of mapped SNPs** |
|---|---|---|---|
| Ca_Desi_SNP2206 | [T/A] | *Ca_Desi_LG04* | 73.946 |
| Ca_Desi_SNP2207 | [T/C] | *Ca_Desi_LG04* | 74.358 |
| Ca_Desi_SNP2208 | [C/T] | *Ca_Desi_LG04* | 74.383 |
| Ca_Desi_SNP2209 | [A/G] | *Ca_Desi_LG04* | 74.604 |
| Ca_Desi_SNP2210 | [A/T] | *Ca_Desi_LG04* | 74.653 |
| Ca_Desi_SNP2211 | [G/A] | *Ca_Desi_LG04* | 74.665 |
| Ca_Desi_SNP2212 | [G/A] | *Ca_Desi_LG04* | 74.730 |
| Ca_Desi_SNP2213 | [C/A] | *Ca_Desi_LG04* | 74.840 |
| Ca_Desi_SNP2214 | [C/T] | *Ca_Desi_LG04* | 74.890 |
| Ca_Desi_SNP2215 | [T/G] | *Ca_Desi_LG04* | 74.894 |
| Ca_Desi_SNP2216 | [A/G] | *Ca_Desi_LG04* | 74.932 |
| Ca_Desi_SNP2217 | [G/A] | *Ca_Desi_LG04* | 75.168 |
| Ca_Desi_SNP2218 | [A/C] | *Ca_Desi_LG04* | 75.244 |
| Ca_Desi_SNP2219 | [A/G] | *Ca_Desi_LG04* | 75.470 |
| Ca_Desi_SNP2220 | [C/T] | *Ca_Desi_LG04* | 75.669 |
| Ca_Desi_SNP2221 | [A/G] | *Ca_Desi_LG04* | 75.773 |
| Ca_Desi_SNP2222 | [A/G] | *Ca_Desi_LG04* | 76.537 |
| Ca_Desi_SNP2223 | [C/T] | *Ca_Desi_LG04* | 76.811 |
| Ca_Desi_SNP2224 | [C/T] | *Ca_Desi_LG04* | 76.908 |
| Ca_Desi_SNP2225 | [T/C] | *Ca_Desi_LG04* | 77.017 |
| Ca_Desi_SNP2226 | [A/G] | *Ca_Desi_LG04* | 77.053 |
| Ca_Desi_SNP2227 | [G/A] | *Ca_Desi_LG04* | 77.198 |
| Ca_Desi_SNP2228 | [G/T] | *Ca_Desi_LG04* | 77.211 |
| Ca_Desi_SNP2229 | [C/G] | *Ca_Desi_LG04* | 77.384 |
| Ca_Desi_SNP2230 | [T/C] | *Ca_Desi_LG04* | 77.574 |
| Ca_Desi_SNP2231 | [T/G] | *Ca_Desi_LG04* | 77.829 |
| Ca_Desi_SNP2232 | [G/A] | *Ca_Desi_LG04* | 77.847 |
| Ca_Desi_SNP2233 | [G/T] | *Ca_Desi_LG04* | 77.967 |
| Ca_Desi_SNP2234 | [A/T] | *Ca_Desi_LG04* | 77.980 |
| Ca_Desi_SNP2235 | [T/A] | *Ca_Desi_LG04* | 78.004 |
| Ca_Desi_SNP2236 | [T/C] | *Ca_Desi_LG04* | 78.180 |
| Ca_Desi_SNP2237 | [T/C] | *Ca_Desi_LG04* | 78.225 |
| Ca_Desi_SNP2238 | [A/G] | *Ca_Desi_LG04* | 78.380 |
| Ca_Desi_SNP2239 | [A/G] | *Ca_Desi_LG04* | 78.491 |
| Ca_Desi_SNP2240 | [C/T] | *Ca_Desi_LG04* | 78.547 |
| Ca_Desi_SNP2241 | [C/T] | *Ca_Desi_LG04* | 78.556 |
| Ca_Desi_SNP2242 | [G/A] | *Ca_Desi_LG04* | 78.633 |
| Ca_Desi_SNP2243 | [A/G] | *Ca_Desi_LG04* | 78.754 |
| Ca_Desi_SNP2244 | [G/A] | *Ca_Desi_LG04* | 78.773 |
| Ca_Desi_SNP2245 | [A/C] | *Ca_Desi_LG04* | 78.874 |
| Ca_Desi_SNP2246 | [T/A] | *Ca_Desi_LG04* | 79.006 |
| Ca_Desi_SNP2247 | [G/A] | *Ca_Desi_LG04* | 79.126 |
| Ca_Desi_SNP2248 | [G/T] | *Ca_Desi_LG04* | 79.225 |
| Ca_Desi_SNP2249 | [A/G] | *Ca_Desi_LG04* | 79.332 |
| Ca_Desi_SNP2250 | [T/C] | *Ca_Desi_LG04* | 79.408 |
| Ca_Desi_SNP2251 | [T/C] | *Ca_Desi_LG04* | 79.426 |
| Ca_Desi_SNP2252 | [A/C] | *Ca_Desi_LG04* | 79.852 |
| Ca_Desi_SNP2253 | [G/A] | *Ca_Desi_LG04* | 80.175 |

| **SNP IDs** | **SNPs** | **Linkage Group (LGs)** | **Genetic positions (cM) of mapped SNPs** |
|---|---|---|---|
| Ca_Desi_SNP2254 | [T/G] | *Ca_Desi_LG04* | 80.279 |
| Ca_Desi_SNP2255 | [T/G] | *Ca_Desi_LG04* | 80.313 |
| Ca_Desi_SNP2256 | [G/T] | *Ca_Desi_LG04* | 80.348 |
| Ca_Desi_SNP2257 | [T/G] | *Ca_Desi_LG04* | 80.693 |
| Ca_Desi_SNP2258 | [T/A] | *Ca_Desi_LG04* | 81.107 |
| Ca_Desi_SNP2259 | [A/G] | *Ca_Desi_LG04* | 81.309 |
| Ca_Desi_SNP2260 | [T/G] | *Ca_Desi_LG04* | 81.983 |
| Ca_Desi_SNP2261 | [A/G] | *Ca_Desi_LG04* | 82.093 |
| Ca_Desi_SNP2262 | [C/G] | *Ca_Desi_LG04* | 82.257 |
| Ca_Desi_SNP2263 | [T/C] | *Ca_Desi_LG04* | 82.790 |
| Ca_Desi_SNP2264 | [A/T] | *Ca_Desi_LG04* | 82.810 |
| Ca_Desi_SNP2265 | [G/A] | *Ca_Desi_LG04* | 82.919 |
| Ca_Desi_SNP2266 | [T/G] | *Ca_Desi_LG04* | 83.078 |
| Ca_Desi_SNP2267 | [C/A] | *Ca_Desi_LG04* | 83.977 |
| Ca_Desi_SNP2268 | [T/G] | *Ca_Desi_LG04* | 84.048 |
| Ca_Desi_SNP2269 | [A/C] | *Ca_Desi_LG04* | 84.664 |
| Ca_Desi_SNP2270 | [T/C] | *Ca_Desi_LG04* | 84.721 |
| Ca_Desi_SNP2271 | [T/G] | *Ca_Desi_LG04* | 85.365 |
| Ca_Desi_SNP2272 | [T/G] | *Ca_Desi_LG04* | 85.944 |
| Ca_Desi_SNP2273 | [T/G] | *Ca_Desi_LG04* | 85.949 |
| Ca_Desi_SNP2274 | [A/C] | *Ca_Desi_LG04* | 86.240 |
| Ca_Desi_SNP2275 | [G/C] | *Ca_Desi_LG04* | 86.290 |
| Ca_Desi_SNP2276 | [T/C] | *Ca_Desi_LG04* | 87.627 |
| Ca_Desi_SNP2277 | [A/C] | *Ca_Desi_LG04* | 91.745 |
| Ca_Desi_SNP2278 | [T/C] | *Ca_Desi_LG04* | 94.250 |
| Ca_Desi_SNP2279 | [A/C] | *Ca_Desi_LG04* | 94.548 |
| Ca_Desi_SNP2280 | [A/G] | *Ca_Desi_LG04* | 94.690 |
| Ca_Desi_SNP2281 | [A/G] | *Ca_Desi_LG04* | 95.921 |
| Ca_Desi_SNP2282 | [G/A] | *Ca_Desi_LG04* | 96.031 |
| Ca_Desi_SNP2283 | [A/C] | *Ca_Desi_LG04* | 97.782 |
| Ca_Desi_SNP2284 | [T/G] | *Ca_Desi_LG05* | 0.000 |
| Ca_Desi_SNP2285 | [T/C] | *Ca_Desi_LG05* | 2.005 |
| Ca_Desi_SNP2286 | [T/C] | *Ca_Desi_LG05* | 2.906 |
| Ca_Desi_SNP2287 | [A/G] | *Ca_Desi_LG05* | 6.592 |
| Ca_Desi_SNP2288 | [A/C] | *Ca_Desi_LG05* | 7.367 |
| Ca_Desi_SNP2289 | [A/T] | *Ca_Desi_LG05* | 8.031 |
| Ca_Desi_SNP2290 | [A/G] | *Ca_Desi_LG05* | 8.168 |
| Ca_Desi_SNP2291 | [A/C] | *Ca_Desi_LG05* | 11.729 |
| Ca_Desi_SNP2292 | [T/C] | *Ca_Desi_LG05* | 11.736 |
| Ca_Desi_SNP2293 | [G/A] | *Ca_Desi_LG05* | 11.945 |
| Ca_Desi_SNP2294 | [A/C] | *Ca_Desi_LG05* | 12.308 |
| Ca_Desi_SNP2295 | [T/A] | *Ca_Desi_LG05* | 13.330 |
| Ca_Desi_SNP2296 | [A/C] | *Ca_Desi_LG05* | 14.077 |
| Ca_Desi_SNP2297 | [T/C] | *Ca_Desi_LG05* | 14.410 |
| Ca_Desi_SNP2298 | [A/G] | *Ca_Desi_LG05* | 14.439 |
| Ca_Desi_SNP2299 | [A/T] | *Ca_Desi_LG05* | 14.800 |
| Ca_Desi_SNP2300 | [C/G] | *Ca_Desi_LG05* | 14.947 |
| Ca_Desi_SNP2301 | [T/G] | *Ca_Desi_LG05* | 15.061 |

| **SNP IDs** | **SNPs** | **Linkage Group (LGs)** | **Genetic positions (cM) of mapped SNPs** |
|---|---|---|---|
| Ca_Desi_SNP2302 | [T/C] | *Ca_Desi_LG05* | 15.267 |
| Ca_Desi_SNP2303 | [A/T] | *Ca_Desi_LG05* | 15.634 |
| Ca_Desi_SNP2304 | [T/C] | *Ca_Desi_LG05* | 15.947 |
| Ca_Desi_SNP2305 | [T/A] | *Ca_Desi_LG05* | 15.994 |
| Ca_Desi_SNP2306 | [T/C] | *Ca_Desi_LG05* | 16.773 |
| Ca_Desi_SNP2307 | [C/T] | *Ca_Desi_LG05* | 16.975 |
| Ca_Desi_SNP2308 | [C/T] | *Ca_Desi_LG05* | 17.521 |
| Ca_Desi_SNP2309 | [T/C] | *Ca_Desi_LG05* | 18.122 |
| Ca_Desi_SNP2310 | [T/C] | *Ca_Desi_LG05* | 18.201 |
| Ca_Desi_SNP2311 | [T/C] | *Ca_Desi_LG05* | 18.461 |
| Ca_Desi_SNP2312 | [G/A] | *Ca_Desi_LG05* | 18.515 |
| Ca_Desi_SNP2313 | [C/T] | *Ca_Desi_LG05* | 18.744 |
| Ca_Desi_SNP2314 | [A/G] | *Ca_Desi_LG05* | 18.990 |
| Ca_Desi_SNP2315 | [T/C] | *Ca_Desi_LG05* | 19.374 |
| Ca_Desi_SNP2316 | [A/G] | *Ca_Desi_LG05* | 19.377 |
| Ca_Desi_SNP2317 | [T/G] | *Ca_Desi_LG05* | 20.006 |
| Ca_Desi_SNP2318 | [A/G] | *Ca_Desi_LG05* | 20.039 |
| Ca_Desi_SNP2319 | [C/T] | *Ca_Desi_LG05* | 20.102 |
| Ca_Desi_SNP2320 | [T/C] | *Ca_Desi_LG05* | 20.388 |
| Ca_Desi_SNP2321 | [A/G] | *Ca_Desi_LG05* | 20.476 |
| Ca_Desi_SNP2322 | [C/T] | *Ca_Desi_LG05* | 20.608 |
| Ca_Desi_SNP2323 | [A/G] | *Ca_Desi_LG05* | 20.810 |
| Ca_Desi_SNP2324 | [C/T] | *Ca_Desi_LG05* | 21.302 |
| Ca_Desi_SNP2325 | [A/G] | *Ca_Desi_LG05* | 21.626 |
| Ca_Desi_SNP2326 | [C/T] | *Ca_Desi_LG05* | 21.885 |
| Ca_Desi_SNP2327 | [A/G] | *Ca_Desi_LG05* | 22.069 |
| Ca_Desi_SNP2328 | [A/G] | *Ca_Desi_LG05* | 22.091 |
| Ca_Desi_SNP2329 | [A/C] | *Ca_Desi_LG05* | 22.126 |
| Ca_Desi_SNP2330 | [A/G] | *Ca_Desi_LG05* | 22.612 |
| Ca_Desi_SNP2331 | [C/T] | *Ca_Desi_LG05* | 22.874 |
| Ca_Desi_SNP2332 | [G/T] | *Ca_Desi_LG05* | 23.046 |
| Ca_Desi_SNP2333 | [A/T] | *Ca_Desi_LG05* | 23.367 |
| Ca_Desi_SNP2334 | [T/C] | *Ca_Desi_LG05* | 23.683 |
| Ca_Desi_SNP2335 | [T/G] | *Ca_Desi_LG05* | 23.744 |
| Ca_Desi_SNP2336 | [T/C] | *Ca_Desi_LG05* | 23.792 |
| Ca_Desi_SNP2337 | [T/A] | *Ca_Desi_LG05* | 23.805 |
| Ca_Desi_SNP2338 | [C/G] | *Ca_Desi_LG05* | 24.875 |
| Ca_Desi_SNP2339 | [T/C] | *Ca_Desi_LG05* | 25.012 |
| Ca_Desi_SNP2340 | [A/C] | *Ca_Desi_LG05* | 25.029 |
| Ca_Desi_SNP2341 | [C/T] | *Ca_Desi_LG05* | 25.833 |
| Ca_Desi_SNP2342 | [A/G] | *Ca_Desi_LG05* | 25.834 |
| Ca_Desi_SNP2343 | [A/G] | *Ca_Desi_LG05* | 25.948 |
| Ca_Desi_SNP2344 | [G/A] | *Ca_Desi_LG05* | 26.237 |
| Ca_Desi_SNP2345 | [A/C] | *Ca_Desi_LG05* | 26.679 |
| Ca_Desi_SNP2346 | [G/A] | *Ca_Desi_LG05* | 26.766 |
| Ca_Desi_SNP2347 | [A/G] | *Ca_Desi_LG05* | 26.913 |
| Ca_Desi_SNP2348 | [T/G] | *Ca_Desi_LG05* | 27.507 |
| Ca_Desi_SNP2349 | [A/G] | *Ca_Desi_LG05* | 27.515 |

| **SNP IDs** | **SNPs** | **Linkage Group (LGs)** | **Genetic positions (cM) of mapped SNPs** |
|---|---|---|---|
| Ca_Desi_SNP2350 | [G/A] | *Ca_Desi_LG05* | 27.620 |
| Ca_Desi_SNP2351 | [A/C] | *Ca_Desi_LG05* | 27.755 |
| Ca_Desi_SNP2352 | [G/A] | *Ca_Desi_LG05* | 27.865 |
| Ca_Desi_SNP2353 | [T/G] | *Ca_Desi_LG05* | 28.042 |
| Ca_Desi_SNP2354 | [C/T] | *Ca_Desi_LG05* | 28.115 |
| Ca_Desi_SNP2355 | [A/T] | *Ca_Desi_LG05* | 28.564 |
| Ca_Desi_SNP2356 | [T/G] | *Ca_Desi_LG05* | 28.659 |
| Ca_Desi_SNP2357 | [C/T] | *Ca_Desi_LG05* | 29.214 |
| Ca_Desi_SNP2358 | [A/G] | *Ca_Desi_LG05* | 29.997 |
| Ca_Desi_SNP2359 | [T/A] | *Ca_Desi_LG05* | 30.007 |
| Ca_Desi_SNP2360 | [T/A] | *Ca_Desi_LG05* | 30.039 |
| Ca_Desi_SNP2361 | [G/T] | *Ca_Desi_LG05* | 31.332 |
| Ca_Desi_SNP2362 | [T/C] | *Ca_Desi_LG05* | 31.552 |
| Ca_Desi_SNP2363 | [C/A] | *Ca_Desi_LG05* | 32.027 |
| Ca_Desi_SNP2364 | [T/G] | *Ca_Desi_LG05* | 32.574 |
| Ca_Desi_SNP2365 | [A/T] | *Ca_Desi_LG05* | 33.214 |
| Ca_Desi_SNP2366 | [G/A] | *Ca_Desi_LG05* | 33.498 |
| Ca_Desi_SNP2367 | [A/C] | *Ca_Desi_LG05* | 33.777 |
| Ca_Desi_SNP2368 | [A/G] | *Ca_Desi_LG05* | 33.814 |
| Ca_Desi_SNP2369 | [C/T] | *Ca_Desi_LG05* | 34.433 |
| Ca_Desi_SNP2370 | [C/G] | *Ca_Desi_LG05* | 34.453 |
| Ca_Desi_SNP2371 | [A/C] | *Ca_Desi_LG05* | 34.668 |
| Ca_Desi_SNP2372 | [G/T] | *Ca_Desi_LG05* | 34.704 |
| Ca_Desi_SNP2373 | [T/C] | *Ca_Desi_LG05* | 34.902 |
| Ca_Desi_SNP2374 | [G/T] | *Ca_Desi_LG05* | 34.985 |
| Ca_Desi_SNP2375 | [T/C] | *Ca_Desi_LG05* | 35.008 |
| Ca_Desi_SNP2376 | [G/C] | *Ca_Desi_LG05* | 35.011 |
| Ca_Desi_SNP2377 | [C/G] | *Ca_Desi_LG05* | 35.250 |
| Ca_Desi_SNP2378 | [A/C] | *Ca_Desi_LG05* | 35.404 |
| Ca_Desi_SNP2379 | [C/T] | *Ca_Desi_LG05* | 35.604 |
| Ca_Desi_SNP2380 | [G/A] | *Ca_Desi_LG05* | 35.638 |
| Ca_Desi_SNP2381 | [A/G] | *Ca_Desi_LG05* | 35.649 |
| Ca_Desi_SNP2382 | [G/T] | *Ca_Desi_LG05* | 35.968 |
| Ca_Desi_SNP2383 | [T/C] | *Ca_Desi_LG05* | 36.068 |
| Ca_Desi_SNP2384 | [T/C] | *Ca_Desi_LG05* | 36.106 |
| Ca_Desi_SNP2385 | [C/T] | *Ca_Desi_LG05* | 36.889 |
| Ca_Desi_SNP2386 | [T/G] | *Ca_Desi_LG05* | 36.895 |
| Ca_Desi_SNP2387 | [G/A] | *Ca_Desi_LG05* | 36.943 |
| Ca_Desi_SNP2388 | [A/C] | *Ca_Desi_LG05* | 37.002 |
| Ca_Desi_SNP2389 | [A/C] | *Ca_Desi_LG05* | 37.108 |
| Ca_Desi_SNP2390 | [T/C] | *Ca_Desi_LG05* | 37.189 |
| Ca_Desi_SNP2391 | [C/T] | *Ca_Desi_LG05* | 37.305 |
| Ca_Desi_SNP2392 | [C/T] | *Ca_Desi_LG05* | 37.314 |
| Ca_Desi_SNP2393 | [T/G] | *Ca_Desi_LG05* | 37.445 |
| Ca_Desi_SNP2394 | [A/G] | *Ca_Desi_LG05* | 37.901 |
| Ca_Desi_SNP2395 | [T/A] | *Ca_Desi_LG05* | 37.996 |
| Ca_Desi_SNP2396 | [C/A] | *Ca_Desi_LG05* | 38.152 |
| Ca_Desi_SNP2397 | [T/G] | *Ca_Desi_LG05* | 38.257 |

| **SNP IDs** | **SNPs** | **Linkage Group (LGs)** | **Genetic positions (cM) of mapped SNPs** |
|---|---|---|---|
| Ca_Desi_SNP2398 | [C/G] | *Ca_Desi_LG05* | 38.350 |
| Ca_Desi_SNP2399 | [T/G] | *Ca_Desi_LG05* | 38.527 |
| Ca_Desi_SNP2400 | [G/A] | *Ca_Desi_LG05* | 39.007 |
| Ca_Desi_SNP2401 | [T/C] | *Ca_Desi_LG05* | 39.380 |
| Ca_Desi_SNP2402 | [A/G] | *Ca_Desi_LG05* | 39.395 |
| Ca_Desi_SNP2403 | [T/G] | *Ca_Desi_LG05* | 39.652 |
| Ca_Desi_SNP2404 | [T/C] | *Ca_Desi_LG05* | 39.691 |
| Ca_Desi_SNP2405 | [C/T] | *Ca_Desi_LG05* | 39.882 |
| Ca_Desi_SNP2406 | [T/G] | *Ca_Desi_LG05* | 40.430 |
| Ca_Desi_SNP2407 | [T/C] | *Ca_Desi_LG05* | 40.572 |
| Ca_Desi_SNP2408 | [G/T] | *Ca_Desi_LG05* | 40.625 |
| Ca_Desi_SNP2409 | [A/C] | *Ca_Desi_LG05* | 40.937 |
| Ca_Desi_SNP2410 | [T/C] | *Ca_Desi_LG05* | 40.983 |
| Ca_Desi_SNP2411 | [G/C] | *Ca_Desi_LG05* | 41.508 |
| Ca_Desi_SNP2412 | [G/T] | *Ca_Desi_LG05* | 41.640 |
| Ca_Desi_SNP2413 | [A/C] | *Ca_Desi_LG05* | 41.930 |
| Ca_Desi_SNP2414 | [A/C] | *Ca_Desi_LG05* | 41.941 |
| Ca_Desi_SNP2415 | [G/A] | *Ca_Desi_LG05* | 41.971 |
| Ca_Desi_SNP2416 | [T/G] | *Ca_Desi_LG05* | 42.064 |
| Ca_Desi_SNP2417 | [C/A] | *Ca_Desi_LG05* | 42.238 |
| Ca_Desi_SNP2418 | [T/G] | *Ca_Desi_LG05* | 42.700 |
| Ca_Desi_SNP2419 | [G/A] | *Ca_Desi_LG05* | 43.661 |
| Ca_Desi_SNP2420 | [G/A] | *Ca_Desi_LG05* | 43.836 |
| Ca_Desi_SNP2421 | [A/C] | *Ca_Desi_LG05* | 43.993 |
| Ca_Desi_SNP2422 | [G/T] | *Ca_Desi_LG05* | 44.208 |
| Ca_Desi_SNP2423 | [G/T] | *Ca_Desi_LG05* | 44.474 |
| Ca_Desi_SNP2424 | [A/T] | *Ca_Desi_LG05* | 45.017 |
| Ca_Desi_SNP2425 | [T/G] | *Ca_Desi_LG05* | 45.090 |
| Ca_Desi_SNP2426 | [T/C] | *Ca_Desi_LG05* | 45.127 |
| Ca_Desi_SNP2427 | [T/C] | *Ca_Desi_LG05* | 45.579 |
| Ca_Desi_SNP2428 | [A/G] | *Ca_Desi_LG05* | 46.096 |
| Ca_Desi_SNP2429 | [C/T] | *Ca_Desi_LG05* | 46.430 |
| Ca_Desi_SNP2430 | [C/A] | *Ca_Desi_LG05* | 46.470 |
| Ca_Desi_SNP2431 | [C/T] | *Ca_Desi_LG05* | 46.514 |
| Ca_Desi_SNP2432 | [C/A] | *Ca_Desi_LG05* | 46.522 |
| Ca_Desi_SNP2433 | [A/G] | *Ca_Desi_LG05* | 46.535 |
| Ca_Desi_SNP2434 | [T/C] | *Ca_Desi_LG05* | 46.614 |
| Ca_Desi_SNP2435 | [C/T] | *Ca_Desi_LG05* | 46.789 |
| Ca_Desi_SNP2436 | [A/C] | *Ca_Desi_LG05* | 46.977 |
| Ca_Desi_SNP2437 | [C/A] | *Ca_Desi_LG05* | 46.991 |
| Ca_Desi_SNP2438 | [A/C] | *Ca_Desi_LG05* | 47.050 |
| Ca_Desi_SNP2439 | [T/G] | *Ca_Desi_LG05* | 47.203 |
| Ca_Desi_SNP2440 | [A/T] | *Ca_Desi_LG05* | 47.589 |
| Ca_Desi_SNP2441 | [A/G] | *Ca_Desi_LG05* | 47.792 |
| Ca_Desi_SNP2442 | [A/G] | *Ca_Desi_LG05* | 47.806 |
| Ca_Desi_SNP2443 | [A/G] | *Ca_Desi_LG05* | 48.249 |
| Ca_Desi_SNP2444 | [G/A] | *Ca_Desi_LG05* | 48.347 |
| Ca_Desi_SNP2445 | [T/A] | *Ca_Desi_LG05* | 48.659 |

| SNP IDs | SNPs | Linkage Group (LGs) | Genetic positions (cM) of mapped SNPs |
|---|---|---|---|
| Ca_Desi_SNP2446 | [C/T] | *Ca_Desi_LG05* | 48.732 |
| Ca_Desi_SNP2447 | [G/A] | *Ca_Desi_LG05* | 48.949 |
| Ca_Desi_SNP2448 | [C/A] | *Ca_Desi_LG05* | 49.043 |
| Ca_Desi_SNP2449 | [C/A] | *Ca_Desi_LG05* | 49.632 |
| Ca_Desi_SNP2450 | [A/G] | *Ca_Desi_LG05* | 50.011 |
| Ca_Desi_SNP2451 | [T/C] | *Ca_Desi_LG05* | 50.109 |
| Ca_Desi_SNP2452 | [A/G] | *Ca_Desi_LG05* | 50.131 |
| Ca_Desi_SNP2453 | [A/C] | *Ca_Desi_LG05* | 50.337 |
| Ca_Desi_SNP2454 | [A/G] | *Ca_Desi_LG05* | 50.490 |
| Ca_Desi_SNP2455 | [C/T] | *Ca_Desi_LG05* | 50.635 |
| Ca_Desi_SNP2456 | [C/A] | *Ca_Desi_LG05* | 50.996 |
| Ca_Desi_SNP2457 | [G/T] | *Ca_Desi_LG05* | 51.409 |
| Ca_Desi_SNP2458 | [A/G] | *Ca_Desi_LG05* | 51.509 |
| Ca_Desi_SNP2459 | [A/G] | *Ca_Desi_LG05* | 51.841 |
| Ca_Desi_SNP2460 | [A/C] | *Ca_Desi_LG05* | 52.125 |
| Ca_Desi_SNP2461 | [T/G] | *Ca_Desi_LG05* | 52.239 |
| Ca_Desi_SNP2462 | [A/C] | *Ca_Desi_LG05* | 52.288 |
| Ca_Desi_SNP2463 | [C/A] | *Ca_Desi_LG05* | 52.417 |
| Ca_Desi_SNP2464 | [A/G] | *Ca_Desi_LG05* | 52.520 |
| Ca_Desi_SNP2465 | [T/A] | *Ca_Desi_LG05* | 52.987 |
| Ca_Desi_SNP2466 | [A/G] | *Ca_Desi_LG05* | 53.113 |
| Ca_Desi_SNP2467 | [A/G] | *Ca_Desi_LG05* | 53.122 |
| Ca_Desi_SNP2468 | [A/G] | *Ca_Desi_LG05* | 53.139 |
| Ca_Desi_SNP2469 | [G/A] | *Ca_Desi_LG05* | 53.336 |
| Ca_Desi_SNP2470 | [T/G] | *Ca_Desi_LG05* | 53.431 |
| Ca_Desi_SNP2471 | [C/T] | *Ca_Desi_LG05* | 53.468 |
| Ca_Desi_SNP2472 | [G/T] | *Ca_Desi_LG05* | 53.566 |
| Ca_Desi_SNP2473 | [A/C] | *Ca_Desi_LG05* | 53.576 |
| Ca_Desi_SNP2474 | [A/C] | *Ca_Desi_LG05* | 54.164 |
| Ca_Desi_SNP2475 | [A/G] | *Ca_Desi_LG05* | 55.078 |
| Ca_Desi_SNP2476 | [A/C] | *Ca_Desi_LG05* | 55.348 |
| Ca_Desi_SNP2477 | [A/G] | *Ca_Desi_LG05* | 55.537 |
| Ca_Desi_SNP2478 | [C/G] | *Ca_Desi_LG05* | 55.708 |
| Ca_Desi_SNP2479 | [G/A] | *Ca_Desi_LG05* | 55.940 |
| Ca_Desi_SNP2480 | [A/T] | *Ca_Desi_LG05* | 56.037 |
| Ca_Desi_SNP2481 | [A/C] | *Ca_Desi_LG05* | 56.450 |
| Ca_Desi_SNP2482 | [A/C] | *Ca_Desi_LG05* | 56.754 |
| Ca_Desi_SNP2483 | [T/C] | *Ca_Desi_LG05* | 56.862 |
| Ca_Desi_SNP2484 | [A/G] | *Ca_Desi_LG05* | 57.089 |
| Ca_Desi_SNP2485 | [A/G] | *Ca_Desi_LG05* | 57.220 |
| Ca_Desi_SNP2486 | [T/G] | *Ca_Desi_LG05* | 57.359 |
| Ca_Desi_SNP2487 | [A/G] | *Ca_Desi_LG05* | 57.598 |
| Ca_Desi_SNP2488 | [T/C] | *Ca_Desi_LG05* | 57.907 |
| Ca_Desi_SNP2489 | [T/A] | *Ca_Desi_LG05* | 57.987 |
| Ca_Desi_SNP2490 | [C/G] | *Ca_Desi_LG05* | 58.056 |
| Ca_Desi_SNP2491 | [G/T] | *Ca_Desi_LG05* | 58.138 |
| Ca_Desi_SNP2492 | [T/C] | *Ca_Desi_LG05* | 58.349 |
| Ca_Desi_SNP2493 | [T/C] | *Ca_Desi_LG05* | 58.382 |

| **SNP IDs** | **SNPs** | **Linkage Group (LGs)** | **Genetic positions (cM) of mapped SNPs** |
|---|---|---|---|
| Ca_Desi_SNP2494 | [A/G] | *Ca_Desi_LG05* | 58.522 |
| Ca_Desi_SNP2495 | [T/A] | *Ca_Desi_LG05* | 58.542 |
| Ca_Desi_SNP2496 | [A/C] | *Ca_Desi_LG05* | 58.639 |
| Ca_Desi_SNP2497 | [T/G] | *Ca_Desi_LG05* | 58.666 |
| Ca_Desi_SNP2498 | [C/T] | *Ca_Desi_LG05* | 58.877 |
| Ca_Desi_SNP2499 | [C/T] | *Ca_Desi_LG05* | 58.958 |
| Ca_Desi_SNP2500 | [A/G] | *Ca_Desi_LG05* | 59.080 |
| Ca_Desi_SNP2501 | [T/G] | *Ca_Desi_LG05* | 59.477 |
| Ca_Desi_SNP2502 | [A/G] | *Ca_Desi_LG05* | 60.009 |
| Ca_Desi_SNP2503 | [G/A] | *Ca_Desi_LG05* | 60.327 |
| Ca_Desi_SNP2504 | [A/T] | *Ca_Desi_LG05* | 60.379 |
| Ca_Desi_SNP2505 | [A/T] | *Ca_Desi_LG05* | 61.296 |
| Ca_Desi_SNP2506 | [A/G] | *Ca_Desi_LG05* | 61.498 |
| Ca_Desi_SNP2507 | [A/C] | *Ca_Desi_LG05* | 61.524 |
| Ca_Desi_SNP2508 | [G/A] | *Ca_Desi_LG05* | 62.042 |
| Ca_Desi_SNP2509 | [T/A] | *Ca_Desi_LG05* | 62.477 |
| Ca_Desi_SNP2510 | [T/G] | *Ca_Desi_LG05* | 62.621 |
| Ca_Desi_SNP2511 | [A/G] | *Ca_Desi_LG05* | 62.871 |
| Ca_Desi_SNP2512 | [G/A] | *Ca_Desi_LG05* | 62.970 |
| Ca_Desi_SNP2513 | [G/T] | *Ca_Desi_LG05* | 63.093 |
| Ca_Desi_SNP2514 | [A/G] | *Ca_Desi_LG05* | 63.503 |
| Ca_Desi_SNP2515 | [T/C] | *Ca_Desi_LG05* | 63.914 |
| Ca_Desi_SNP2516 | [T/C] | *Ca_Desi_LG05* | 64.778 |
| Ca_Desi_SNP2517 | [G/T] | *Ca_Desi_LG05* | 64.950 |
| Ca_Desi_SNP2518 | [T/G] | *Ca_Desi_LG05* | 65.084 |
| Ca_Desi_SNP2519 | [A/C] | *Ca_Desi_LG05* | 65.211 |
| Ca_Desi_SNP2520 | [T/C] | *Ca_Desi_LG05* | 65.704 |
| Ca_Desi_SNP2521 | [T/C] | *Ca_Desi_LG05* | 66.210 |
| Ca_Desi_SNP2522 | [T/C] | *Ca_Desi_LG05* | 66.340 |
| Ca_Desi_SNP2523 | [T/C] | *Ca_Desi_LG05* | 67.118 |
| Ca_Desi_SNP2524 | [A/G] | *Ca_Desi_LG05* | 67.128 |
| Ca_Desi_SNP2525 | [G/A] | *Ca_Desi_LG05* | 67.397 |
| Ca_Desi_SNP2526 | [T/C] | *Ca_Desi_LG05* | 67.550 |
| Ca_Desi_SNP2527 | [T/C] | *Ca_Desi_LG05* | 67.909 |
| Ca_Desi_SNP2528 | [A/C] | *Ca_Desi_LG05* | 68.591 |
| Ca_Desi_SNP2529 | [T/G] | *Ca_Desi_LG05* | 68.821 |
| Ca_Desi_SNP2530 | [T/G] | *Ca_Desi_LG05* | 68.973 |
| Ca_Desi_SNP2531 | [T/C] | *Ca_Desi_LG05* | 69.183 |
| Ca_Desi_SNP2532 | [A/C] | *Ca_Desi_LG05* | 69.732 |
| Ca_Desi_SNP2533 | [A/G] | *Ca_Desi_LG05* | 70.021 |
| Ca_Desi_SNP2534 | [A/C] | *Ca_Desi_LG05* | 70.210 |
| Ca_Desi_SNP2535 | [G/T] | *Ca_Desi_LG05* | 70.290 |
| Ca_Desi_SNP2536 | [T/G] | *Ca_Desi_LG05* | 70.500 |
| Ca_Desi_SNP2537 | [C/T] | *Ca_Desi_LG05* | 70.547 |
| Ca_Desi_SNP2538 | [T/G] | *Ca_Desi_LG05* | 70.600 |
| Ca_Desi_SNP2539 | [C/T] | *Ca_Desi_LG05* | 70.612 |
| Ca_Desi_SNP2540 | [C/T] | *Ca_Desi_LG05* | 70.696 |
| Ca_Desi_SNP2541 | [A/G] | *Ca_Desi_LG05* | 71.736 |

| **SNP IDs** | **SNPs** | **Linkage Group (LGs)** | **Genetic positions (cM) of mapped SNPs** |
|---|---|---|---|
| Ca_Desi_SNP2542 | [A/C] | *Ca_Desi_LG05* | 72.170 |
| Ca_Desi_SNP2543 | [T/G] | *Ca_Desi_LG05* | 72.439 |
| Ca_Desi_SNP2544 | [G/C] | *Ca_Desi_LG05* | 72.464 |
| Ca_Desi_SNP2545 | [G/A] | *Ca_Desi_LG05* | 72.629 |
| Ca_Desi_SNP2546 | [A/G] | *Ca_Desi_LG05* | 73.403 |
| Ca_Desi_SNP2547 | [A/G] | *Ca_Desi_LG05* | 73.479 |
| Ca_Desi_SNP2548 | [A/C] | *Ca_Desi_LG05* | 73.532 |
| Ca_Desi_SNP2549 | [C/T] | *Ca_Desi_LG05* | 74.330 |
| Ca_Desi_SNP2550 | [A/G] | *Ca_Desi_LG05* | 74.490 |
| Ca_Desi_SNP2551 | [T/C] | *Ca_Desi_LG05* | 74.829 |
| Ca_Desi_SNP2552 | [A/C] | *Ca_Desi_LG05* | 74.934 |
| Ca_Desi_SNP2553 | [A/C] | *Ca_Desi_LG05* | 75.585 |
| Ca_Desi_SNP2554 | [T/C] | *Ca_Desi_LG05* | 75.857 |
| Ca_Desi_SNP2555 | [A/C] | *Ca_Desi_LG05* | 76.213 |
| Ca_Desi_SNP2556 | [A/T] | *Ca_Desi_LG05* | 76.770 |
| Ca_Desi_SNP2557 | [G/C] | *Ca_Desi_LG05* | 76.802 |
| Ca_Desi_SNP2558 | [T/A] | *Ca_Desi_LG05* | 77.003 |
| Ca_Desi_SNP2559 | [C/A] | *Ca_Desi_LG05* | 77.188 |
| Ca_Desi_SNP2560 | [T/C] | *Ca_Desi_LG05* | 77.195 |
| Ca_Desi_SNP2561 | [T/C] | *Ca_Desi_LG05* | 77.911 |
| Ca_Desi_SNP2562 | [T/C] | *Ca_Desi_LG05* | 78.024 |
| Ca_Desi_SNP2563 | [G/A] | *Ca_Desi_LG05* | 78.207 |
| Ca_Desi_SNP2564 | [G/A] | *Ca_Desi_LG05* | 78.250 |
| Ca_Desi_SNP2565 | [T/C] | *Ca_Desi_LG05* | 79.720 |
| Ca_Desi_SNP2566 | [C/T] | *Ca_Desi_LG05* | 79.861 |
| Ca_Desi_SNP2567 | [C/G] | *Ca_Desi_LG05* | 82.027 |
| Ca_Desi_SNP2568 | [A/T] | *Ca_Desi_LG05* | 83.557 |
| Ca_Desi_SNP2569 | [A/C] | *Ca_Desi_LG05* | 84.955 |
| Ca_Desi_SNP2570 | [C/T] | *Ca_Desi_LG05* | 84.994 |
| Ca_Desi_SNP2571 | [T/C] | *Ca_Desi_LG05* | 85.313 |
| Ca_Desi_SNP2572 | [G/T] | *Ca_Desi_LG05* | 86.979 |
| Ca_Desi_SNP2573 | [G/A] | *Ca_Desi_LG05* | 89.402 |
| Ca_Desi_SNP2574 | [T/G] | *Ca_Desi_LG05* | 94.059 |
| Ca_Desi_SNP2575 | [A/C] | *Ca_Desi_LG05* | 94.069 |
| Ca_Desi_SNP2576 | [C/A] | *Ca_Desi_LG06* | 0.000 |
| Ca_Desi_SNP2577 | [A/G] | *Ca_Desi_LG06* | 0.105 |
| Ca_Desi_SNP2578 | [C/A] | *Ca_Desi_LG06* | 0.122 |
| Ca_Desi_SNP2579 | [C/G] | *Ca_Desi_LG06* | 0.565 |
| Ca_Desi_SNP2580 | [A/G] | *Ca_Desi_LG06* | 0.745 |
| Ca_Desi_SNP2581 | [A/G] | *Ca_Desi_LG06* | 3.184 |
| Ca_Desi_SNP2582 | [C/A] | *Ca_Desi_LG06* | 3.440 |
| Ca_Desi_SNP2583 | [T/G] | *Ca_Desi_LG06* | 4.550 |
| Ca_Desi_SNP2584 | [T/G] | *Ca_Desi_LG06* | 4.790 |
| Ca_Desi_SNP2585 | [A/G] | *Ca_Desi_LG06* | 5.066 |
| Ca_Desi_SNP2586 | [C/G] | *Ca_Desi_LG06* | 5.078 |
| Ca_Desi_SNP2587 | [C/T] | *Ca_Desi_LG06* | 5.448 |
| Ca_Desi_SNP2588 | [A/G] | *Ca_Desi_LG06* | 6.020 |
| Ca_Desi_SNP2589 | [A/C] | *Ca_Desi_LG06* | 6.572 |

| **SNP IDs** | **SNPs** | **Linkage Group (LGs)** | **Genetic positions (cM) of mapped SNPs** |
|---|---|---|---|
| Ca_Desi_SNP2590 | [T/C] | *Ca_Desi_LG06* | 6.673 |
| Ca_Desi_SNP2591 | [A/G] | *Ca_Desi_LG06* | 6.868 |
| Ca_Desi_SNP2592 | [C/A] | *Ca_Desi_LG06* | 7.149 |
| Ca_Desi_SNP2593 | [G/T] | *Ca_Desi_LG06* | 7.595 |
| Ca_Desi_SNP2594 | [T/C] | *Ca_Desi_LG06* | 8.146 |
| Ca_Desi_SNP2595 | [A/G] | *Ca_Desi_LG06* | 8.328 |
| Ca_Desi_SNP2596 | [C/T] | *Ca_Desi_LG06* | 8.995 |
| Ca_Desi_SNP2597 | [A/G] | *Ca_Desi_LG06* | 9.261 |
| Ca_Desi_SNP2598 | [G/A] | *Ca_Desi_LG06* | 9.373 |
| Ca_Desi_SNP2599 | [C/A] | *Ca_Desi_LG06* | 9.581 |
| Ca_Desi_SNP2600 | [T/G] | *Ca_Desi_LG06* | 9.637 |
| Ca_Desi_SNP2601 | [G/T] | *Ca_Desi_LG06* | 9.735 |
| Ca_Desi_SNP2602 | [T/C] | *Ca_Desi_LG06* | 10.228 |
| Ca_Desi_SNP2603 | [T/C] | *Ca_Desi_LG06* | 10.295 |
| Ca_Desi_SNP2604 | [A/G] | *Ca_Desi_LG06* | 10.389 |
| Ca_Desi_SNP2605 | [A/C] | *Ca_Desi_LG06* | 10.414 |
| Ca_Desi_SNP2606 | [T/G] | *Ca_Desi_LG06* | 10.777 |
| Ca_Desi_SNP2607 | [T/A] | *Ca_Desi_LG06* | 10.924 |
| Ca_Desi_SNP2608 | [T/G] | *Ca_Desi_LG06* | 10.981 |
| Ca_Desi_SNP2609 | [G/A] | *Ca_Desi_LG06* | 11.505 |
| Ca_Desi_SNP2610 | [G/A] | *Ca_Desi_LG06* | 11.552 |
| Ca_Desi_SNP2611 | [A/C] | *Ca_Desi_LG06* | 12.101 |
| Ca_Desi_SNP2612 | [G/A] | *Ca_Desi_LG06* | 12.721 |
| Ca_Desi_SNP2613 | [T/C] | *Ca_Desi_LG06* | 12.767 |
| Ca_Desi_SNP2614 | [G/T] | *Ca_Desi_LG06* | 12.823 |
| Ca_Desi_SNP2615 | [A/G] | *Ca_Desi_LG06* | 12.966 |
| Ca_Desi_SNP2616 | [C/T] | *Ca_Desi_LG06* | 13.102 |
| Ca_Desi_SNP2617 | [C/T] | *Ca_Desi_LG06* | 13.145 |
| Ca_Desi_SNP2618 | [T/G] | *Ca_Desi_LG06* | 13.808 |
| Ca_Desi_SNP2619 | [A/G] | *Ca_Desi_LG06* | 14.051 |
| Ca_Desi_SNP2620 | [G/A] | *Ca_Desi_LG06* | 14.080 |
| Ca_Desi_SNP2621 | [A/C] | *Ca_Desi_LG06* | 14.088 |
| Ca_Desi_SNP2622 | [T/A] | *Ca_Desi_LG06* | 14.108 |
| Ca_Desi_SNP2623 | [A/C] | *Ca_Desi_LG06* | 14.222 |
| Ca_Desi_SNP2624 | [T/G] | *Ca_Desi_LG06* | 14.772 |
| Ca_Desi_SNP2625 | [A/G] | *Ca_Desi_LG06* | 14.983 |
| Ca_Desi_SNP2626 | [C/G] | *Ca_Desi_LG06* | 15.077 |
| Ca_Desi_SNP2627 | [A/G] | *Ca_Desi_LG06* | 15.173 |
| Ca_Desi_SNP2628 | [A/T] | *Ca_Desi_LG06* | 15.677 |
| Ca_Desi_SNP2629 | [A/G] | *Ca_Desi_LG06* | 15.762 |
| Ca_Desi_SNP2630 | [T/G] | *Ca_Desi_LG06* | 15.892 |
| Ca_Desi_SNP2631 | [T/G] | *Ca_Desi_LG06* | 16.022 |
| Ca_Desi_SNP2632 | [A/G] | *Ca_Desi_LG06* | 16.215 |
| Ca_Desi_SNP2633 | [T/C] | *Ca_Desi_LG06* | 16.423 |
| Ca_Desi_SNP2634 | [A/G] | *Ca_Desi_LG06* | 16.446 |
| Ca_Desi_SNP2635 | [A/G] | *Ca_Desi_LG06* | 16.515 |
| Ca_Desi_SNP2636 | [C/T] | *Ca_Desi_LG06* | 16.698 |
| Ca_Desi_SNP2637 | [T/A] | *Ca_Desi_LG06* | 16.735 |

| **SNP IDs** | **SNPs** | **Linkage Group (LGs)** | **Genetic positions (cM) of mapped SNPs** |
|---|---|---|---|
| Ca_Desi_SNP2638 | [G/A] | *Ca_Desi_LG06* | 16.891 |
| Ca_Desi_SNP2639 | [A/T] | *Ca_Desi_LG06* | 16.974 |
| Ca_Desi_SNP2640 | [T/C] | *Ca_Desi_LG06* | 16.987 |
| Ca_Desi_SNP2641 | [T/G] | *Ca_Desi_LG06* | 17.034 |
| Ca_Desi_SNP2642 | [A/T] | *Ca_Desi_LG06* | 17.263 |
| Ca_Desi_SNP2643 | [A/G] | *Ca_Desi_LG06* | 17.679 |
| Ca_Desi_SNP2644 | [C/A] | *Ca_Desi_LG06* | 18.083 |
| Ca_Desi_SNP2645 | [C/T] | *Ca_Desi_LG06* | 18.170 |
| Ca_Desi_SNP2646 | [C/A] | *Ca_Desi_LG06* | 18.197 |
| Ca_Desi_SNP2647 | [T/C] | *Ca_Desi_LG06* | 18.372 |
| Ca_Desi_SNP2648 | [A/G] | *Ca_Desi_LG06* | 18.788 |
| Ca_Desi_SNP2649 | [A/C] | *Ca_Desi_LG06* | 18.808 |
| Ca_Desi_SNP2650 | [T/C] | *Ca_Desi_LG06* | 18.812 |
| Ca_Desi_SNP2651 | [C/T] | *Ca_Desi_LG06* | 18.818 |
| Ca_Desi_SNP2652 | [G/A] | *Ca_Desi_LG06* | 18.822 |
| Ca_Desi_SNP2653 | [C/T] | *Ca_Desi_LG06* | 18.838 |
| Ca_Desi_SNP2654 | [A/G] | *Ca_Desi_LG06* | 19.128 |
| Ca_Desi_SNP2655 | [A/C] | *Ca_Desi_LG06* | 19.290 |
| Ca_Desi_SNP2656 | [C/T] | *Ca_Desi_LG06* | 19.483 |
| Ca_Desi_SNP2657 | [A/C] | *Ca_Desi_LG06* | 19.873 |
| Ca_Desi_SNP2658 | [C/G] | *Ca_Desi_LG06* | 19.941 |
| Ca_Desi_SNP2659 | [T/G] | *Ca_Desi_LG06* | 20.284 |
| Ca_Desi_SNP2660 | [T/C] | *Ca_Desi_LG06* | 20.435 |
| Ca_Desi_SNP2661 | [A/T] | *Ca_Desi_LG06* | 20.481 |
| Ca_Desi_SNP2662 | [C/A] | *Ca_Desi_LG06* | 20.848 |
| Ca_Desi_SNP2663 | [A/G] | *Ca_Desi_LG06* | 21.060 |
| Ca_Desi_SNP2664 | [A/G] | *Ca_Desi_LG06* | 21.227 |
| Ca_Desi_SNP2665 | [A/G] | *Ca_Desi_LG06* | 21.265 |
| Ca_Desi_SNP2666 | [G/T] | *Ca_Desi_LG06* | 21.309 |
| Ca_Desi_SNP2667 | [G/C] | *Ca_Desi_LG06* | 21.373 |
| Ca_Desi_SNP2668 | [A/C] | *Ca_Desi_LG06* | 21.409 |
| Ca_Desi_SNP2669 | [T/G] | *Ca_Desi_LG06* | 21.547 |
| Ca_Desi_SNP2670 | [T/C] | *Ca_Desi_LG06* | 21.913 |
| Ca_Desi_SNP2671 | [T/G] | *Ca_Desi_LG06* | 22.052 |
| Ca_Desi_SNP2672 | [T/A] | *Ca_Desi_LG06* | 22.242 |
| Ca_Desi_SNP2673 | [A/G] | *Ca_Desi_LG06* | 22.246 |
| Ca_Desi_SNP2674 | [A/C] | *Ca_Desi_LG06* | 22.421 |
| Ca_Desi_SNP2675 | [C/T] | *Ca_Desi_LG06* | 22.486 |
| Ca_Desi_SNP2676 | [T/A] | *Ca_Desi_LG06* | 22.672 |
| Ca_Desi_SNP2677 | [T/A] | *Ca_Desi_LG06* | 23.419 |
| Ca_Desi_SNP2678 | [A/G] | *Ca_Desi_LG06* | 23.454 |
| Ca_Desi_SNP2679 | [A/C] | *Ca_Desi_LG06* | 23.931 |
| Ca_Desi_SNP2680 | [T/A] | *Ca_Desi_LG06* | 24.029 |
| Ca_Desi_SNP2681 | [C/A] | *Ca_Desi_LG06* | 24.040 |
| Ca_Desi_SNP2682 | [T/G] | *Ca_Desi_LG06* | 24.259 |
| Ca_Desi_SNP2683 | [T/G] | *Ca_Desi_LG06* | 24.392 |
| Ca_Desi_SNP2684 | [T/G] | *Ca_Desi_LG06* | 24.410 |
| Ca_Desi_SNP2685 | [T/G] | *Ca_Desi_LG06* | 24.419 |

| **SNP IDs** | **SNPs** | **Linkage Group (LGs)** | **Genetic positions (cM) of mapped SNPs** |
|---|---|---|---|
| Ca_Desi_SNP2686 | [T/C] | *Ca_Desi_LG06* | 24.585 |
| Ca_Desi_SNP2687 | [A/G] | *Ca_Desi_LG06* | 24.661 |
| Ca_Desi_SNP2688 | [C/T] | *Ca_Desi_LG06* | 24.683 |
| Ca_Desi_SNP2689 | [A/C] | *Ca_Desi_LG06* | 24.697 |
| Ca_Desi_SNP2690 | [C/T] | *Ca_Desi_LG06* | 24.809 |
| Ca_Desi_SNP2691 | [G/T] | *Ca_Desi_LG06* | 25.258 |
| Ca_Desi_SNP2692 | [T/A] | *Ca_Desi_LG06* | 25.388 |
| Ca_Desi_SNP2693 | [T/C] | *Ca_Desi_LG06* | 25.403 |
| Ca_Desi_SNP2694 | [A/C] | *Ca_Desi_LG06* | 25.616 |
| Ca_Desi_SNP2695 | [A/C] | *Ca_Desi_LG06* | 25.677 |
| Ca_Desi_SNP2696 | [C/T] | *Ca_Desi_LG06* | 25.767 |
| Ca_Desi_SNP2697 | [C/T] | *Ca_Desi_LG06* | 25.799 |
| Ca_Desi_SNP2698 | [G/C] | *Ca_Desi_LG06* | 25.853 |
| Ca_Desi_SNP2699 | [T/C] | *Ca_Desi_LG06* | 25.953 |
| Ca_Desi_SNP2700 | [A/G] | *Ca_Desi_LG06* | 26.189 |
| Ca_Desi_SNP2701 | [A/G] | *Ca_Desi_LG06* | 26.211 |
| Ca_Desi_SNP2702 | [T/G] | *Ca_Desi_LG06* | 26.239 |
| Ca_Desi_SNP2703 | [C/T] | *Ca_Desi_LG06* | 26.285 |
| Ca_Desi_SNP2704 | [C/T] | *Ca_Desi_LG06* | 26.383 |
| Ca_Desi_SNP2705 | [G/A] | *Ca_Desi_LG06* | 26.630 |
| Ca_Desi_SNP2706 | [A/G] | *Ca_Desi_LG06* | 26.807 |
| Ca_Desi_SNP2707 | [T/G] | *Ca_Desi_LG06* | 26.880 |
| Ca_Desi_SNP2708 | [T/G] | *Ca_Desi_LG06* | 26.954 |
| Ca_Desi_SNP2709 | [A/C] | *Ca_Desi_LG06* | 26.966 |
| Ca_Desi_SNP2710 | [A/G] | *Ca_Desi_LG06* | 27.064 |
| Ca_Desi_SNP2711 | [T/G] | *Ca_Desi_LG06* | 27.254 |
| Ca_Desi_SNP2712 | [C/T] | *Ca_Desi_LG06* | 27.556 |
| Ca_Desi_SNP2713 | [C/T] | *Ca_Desi_LG06* | 27.884 |
| Ca_Desi_SNP2714 | [C/A] | *Ca_Desi_LG06* | 28.375 |
| Ca_Desi_SNP2715 | [A/C] | *Ca_Desi_LG06* | 28.810 |
| Ca_Desi_SNP2716 | [T/G] | *Ca_Desi_LG06* | 28.980 |
| Ca_Desi_SNP2717 | [C/A] | *Ca_Desi_LG06* | 29.138 |
| Ca_Desi_SNP2718 | [G/A] | *Ca_Desi_LG06* | 29.484 |
| Ca_Desi_SNP2719 | [A/C] | *Ca_Desi_LG06* | 29.517 |
| Ca_Desi_SNP2720 | [G/A] | *Ca_Desi_LG06* | 29.608 |
| Ca_Desi_SNP2721 | [C/A] | *Ca_Desi_LG06* | 29.628 |
| Ca_Desi_SNP2722 | [A/C] | *Ca_Desi_LG06* | 29.665 |
| Ca_Desi_SNP2723 | [G/C] | *Ca_Desi_LG06* | 29.855 |
| Ca_Desi_SNP2724 | [G/A] | *Ca_Desi_LG06* | 29.875 |
| Ca_Desi_SNP2725 | [G/A] | *Ca_Desi_LG06* | 30.145 |
| Ca_Desi_SNP2726 | [G/A] | *Ca_Desi_LG06* | 30.173 |
| Ca_Desi_SNP2727 | [C/A] | *Ca_Desi_LG06* | 30.336 |
| Ca_Desi_SNP2728 | [C/T] | *Ca_Desi_LG06* | 30.577 |
| Ca_Desi_SNP2729 | [T/A] | *Ca_Desi_LG06* | 30.961 |
| Ca_Desi_SNP2730 | [C/G] | *Ca_Desi_LG06* | 31.301 |
| Ca_Desi_SNP2731 | [A/G] | *Ca_Desi_LG06* | 31.317 |
| Ca_Desi_SNP2732 | [C/T] | *Ca_Desi_LG06* | 31.381 |
| Ca_Desi_SNP2733 | [T/A] | *Ca_Desi_LG06* | 31.403 |

| **SNP IDs** | **SNPs** | **Linkage Group (LGs)** | **Genetic positions (cM) of mapped SNPs** |
|---|---|---|---|
| Ca_Desi_SNP2734 | [G/C] | *Ca_Desi_LG06* | 31.413 |
| Ca_Desi_SNP2735 | [T/C] | *Ca_Desi_LG06* | 31.443 |
| Ca_Desi_SNP2736 | [C/T] | *Ca_Desi_LG06* | 31.646 |
| Ca_Desi_SNP2737 | [C/T] | *Ca_Desi_LG06* | 31.649 |
| Ca_Desi_SNP2738 | [G/C] | *Ca_Desi_LG06* | 31.935 |
| Ca_Desi_SNP2739 | [A/C] | *Ca_Desi_LG06* | 32.148 |
| Ca_Desi_SNP2740 | [T/A] | *Ca_Desi_LG06* | 32.308 |
| Ca_Desi_SNP2741 | [A/C] | *Ca_Desi_LG06* | 32.316 |
| Ca_Desi_SNP2742 | [C/A] | *Ca_Desi_LG06* | 32.950 |
| Ca_Desi_SNP2743 | [T/C] | *Ca_Desi_LG06* | 33.070 |
| Ca_Desi_SNP2744 | [T/C] | *Ca_Desi_LG06* | 33.278 |
| Ca_Desi_SNP2745 | [G/A] | *Ca_Desi_LG06* | 33.320 |
| Ca_Desi_SNP2746 | [C/T] | *Ca_Desi_LG06* | 33.462 |
| Ca_Desi_SNP2747 | [G/A] | *Ca_Desi_LG06* | 33.583 |
| Ca_Desi_SNP2748 | [A/C] | *Ca_Desi_LG06* | 34.054 |
| Ca_Desi_SNP2749 | [C/T] | *Ca_Desi_LG06* | 34.525 |
| Ca_Desi_SNP2750 | [T/G] | *Ca_Desi_LG06* | 34.712 |
| Ca_Desi_SNP2751 | [A/C] | *Ca_Desi_LG06* | 34.823 |
| Ca_Desi_SNP2752 | [T/A] | *Ca_Desi_LG06* | 34.856 |
| Ca_Desi_SNP2753 | [A/C] | *Ca_Desi_LG06* | 35.055 |
| Ca_Desi_SNP2754 | [T/G] | *Ca_Desi_LG06* | 35.444 |
| Ca_Desi_SNP2755 | [A/C] | *Ca_Desi_LG06* | 35.541 |
| Ca_Desi_SNP2756 | [T/C] | *Ca_Desi_LG06* | 35.860 |
| Ca_Desi_SNP2757 | [G/T] | *Ca_Desi_LG06* | 35.894 |
| Ca_Desi_SNP2758 | [A/C] | *Ca_Desi_LG06* | 35.988 |
| Ca_Desi_SNP2759 | [G/T] | *Ca_Desi_LG06* | 35.989 |
| Ca_Desi_SNP2760 | [C/T] | *Ca_Desi_LG06* | 36.576 |
| Ca_Desi_SNP2761 | [T/C] | *Ca_Desi_LG06* | 36.685 |
| Ca_Desi_SNP2762 | [A/G] | *Ca_Desi_LG06* | 36.868 |
| Ca_Desi_SNP2763 | [G/A] | *Ca_Desi_LG06* | 37.069 |
| Ca_Desi_SNP2764 | [T/C] | *Ca_Desi_LG06* | 37.103 |
| Ca_Desi_SNP2765 | [A/G] | *Ca_Desi_LG06* | 37.199 |
| Ca_Desi_SNP2766 | [C/T] | *Ca_Desi_LG06* | 37.225 |
| Ca_Desi_SNP2767 | [G/A] | *Ca_Desi_LG06* | 37.517 |
| Ca_Desi_SNP2768 | [A/G] | *Ca_Desi_LG06* | 37.574 |
| Ca_Desi_SNP2769 | [C/T] | *Ca_Desi_LG06* | 37.587 |
| Ca_Desi_SNP2770 | [C/G] | *Ca_Desi_LG06* | 37.703 |
| Ca_Desi_SNP2771 | [C/G] | *Ca_Desi_LG06* | 37.828 |
| Ca_Desi_SNP2772 | [A/T] | *Ca_Desi_LG06* | 37.987 |
| Ca_Desi_SNP2773 | [T/A] | *Ca_Desi_LG06* | 38.109 |
| Ca_Desi_SNP2774 | [G/A] | *Ca_Desi_LG06* | 38.353 |
| Ca_Desi_SNP2775 | [A/G] | *Ca_Desi_LG06* | 38.398 |
| Ca_Desi_SNP2776 | [C/A] | *Ca_Desi_LG06* | 38.707 |
| Ca_Desi_SNP2777 | [T/C] | *Ca_Desi_LG06* | 38.738 |
| Ca_Desi_SNP2778 | [T/C] | *Ca_Desi_LG06* | 39.011 |
| Ca_Desi_SNP2779 | [C/T] | *Ca_Desi_LG06* | 39.014 |
| Ca_Desi_SNP2780 | [G/A] | *Ca_Desi_LG06* | 39.117 |
| Ca_Desi_SNP2781 | [A/C] | *Ca_Desi_LG06* | 39.450 |

| **SNP IDs** | **SNPs** | **Linkage Group (LGs)** | **Genetic positions (cM) of mapped SNPs** |
|---|---|---|---|
| Ca_Desi_SNP2782 | [G/A] | *Ca_Desi_LG06* | 39.676 |
| Ca_Desi_SNP2783 | [T/A] | *Ca_Desi_LG06* | 39.819 |
| Ca_Desi_SNP2784 | [T/G] | *Ca_Desi_LG06* | 40.017 |
| Ca_Desi_SNP2785 | [C/T] | *Ca_Desi_LG06* | 40.017 |
| Ca_Desi_SNP2786 | [C/T] | *Ca_Desi_LG06* | 40.360 |
| Ca_Desi_SNP2787 | [A/G] | *Ca_Desi_LG06* | 40.789 |
| Ca_Desi_SNP2788 | [G/T] | *Ca_Desi_LG06* | 40.881 |
| Ca_Desi_SNP2789 | [T/C] | *Ca_Desi_LG06* | 41.035 |
| Ca_Desi_SNP2790 | [A/G] | *Ca_Desi_LG06* | 41.075 |
| Ca_Desi_SNP2791 | [T/C] | *Ca_Desi_LG06* | 41.088 |
| Ca_Desi_SNP2792 | [T/A] | *Ca_Desi_LG06* | 41.264 |
| Ca_Desi_SNP2793 | [T/C] | *Ca_Desi_LG06* | 41.490 |
| Ca_Desi_SNP2794 | [C/A] | *Ca_Desi_LG06* | 41.551 |
| Ca_Desi_SNP2795 | [C/A] | *Ca_Desi_LG06* | 41.576 |
| Ca_Desi_SNP2796 | [C/T] | *Ca_Desi_LG06* | 41.817 |
| Ca_Desi_SNP2797 | [G/A] | *Ca_Desi_LG06* | 41.932 |
| Ca_Desi_SNP2798 | [A/G] | *Ca_Desi_LG06* | 42.006 |
| Ca_Desi_SNP2799 | [A/C] | *Ca_Desi_LG06* | 42.041 |
| Ca_Desi_SNP2800 | [G/A] | *Ca_Desi_LG06* | 42.168 |
| Ca_Desi_SNP2801 | [A/T] | *Ca_Desi_LG06* | 42.249 |
| Ca_Desi_SNP2802 | [C/T] | *Ca_Desi_LG06* | 42.387 |
| Ca_Desi_SNP2803 | [C/T] | *Ca_Desi_LG06* | 42.654 |
| Ca_Desi_SNP2804 | [G/T] | *Ca_Desi_LG06* | 42.714 |
| Ca_Desi_SNP2805 | [C/T] | *Ca_Desi_LG06* | 42.775 |
| Ca_Desi_SNP2806 | [T/G] | *Ca_Desi_LG06* | 42.820 |
| Ca_Desi_SNP2807 | [T/C] | *Ca_Desi_LG06* | 43.307 |
| Ca_Desi_SNP2808 | [A/C] | *Ca_Desi_LG06* | 43.414 |
| Ca_Desi_SNP2809 | [A/G] | *Ca_Desi_LG06* | 43.554 |
| Ca_Desi_SNP2810 | [T/C] | *Ca_Desi_LG06* | 43.588 |
| Ca_Desi_SNP2811 | [T/C] | *Ca_Desi_LG06* | 43.649 |
| Ca_Desi_SNP2812 | [T/G] | *Ca_Desi_LG06* | 43.679 |
| Ca_Desi_SNP2813 | [G/A] | *Ca_Desi_LG06* | 44.023 |
| Ca_Desi_SNP2814 | [A/G] | *Ca_Desi_LG06* | 44.114 |
| Ca_Desi_SNP2815 | [A/G] | *Ca_Desi_LG06* | 44.665 |
| Ca_Desi_SNP2816 | [T/C] | *Ca_Desi_LG06* | 44.774 |
| Ca_Desi_SNP2817 | [C/T] | *Ca_Desi_LG06* | 44.879 |
| Ca_Desi_SNP2818 | [A/C] | *Ca_Desi_LG06* | 44.881 |
| Ca_Desi_SNP2819 | [A/C] | *Ca_Desi_LG06* | 45.109 |
| Ca_Desi_SNP2820 | [T/C] | *Ca_Desi_LG06* | 45.170 |
| Ca_Desi_SNP2821 | [G/A] | *Ca_Desi_LG06* | 45.229 |
| Ca_Desi_SNP2822 | [T/C] | *Ca_Desi_LG06* | 45.231 |
| Ca_Desi_SNP2823 | [T/C] | *Ca_Desi_LG06* | 45.469 |
| Ca_Desi_SNP2824 | [G/T] | *Ca_Desi_LG06* | 45.490 |
| Ca_Desi_SNP2825 | [A/G] | *Ca_Desi_LG06* | 45.510 |
| Ca_Desi_SNP2826 | [C/T] | *Ca_Desi_LG06* | 45.532 |
| Ca_Desi_SNP2827 | [G/T] | *Ca_Desi_LG06* | 45.588 |
| Ca_Desi_SNP2828 | [T/A] | *Ca_Desi_LG06* | 45.683 |
| Ca_Desi_SNP2829 | [A/G] | *Ca_Desi_LG06* | 45.728 |

| **SNP IDs** | **SNPs** | **Linkage Group (LGs)** | **Genetic positions (cM) of mapped SNPs** |
|---|---|---|---|
| Ca_Desi_SNP2830 | [T/C] | *Ca_Desi_LG06* | 45.752 |
| Ca_Desi_SNP2831 | [T/C] | *Ca_Desi_LG06* | 45.796 |
| Ca_Desi_SNP2832 | [T/C] | *Ca_Desi_LG06* | 45.857 |
| Ca_Desi_SNP2833 | [G/A] | *Ca_Desi_LG06* | 46.039 |
| Ca_Desi_SNP2834 | [G/A] | *Ca_Desi_LG06* | 46.146 |
| Ca_Desi_SNP2835 | [A/C] | *Ca_Desi_LG06* | 46.160 |
| Ca_Desi_SNP2836 | [A/G] | *Ca_Desi_LG06* | 46.577 |
| Ca_Desi_SNP2837 | [A/C] | *Ca_Desi_LG06* | 46.666 |
| Ca_Desi_SNP2838 | [C/T] | *Ca_Desi_LG06* | 46.711 |
| Ca_Desi_SNP2839 | [A/C] | *Ca_Desi_LG06* | 46.750 |
| Ca_Desi_SNP2840 | [A/T] | *Ca_Desi_LG06* | 46.780 |
| Ca_Desi_SNP2841 | [A/G] | *Ca_Desi_LG06* | 46.794 |
| Ca_Desi_SNP2842 | [T/A] | *Ca_Desi_LG06* | 46.807 |
| Ca_Desi_SNP2843 | [T/G] | *Ca_Desi_LG06* | 47.138 |
| Ca_Desi_SNP2844 | [G/A] | *Ca_Desi_LG06* | 47.667 |
| Ca_Desi_SNP2845 | [A/C] | *Ca_Desi_LG06* | 47.682 |
| Ca_Desi_SNP2846 | [A/T] | *Ca_Desi_LG06* | 47.695 |
| Ca_Desi_SNP2847 | [A/C] | *Ca_Desi_LG06* | 48.051 |
| Ca_Desi_SNP2848 | [A/G] | *Ca_Desi_LG06* | 48.306 |
| Ca_Desi_SNP2849 | [T/G] | *Ca_Desi_LG06* | 48.449 |
| Ca_Desi_SNP2850 | [T/C] | *Ca_Desi_LG06* | 48.876 |
| Ca_Desi_SNP2851 | [C/G] | *Ca_Desi_LG06* | 48.931 |
| Ca_Desi_SNP2852 | [A/C] | *Ca_Desi_LG06* | 49.123 |
| Ca_Desi_SNP2853 | [T/A] | *Ca_Desi_LG06* | 49.242 |
| Ca_Desi_SNP2854 | [G/A] | *Ca_Desi_LG06* | 49.284 |
| Ca_Desi_SNP2855 | [T/C] | *Ca_Desi_LG06* | 49.692 |
| Ca_Desi_SNP2856 | [G/T] | *Ca_Desi_LG06* | 49.796 |
| Ca_Desi_SNP2857 | [A/G] | *Ca_Desi_LG06* | 49.970 |
| Ca_Desi_SNP2858 | [G/C] | *Ca_Desi_LG06* | 50.111 |
| Ca_Desi_SNP2859 | [T/G] | *Ca_Desi_LG06* | 50.172 |
| Ca_Desi_SNP2860 | [C/T] | *Ca_Desi_LG06* | 50.698 |
| Ca_Desi_SNP2861 | [T/C] | *Ca_Desi_LG06* | 50.758 |
| Ca_Desi_SNP2862 | [T/C] | *Ca_Desi_LG06* | 50.820 |
| Ca_Desi_SNP2863 | [A/G] | *Ca_Desi_LG06* | 51.156 |
| Ca_Desi_SNP2864 | [A/C] | *Ca_Desi_LG06* | 51.292 |
| Ca_Desi_SNP2865 | [T/G] | *Ca_Desi_LG06* | 51.315 |
| Ca_Desi_SNP2866 | [A/G] | *Ca_Desi_LG06* | 51.944 |
| Ca_Desi_SNP2867 | [C/A] | *Ca_Desi_LG06* | 51.946 |
| Ca_Desi_SNP2868 | [A/C] | *Ca_Desi_LG06* | 51.965 |
| Ca_Desi_SNP2869 | [T/C] | *Ca_Desi_LG06* | 52.199 |
| Ca_Desi_SNP2870 | [T/C] | *Ca_Desi_LG06* | 52.641 |
| Ca_Desi_SNP2871 | [A/C] | *Ca_Desi_LG06* | 52.658 |
| Ca_Desi_SNP2872 | [G/A] | *Ca_Desi_LG06* | 52.909 |
| Ca_Desi_SNP2873 | [T/C] | *Ca_Desi_LG06* | 52.993 |
| Ca_Desi_SNP2874 | [T/C] | *Ca_Desi_LG06* | 53.028 |
| Ca_Desi_SNP2875 | [T/C] | *Ca_Desi_LG06* | 53.311 |
| Ca_Desi_SNP2876 | [C/T] | *Ca_Desi_LG06* | 53.400 |
| Ca_Desi_SNP2877 | [T/G] | *Ca_Desi_LG06* | 53.424 |

| **SNP IDs** | **SNPs** | **Linkage Group (LGs)** | **Genetic positions (cM) of mapped SNPs** |
|---|---|---|---|
| Ca_Desi_SNP2878 | [G/A] | *Ca_Desi_LG06* | 53.451 |
| Ca_Desi_SNP2879 | [A/C] | *Ca_Desi_LG06* | 53.479 |
| Ca_Desi_SNP2880 | [A/C] | *Ca_Desi_LG06* | 53.846 |
| Ca_Desi_SNP2881 | [T/A] | *Ca_Desi_LG06* | 54.069 |
| Ca_Desi_SNP2882 | [A/G] | *Ca_Desi_LG06* | 54.073 |
| Ca_Desi_SNP2883 | [T/C] | *Ca_Desi_LG06* | 54.109 |
| Ca_Desi_SNP2884 | [G/T] | *Ca_Desi_LG06* | 54.164 |
| Ca_Desi_SNP2885 | [A/G] | *Ca_Desi_LG06* | 54.349 |
| Ca_Desi_SNP2886 | [A/T] | *Ca_Desi_LG06* | 54.569 |
| Ca_Desi_SNP2887 | [A/C] | *Ca_Desi_LG06* | 54.625 |
| Ca_Desi_SNP2888 | [G/A] | *Ca_Desi_LG06* | 54.676 |
| Ca_Desi_SNP2889 | [A/C] | *Ca_Desi_LG06* | 54.737 |
| Ca_Desi_SNP2890 | [A/G] | *Ca_Desi_LG06* | 54.800 |
| Ca_Desi_SNP2891 | [A/G] | *Ca_Desi_LG06* | 55.013 |
| Ca_Desi_SNP2892 | [T/C] | *Ca_Desi_LG06* | 55.028 |
| Ca_Desi_SNP2893 | [T/G] | *Ca_Desi_LG06* | 55.186 |
| Ca_Desi_SNP2894 | [A/C] | *Ca_Desi_LG06* | 55.424 |
| Ca_Desi_SNP2895 | [T/G] | *Ca_Desi_LG06* | 55.864 |
| Ca_Desi_SNP2896 | [T/C] | *Ca_Desi_LG06* | 55.946 |
| Ca_Desi_SNP2897 | [T/C] | *Ca_Desi_LG06* | 56.149 |
| Ca_Desi_SNP2898 | [C/T] | *Ca_Desi_LG06* | 56.494 |
| Ca_Desi_SNP2899 | [A/C] | *Ca_Desi_LG06* | 56.508 |
| Ca_Desi_SNP2900 | [A/G] | *Ca_Desi_LG06* | 56.539 |
| Ca_Desi_SNP2901 | [G/A] | *Ca_Desi_LG06* | 56.715 |
| Ca_Desi_SNP2902 | [G/T] | *Ca_Desi_LG06* | 56.733 |
| Ca_Desi_SNP2903 | [A/C] | *Ca_Desi_LG06* | 56.755 |
| Ca_Desi_SNP2904 | [A/C] | *Ca_Desi_LG06* | 57.250 |
| Ca_Desi_SNP2905 | [C/T] | *Ca_Desi_LG06* | 57.254 |
| Ca_Desi_SNP2906 | [A/C] | *Ca_Desi_LG06* | 57.325 |
| Ca_Desi_SNP2907 | [T/G] | *Ca_Desi_LG06* | 57.422 |
| Ca_Desi_SNP2908 | [C/A] | *Ca_Desi_LG06* | 57.425 |
| Ca_Desi_SNP2909 | [A/G] | *Ca_Desi_LG06* | 57.677 |
| Ca_Desi_SNP2910 | [T/A] | *Ca_Desi_LG06* | 57.730 |
| Ca_Desi_SNP2911 | [T/A] | *Ca_Desi_LG06* | 57.871 |
| Ca_Desi_SNP2912 | [G/T] | *Ca_Desi_LG06* | 57.966 |
| Ca_Desi_SNP2913 | [A/C] | *Ca_Desi_LG06* | 58.050 |
| Ca_Desi_SNP2914 | [T/C] | *Ca_Desi_LG06* | 58.157 |
| Ca_Desi_SNP2915 | [G/T] | *Ca_Desi_LG06* | 58.644 |
| Ca_Desi_SNP2916 | [A/C] | *Ca_Desi_LG06* | 58.744 |
| Ca_Desi_SNP2917 | [T/G] | *Ca_Desi_LG06* | 58.979 |
| Ca_Desi_SNP2918 | [A/G] | *Ca_Desi_LG06* | 59.178 |
| Ca_Desi_SNP2919 | [C/T] | *Ca_Desi_LG06* | 59.362 |
| Ca_Desi_SNP2920 | [C/T] | *Ca_Desi_LG06* | 59.512 |
| Ca_Desi_SNP2921 | [T/C] | *Ca_Desi_LG06* | 59.532 |
| Ca_Desi_SNP2922 | [C/T] | *Ca_Desi_LG06* | 59.836 |
| Ca_Desi_SNP2923 | [G/A] | *Ca_Desi_LG06* | 59.900 |
| Ca_Desi_SNP2924 | [T/C] | *Ca_Desi_LG06* | 60.127 |
| Ca_Desi_SNP2925 | [A/C] | *Ca_Desi_LG06* | 60.288 |

| **SNP IDs** | **SNPs** | **Linkage Group (LGs)** | **Genetic positions (cM) of mapped SNPs** |
|---|---|---|---|
| Ca_Desi_SNP2926 | [A/T] | *Ca_Desi_LG06* | 60.352 |
| Ca_Desi_SNP2927 | [T/C] | *Ca_Desi_LG06* | 60.530 |
| Ca_Desi_SNP2928 | [G/A] | *Ca_Desi_LG06* | 60.885 |
| Ca_Desi_SNP2929 | [G/A] | *Ca_Desi_LG06* | 60.964 |
| Ca_Desi_SNP2930 | [C/A] | *Ca_Desi_LG06* | 61.967 |
| Ca_Desi_SNP2931 | [C/A] | *Ca_Desi_LG06* | 62.038 |
| Ca_Desi_SNP2932 | [T/C] | *Ca_Desi_LG06* | 62.256 |
| Ca_Desi_SNP2933 | [G/T] | *Ca_Desi_LG06* | 62.381 |
| Ca_Desi_SNP2934 | [C/A] | *Ca_Desi_LG06* | 62.480 |
| Ca_Desi_SNP2935 | [A/G] | *Ca_Desi_LG06* | 62.677 |
| Ca_Desi_SNP2936 | [A/T] | *Ca_Desi_LG06* | 62.735 |
| Ca_Desi_SNP2937 | [G/A] | *Ca_Desi_LG06* | 62.811 |
| Ca_Desi_SNP2938 | [G/A] | *Ca_Desi_LG06* | 62.986 |
| Ca_Desi_SNP2939 | [T/C] | *Ca_Desi_LG06* | 63.015 |
| Ca_Desi_SNP2940 | [A/G] | *Ca_Desi_LG06* | 63.173 |
| Ca_Desi_SNP2941 | [C/T] | *Ca_Desi_LG06* | 63.288 |
| Ca_Desi_SNP2942 | [T/C] | *Ca_Desi_LG06* | 63.290 |
| Ca_Desi_SNP2943 | [G/C] | *Ca_Desi_LG06* | 63.369 |
| Ca_Desi_SNP2944 | [T/C] | *Ca_Desi_LG06* | 63.533 |
| Ca_Desi_SNP2945 | [G/C] | *Ca_Desi_LG06* | 63.684 |
| Ca_Desi_SNP2946 | [T/A] | *Ca_Desi_LG06* | 63.888 |
| Ca_Desi_SNP2947 | [C/T] | *Ca_Desi_LG06* | 63.996 |
| Ca_Desi_SNP2948 | [A/G] | *Ca_Desi_LG06* | 64.307 |
| Ca_Desi_SNP2949 | [C/T] | *Ca_Desi_LG06* | 64.578 |
| Ca_Desi_SNP2950 | [T/G] | *Ca_Desi_LG06* | 64.725 |
| Ca_Desi_SNP2951 | [G/A] | *Ca_Desi_LG06* | 64.900 |
| Ca_Desi_SNP2952 | [C/G] | *Ca_Desi_LG06* | 65.074 |
| Ca_Desi_SNP2953 | [G/A] | *Ca_Desi_LG06* | 65.144 |
| Ca_Desi_SNP2954 | [T/C] | *Ca_Desi_LG06* | 65.806 |
| Ca_Desi_SNP2955 | [A/C] | *Ca_Desi_LG06* | 65.837 |
| Ca_Desi_SNP2956 | [A/G] | *Ca_Desi_LG06* | 66.043 |
| Ca_Desi_SNP2957 | [A/G] | *Ca_Desi_LG06* | 66.046 |
| Ca_Desi_SNP2958 | [T/C] | *Ca_Desi_LG06* | 66.092 |
| Ca_Desi_SNP2959 | [T/G] | *Ca_Desi_LG06* | 66.369 |
| Ca_Desi_SNP2960 | [T/G] | *Ca_Desi_LG06* | 66.699 |
| Ca_Desi_SNP2961 | [A/C] | *Ca_Desi_LG06* | 66.754 |
| Ca_Desi_SNP2962 | [T/C] | *Ca_Desi_LG06* | 67.060 |
| Ca_Desi_SNP2963 | [C/T] | *Ca_Desi_LG06* | 67.196 |
| Ca_Desi_SNP2964 | [G/T] | *Ca_Desi_LG06* | 67.790 |
| Ca_Desi_SNP2965 | [A/G] | *Ca_Desi_LG06* | 67.997 |
| Ca_Desi_SNP2966 | [C/T] | *Ca_Desi_LG06* | 68.102 |
| Ca_Desi_SNP2967 | [A/C] | *Ca_Desi_LG06* | 68.282 |
| Ca_Desi_SNP2968 | [A/G] | *Ca_Desi_LG06* | 68.471 |
| Ca_Desi_SNP2969 | [T/G] | *Ca_Desi_LG06* | 68.560 |
| Ca_Desi_SNP2970 | [G/T] | *Ca_Desi_LG06* | 68.564 |
| Ca_Desi_SNP2971 | [T/G] | *Ca_Desi_LG06* | 69.062 |
| Ca_Desi_SNP2972 | [C/G] | *Ca_Desi_LG06* | 69.223 |
| Ca_Desi_SNP2973 | [G/A] | *Ca_Desi_LG06* | 69.407 |

| **SNP IDs** | **SNPs** | **Linkage Group (LGs)** | **Genetic positions (cM) of mapped SNPs** |
|---|---|---|---|
| Ca_Desi_SNP2974 | [T/C] | *Ca_Desi_LG06* | 69.439 |
| Ca_Desi_SNP2975 | [T/C] | *Ca_Desi_LG06* | 69.735 |
| Ca_Desi_SNP2976 | [G/A] | *Ca_Desi_LG06* | 69.785 |
| Ca_Desi_SNP2977 | [T/C] | *Ca_Desi_LG06* | 70.041 |
| Ca_Desi_SNP2978 | [T/C] | *Ca_Desi_LG06* | 70.071 |
| Ca_Desi_SNP2979 | [A/G] | *Ca_Desi_LG06* | 70.322 |
| Ca_Desi_SNP2980 | [T/C] | *Ca_Desi_LG06* | 70.536 |
| Ca_Desi_SNP2981 | [G/C] | *Ca_Desi_LG06* | 71.494 |
| Ca_Desi_SNP2982 | [A/T] | *Ca_Desi_LG06* | 72.057 |
| Ca_Desi_SNP2983 | [T/G] | *Ca_Desi_LG06* | 72.216 |
| Ca_Desi_SNP2984 | [T/C] | *Ca_Desi_LG06* | 72.274 |
| Ca_Desi_SNP2985 | [A/G] | *Ca_Desi_LG06* | 73.465 |
| Ca_Desi_SNP2986 | [A/G] | *Ca_Desi_LG06* | 73.544 |
| Ca_Desi_SNP2987 | [T/C] | *Ca_Desi_LG06* | 73.577 |
| Ca_Desi_SNP2988 | [T/G] | *Ca_Desi_LG06* | 73.994 |
| Ca_Desi_SNP2989 | [T/C] | *Ca_Desi_LG06* | 75.127 |
| Ca_Desi_SNP2990 | [G/A] | *Ca_Desi_LG06* | 75.986 |
| Ca_Desi_SNP2991 | [T/C] | *Ca_Desi_LG06* | 76.795 |
| Ca_Desi_SNP2992 | [A/G] | *Ca_Desi_LG06* | 76.952 |
| Ca_Desi_SNP2993 | [A/T] | *Ca_Desi_LG06* | 77.157 |
| Ca_Desi_SNP2994 | [C/T] | *Ca_Desi_LG06* | 77.497 |
| Ca_Desi_SNP2995 | [C/A] | *Ca_Desi_LG06* | 78.929 |
| Ca_Desi_SNP2996 | [T/A] | *Ca_Desi_LG06* | 79.362 |
| Ca_Desi_SNP2997 | [T/C] | *Ca_Desi_LG06* | 79.404 |
| Ca_Desi_SNP2998 | [C/T] | *Ca_Desi_LG06* | 82.775 |
| Ca_Desi_SNP2999 | [G/T] | *Ca_Desi_LG06* | 83.224 |
| Ca_Desi_SNP3000 | [G/A] | *Ca_Desi_LG06* | 84.404 |
| Ca_Desi_SNP3001 | [A/G] | *Ca_Desi_LG06* | 84.604 |
| Ca_Desi_SNP3002 | [A/G] | *Ca_Desi_LG06* | 85.048 |
| Ca_Desi_SNP3003 | [T/C] | *Ca_Desi_LG06* | 85.182 |
| Ca_Desi_SNP3004 | [A/G] | *Ca_Desi_LG06* | 86.201 |
| Ca_Desi_SNP3005 | [G/T] | *Ca_Desi_LG06* | 87.601 |
| Ca_Desi_SNP3006 | [T/G] | *Ca_Desi_LG07* | 0.000 |
| Ca_Desi_SNP3007 | [T/C] | *Ca_Desi_LG07* | 1.014 |
| Ca_Desi_SNP3008 | [T/C] | *Ca_Desi_LG07* | 1.550 |
| Ca_Desi_SNP3009 | [T/C] | *Ca_Desi_LG07* | 1.591 |
| Ca_Desi_SNP3010 | [A/C] | *Ca_Desi_LG07* | 1.652 |
| Ca_Desi_SNP3011 | [A/C] | *Ca_Desi_LG07* | 1.680 |
| Ca_Desi_SNP3012 | [T/C] | *Ca_Desi_LG07* | 2.858 |
| Ca_Desi_SNP3013 | [A/C] | *Ca_Desi_LG07* | 3.387 |
| Ca_Desi_SNP3014 | [C/A] | *Ca_Desi_LG07* | 3.414 |
| Ca_Desi_SNP3015 | [A/G] | *Ca_Desi_LG07* | 4.643 |
| Ca_Desi_SNP3016 | [T/G] | *Ca_Desi_LG07* | 4.961 |
| Ca_Desi_SNP3017 | [T/C] | *Ca_Desi_LG07* | 5.342 |
| Ca_Desi_SNP3018 | [C/A] | *Ca_Desi_LG07* | 6.094 |
| Ca_Desi_SNP3019 | [A/G] | *Ca_Desi_LG07* | 7.157 |
| Ca_Desi_SNP3020 | [A/C] | *Ca_Desi_LG07* | 7.166 |
| Ca_Desi_SNP3021 | [A/G] | *Ca_Desi_LG07* | 7.278 |

| **SNP IDs** | **SNPs** | **Linkage Group (LGs)** | **Genetic positions (cM) of mapped SNPs** |
|---|---|---|---|
| Ca_Desi_SNP3022 | [C/A] | *Ca_Desi_LG07* | 7.289 |
| Ca_Desi_SNP3023 | [A/G] | *Ca_Desi_LG07* | 7.403 |
| Ca_Desi_SNP3024 | [T/G] | *Ca_Desi_LG07* | 7.453 |
| Ca_Desi_SNP3025 | [A/C] | *Ca_Desi_LG07* | 7.687 |
| Ca_Desi_SNP3026 | [T/C] | *Ca_Desi_LG07* | 7.701 |
| Ca_Desi_SNP3027 | [A/G] | *Ca_Desi_LG07* | 7.745 |
| Ca_Desi_SNP3028 | [T/C] | *Ca_Desi_LG07* | 8.442 |
| Ca_Desi_SNP3029 | [A/C] | *Ca_Desi_LG07* | 8.444 |
| Ca_Desi_SNP3030 | [C/A] | *Ca_Desi_LG07* | 8.790 |
| Ca_Desi_SNP3031 | [T/C] | *Ca_Desi_LG07* | 8.873 |
| Ca_Desi_SNP3032 | [G/T] | *Ca_Desi_LG07* | 9.351 |
| Ca_Desi_SNP3033 | [C/G] | *Ca_Desi_LG07* | 9.932 |
| Ca_Desi_SNP3034 | [T/C] | *Ca_Desi_LG07* | 10.199 |
| Ca_Desi_SNP3035 | [C/T] | *Ca_Desi_LG07* | 10.304 |
| Ca_Desi_SNP3036 | [A/G] | *Ca_Desi_LG07* | 10.407 |
| Ca_Desi_SNP3037 | [T/C] | *Ca_Desi_LG07* | 10.424 |
| Ca_Desi_SNP3038 | [T/G] | *Ca_Desi_LG07* | 10.759 |
| Ca_Desi_SNP3039 | [A/C] | *Ca_Desi_LG07* | 10.942 |
| Ca_Desi_SNP3040 | [C/T] | *Ca_Desi_LG07* | 11.044 |
| Ca_Desi_SNP3041 | [C/A] | *Ca_Desi_LG07* | 11.363 |
| Ca_Desi_SNP3042 | [G/A] | *Ca_Desi_LG07* | 11.569 |
| Ca_Desi_SNP3043 | [T/C] | *Ca_Desi_LG07* | 12.006 |
| Ca_Desi_SNP3044 | [A/T] | *Ca_Desi_LG07* | 12.154 |
| Ca_Desi_SNP3045 | [A/G] | *Ca_Desi_LG07* | 12.285 |
| Ca_Desi_SNP3046 | [T/C] | *Ca_Desi_LG07* | 12.489 |
| Ca_Desi_SNP3047 | [G/A] | *Ca_Desi_LG07* | 13.236 |
| Ca_Desi_SNP3048 | [A/G] | *Ca_Desi_LG07* | 13.466 |
| Ca_Desi_SNP3049 | [A/G] | *Ca_Desi_LG07* | 13.613 |
| Ca_Desi_SNP3050 | [A/C] | *Ca_Desi_LG07* | 14.150 |
| Ca_Desi_SNP3051 | [A/G] | *Ca_Desi_LG07* | 14.259 |
| Ca_Desi_SNP3052 | [T/C] | *Ca_Desi_LG07* | 14.424 |
| Ca_Desi_SNP3053 | [A/C] | *Ca_Desi_LG07* | 14.724 |
| Ca_Desi_SNP3054 | [T/G] | *Ca_Desi_LG07* | 14.836 |
| Ca_Desi_SNP3055 | [T/C] | *Ca_Desi_LG07* | 15.299 |
| Ca_Desi_SNP3056 | [A/G] | *Ca_Desi_LG07* | 15.469 |
| Ca_Desi_SNP3057 | [A/C] | *Ca_Desi_LG07* | 15.869 |
| Ca_Desi_SNP3058 | [G/A] | *Ca_Desi_LG07* | 16.227 |
| Ca_Desi_SNP3059 | [A/G] | *Ca_Desi_LG07* | 16.242 |
| Ca_Desi_SNP3060 | [A/C] | *Ca_Desi_LG07* | 16.477 |
| Ca_Desi_SNP3061 | [A/G] | *Ca_Desi_LG07* | 16.485 |
| Ca_Desi_SNP3062 | [G/T] | *Ca_Desi_LG07* | 16.832 |
| Ca_Desi_SNP3063 | [T/C] | *Ca_Desi_LG07* | 16.852 |
| Ca_Desi_SNP3064 | [A/G] | *Ca_Desi_LG07* | 17.560 |
| Ca_Desi_SNP3065 | [T/G] | *Ca_Desi_LG07* | 17.648 |
| Ca_Desi_SNP3066 | [T/A] | *Ca_Desi_LG07* | 17.765 |
| Ca_Desi_SNP3067 | [T/C] | *Ca_Desi_LG07* | 17.857 |
| Ca_Desi_SNP3068 | [T/C] | *Ca_Desi_LG07* | 18.042 |
| Ca_Desi_SNP3069 | [C/A] | *Ca_Desi_LG07* | 18.335 |

| **SNP IDs** | **SNPs** | **Linkage Group (LGs)** | **Genetic positions (cM) of mapped SNPs** |
|---|---|---|---|
| Ca_Desi_SNP3070 | [T/C] | *Ca_Desi_LG07* | 18.727 |
| Ca_Desi_SNP3071 | [A/C] | *Ca_Desi_LG07* | 19.035 |
| Ca_Desi_SNP3072 | [A/G] | *Ca_Desi_LG07* | 20.681 |
| Ca_Desi_SNP3073 | [T/C] | *Ca_Desi_LG07* | 20.687 |
| Ca_Desi_SNP3074 | [T/A] | *Ca_Desi_LG07* | 20.890 |
| Ca_Desi_SNP3075 | [T/C] | *Ca_Desi_LG07* | 21.296 |
| Ca_Desi_SNP3076 | [T/C] | *Ca_Desi_LG07* | 21.648 |
| Ca_Desi_SNP3077 | [C/A] | *Ca_Desi_LG07* | 22.457 |
| Ca_Desi_SNP3078 | [T/C] | *Ca_Desi_LG07* | 22.653 |
| Ca_Desi_SNP3079 | [T/C] | *Ca_Desi_LG07* | 22.774 |
| Ca_Desi_SNP3080 | [A/G] | *Ca_Desi_LG07* | 22.866 |
| Ca_Desi_SNP3081 | [G/C] | *Ca_Desi_LG07* | 23.190 |
| Ca_Desi_SNP3082 | [T/C] | *Ca_Desi_LG07* | 23.508 |
| Ca_Desi_SNP3083 | [C/A] | *Ca_Desi_LG07* | 23.542 |
| Ca_Desi_SNP3084 | [A/G] | *Ca_Desi_LG07* | 23.584 |
| Ca_Desi_SNP3085 | [A/C] | *Ca_Desi_LG07* | 24.208 |
| Ca_Desi_SNP3086 | [A/G] | *Ca_Desi_LG07* | 24.422 |
| Ca_Desi_SNP3087 | [T/C] | *Ca_Desi_LG07* | 24.428 |
| Ca_Desi_SNP3088 | [T/G] | *Ca_Desi_LG07* | 24.584 |
| Ca_Desi_SNP3089 | [T/C] | *Ca_Desi_LG07* | 24.832 |
| Ca_Desi_SNP3090 | [C/A] | *Ca_Desi_LG07* | 24.837 |
| Ca_Desi_SNP3091 | [C/A] | *Ca_Desi_LG07* | 24.848 |
| Ca_Desi_SNP3092 | [T/C] | *Ca_Desi_LG07* | 24.926 |
| Ca_Desi_SNP3093 | [C/T] | *Ca_Desi_LG07* | 25.333 |
| Ca_Desi_SNP3094 | [C/T] | *Ca_Desi_LG07* | 25.472 |
| Ca_Desi_SNP3095 | [C/G] | *Ca_Desi_LG07* | 26.078 |
| Ca_Desi_SNP3096 | [T/G] | *Ca_Desi_LG07* | 26.279 |
| Ca_Desi_SNP3097 | [C/A] | *Ca_Desi_LG07* | 26.369 |
| Ca_Desi_SNP3098 | [G/C] | *Ca_Desi_LG07* | 26.633 |
| Ca_Desi_SNP3099 | [A/G] | *Ca_Desi_LG07* | 26.835 |
| Ca_Desi_SNP3100 | [A/G] | *Ca_Desi_LG07* | 26.879 |
| Ca_Desi_SNP3101 | [T/C] | *Ca_Desi_LG07* | 26.885 |
| Ca_Desi_SNP3102 | [A/G] | *Ca_Desi_LG07* | 27.167 |
| Ca_Desi_SNP3103 | [G/A] | *Ca_Desi_LG07* | 27.212 |
| Ca_Desi_SNP3104 | [T/C] | *Ca_Desi_LG07* | 27.261 |
| Ca_Desi_SNP3105 | [G/A] | *Ca_Desi_LG07* | 27.284 |
| Ca_Desi_SNP3106 | [A/C] | *Ca_Desi_LG07* | 27.457 |
| Ca_Desi_SNP3107 | [T/G] | *Ca_Desi_LG07* | 27.490 |
| Ca_Desi_SNP3108 | [G/C] | *Ca_Desi_LG07* | 27.560 |
| Ca_Desi_SNP3109 | [A/G] | *Ca_Desi_LG07* | 27.689 |
| Ca_Desi_SNP3110 | [C/T] | *Ca_Desi_LG07* | 27.751 |
| Ca_Desi_SNP3111 | [G/A] | *Ca_Desi_LG07* | 27.884 |
| Ca_Desi_SNP3112 | [C/T] | *Ca_Desi_LG07* | 28.137 |
| Ca_Desi_SNP3113 | [T/G] | *Ca_Desi_LG07* | 28.215 |
| Ca_Desi_SNP3114 | [C/T] | *Ca_Desi_LG07* | 28.398 |
| Ca_Desi_SNP3115 | [A/C] | *Ca_Desi_LG07* | 28.537 |
| Ca_Desi_SNP3116 | [A/C] | *Ca_Desi_LG07* | 28.627 |
| Ca_Desi_SNP3117 | [A/C] | *Ca_Desi_LG07* | 28.724 |

| **SNP IDs** | **SNPs** | **Linkage Group (LGs)** | **Genetic positions (cM) of mapped SNPs** |
|---|---|---|---|
| Ca_Desi_SNP3118 | [T/G] | *Ca_Desi_LG07* | 28.746 |
| Ca_Desi_SNP3119 | [G/T] | *Ca_Desi_LG07* | 28.888 |
| Ca_Desi_SNP3120 | [C/T] | *Ca_Desi_LG07* | 29.134 |
| Ca_Desi_SNP3121 | [A/G] | *Ca_Desi_LG07* | 29.182 |
| Ca_Desi_SNP3122 | [A/C] | *Ca_Desi_LG07* | 29.504 |
| Ca_Desi_SNP3123 | [A/G] | *Ca_Desi_LG07* | 29.561 |
| Ca_Desi_SNP3124 | [A/G] | *Ca_Desi_LG07* | 29.689 |
| Ca_Desi_SNP3125 | [T/C] | *Ca_Desi_LG07* | 29.703 |
| Ca_Desi_SNP3126 | [T/C] | *Ca_Desi_LG07* | 29.822 |
| Ca_Desi_SNP3127 | [C/A] | *Ca_Desi_LG07* | 29.898 |
| Ca_Desi_SNP3128 | [G/A] | *Ca_Desi_LG07* | 30.307 |
| Ca_Desi_SNP3129 | [A/C] | *Ca_Desi_LG07* | 30.550 |
| Ca_Desi_SNP3130 | [C/T] | *Ca_Desi_LG07* | 30.789 |
| Ca_Desi_SNP3131 | [T/G] | *Ca_Desi_LG07* | 30.951 |
| Ca_Desi_SNP3132 | [T/C] | *Ca_Desi_LG07* | 31.012 |
| Ca_Desi_SNP3133 | [T/G] | *Ca_Desi_LG07* | 31.029 |
| Ca_Desi_SNP3134 | [T/G] | *Ca_Desi_LG07* | 31.040 |
| Ca_Desi_SNP3135 | [A/T] | *Ca_Desi_LG07* | 31.195 |
| Ca_Desi_SNP3136 | [C/T] | *Ca_Desi_LG07* | 31.196 |
| Ca_Desi_SNP3137 | [T/C] | *Ca_Desi_LG07* | 31.249 |
| Ca_Desi_SNP3138 | [T/C] | *Ca_Desi_LG07* | 31.396 |
| Ca_Desi_SNP3139 | [G/A] | *Ca_Desi_LG07* | 31.429 |
| Ca_Desi_SNP3140 | [A/G] | *Ca_Desi_LG07* | 31.520 |
| Ca_Desi_SNP3141 | [C/G] | *Ca_Desi_LG07* | 31.609 |
| Ca_Desi_SNP3142 | [T/G] | *Ca_Desi_LG07* | 31.838 |
| Ca_Desi_SNP3143 | [G/A] | *Ca_Desi_LG07* | 31.919 |
| Ca_Desi_SNP3144 | [T/C] | *Ca_Desi_LG07* | 32.292 |
| Ca_Desi_SNP3145 | [G/T] | *Ca_Desi_LG07* | 32.329 |
| Ca_Desi_SNP3146 | [G/A] | *Ca_Desi_LG07* | 32.371 |
| Ca_Desi_SNP3147 | [A/T] | *Ca_Desi_LG07* | 32.502 |
| Ca_Desi_SNP3148 | [T/C] | *Ca_Desi_LG07* | 32.506 |
| Ca_Desi_SNP3149 | [T/C] | *Ca_Desi_LG07* | 32.586 |
| Ca_Desi_SNP3150 | [A/G] | *Ca_Desi_LG07* | 32.997 |
| Ca_Desi_SNP3151 | [C/G] | *Ca_Desi_LG07* | 33.246 |
| Ca_Desi_SNP3152 | [A/G] | *Ca_Desi_LG07* | 33.316 |
| Ca_Desi_SNP3153 | [G/A] | *Ca_Desi_LG07* | 33.544 |
| Ca_Desi_SNP3154 | [G/A] | *Ca_Desi_LG07* | 33.718 |
| Ca_Desi_SNP3155 | [T/C] | *Ca_Desi_LG07* | 33.835 |
| Ca_Desi_SNP3156 | [A/C] | *Ca_Desi_LG07* | 34.103 |
| Ca_Desi_SNP3157 | [T/A] | *Ca_Desi_LG07* | 34.458 |
| Ca_Desi_SNP3158 | [C/T] | *Ca_Desi_LG07* | 34.818 |
| Ca_Desi_SNP3159 | [T/C] | *Ca_Desi_LG07* | 34.858 |
| Ca_Desi_SNP3160 | [A/G] | *Ca_Desi_LG07* | 35.043 |
| Ca_Desi_SNP3161 | [A/G] | *Ca_Desi_LG07* | 35.227 |
| Ca_Desi_SNP3162 | [C/T] | *Ca_Desi_LG07* | 35.292 |
| Ca_Desi_SNP3163 | [A/T] | *Ca_Desi_LG07* | 35.298 |
| Ca_Desi_SNP3164 | [A/G] | *Ca_Desi_LG07* | 35.433 |
| Ca_Desi_SNP3165 | [T/C] | *Ca_Desi_LG07* | 35.434 |

| **SNP IDs** | **SNPs** | **Linkage Group (LGs)** | **Genetic positions (cM) of mapped SNPs** |
|---|---|---|---|
| Ca_Desi_SNP3166 | [A/T] | *Ca_Desi_LG07* | 35.496 |
| Ca_Desi_SNP3167 | [T/C] | *Ca_Desi_LG07* | 35.587 |
| Ca_Desi_SNP3168 | [G/A] | *Ca_Desi_LG07* | 35.875 |
| Ca_Desi_SNP3169 | [A/C] | *Ca_Desi_LG07* | 35.916 |
| Ca_Desi_SNP3170 | [T/G] | *Ca_Desi_LG07* | 36.337 |
| Ca_Desi_SNP3171 | [C/T] | *Ca_Desi_LG07* | 36.383 |
| Ca_Desi_SNP3172 | [T/G] | *Ca_Desi_LG07* | 36.436 |
| Ca_Desi_SNP3173 | [A/G] | *Ca_Desi_LG07* | 36.457 |
| Ca_Desi_SNP3174 | [C/A] | *Ca_Desi_LG07* | 36.459 |
| Ca_Desi_SNP3175 | [G/C] | *Ca_Desi_LG07* | 36.511 |
| Ca_Desi_SNP3176 | [A/G] | *Ca_Desi_LG07* | 36.530 |
| Ca_Desi_SNP3177 | [T/A] | *Ca_Desi_LG07* | 36.621 |
| Ca_Desi_SNP3178 | [C/T] | *Ca_Desi_LG07* | 36.707 |
| Ca_Desi_SNP3179 | [T/G] | *Ca_Desi_LG07* | 36.909 |
| Ca_Desi_SNP3180 | [C/T] | *Ca_Desi_LG07* | 37.333 |
| Ca_Desi_SNP3181 | [G/T] | *Ca_Desi_LG07* | 37.671 |
| Ca_Desi_SNP3182 | [T/G] | *Ca_Desi_LG07* | 37.711 |
| Ca_Desi_SNP3183 | [G/T] | *Ca_Desi_LG07* | 37.785 |
| Ca_Desi_SNP3184 | [A/G] | *Ca_Desi_LG07* | 38.125 |
| Ca_Desi_SNP3185 | [G/T] | *Ca_Desi_LG07* | 38.563 |
| Ca_Desi_SNP3186 | [G/A] | *Ca_Desi_LG07* | 38.607 |
| Ca_Desi_SNP3187 | [C/T] | *Ca_Desi_LG07* | 38.618 |
| Ca_Desi_SNP3188 | [A/C] | *Ca_Desi_LG07* | 39.289 |
| Ca_Desi_SNP3189 | [T/G] | *Ca_Desi_LG07* | 39.329 |
| Ca_Desi_SNP3190 | [C/T] | *Ca_Desi_LG07* | 39.470 |
| Ca_Desi_SNP3191 | [A/T] | *Ca_Desi_LG07* | 39.649 |
| Ca_Desi_SNP3192 | [G/A] | *Ca_Desi_LG07* | 39.672 |
| Ca_Desi_SNP3193 | [C/A] | *Ca_Desi_LG07* | 39.735 |
| Ca_Desi_SNP3194 | [T/G] | *Ca_Desi_LG07* | 40.143 |
| Ca_Desi_SNP3195 | [A/G] | *Ca_Desi_LG07* | 40.432 |
| Ca_Desi_SNP3196 | [C/G] | *Ca_Desi_LG07* | 40.563 |
| Ca_Desi_SNP3197 | [G/A] | *Ca_Desi_LG07* | 40.602 |
| Ca_Desi_SNP3198 | [G/A] | *Ca_Desi_LG07* | 40.724 |
| Ca_Desi_SNP3199 | [C/T] | *Ca_Desi_LG07* | 41.434 |
| Ca_Desi_SNP3200 | [C/T] | *Ca_Desi_LG07* | 41.561 |
| Ca_Desi_SNP3201 | [C/T] | *Ca_Desi_LG07* | 41.623 |
| Ca_Desi_SNP3202 | [C/T] | *Ca_Desi_LG07* | 41.854 |
| Ca_Desi_SNP3203 | [A/G] | *Ca_Desi_LG07* | 41.898 |
| Ca_Desi_SNP3204 | [G/T] | *Ca_Desi_LG07* | 41.977 |
| Ca_Desi_SNP3205 | [A/T] | *Ca_Desi_LG07* | 41.998 |
| Ca_Desi_SNP3206 | [A/C] | *Ca_Desi_LG07* | 41.999 |
| Ca_Desi_SNP3207 | [T/C] | *Ca_Desi_LG07* | 42.100 |
| Ca_Desi_SNP3208 | [T/G] | *Ca_Desi_LG07* | 42.522 |
| Ca_Desi_SNP3209 | [G/T] | *Ca_Desi_LG07* | 42.561 |
| Ca_Desi_SNP3210 | [T/G] | *Ca_Desi_LG07* | 42.606 |
| Ca_Desi_SNP3211 | [A/G] | *Ca_Desi_LG07* | 42.800 |
| Ca_Desi_SNP3212 | [T/G] | *Ca_Desi_LG07* | 42.862 |
| Ca_Desi_SNP3213 | [G/A] | *Ca_Desi_LG07* | 42.897 |

| **SNP IDs** | **SNPs** | **Linkage Group (LGs)** | **Genetic positions (cM) of mapped SNPs** |
|---|---|---|---|
| Ca_Desi_SNP3214 | [A/G] | *Ca_Desi_LG07* | 42.933 |
| Ca_Desi_SNP3215 | [A/T] | *Ca_Desi_LG07* | 43.070 |
| Ca_Desi_SNP3216 | [T/C] | *Ca_Desi_LG07* | 43.071 |
| Ca_Desi_SNP3217 | [C/G] | *Ca_Desi_LG07* | 43.110 |
| Ca_Desi_SNP3218 | [G/A] | *Ca_Desi_LG07* | 43.228 |
| Ca_Desi_SNP3219 | [A/G] | *Ca_Desi_LG07* | 43.405 |
| Ca_Desi_SNP3220 | [T/C] | *Ca_Desi_LG07* | 43.529 |
| Ca_Desi_SNP3221 | [T/C] | *Ca_Desi_LG07* | 43.654 |
| Ca_Desi_SNP3222 | [A/G] | *Ca_Desi_LG07* | 43.825 |
| Ca_Desi_SNP3223 | [A/G] | *Ca_Desi_LG07* | 43.830 |
| Ca_Desi_SNP3224 | [T/C] | *Ca_Desi_LG07* | 43.866 |
| Ca_Desi_SNP3225 | [C/A] | *Ca_Desi_LG07* | 44.427 |
| Ca_Desi_SNP3226 | [A/C] | *Ca_Desi_LG07* | 44.534 |
| Ca_Desi_SNP3227 | [T/C] | *Ca_Desi_LG07* | 44.578 |
| Ca_Desi_SNP3228 | [C/T] | *Ca_Desi_LG07* | 44.735 |
| Ca_Desi_SNP3229 | [G/A] | *Ca_Desi_LG07* | 44.790 |
| Ca_Desi_SNP3230 | [G/A] | *Ca_Desi_LG07* | 44.792 |
| Ca_Desi_SNP3231 | [T/C] | *Ca_Desi_LG07* | 45.036 |
| Ca_Desi_SNP3232 | [A/G] | *Ca_Desi_LG07* | 45.282 |
| Ca_Desi_SNP3233 | [A/G] | *Ca_Desi_LG07* | 45.385 |
| Ca_Desi_SNP3234 | [T/C] | *Ca_Desi_LG07* | 45.604 |
| Ca_Desi_SNP3235 | [C/G] | *Ca_Desi_LG07* | 45.724 |
| Ca_Desi_SNP3236 | [C/A] | *Ca_Desi_LG07* | 45.791 |
| Ca_Desi_SNP3237 | [T/C] | *Ca_Desi_LG07* | 45.938 |
| Ca_Desi_SNP3238 | [T/C] | *Ca_Desi_LG07* | 47.058 |
| Ca_Desi_SNP3239 | [A/G] | *Ca_Desi_LG07* | 47.232 |
| Ca_Desi_SNP3240 | [C/A] | *Ca_Desi_LG07* | 47.290 |
| Ca_Desi_SNP3241 | [A/C] | *Ca_Desi_LG07* | 47.507 |
| Ca_Desi_SNP3242 | [A/G] | *Ca_Desi_LG07* | 48.289 |
| Ca_Desi_SNP3243 | [G/A] | *Ca_Desi_LG07* | 48.751 |
| Ca_Desi_SNP3244 | [C/A] | *Ca_Desi_LG07* | 48.954 |
| Ca_Desi_SNP3245 | [T/C] | *Ca_Desi_LG07* | 49.226 |
| Ca_Desi_SNP3246 | [A/G] | *Ca_Desi_LG07* | 49.284 |
| Ca_Desi_SNP3247 | [G/A] | *Ca_Desi_LG07* | 49.367 |
| Ca_Desi_SNP3248 | [A/C] | *Ca_Desi_LG07* | 49.377 |
| Ca_Desi_SNP3249 | [A/C] | *Ca_Desi_LG07* | 50.078 |
| Ca_Desi_SNP3250 | [C/T] | *Ca_Desi_LG07* | 50.317 |
| Ca_Desi_SNP3251 | [C/T] | *Ca_Desi_LG07* | 50.349 |
| Ca_Desi_SNP3252 | [T/C] | *Ca_Desi_LG07* | 50.395 |
| Ca_Desi_SNP3253 | [T/A] | *Ca_Desi_LG07* | 50.527 |
| Ca_Desi_SNP3254 | [T/G] | *Ca_Desi_LG07* | 51.257 |
| Ca_Desi_SNP3255 | [C/T] | *Ca_Desi_LG07* | 51.299 |
| Ca_Desi_SNP3256 | [A/C] | *Ca_Desi_LG07* | 51.846 |
| Ca_Desi_SNP3257 | [A/C] | *Ca_Desi_LG07* | 51.888 |
| Ca_Desi_SNP3258 | [A/T] | *Ca_Desi_LG07* | 51.984 |
| Ca_Desi_SNP3259 | [A/G] | *Ca_Desi_LG07* | 52.282 |
| Ca_Desi_SNP3260 | [T/G] | *Ca_Desi_LG07* | 52.435 |
| Ca_Desi_SNP3261 | [G/A] | *Ca_Desi_LG07* | 52.596 |

| SNP IDs | SNPs | Linkage Group (LGs) | Genetic positions (cM) of mapped SNPs |
|---|---|---|---|
| Ca_Desi_SNP3262 | [C/A] | *Ca_Desi_LG07* | 53.191 |
| Ca_Desi_SNP3263 | [A/G] | *Ca_Desi_LG07* | 53.442 |
| Ca_Desi_SNP3264 | [G/T] | *Ca_Desi_LG07* | 53.482 |
| Ca_Desi_SNP3265 | [A/C] | *Ca_Desi_LG07* | 53.575 |
| Ca_Desi_SNP3266 | [A/G] | *Ca_Desi_LG07* | 53.596 |
| Ca_Desi_SNP3267 | [A/G] | *Ca_Desi_LG07* | 54.031 |
| Ca_Desi_SNP3268 | [T/G] | *Ca_Desi_LG07* | 54.097 |
| Ca_Desi_SNP3269 | [A/T] | *Ca_Desi_LG07* | 54.679 |
| Ca_Desi_SNP3270 | [T/G] | *Ca_Desi_LG07* | 54.705 |
| Ca_Desi_SNP3271 | [T/G] | *Ca_Desi_LG07* | 54.792 |
| Ca_Desi_SNP3272 | [C/T] | *Ca_Desi_LG07* | 54.802 |
| Ca_Desi_SNP3273 | [C/A] | *Ca_Desi_LG07* | 55.041 |
| Ca_Desi_SNP3274 | [A/C] | *Ca_Desi_LG07* | 55.156 |
| Ca_Desi_SNP3275 | [T/G] | *Ca_Desi_LG07* | 55.228 |
| Ca_Desi_SNP3276 | [T/G] | *Ca_Desi_LG07* | 55.255 |
| Ca_Desi_SNP3277 | [A/C] | *Ca_Desi_LG07* | 56.386 |
| Ca_Desi_SNP3278 | [T/G] | *Ca_Desi_LG07* | 56.559 |
| Ca_Desi_SNP3279 | [A/G] | *Ca_Desi_LG07* | 56.764 |
| Ca_Desi_SNP3280 | [C/G] | *Ca_Desi_LG07* | 56.802 |
| Ca_Desi_SNP3281 | [T/A] | *Ca_Desi_LG07* | 57.275 |
| Ca_Desi_SNP3282 | [A/C] | *Ca_Desi_LG07* | 57.373 |
| Ca_Desi_SNP3283 | [A/G] | *Ca_Desi_LG07* | 57.622 |
| Ca_Desi_SNP3284 | [C/T] | *Ca_Desi_LG07* | 57.719 |
| Ca_Desi_SNP3285 | [A/G] | *Ca_Desi_LG07* | 57.843 |
| Ca_Desi_SNP3286 | [A/G] | *Ca_Desi_LG07* | 57.959 |
| Ca_Desi_SNP3287 | [A/C] | *Ca_Desi_LG07* | 58.030 |
| Ca_Desi_SNP3288 | [A/G] | *Ca_Desi_LG07* | 58.060 |
| Ca_Desi_SNP3289 | [A/G] | *Ca_Desi_LG07* | 58.150 |
| Ca_Desi_SNP3290 | [C/T] | *Ca_Desi_LG07* | 58.197 |
| Ca_Desi_SNP3291 | [T/G] | *Ca_Desi_LG07* | 58.469 |
| Ca_Desi_SNP3292 | [A/G] | *Ca_Desi_LG07* | 58.513 |
| Ca_Desi_SNP3293 | [T/C] | *Ca_Desi_LG07* | 58.553 |
| Ca_Desi_SNP3294 | [A/G] | *Ca_Desi_LG07* | 58.736 |
| Ca_Desi_SNP3295 | [T/C] | *Ca_Desi_LG07* | 59.121 |
| Ca_Desi_SNP3296 | [A/T] | *Ca_Desi_LG07* | 59.198 |
| Ca_Desi_SNP3297 | [T/C] | *Ca_Desi_LG07* | 59.353 |
| Ca_Desi_SNP3298 | [A/G] | *Ca_Desi_LG07* | 59.561 |
| Ca_Desi_SNP3299 | [A/G] | *Ca_Desi_LG07* | 59.719 |
| Ca_Desi_SNP3300 | [A/C] | *Ca_Desi_LG07* | 59.969 |
| Ca_Desi_SNP3301 | [C/T] | *Ca_Desi_LG07* | 60.242 |
| Ca_Desi_SNP3302 | [A/G] | *Ca_Desi_LG07* | 60.316 |
| Ca_Desi_SNP3303 | [A/C] | *Ca_Desi_LG07* | 61.140 |
| Ca_Desi_SNP3304 | [T/C] | *Ca_Desi_LG07* | 61.778 |
| Ca_Desi_SNP3305 | [C/G] | *Ca_Desi_LG07* | 61.887 |
| Ca_Desi_SNP3306 | [A/G] | *Ca_Desi_LG07* | 62.086 |
| Ca_Desi_SNP3307 | [A/G] | *Ca_Desi_LG07* | 62.367 |
| Ca_Desi_SNP3308 | [A/G] | *Ca_Desi_LG07* | 62.484 |
| Ca_Desi_SNP3309 | [C/G] | *Ca_Desi_LG07* | 62.695 |

| **SNP IDs** | **SNPs** | **Linkage Group (LGs)** | **Genetic positions (cM) of mapped SNPs** |
|---|---|---|---|
| Ca_Desi_SNP3310 | [C/T] | *Ca_Desi_LG07* | 62.993 |
| Ca_Desi_SNP3311 | [A/G] | *Ca_Desi_LG07* | 63.055 |
| Ca_Desi_SNP3312 | [A/G] | *Ca_Desi_LG07* | 63.067 |
| Ca_Desi_SNP3313 | [G/A] | *Ca_Desi_LG07* | 63.674 |
| Ca_Desi_SNP3314 | [T/C] | *Ca_Desi_LG07* | 63.792 |
| Ca_Desi_SNP3315 | [A/C] | *Ca_Desi_LG07* | 63.926 |
| Ca_Desi_SNP3316 | [T/C] | *Ca_Desi_LG07* | 64.185 |
| Ca_Desi_SNP3317 | [T/C] | *Ca_Desi_LG07* | 64.304 |
| Ca_Desi_SNP3318 | [A/C] | *Ca_Desi_LG07* | 64.925 |
| Ca_Desi_SNP3319 | [G/A] | *Ca_Desi_LG07* | 64.944 |
| Ca_Desi_SNP3320 | [A/G] | *Ca_Desi_LG07* | 65.266 |
| Ca_Desi_SNP3321 | [T/G] | *Ca_Desi_LG07* | 65.326 |
| Ca_Desi_SNP3322 | [G/A] | *Ca_Desi_LG07* | 65.994 |
| Ca_Desi_SNP3323 | [C/T] | *Ca_Desi_LG07* | 66.154 |
| Ca_Desi_SNP3324 | [C/T] | *Ca_Desi_LG07* | 66.564 |
| Ca_Desi_SNP3325 | [C/A] | *Ca_Desi_LG07* | 67.069 |
| Ca_Desi_SNP3326 | [C/T] | *Ca_Desi_LG07* | 67.259 |
| Ca_Desi_SNP3327 | [T/C] | *Ca_Desi_LG07* | 67.260 |
| Ca_Desi_SNP3328 | [A/G] | *Ca_Desi_LG07* | 67.562 |
| Ca_Desi_SNP3329 | [T/A] | *Ca_Desi_LG07* | 68.509 |
| Ca_Desi_SNP3330 | [A/G] | *Ca_Desi_LG07* | 68.885 |
| Ca_Desi_SNP3331 | [T/A] | *Ca_Desi_LG07* | 68.901 |
| Ca_Desi_SNP3332 | [A/G] | *Ca_Desi_LG07* | 70.137 |
| Ca_Desi_SNP3333 | [C/G] | *Ca_Desi_LG07* | 70.874 |
| Ca_Desi_SNP3334 | [C/T] | *Ca_Desi_LG07* | 71.415 |
| Ca_Desi_SNP3335 | [T/G] | *Ca_Desi_LG07* | 71.902 |
| Ca_Desi_SNP3336 | [C/A] | *Ca_Desi_LG07* | 72.125 |
| Ca_Desi_SNP3337 | [G/A] | *Ca_Desi_LG07* | 72.506 |
| Ca_Desi_SNP3338 | [A/G] | *Ca_Desi_LG07* | 73.168 |
| Ca_Desi_SNP3339 | [A/G] | *Ca_Desi_LG07* | 74.352 |
| Ca_Desi_SNP3340 | [T/C] | *Ca_Desi_LG07* | 75.210 |
| Ca_Desi_SNP3341 | [T/C] | *Ca_Desi_LG07* | 75.253 |
| Ca_Desi_SNP3342 | [T/G] | *Ca_Desi_LG07* | 75.491 |
| Ca_Desi_SNP3343 | [C/T] | *Ca_Desi_LG07* | 77.740 |
| Ca_Desi_SNP3344 | [G/T] | *Ca_Desi_LG07* | 87.821 |
| Ca_Desi_SNP3345 | [C/A] | *Ca_Desi_LG08* | 0.000 |
| Ca_Desi_SNP3346 | [A/G] | *Ca_Desi_LG08* | 0.340 |
| Ca_Desi_SNP3347 | [C/A] | *Ca_Desi_LG08* | 0.603 |
| Ca_Desi_SNP3348 | [A/G] | *Ca_Desi_LG08* | 1.078 |
| Ca_Desi_SNP3349 | [A/G] | *Ca_Desi_LG08* | 1.621 |
| Ca_Desi_SNP3350 | [T/C] | *Ca_Desi_LG08* | 2.357 |
| Ca_Desi_SNP3351 | [A/G] | *Ca_Desi_LG08* | 2.731 |
| Ca_Desi_SNP3352 | [G/A] | *Ca_Desi_LG08* | 3.079 |
| Ca_Desi_SNP3353 | [T/A] | *Ca_Desi_LG08* | 3.922 |
| Ca_Desi_SNP3354 | [G/A] | *Ca_Desi_LG08* | 5.042 |
| Ca_Desi_SNP3355 | [A/G] | *Ca_Desi_LG08* | 5.183 |
| Ca_Desi_SNP3356 | [T/C] | *Ca_Desi_LG08* | 5.705 |
| Ca_Desi_SNP3357 | [A/G] | *Ca_Desi_LG08* | 7.258 |

| **SNP IDs** | **SNPs** | **Linkage Group (LGs)** | **Genetic positions (cM) of mapped SNPs** |
|---|---|---|---|
| Ca_Desi_SNP3358 | [A/G] | *Ca_Desi_LG08* | 8.242 |
| Ca_Desi_SNP3359 | [G/A] | *Ca_Desi_LG08* | 8.558 |
| Ca_Desi_SNP3360 | [G/A] | *Ca_Desi_LG08* | 8.729 |
| Ca_Desi_SNP3361 | [C/A] | *Ca_Desi_LG08* | 8.873 |
| Ca_Desi_SNP3362 | [C/G] | *Ca_Desi_LG08* | 9.540 |
| Ca_Desi_SNP3363 | [T/C] | *Ca_Desi_LG08* | 9.661 |
| Ca_Desi_SNP3364 | [C/A] | *Ca_Desi_LG08* | 10.113 |
| Ca_Desi_SNP3365 | [T/G] | *Ca_Desi_LG08* | 10.320 |
| Ca_Desi_SNP3366 | [A/T] | *Ca_Desi_LG08* | 10.572 |
| Ca_Desi_SNP3367 | [A/C] | *Ca_Desi_LG08* | 10.632 |
| Ca_Desi_SNP3368 | [A/G] | *Ca_Desi_LG08* | 10.879 |
| Ca_Desi_SNP3369 | [A/G] | *Ca_Desi_LG08* | 11.100 |
| Ca_Desi_SNP3370 | [C/T] | *Ca_Desi_LG08* | 11.262 |
| Ca_Desi_SNP3371 | [A/C] | *Ca_Desi_LG08* | 11.366 |
| Ca_Desi_SNP3372 | [A/G] | *Ca_Desi_LG08* | 11.725 |
| Ca_Desi_SNP3373 | [T/G] | *Ca_Desi_LG08* | 11.745 |
| Ca_Desi_SNP3374 | [T/G] | *Ca_Desi_LG08* | 12.047 |
| Ca_Desi_SNP3375 | [G/A] | *Ca_Desi_LG08* | 12.064 |
| Ca_Desi_SNP3376 | [T/C] | *Ca_Desi_LG08* | 13.112 |
| Ca_Desi_SNP3377 | [A/G] | *Ca_Desi_LG08* | 13.656 |
| Ca_Desi_SNP3378 | [T/C] | *Ca_Desi_LG08* | 13.832 |
| Ca_Desi_SNP3379 | [G/A] | *Ca_Desi_LG08* | 14.383 |
| Ca_Desi_SNP3380 | [C/T] | *Ca_Desi_LG08* | 14.916 |
| Ca_Desi_SNP3381 | [T/C] | *Ca_Desi_LG08* | 15.680 |
| Ca_Desi_SNP3382 | [T/C] | *Ca_Desi_LG08* | 16.660 |
| Ca_Desi_SNP3383 | [T/C] | *Ca_Desi_LG08* | 17.169 |
| Ca_Desi_SNP3384 | [G/C] | *Ca_Desi_LG08* | 18.339 |
| Ca_Desi_SNP3385 | [C/T] | *Ca_Desi_LG08* | 18.690 |
| Ca_Desi_SNP3386 | [A/T] | *Ca_Desi_LG08* | 19.091 |
| Ca_Desi_SNP3387 | [T/C] | *Ca_Desi_LG08* | 19.508 |
| Ca_Desi_SNP3388 | [T/C] | *Ca_Desi_LG08* | 19.650 |
| Ca_Desi_SNP3389 | [G/C] | *Ca_Desi_LG08* | 19.651 |
| Ca_Desi_SNP3390 | [G/T] | *Ca_Desi_LG08* | 19.733 |
| Ca_Desi_SNP3391 | [C/G] | *Ca_Desi_LG08* | 20.146 |
| Ca_Desi_SNP3392 | [T/C] | *Ca_Desi_LG08* | 20.174 |
| Ca_Desi_SNP3393 | [G/A] | *Ca_Desi_LG08* | 20.558 |
| Ca_Desi_SNP3394 | [G/C] | *Ca_Desi_LG08* | 20.582 |
| Ca_Desi_SNP3395 | [T/G] | *Ca_Desi_LG08* | 20.724 |
| Ca_Desi_SNP3396 | [G/A] | *Ca_Desi_LG08* | 21.005 |
| Ca_Desi_SNP3397 | [T/C] | *Ca_Desi_LG08* | 21.044 |
| Ca_Desi_SNP3398 | [C/A] | *Ca_Desi_LG08* | 21.049 |
| Ca_Desi_SNP3399 | [A/G] | *Ca_Desi_LG08* | 21.137 |
| Ca_Desi_SNP3400 | [T/G] | *Ca_Desi_LG08* | 21.329 |
| Ca_Desi_SNP3401 | [C/T] | *Ca_Desi_LG08* | 21.382 |
| Ca_Desi_SNP3402 | [C/T] | *Ca_Desi_LG08* | 21.882 |
| Ca_Desi_SNP3403 | [A/T] | *Ca_Desi_LG08* | 21.921 |
| Ca_Desi_SNP3404 | [C/T] | *Ca_Desi_LG08* | 21.949 |
| Ca_Desi_SNP3405 | [T/C] | *Ca_Desi_LG08* | 21.953 |

| SNP IDs | SNPs | Linkage Group (LGs) | Genetic positions (cM) of mapped SNPs |
|---|---|---|---|
| Ca_Desi_SNP3406 | [T/A] | *Ca_Desi_LG08* | 21.971 |
| Ca_Desi_SNP3407 | [G/A] | *Ca_Desi_LG08* | 21.976 |
| Ca_Desi_SNP3408 | [G/T] | *Ca_Desi_LG08* | 22.014 |
| Ca_Desi_SNP3409 | [C/G] | *Ca_Desi_LG08* | 22.207 |
| Ca_Desi_SNP3410 | [T/C] | *Ca_Desi_LG08* | 22.232 |
| Ca_Desi_SNP3411 | [A/G] | *Ca_Desi_LG08* | 22.455 |
| Ca_Desi_SNP3412 | [C/T] | *Ca_Desi_LG08* | 22.610 |
| Ca_Desi_SNP3413 | [A/G] | *Ca_Desi_LG08* | 22.777 |
| Ca_Desi_SNP3414 | [A/C] | *Ca_Desi_LG08* | 22.789 |
| Ca_Desi_SNP3415 | [C/T] | *Ca_Desi_LG08* | 22.790 |
| Ca_Desi_SNP3416 | [G/A] | *Ca_Desi_LG08* | 22.818 |
| Ca_Desi_SNP3417 | [A/C] | *Ca_Desi_LG08* | 22.912 |
| Ca_Desi_SNP3418 | [G/A] | *Ca_Desi_LG08* | 23.656 |
| Ca_Desi_SNP3419 | [T/G] | *Ca_Desi_LG08* | 23.664 |
| Ca_Desi_SNP3420 | [T/A] | *Ca_Desi_LG08* | 23.935 |
| Ca_Desi_SNP3421 | [C/T] | *Ca_Desi_LG08* | 24.262 |
| Ca_Desi_SNP3422 | [C/A] | *Ca_Desi_LG08* | 24.434 |
| Ca_Desi_SNP3423 | [G/A] | *Ca_Desi_LG08* | 24.547 |
| Ca_Desi_SNP3424 | [T/G] | *Ca_Desi_LG08* | 24.855 |
| Ca_Desi_SNP3425 | [T/C] | *Ca_Desi_LG08* | 25.610 |
| Ca_Desi_SNP3426 | [T/G] | *Ca_Desi_LG08* | 25.753 |
| Ca_Desi_SNP3427 | [A/T] | *Ca_Desi_LG08* | 25.756 |
| Ca_Desi_SNP3428 | [G/A] | *Ca_Desi_LG08* | 25.812 |
| Ca_Desi_SNP3429 | [A/G] | *Ca_Desi_LG08* | 26.103 |
| Ca_Desi_SNP3430 | [C/G] | *Ca_Desi_LG08* | 26.439 |
| Ca_Desi_SNP3431 | [G/A] | *Ca_Desi_LG08* | 26.439 |
| Ca_Desi_SNP3432 | [A/G] | *Ca_Desi_LG08* | 26.516 |
| Ca_Desi_SNP3433 | [G/T] | *Ca_Desi_LG08* | 26.835 |
| Ca_Desi_SNP3434 | [T/G] | *Ca_Desi_LG08* | 27.063 |
| Ca_Desi_SNP3435 | [A/G] | *Ca_Desi_LG08* | 27.550 |
| Ca_Desi_SNP3436 | [C/T] | *Ca_Desi_LG08* | 27.788 |
| Ca_Desi_SNP3437 | [G/T] | *Ca_Desi_LG08* | 27.834 |
| Ca_Desi_SNP3438 | [A/G] | *Ca_Desi_LG08* | 27.909 |
| Ca_Desi_SNP3439 | [T/G] | *Ca_Desi_LG08* | 27.981 |
| Ca_Desi_SNP3440 | [C/T] | *Ca_Desi_LG08* | 28.612 |
| Ca_Desi_SNP3441 | [C/G] | *Ca_Desi_LG08* | 28.997 |
| Ca_Desi_SNP3442 | [C/G] | *Ca_Desi_LG08* | 29.105 |
| Ca_Desi_SNP3443 | [A/C] | *Ca_Desi_LG08* | 29.244 |
| Ca_Desi_SNP3444 | [G/A] | *Ca_Desi_LG08* | 29.389 |
| Ca_Desi_SNP3445 | [A/G] | *Ca_Desi_LG08* | 29.455 |
| Ca_Desi_SNP3446 | [T/G] | *Ca_Desi_LG08* | 30.042 |
| Ca_Desi_SNP3447 | [A/C] | *Ca_Desi_LG08* | 30.299 |
| Ca_Desi_SNP3448 | [T/C] | *Ca_Desi_LG08* | 30.430 |
| Ca_Desi_SNP3449 | [T/C] | *Ca_Desi_LG08* | 30.550 |
| Ca_Desi_SNP3450 | [A/C] | *Ca_Desi_LG08* | 30.629 |
| Ca_Desi_SNP3451 | [A/G] | *Ca_Desi_LG08* | 31.181 |
| Ca_Desi_SNP3452 | [T/A] | *Ca_Desi_LG08* | 32.666 |
| Ca_Desi_SNP3453 | [A/T] | *Ca_Desi_LG08* | 32.873 |

| **SNP IDs** | **SNPs** | **Linkage Group (LGs)** | **Genetic positions (cM) of mapped SNPs** |
|---|---|---|---|
| Ca_Desi_SNP3454 | [C/G] | *Ca_Desi_LG08* | 34.621 |
| Ca_Desi_SNP3455 | [A/G] | *Ca_Desi_LG08* | 34.668 |
| Ca_Desi_SNP3456 | [T/G] | *Ca_Desi_LG08* | 34.738 |
| Ca_Desi_SNP3457 | [T/G] | *Ca_Desi_LG08* | 35.325 |
| Ca_Desi_SNP3458 | [T/C] | *Ca_Desi_LG08* | 35.810 |
| Ca_Desi_SNP3459 | [G/A] | *Ca_Desi_LG08* | 36.055 |
| Ca_Desi_SNP3460 | [A/G] | *Ca_Desi_LG08* | 36.209 |
| Ca_Desi_SNP3461 | [A/G] | *Ca_Desi_LG08* | 36.253 |
| Ca_Desi_SNP3462 | [T/G] | *Ca_Desi_LG08* | 36.340 |
| Ca_Desi_SNP3463 | [G/A] | *Ca_Desi_LG08* | 36.542 |
| Ca_Desi_SNP3464 | [C/T] | *Ca_Desi_LG08* | 36.803 |
| Ca_Desi_SNP3465 | [C/T] | *Ca_Desi_LG08* | 37.290 |
| Ca_Desi_SNP3466 | [A/C] | *Ca_Desi_LG08* | 37.963 |
| Ca_Desi_SNP3467 | [A/G] | *Ca_Desi_LG08* | 38.257 |
| Ca_Desi_SNP3468 | [T/G] | *Ca_Desi_LG08* | 38.628 |
| Ca_Desi_SNP3469 | [A/G] | *Ca_Desi_LG08* | 38.657 |
| Ca_Desi_SNP3470 | [T/G] | *Ca_Desi_LG08* | 38.824 |
| Ca_Desi_SNP3471 | [T/C] | *Ca_Desi_LG08* | 39.091 |
| Ca_Desi_SNP3472 | [C/T] | *Ca_Desi_LG08* | 39.843 |
| Ca_Desi_SNP3473 | [T/C] | *Ca_Desi_LG08* | 40.210 |
| Ca_Desi_SNP3474 | [A/C] | *Ca_Desi_LG08* | 40.402 |
| Ca_Desi_SNP3475 | [T/G] | *Ca_Desi_LG08* | 40.777 |
| Ca_Desi_SNP3476 | [A/G] | *Ca_Desi_LG08* | 40.793 |
| Ca_Desi_SNP3477 | [A/C] | *Ca_Desi_LG08* | 41.009 |
| Ca_Desi_SNP3478 | [T/G] | *Ca_Desi_LG08* | 41.031 |
| Ca_Desi_SNP3479 | [A/T] | *Ca_Desi_LG08* | 41.254 |
| Ca_Desi_SNP3480 | [A/G] | *Ca_Desi_LG08* | 41.740 |
| Ca_Desi_SNP3481 | [A/G] | *Ca_Desi_LG08* | 42.006 |
| Ca_Desi_SNP3482 | [G/A] | *Ca_Desi_LG08* | 42.361 |
| Ca_Desi_SNP3483 | [A/C] | *Ca_Desi_LG08* | 42.377 |
| Ca_Desi_SNP3484 | [A/C] | *Ca_Desi_LG08* | 42.485 |
| Ca_Desi_SNP3485 | [A/G] | *Ca_Desi_LG08* | 42.492 |
| Ca_Desi_SNP3486 | [A/G] | *Ca_Desi_LG08* | 42.569 |
| Ca_Desi_SNP3487 | [T/G] | *Ca_Desi_LG08* | 43.068 |
| Ca_Desi_SNP3488 | [T/C] | *Ca_Desi_LG08* | 43.329 |
| Ca_Desi_SNP3489 | [A/C] | *Ca_Desi_LG08* | 43.764 |
| Ca_Desi_SNP3490 | [C/T] | *Ca_Desi_LG08* | 44.015 |
| Ca_Desi_SNP3491 | [T/G] | *Ca_Desi_LG08* | 44.194 |
| Ca_Desi_SNP3492 | [A/T] | *Ca_Desi_LG08* | 44.663 |
| Ca_Desi_SNP3493 | [A/G] | *Ca_Desi_LG08* | 44.882 |
| Ca_Desi_SNP3494 | [G/A] | *Ca_Desi_LG08* | 45.071 |
| Ca_Desi_SNP3495 | [C/G] | *Ca_Desi_LG08* | 45.440 |
| Ca_Desi_SNP3496 | [T/C] | *Ca_Desi_LG08* | 45.879 |
| Ca_Desi_SNP3497 | [C/A] | *Ca_Desi_LG08* | 45.989 |
| Ca_Desi_SNP3498 | [G/T] | *Ca_Desi_LG08* | 46.059 |
| Ca_Desi_SNP3499 | [A/C] | *Ca_Desi_LG08* | 46.342 |
| Ca_Desi_SNP3500 | [C/T] | *Ca_Desi_LG08* | 46.352 |
| Ca_Desi_SNP3501 | [C/T] | *Ca_Desi_LG08* | 47.010 |

| **SNP IDs** | **SNPs** | **Linkage Group (LGs)** | **Genetic positions (cM) of mapped SNPs** |
|---|---|---|---|
| Ca_Desi_SNP3502 | [T/C] | *Ca_Desi_LG08* | 47.164 |
| Ca_Desi_SNP3503 | [G/A] | *Ca_Desi_LG08* | 47.438 |
| Ca_Desi_SNP3504 | [A/G] | *Ca_Desi_LG08* | 47.454 |
| Ca_Desi_SNP3505 | [A/C] | *Ca_Desi_LG08* | 47.750 |
| Ca_Desi_SNP3506 | [T/A] | *Ca_Desi_LG08* | 47.947 |
| Ca_Desi_SNP3507 | [C/A] | *Ca_Desi_LG08* | 48.426 |
| Ca_Desi_SNP3508 | [C/A] | *Ca_Desi_LG08* | 48.559 |
| Ca_Desi_SNP3509 | [T/A] | *Ca_Desi_LG08* | 49.495 |
| Ca_Desi_SNP3510 | [A/G] | *Ca_Desi_LG08* | 49.591 |
| Ca_Desi_SNP3511 | [T/G] | *Ca_Desi_LG08* | 49.635 |
| Ca_Desi_SNP3512 | [T/C] | *Ca_Desi_LG08* | 49.676 |
| Ca_Desi_SNP3513 | [G/A] | *Ca_Desi_LG08* | 49.792 |
| Ca_Desi_SNP3514 | [G/C] | *Ca_Desi_LG08* | 49.829 |
| Ca_Desi_SNP3515 | [T/C] | *Ca_Desi_LG08* | 49.938 |
| Ca_Desi_SNP3516 | [T/C] | *Ca_Desi_LG08* | 50.067 |
| Ca_Desi_SNP3517 | [T/C] | *Ca_Desi_LG08* | 50.074 |
| Ca_Desi_SNP3518 | [T/C] | *Ca_Desi_LG08* | 50.174 |
| Ca_Desi_SNP3519 | [A/C] | *Ca_Desi_LG08* | 50.202 |
| Ca_Desi_SNP3520 | [T/G] | *Ca_Desi_LG08* | 50.204 |
| Ca_Desi_SNP3521 | [A/C] | *Ca_Desi_LG08* | 50.549 |
| Ca_Desi_SNP3522 | [A/T] | *Ca_Desi_LG08* | 50.617 |
| Ca_Desi_SNP3523 | [C/A] | *Ca_Desi_LG08* | 50.623 |
| Ca_Desi_SNP3524 | [T/C] | *Ca_Desi_LG08* | 51.029 |
| Ca_Desi_SNP3525 | [G/T] | *Ca_Desi_LG08* | 51.542 |
| Ca_Desi_SNP3526 | [G/T] | *Ca_Desi_LG08* | 51.556 |
| Ca_Desi_SNP3527 | [A/G] | *Ca_Desi_LG08* | 51.751 |
| Ca_Desi_SNP3528 | [G/C] | *Ca_Desi_LG08* | 51.842 |
| Ca_Desi_SNP3529 | [A/C] | *Ca_Desi_LG08* | 51.862 |
| Ca_Desi_SNP3530 | [C/A] | *Ca_Desi_LG08* | 52.211 |
| Ca_Desi_SNP3531 | [C/G] | *Ca_Desi_LG08* | 52.456 |
| Ca_Desi_SNP3532 | [A/T] | *Ca_Desi_LG08* | 52.468 |
| Ca_Desi_SNP3533 | [A/C] | *Ca_Desi_LG08* | 53.645 |
| Ca_Desi_SNP3534 | [C/A] | *Ca_Desi_LG08* | 53.727 |
| Ca_Desi_SNP3535 | [T/G] | *Ca_Desi_LG08* | 53.793 |
| Ca_Desi_SNP3536 | [T/G] | *Ca_Desi_LG08* | 54.055 |
| Ca_Desi_SNP3537 | [T/C] | *Ca_Desi_LG08* | 54.212 |
| Ca_Desi_SNP3538 | [A/G] | *Ca_Desi_LG08* | 54.218 |
| Ca_Desi_SNP3539 | [A/G] | *Ca_Desi_LG08* | 54.457 |
| Ca_Desi_SNP3540 | [C/A] | *Ca_Desi_LG08* | 54.497 |
| Ca_Desi_SNP3541 | [C/G] | *Ca_Desi_LG08* | 54.553 |
| Ca_Desi_SNP3542 | [A/T] | *Ca_Desi_LG08* | 54.593 |
| Ca_Desi_SNP3543 | [T/C] | *Ca_Desi_LG08* | 54.839 |
| Ca_Desi_SNP3544 | [A/G] | *Ca_Desi_LG08* | 54.904 |
| Ca_Desi_SNP3545 | [C/T] | *Ca_Desi_LG08* | 55.002 |
| Ca_Desi_SNP3546 | [C/T] | *Ca_Desi_LG08* | 55.050 |
| Ca_Desi_SNP3547 | [A/C] | *Ca_Desi_LG08* | 55.244 |
| Ca_Desi_SNP3548 | [A/G] | *Ca_Desi_LG08* | 55.939 |
| Ca_Desi_SNP3549 | [C/G] | *Ca_Desi_LG08* | 56.131 |

| SNP IDs | SNPs | Linkage Group (LGs) | Genetic positions (cM) of mapped SNPs |
|---|---|---|---|
| Ca_Desi_SNP3550 | [G/A] | *Ca_Desi_LG08* | 56.287 |
| Ca_Desi_SNP3551 | [A/G] | *Ca_Desi_LG08* | 56.294 |
| Ca_Desi_SNP3552 | [C/G] | *Ca_Desi_LG08* | 56.429 |
| Ca_Desi_SNP3553 | [A/C] | *Ca_Desi_LG08* | 56.482 |
| Ca_Desi_SNP3554 | [T/G] | *Ca_Desi_LG08* | 56.577 |
| Ca_Desi_SNP3555 | [T/G] | *Ca_Desi_LG08* | 57.041 |
| Ca_Desi_SNP3556 | [A/G] | *Ca_Desi_LG08* | 57.270 |
| Ca_Desi_SNP3557 | [T/C] | *Ca_Desi_LG08* | 57.292 |
| Ca_Desi_SNP3558 | [T/G] | *Ca_Desi_LG08* | 57.626 |
| Ca_Desi_SNP3559 | [T/G] | *Ca_Desi_LG08* | 57.855 |
| Ca_Desi_SNP3560 | [A/G] | *Ca_Desi_LG08* | 57.861 |
| Ca_Desi_SNP3561 | [T/A] | *Ca_Desi_LG08* | 58.066 |
| Ca_Desi_SNP3562 | [T/G] | *Ca_Desi_LG08* | 58.139 |
| Ca_Desi_SNP3563 | [T/A] | *Ca_Desi_LG08* | 59.089 |
| Ca_Desi_SNP3564 | [T/A] | *Ca_Desi_LG08* | 59.130 |
| Ca_Desi_SNP3565 | [T/C] | *Ca_Desi_LG08* | 59.490 |
| Ca_Desi_SNP3566 | [G/A] | *Ca_Desi_LG08* | 59.519 |
| Ca_Desi_SNP3567 | [A/G] | *Ca_Desi_LG08* | 59.585 |
| Ca_Desi_SNP3568 | [G/A] | *Ca_Desi_LG08* | 59.613 |
| Ca_Desi_SNP3569 | [A/G] | *Ca_Desi_LG08* | 59.913 |
| Ca_Desi_SNP3570 | [G/T] | *Ca_Desi_LG08* | 59.921 |
| Ca_Desi_SNP3571 | [T/C] | *Ca_Desi_LG08* | 60.149 |
| Ca_Desi_SNP3572 | [C/T] | *Ca_Desi_LG08* | 60.533 |
| Ca_Desi_SNP3573 | [T/A] | *Ca_Desi_LG08* | 60.605 |
| Ca_Desi_SNP3574 | [C/T] | *Ca_Desi_LG08* | 60.784 |
| Ca_Desi_SNP3575 | [A/G] | *Ca_Desi_LG08* | 60.871 |
| Ca_Desi_SNP3576 | [A/G] | *Ca_Desi_LG08* | 60.894 |
| Ca_Desi_SNP3577 | [G/A] | *Ca_Desi_LG08* | 60.958 |
| Ca_Desi_SNP3578 | [G/A] | *Ca_Desi_LG08* | 61.006 |
| Ca_Desi_SNP3579 | [G/A] | *Ca_Desi_LG08* | 61.503 |
| Ca_Desi_SNP3580 | [A/C] | *Ca_Desi_LG08* | 61.786 |
| Ca_Desi_SNP3581 | [T/C] | *Ca_Desi_LG08* | 61.989 |
| Ca_Desi_SNP3582 | [A/G] | *Ca_Desi_LG08* | 62.020 |
| Ca_Desi_SNP3583 | [T/C] | *Ca_Desi_LG08* | 62.119 |
| Ca_Desi_SNP3584 | [C/T] | *Ca_Desi_LG08* | 62.240 |
| Ca_Desi_SNP3585 | [A/G] | *Ca_Desi_LG08* | 62.590 |
| Ca_Desi_SNP3586 | [T/C] | *Ca_Desi_LG08* | 62.997 |
| Ca_Desi_SNP3587 | [A/G] | *Ca_Desi_LG08* | 63.151 |
| Ca_Desi_SNP3588 | [T/C] | *Ca_Desi_LG08* | 63.406 |
| Ca_Desi_SNP3589 | [A/G] | *Ca_Desi_LG08* | 63.731 |
| Ca_Desi_SNP3590 | [A/G] | *Ca_Desi_LG08* | 63.815 |
| Ca_Desi_SNP3591 | [T/G] | *Ca_Desi_LG08* | 63.850 |
| Ca_Desi_SNP3592 | [T/G] | *Ca_Desi_LG08* | 64.099 |
| Ca_Desi_SNP3593 | [A/G] | *Ca_Desi_LG08* | 64.489 |
| Ca_Desi_SNP3594 | [G/T] | *Ca_Desi_LG08* | 64.662 |
| Ca_Desi_SNP3595 | [A/G] | *Ca_Desi_LG08* | 64.671 |
| Ca_Desi_SNP3596 | [A/G] | *Ca_Desi_LG08* | 64.776 |
| Ca_Desi_SNP3597 | [G/A] | *Ca_Desi_LG08* | 64.925 |

| **SNP IDs** | **SNPs** | **Linkage Group (LGs)** | **Genetic positions (cM) of mapped SNPs** |
|---|---|---|---|
| Ca_Desi_SNP3598 | [C/A] | *Ca_Desi_LG08* | 65.180 |
| Ca_Desi_SNP3599 | [A/T] | *Ca_Desi_LG08* | 65.229 |
| Ca_Desi_SNP3600 | [T/G] | *Ca_Desi_LG08* | 65.498 |
| Ca_Desi_SNP3601 | [A/G] | *Ca_Desi_LG08* | 65.499 |
| Ca_Desi_SNP3602 | [A/G] | *Ca_Desi_LG08* | 65.529 |
| Ca_Desi_SNP3603 | [T/A] | *Ca_Desi_LG08* | 65.657 |
| Ca_Desi_SNP3604 | [A/G] | *Ca_Desi_LG08* | 66.029 |
| Ca_Desi_SNP3605 | [G/T] | *Ca_Desi_LG08* | 66.290 |
| Ca_Desi_SNP3606 | [T/C] | *Ca_Desi_LG08* | 66.881 |
| Ca_Desi_SNP3607 | [A/G] | *Ca_Desi_LG08* | 67.785 |
| Ca_Desi_SNP3608 | [A/G] | *Ca_Desi_LG08* | 67.838 |
| Ca_Desi_SNP3609 | [A/G] | *Ca_Desi_LG08* | 68.508 |
| Ca_Desi_SNP3610 | [T/G] | *Ca_Desi_LG08* | 69.895 |
| Ca_Desi_SNP3611 | [C/A] | *Ca_Desi_LG08* | 69.976 |
| Ca_Desi_SNP3612 | [A/G] | *Ca_Desi_LG08* | 70.526 |
| Ca_Desi_SNP3613 | [T/C] | *Ca_Desi_LG08* | 71.006 |
| Ca_Desi_SNP3614 | [T/G] | *Ca_Desi_LG08* | 71.265 |
| Ca_Desi_SNP3615 | [T/C] | *Ca_Desi_LG08* | 71.606 |
| Ca_Desi_SNP3616 | [C/T] | *Ca_Desi_LG08* | 71.844 |
| Ca_Desi_SNP3617 | [A/C] | *Ca_Desi_LG08* | 73.520 |
| Ca_Desi_SNP3618 | [A/C] | *Ca_Desi_LG08* | 76.736 |
| Ca_Desi_SNP3619 | [T/G] | *Ca_Desi_LG08* | 77.683 |
| Ca_Desi_SNP3620 | [A/G] | *Ca_Desi_LG08* | 78.156 |
| Ca_Desi_SNP3621 | [G/A] | *Ca_Desi_LG08* | 78.509 |
| Ca_Desi_SNP3622 | [T/C] | *Ca_Desi_LG08* | 79.375 |
| Ca_Desi_SNP3623 | [A/T] | *Ca_Desi_LG08* | 80.766 |
| Ca_Desi_SNP3624 | [A/C] | *Ca_Desi_LG08* | 81.334 |
| Ca_Desi_SNP3625 | [T/C] | *Ca_Desi_LG08* | 89.085 |

## Table S2: Accessions selected for PH trait association mapping

| Sl. No. | Accession Numbers | Cultivar types | Geographical origin | Biological status |
|---|---|---|---|---|
| 1 | ICC5590 | *Desi* | India | Breeding/Research material |
| 2 | ICC6013 | *Desi* | India | Breeding/Research material |
| 3 | ICC5002 | *Desi* | India | Breeding/Research material |
| 4 | ICC7184 | *Desi* | Turkey | Traditional cultivar/Landrace |
| 5 | ICC4926 | *Desi* | India | Breeding/Research material |
| 6 | ICC4657 | *Desi* | India | Traditional cultivar/Landrace |
| 7 | ICC12299 | *Desi* | Nepal | Traditional cultivar/Landrace |
| 8 | ICC456 | *Desi* | India | Traditional cultivar/Landrace |
| 9 | ICC12726 | *Desi* | Ethiopia | Traditional cultivar/Landrace |
| 10 | ICC11944 | *Desi* | Nepal | Traditional cultivar/Landrace |
| 11 | ICC11498 | *Desi* | India | Traditional cultivar/Landrace |
| 12 | ICC9942 | *Desi* | India | Traditional cultivar/Landrace |
| 13 | ICC2072 | *Desi* | India | Traditional cultivar/Landrace |
| 14 | ICC9737 | *Desi* | Afghanistan | Traditional cultivar/Landrace |
| 15 | ICC9002 | *Desi* | Iran | Traditional cultivar/Landrace |
| 16 | ICC16374 | *Desi* | Malawi | Breeding material |
| 17 | ICC12028 | *Desi* | Mexico | Traditional cultivar/Landrace |
| 18 | ICC8318 | *Desi* | India | Traditional cultivar/Landrace |
| 19 | ICC15610 | *Desi* | India | Traditional cultivar/Landrace |
| 20 | ICC4918 | *Desi* | India | Advanced cultivar |
| 21 | ICC1836 | *Desi* | India | Traditional cultivar/Landrace |
| 22 | ICC15061 | *Desi* | India | Traditional cultivar/Landrace |
| 23 | ICC13523 | *Kabuli* | Iran | Traditional cultivar/Landrace |
| 24 | ICC15802 | *Kabuli* | Syria | Traditional cultivar/Landrace |
| 25 | ICC13077 | *Kabuli* | India | Traditional cultivar/Landrace |
| 26 | ICC15264 | *Kabuli* | Iran | Traditional cultivar/Landrace |
| 27 | ICC7308 | *Kabuli* | Peru | Traditional cultivar/Landrace |
| 28 | ICC15435 | *Kabuli* | Morocco | Traditional cultivar/Landrace |
| 29 | ICC7295 | *Kabuli* | Tunisia | Traditional cultivar/Landrace |
| 30 | ICC12328 | *Kabuli* | Cyprus | Traditional cultivar/Landrace |
| 31 | ICC14446 | *Kabuli* | Italy | Traditional cultivar/Landrace |
| 32 | ICC10755 | *Kabuli* | Turkey | Traditional cultivar/Landrace |
| 33 | ICC14190 | *Kabuli* | India | Traditional cultivar/Landrace |
| 34 | ICC8042 | *Kabuli* | Iran | Traditional cultivar/Landrace |
| 35 | ICC8261 | *Kabuli* | Turkey | Traditional cultivar/Landrace |
| 36 | ICC15333 | *Kabuli* | Iran | Traditional cultivar/Landrace |
| 37 | ICC15512 | *Kabuli* | Morocco | Traditional cultivar/Landrace |
| 38 | ICC10884 | *Kabuli* | Ethiopia | Traditional cultivar/Landrace |
| 39 | ICC15551 | *Kabuli* | Australia | Traditional cultivar/Landrace |
| 40 | ICC15725 | *Kabuli* | Syrian Arab Republic | Traditional cultivar/Landrace |
| 41 | ICC6204 | *Kabuli* | Spain | Traditional cultivar/Landrace |
| 42 | ICC6210 | *Kabuli* | Spain | Traditional cultivar/Landrace |
| 43 | ICC7654 | *Kabuli* | Turkey | Traditional cultivar/Landrace |
| 44 | ICC11847 | *Kabuli* | Chile | Traditional cultivar/Landrace |
| 45 | ICC11749 | *Kabuli* | Chile | Traditional cultivar/Landrace |
| 46 | ICC14199 | *Kabuli* | Mexico | Breeding material |
| 47 | ICC15518 | *Kabuli* | Morocco | Traditional cultivar/Landrace |
| 48 | ICC16814 | *Kabuli* | Portugal | Traditional cultivar/Landrace |

| Sl. No. | Accession Numbers | Cultivar types | Geographical origin | Biological status |
|---|---|---|---|---|
| 49 | ICC16811 | *Kabuli* | Portugal | Traditional cultivar/Landrace |
| 50 | ICC6253 | *Kabuli* | Morocco | Traditional cultivar/Landrace |
| 51 | ICC11301 | *Kabuli* | United States of America | Traditional cultivar/Landrace |
| 52 | ICC10749 | *Kabuli* | Turkey | Traditional cultivar/Landrace |
| 53 | ICC14216 | *Kabuli* | Mexico | Breeding/Research material |
| 54 | ICC14203 | *Kabuli* | Mexico | Traditional cultivar/Landrace |
| 55 | ICC11742 | *Kabuli* | Chile | Traditional cultivar/Landrace |
| 56 | ICC13821 | *Kabuli* | Ethiopia | Traditional cultivar/Landrace |
| 57 | ICC14462 | *Kabuli* | India | Traditional cultivar/Landrace |
| 58 | ICC14220 | *Kabuli* | Kenya | Traditional cultivar/Landrace |
| 59 | ICC15944 | *Kabuli* | United States of America | Advanced/Improved cultivar |
| 60 | ICC11303 | *Kabuli* | Chile | Traditional cultivar/Landrace |
| 61 | ICC12034 | *Kabuli* | Mexico | Advanced/Improved cultivar |
| 62 | ICC7346 | *Kabuli* | Mexico | Breeding/Research material |
| 63 | ICC15994 | *Kabuli* | Spain | Traditional cultivar/Landrace |
| 64 | ICC18591 | *Kabuli* | Mexico | Traditional cultivar/Landrace |
| 65 | ICC8155 | *Kabuli* | United States of America | Traditional cultivar/Landrace |
